# Supplementary material for: Cistrome and transcriptome analysis identifies unique androgen receptor (AR) and AR-V7 splice variant chromatin binding and transcriptional activities
Source: Sci Rep. 2022 Mar 30;12:5351. doi: 10.1038/s41598-022-09371-x (PMC8969163; doi:10.1038/s41598-022-09371-x)
Supplement: Supplementary file 1 — Supplementary Information. [file 41598_2022_9371_MOESM1_ESM.pdf]

Supplementary Methods

ChIP analyses

ChIP assays were performed from nuclear extracts prepared in a water bath sonicator (Bioruptor from Diagenode, Denville, NJ). Samples were sonicated with 10 cycles of 30 seconds on and 30 seconds off each in a final volume of 1 mL. 5% of the lysate was reserved as input and the remainder was pre-cleared with 25 µL of Protein A/G Magnetic Agarose (Pierce, Thermo Scientific, Waltham, MA) conditioned with 20 µg/mL salmon sperm DNA. Lysates were split into equal portions and immunoprecipitations were performed with 50 µL of Magnetic Agarose using 2 µg of rabbit anti-mouse IgG (Invitrogen), 2 µg of AR antibody (ActiveMotif, Carlsbad, CA) or AR C-19 (Santa Cruz Biotechnology, Dallas, TX). Eluted ChIP fractions were quantified by qPCR and normalised using the primer sequences listed in STable 1.

ChIP-exo datasets in STable 1 were used to map AR and AR-V7 interaction with DNA at single base resolution. Cells in CSS were treated for 24 hours with 10 nM R1881 (LNCaP) or 20 ng/mL Dox (LNCaP AR-V7). Cells were fixed in 1% formaldehyde. Six independent experiments were performed, and cells shipped to ActiveMotif where like samples were combined and ChIP-exo performed using the ActiveMotif kit. ChIP-exo libraries were prepared using the TruSeq ChIP library preparation kit from Illumina, San Diego, CA and sequenced on an Illumina HiSeq 2000 platform.

ChIP-seq data analysis

ChIP-seq and ChIP-exo raw reads were aligned to the human reference genome from UCSC genome (hg19) by the bowtie2 (version 2.3, default parameters) alignment software [55]. Duplicate reads were ignored while calling peaks using macs2 (version 2.1; -*q* <.05, otherwise mentioned) with input as control when available [56]. Peaks overlapping (at least 1bp) with the “hg19 blacklist regions” reported by the ENCODE project [61] were also removed. Peaks were annotated as AR variant specific or common peaks based on their overlap in genomic coordinates (at least 1bp). Annotation function in HOMER (version 4.10) [62] was used to assign peaks to genes. In-house scripts and BEDTools (version 2.28.0) [63] were used to annotate proximal promoter (±100 bp from

transcription start site, TSS) and distal enhancer peaks (upstream to transcription start site, without interfering genes and -100bp to TSS) using the hg19 RefSeq annotation as reference. Genes with either a promoter or enhancer peak were considered to be potential AR isoform targets and target genes with differential expression as possible direct targets. ChIP-seq intensities and read coverage were calculated (at each 10 bp bin, by extending 100 bp) from aligned reads at the reported peak regions. Average ChIP-seq intensities within ±1000 bp of peaks were plotted as profile and heatmaps using deepTools [64]. Overrepresentation of motif sequences in the peak locations were identified by the software HOMER, within 200 bp of reported peak summits.

RNA-seq data generation

RNA-seq datasets in STable 1 were used to probe the AR and AR-V7 transcriptome. In short, RNA-seq was performed using LN95 cells treated with either: 10 nM R1881 or an equivalent volume of the vehicle control. For knock-down experiments cells were treated with control siRNA, siRNA targeting exon7 or siRNA targeting cryptic exon of AR-V7 for 48 hours before harvesting. LNCaP $^{AR\text{-}V7/pLenti}$ cell samples [26] and VCaP AR-V7 were treated with either: 10 nM R1881, 0.25 ng/mL Dox, or an equivalent volume of the vehicle control. RNA was isolated After 24 hours. RNeasy column purification kit (Qiagen, MD) was used according to the manufacturer's protocol to extract RNA from all samples. The clean RNA samples having RIN more than 8.0 were used for library preparation. Library preparation was performed with the TruSeq™ RNA Sample Prep Kit v1 (Illumina, San Diego, CA, Cat#FC-122-100). LN95 cDNA libraries were sequenced on the Illumina NextSeq-500 or NovaSeq-6000 platform, LNCaP cDNA libraries were sequenced on the Hiseq 2500 platform, Illumina, San Diego, CA in the Genomic and RNA Profiling Core facility, Baylor College of Medicine and VCaP cDNA libraries were sequenced on the NextSeq 500 platform, (Illumina) by the University of Houston Sequencing core.

RNA-seq data analysis

RNA-seq data were used to identify the AR and AR-V7 transcriptome. Raw read sequences from fastq files for each sample were trimmed using Trim Galore (https://github.com/FelixKrueger/TrimGalore). After quality trimming, using a Phred score of 20, the sequences for each sample were aligned to the prebuilt hg19 genome from UCSC using HISAT2 [57] using default parameters. Aligned reads for each sample were then quantified using featureCounts [59] and a transcript annotation reference for the hg19 genome build (V24). Differential gene expression analysis of the protein coding genes was then performed using the Bioconductor package edgeR [60]. A change in expression for a protein coding gene was considered significant if it had a BH-adjusted p-value of less than or equal to 0.05. Gene signatures were generated by filtering the results for statistically significant genes that had at least the indicated log2 fold change in expression. Ranked gene lists of all the detected genes were created for each comparison using the log2 fold change results from the differential gene expression analysis. Gene set enrichment analysis (GSEA) of these ranked gene lists was then performed against the entire molecular signature database (MSigDB) using the java based GSEA software developed by UC San Diego and the Broad Institute [65]. Plots are not proportional unless specifically indicated.

Analysis of public datasets

We utilised public gene expression datasets and ChIP datasets listed in STable 1 for comparisons in this study. LNCaP ABL [28] and C4-2B [27] datasets were used for aberrant AR activation signature in prostate cancer cells expressing only AR. Datasets from 22RV1 were used to compare AR and AR-V7 transcriptomes in endogenous models [17,19]. Transcriptome dataset of FOXA1 KD in LNCaP cells was also used in this study. GEO2R function in the GEO database was used with BH multiple testing correction and other default parameters to test differential gene expression. Raw RNA-seq datasets from 22RV1 were also downloaded and analysed as previously mentioned. Other public ChIP datasets used in this study are also listed in STable 1. These include AR ChIP-exo data from LNCaP cells treated with vehicle control, FOXA1 ChIP-exo data from LNCaP cells

treated with DHT and HOXB13 ChIP-seq data from LNCaP cells in 10% FBS. Raw sequence data was processed as per the previous description.

Supplementary Figure 1

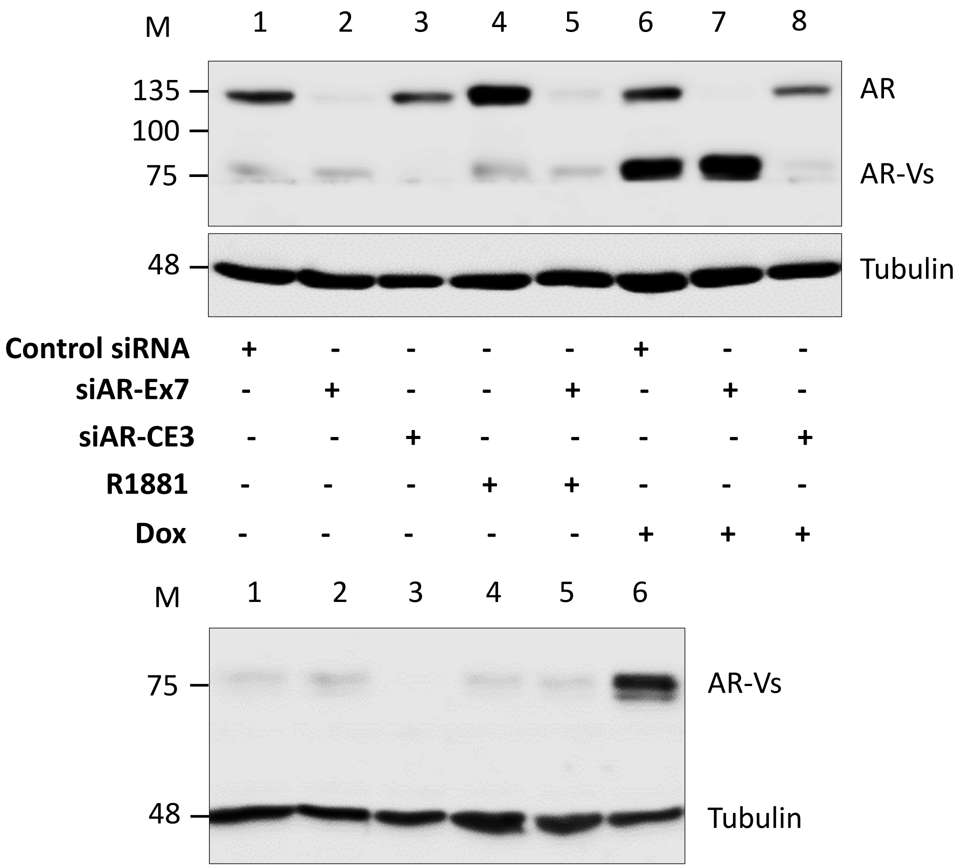

Supplementary Figure 1. AR variant expression in LNCaP AR-V7 cells. Cells in 10% CSS medium were treated with the indicated siRNAs for 72 hours, protein lysates prepared, separated by SDS gel electrophoresis and AR isoforms detected using AR441 (top) and AR-V7 antibodies (bottom). Lanes represent LNCaP AR-V7 cells treated with 1. Control siRNA, 2. siRNA against AR (Exon 7), 3. siRNA against AR-V7 (CE3), 4. 1 nM R1881, 5. siRNA against AR (exon 7) + 1 nM R1881, 6. Control siRNA + 20 ng/ml Dox, 7. siRNA against AR (Exon 7) + 20 ng/ml Dox. 8. siRNA against AR-V7 (CE3) + 20 ng/ml Dox.

Supplementary Figure 2

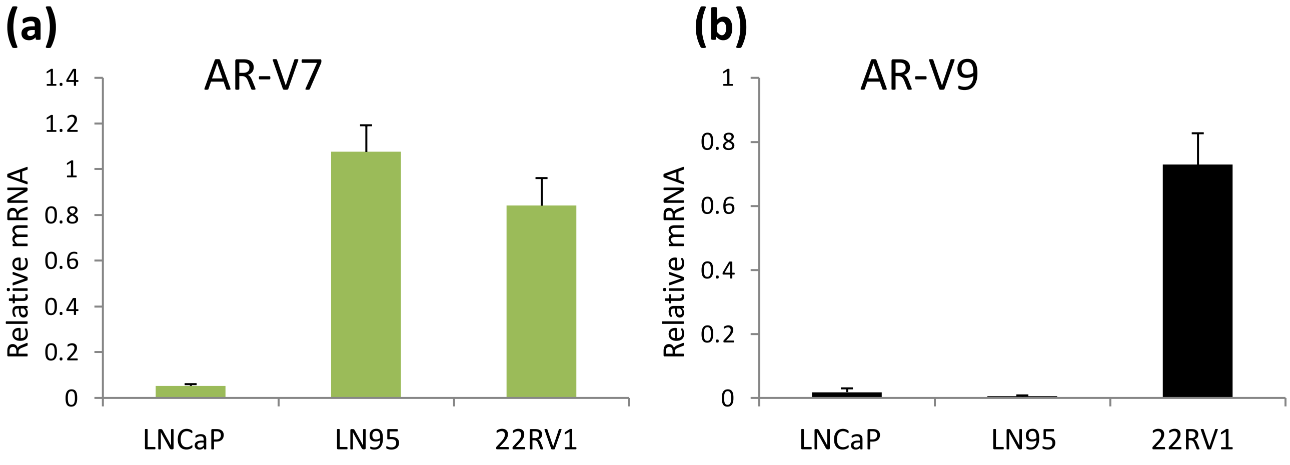

Supplementary Figure 2. AR-V9 expression in different cell lines. Cells were transferred to medium containing CSS, harvested after 24 hours and RNA isolated for qPCR analyses. **a**. AR-V7 expression measured using qPCR in LNCaP, LN95 and 22RV1 cells. **b**. AR-V9 expression measured using qPCR in LNCaP, LN95 and 22RV1 cells.

Supplementary Figure 3

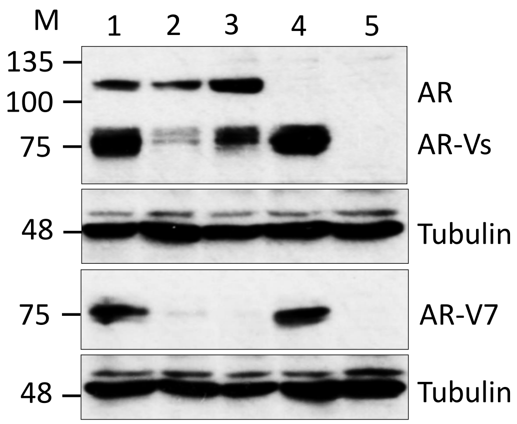

Supplementary Figure 3. AR variant expression in 22RV1 cells. Cells in CSS medium were treated with the indicated siRNAs for 72 hours, protein lysates prepared, separated by SDS gel electrophoresis and AR isoforms detected using AR441 (top) and AR-V7 antibodies (bottom). Lanes represent 22RV1 cells treated with 1. Control siRNA, 2. siRNA against AR-V7 II, 3. siRNA against AR-V7 III, 4. siRNA against AR (exon 7), 5. siRNA against all AR isoforms (exon 1).

Supplementary figure 4

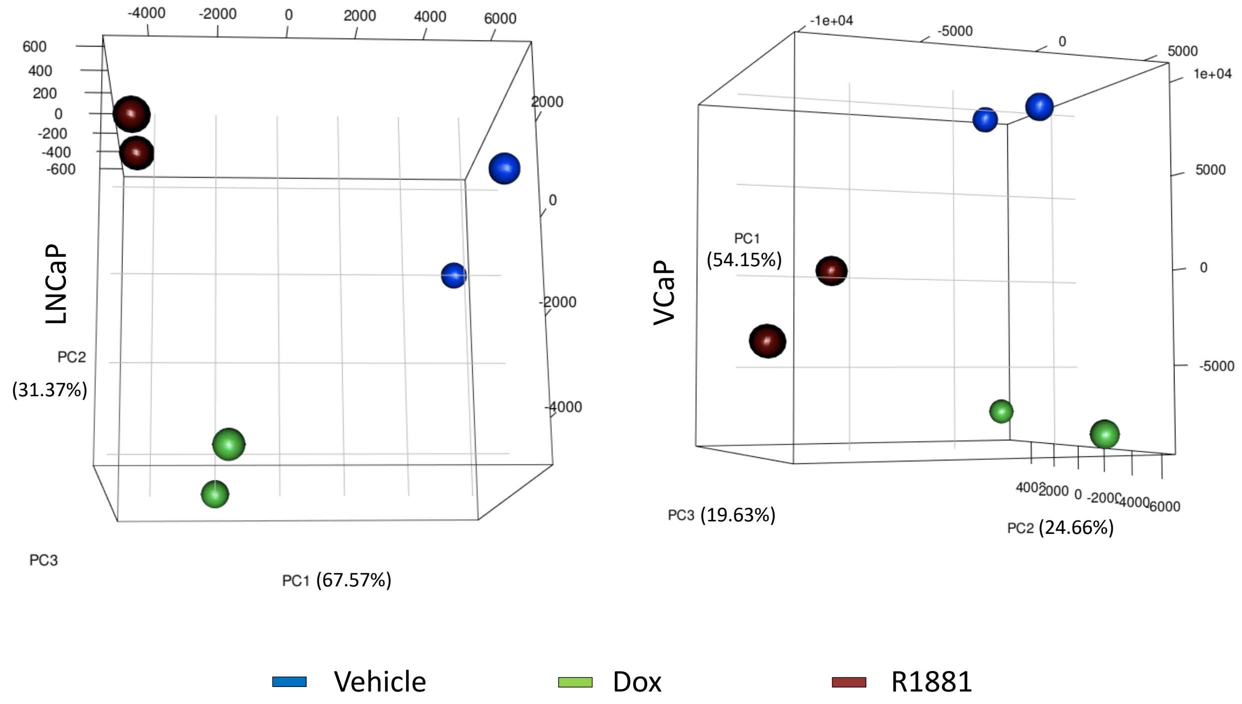

Supplementary Figure 4. Principal component analysis for LNCaP and VCaP RNA-Seq datasets. First three components are plotted, and they explained most of the variability. Vehicle treated, Dox treated and R1881 treated samples show a clear separation from each other. Replicates also behave similarly although there is some variation between samples.

Supplementary figure 5

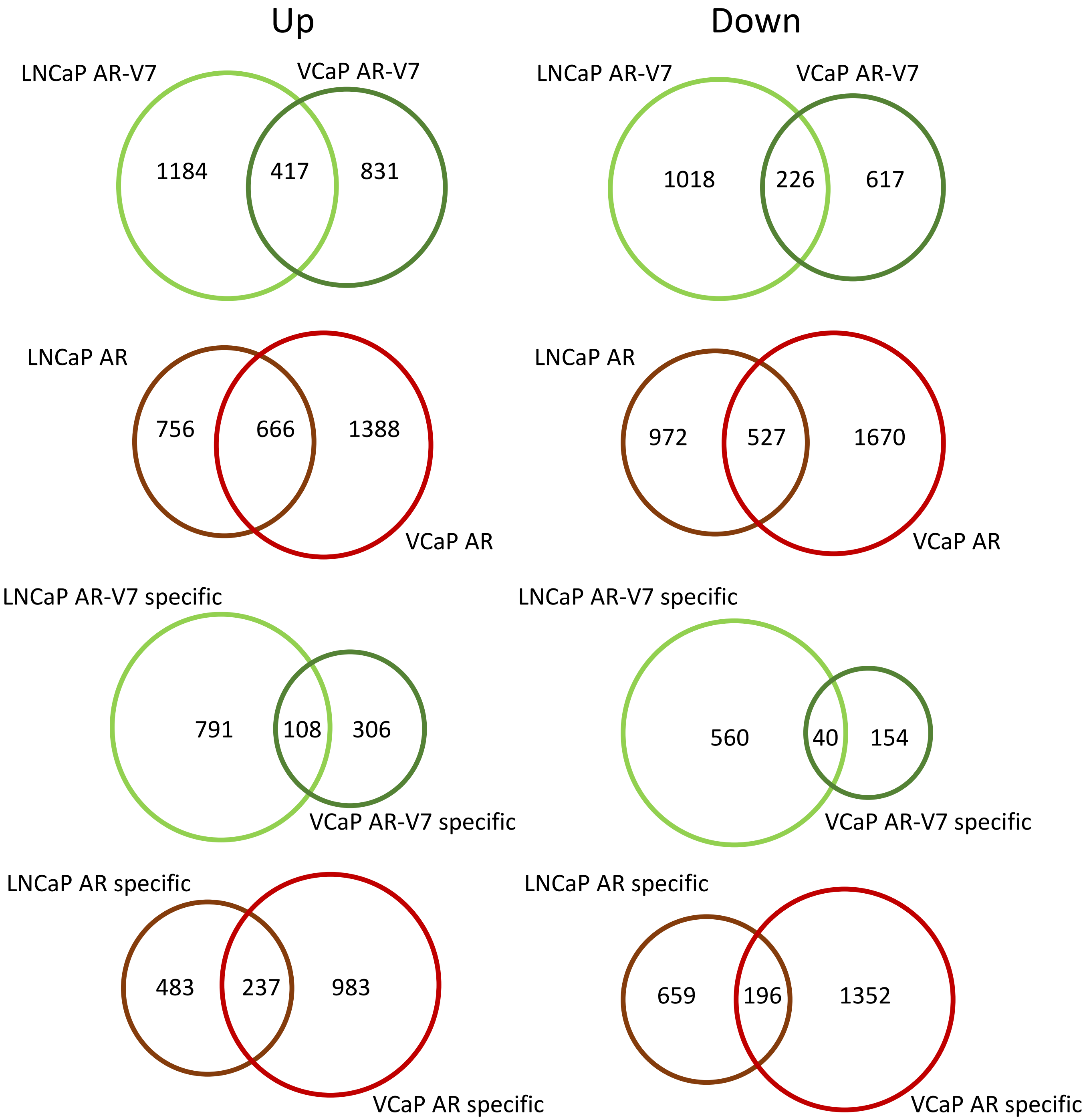

Supplementary Figure 5. Comparison of AR isoform mediated gene expression in the LNCaP and VCaP models. Venn diagrams were prepared to show the overlap between genes regulated by the AR isoforms in the two inducible models. Upper portion: Comparison of all genes up or down regulated by AR or AR-V7 at least 1.5 fold adjusted p <0.05. Lower portion: A comparison of genes specifically regulated by one isoform, but not the other. Venn diagrams are plotted as circles proportional to the numbers.

Supplementary Figure 6

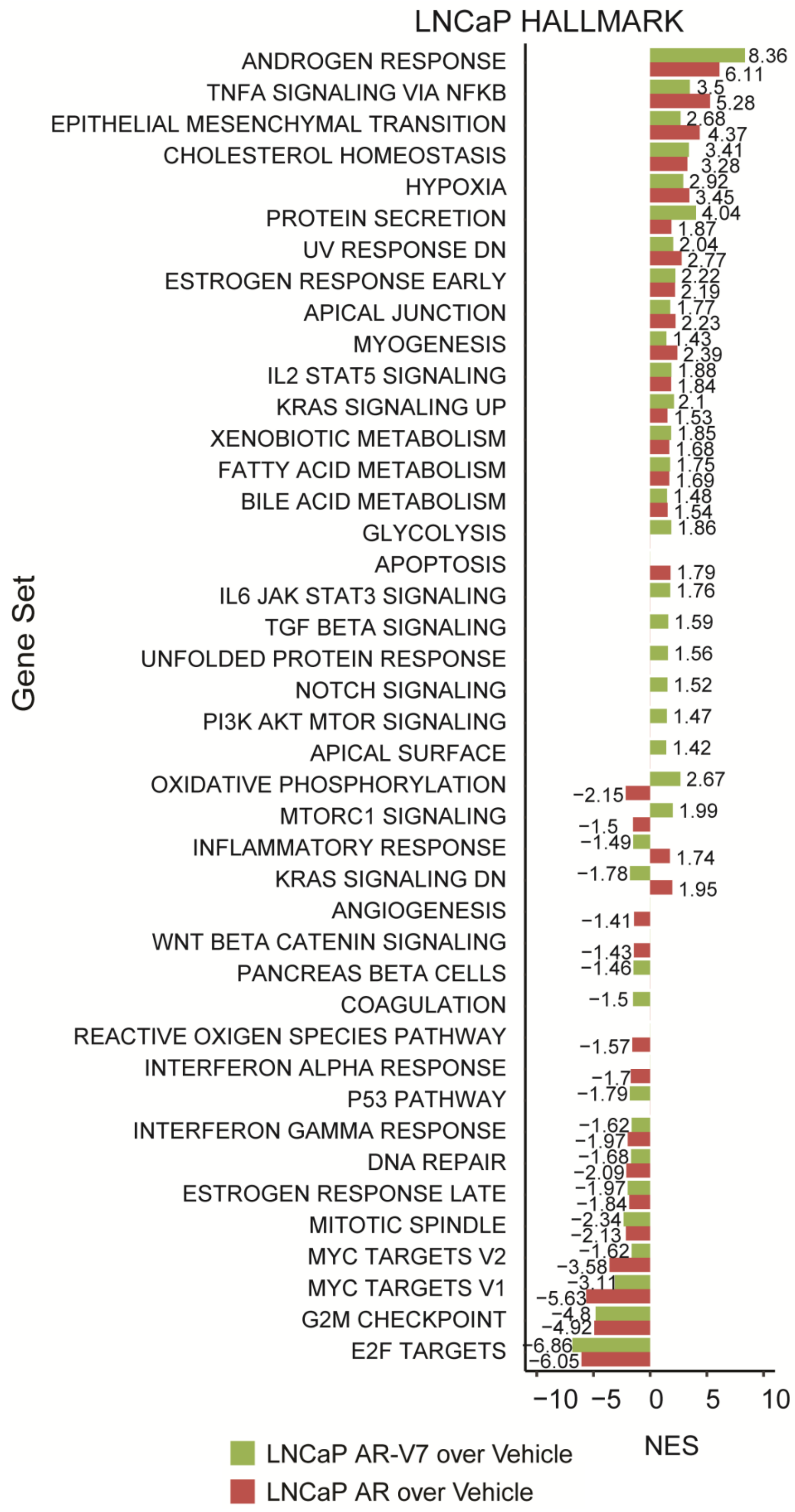

Supplementary Figure 6. Hallmark pathways regulated in LNCaP AR-V7 cells. All Hallmark pathways significantly regulated by AR isoforms in the LNCaP model. Rank is determined by AR-V7 value.

Supplementary Figure 7

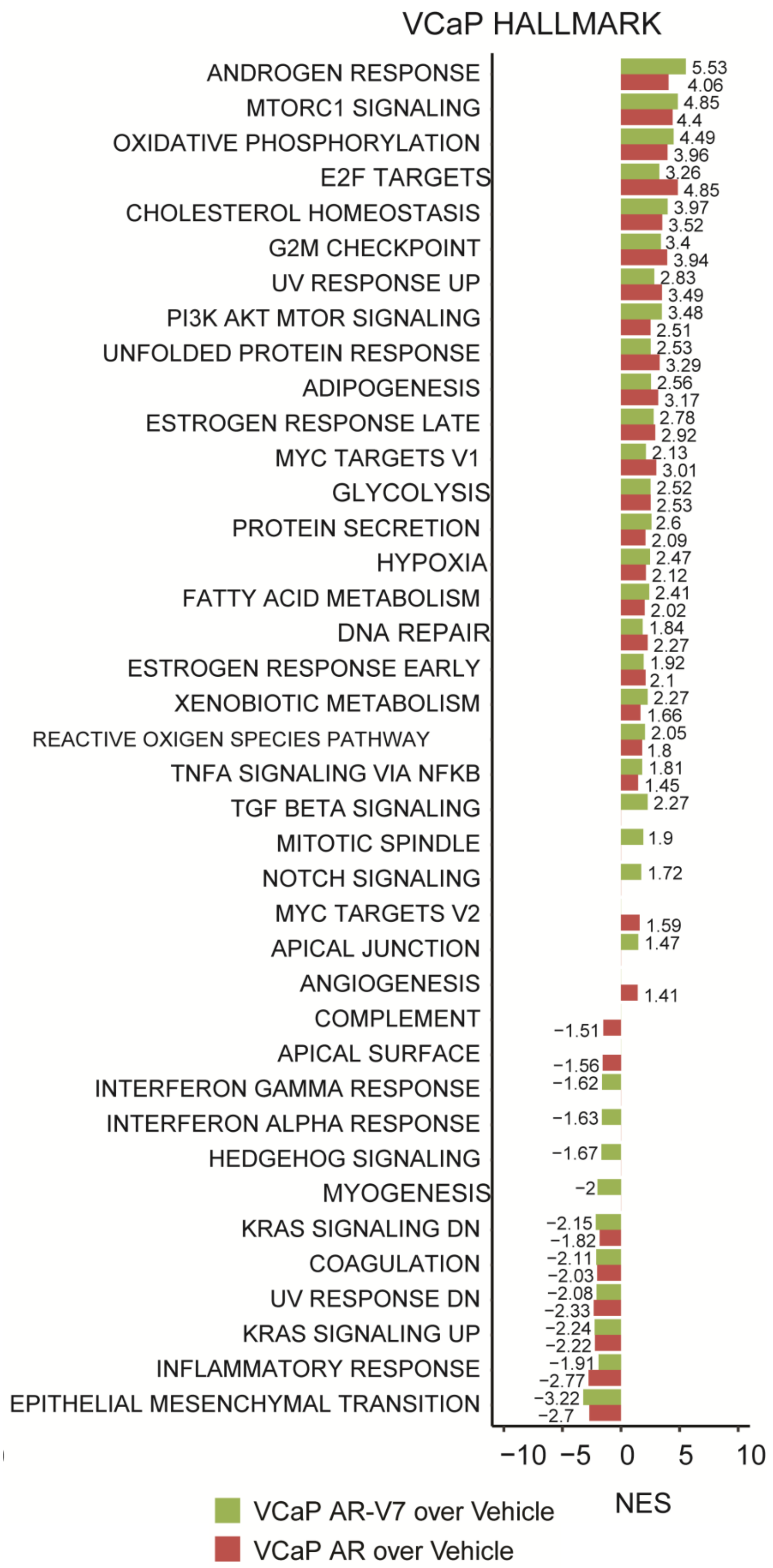

Supplementary Figure 7. Hallmark pathways regulated in VCaP AR-V7 cells. All Hallmark pathways significantly regulated by AR isoforms in the VCaP model. Rank is determined by AR-V7 value

Supplementary figure 8

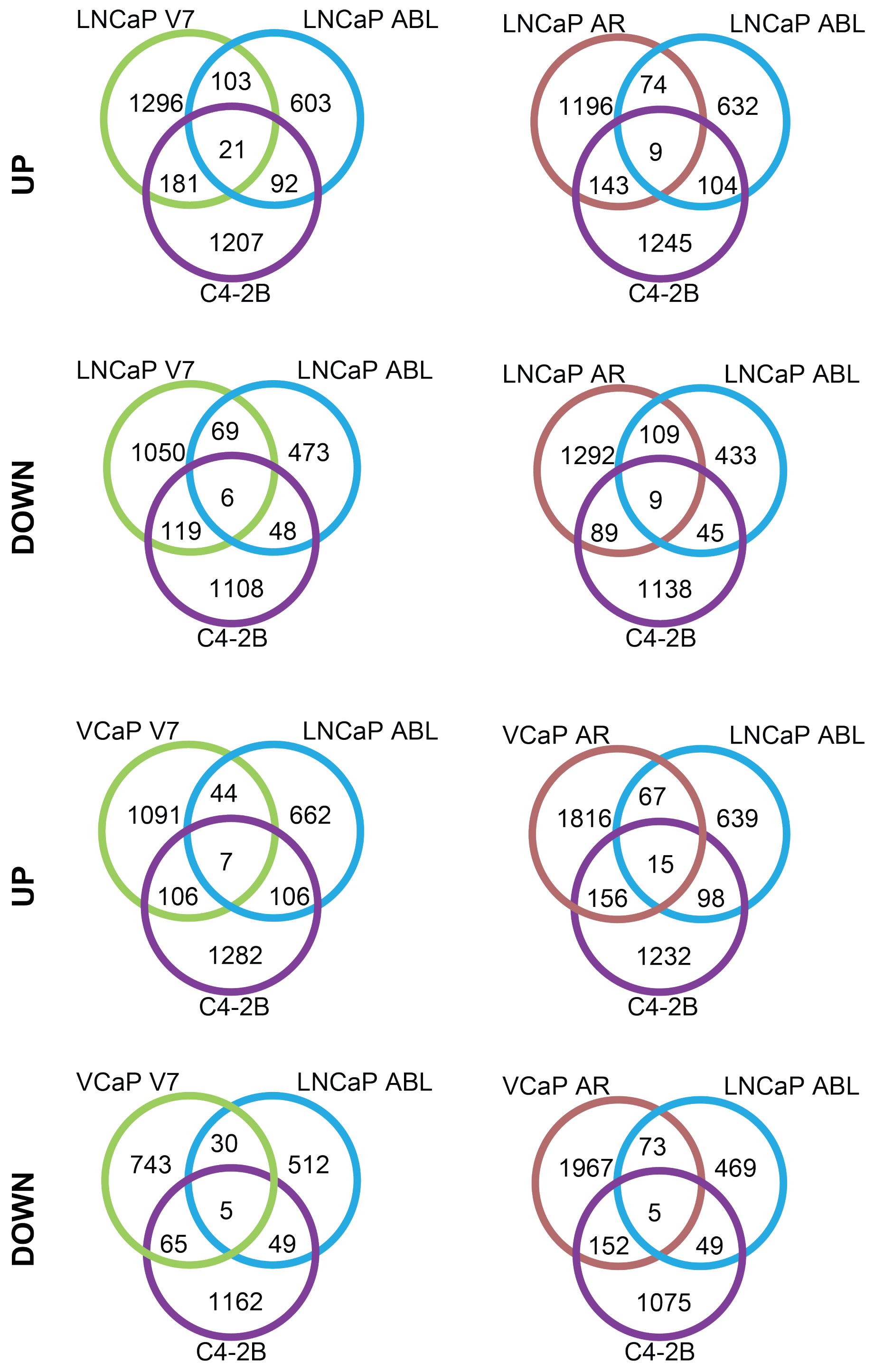

Supplementary Figure 8. Comparison of Gene Expression in LNCaP AR-V7 and VCaP AR-V7 cells with LNCaP ABL and C4-2B cells. All Genes up or down regulated by AR or AR-V7 in the two inducible models were compared with genes regulated by depletion of AR in the LNCaP ABL and C4-2B models. Only those genes with 1.5 fold regulation and $p<0.05$ were included. Left, AR-V7 comparisons; Right, AR comparisons. Venn diagrams are plotting symmetrical circles and not proportionate to numbers.

Supplementary Figure 9

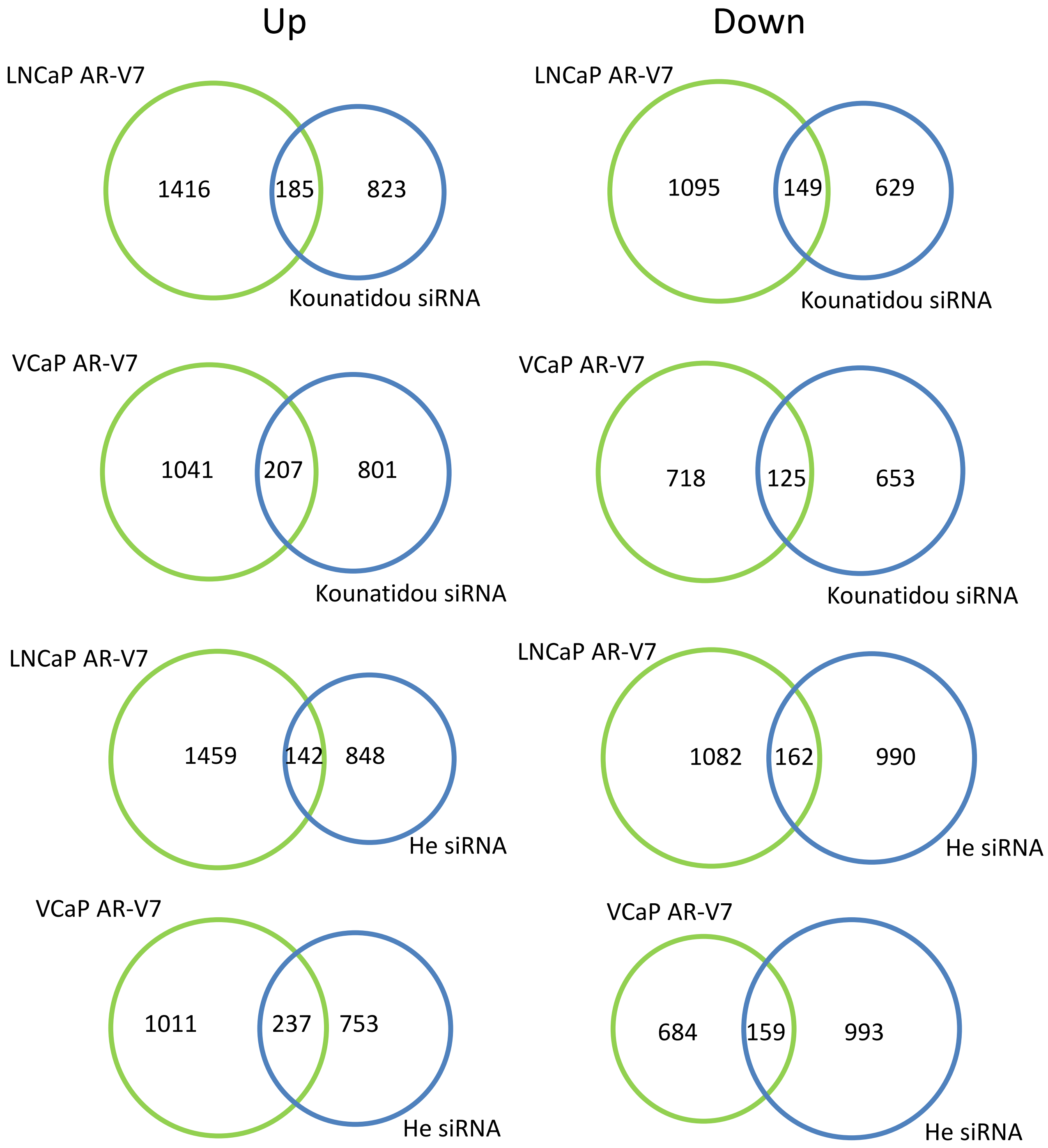

Supplementary Figure 9. Comparison of genes regulated in the LNCaP AR-V7 and VCaP AR-V7 models with genes regulated in 22RV1 models. Venn diagrams showing the overlaps between AR-V7 up or down-regulated genes from the engineered models with genes regulated by variants in two separate data sets in 22RV1 cells derived from depletion of variants using mixed siRNAs targeting variants(He) or by eliminating full length AR CRISPR followed by depletion of all AR variants using siRNA targeting exon 1 (Kounatidou). Venn diagrams are plotted as circles proportional to the numbers.

Supplementary figure 10

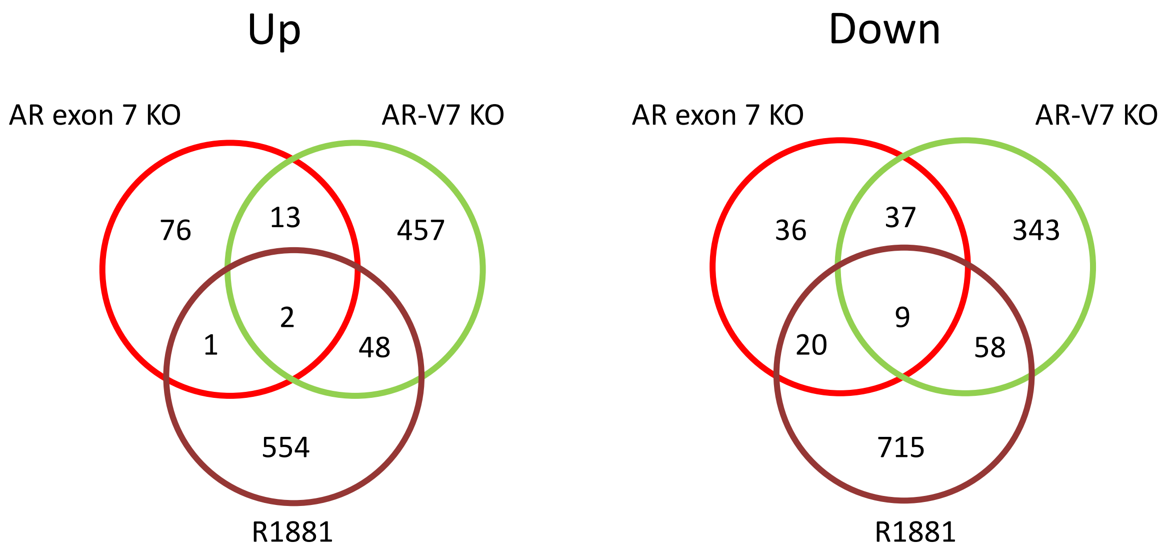

Supplementary Figure 10. Comparison of genes regulated by AR-V7, AR, and R1881 in LN95 cells RNA was isolated from LN-95 cells depleted of AR (si-exon 7), or AR-V7 (siCE3) or control siRNA. To identify R1881 dependent genes, cells were treated with vehicle or 10 nM R1881 and all samples were subjected to RNA-Seq. AR exon 7 KO (control si/exon 7si); AR-V7 KO (controlsi/CE3 si). R1881 (R1881/vehicle). Only those genes with 1.5 fold regulation and $p<0.05$ were included. Two biological replicates prepared in separate weeks were used for each treatment condition. Venn diagrams are plotting symmetrical circles and not proportionate to numbers.

Supplementary figure 11

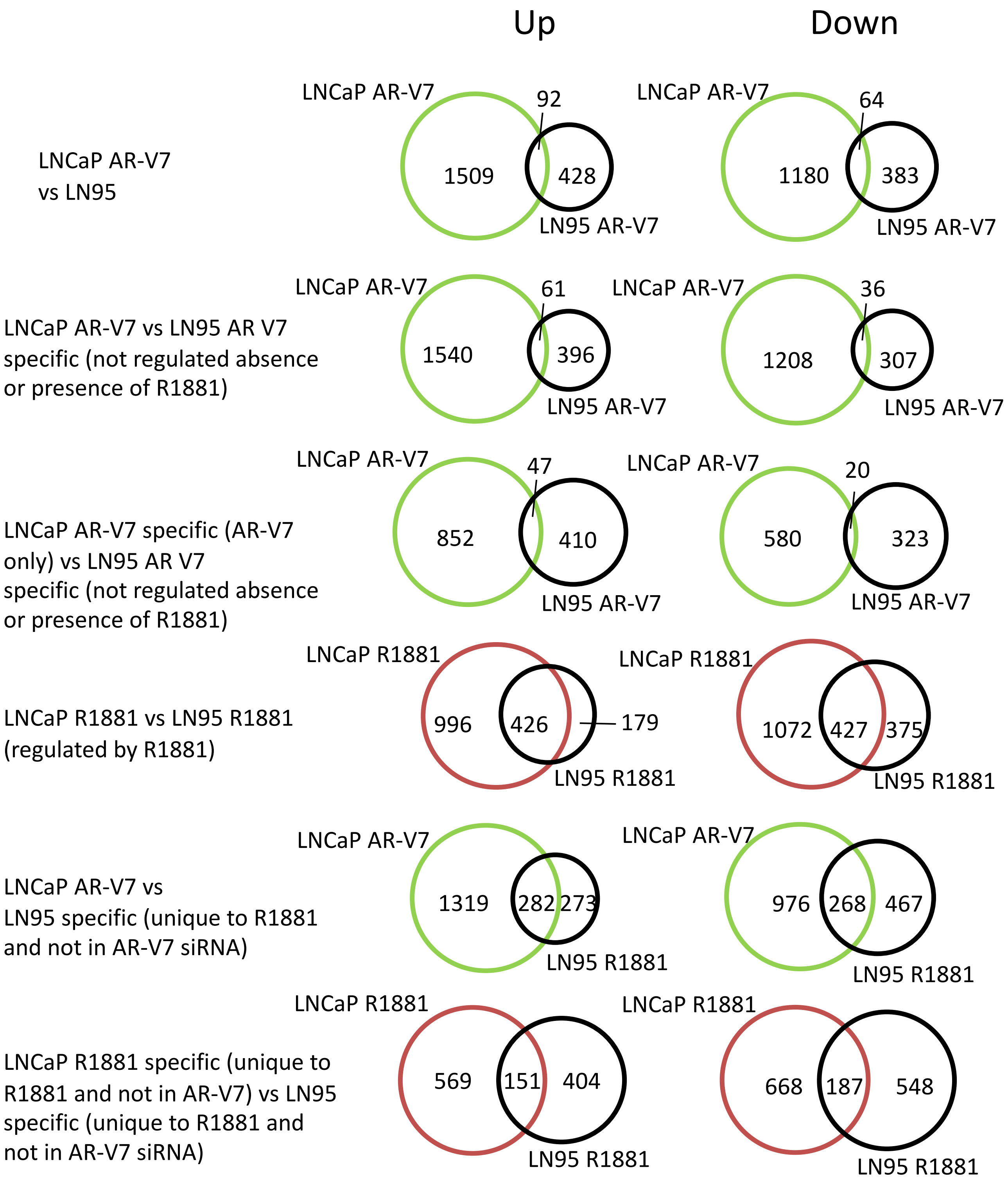

Supplementary Figure 11. Comparison of genes regulated in the LN95 model and in the LNCaP AR-V7 model. Genes from the sets described in SFig. 10 were compared to total AR-V7 regulated genes in both models or AR-V7 specific genes in both models. Total and specific R1881 regulated genes in both models also were compared. Finally, total AR-V7 regulated genes in the LNCaP model were compared with R1881 regulated genes in the LN95 model. Venn diagrams are plotted as circles proportional to the numbers.

Supplementary Figure 12

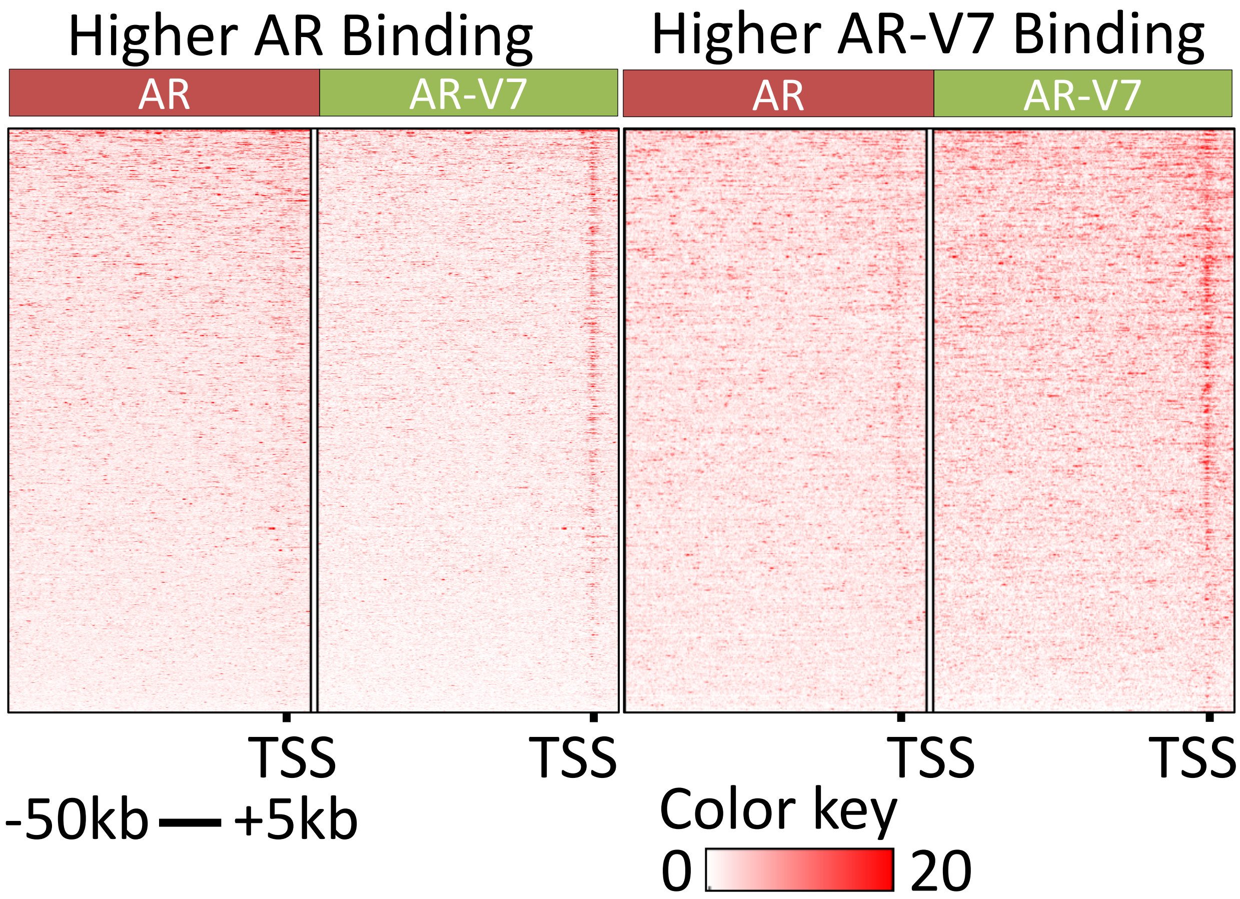

Supplementary Figure 12. AR and AR-V7 Exhibit Differential Binding Patterns. Characterization of Transcription factor binding at Transcription Start Sites in the Human Genome. A. Average ChIP-exo signal (normalized read counts) for AR and AR-V7 at TSS plotted as a profile within 50 kb upstream and 5 kb downstream. ChIP-exo signal > 4-fold is considered as higher binding.

Supplementary Figure 13

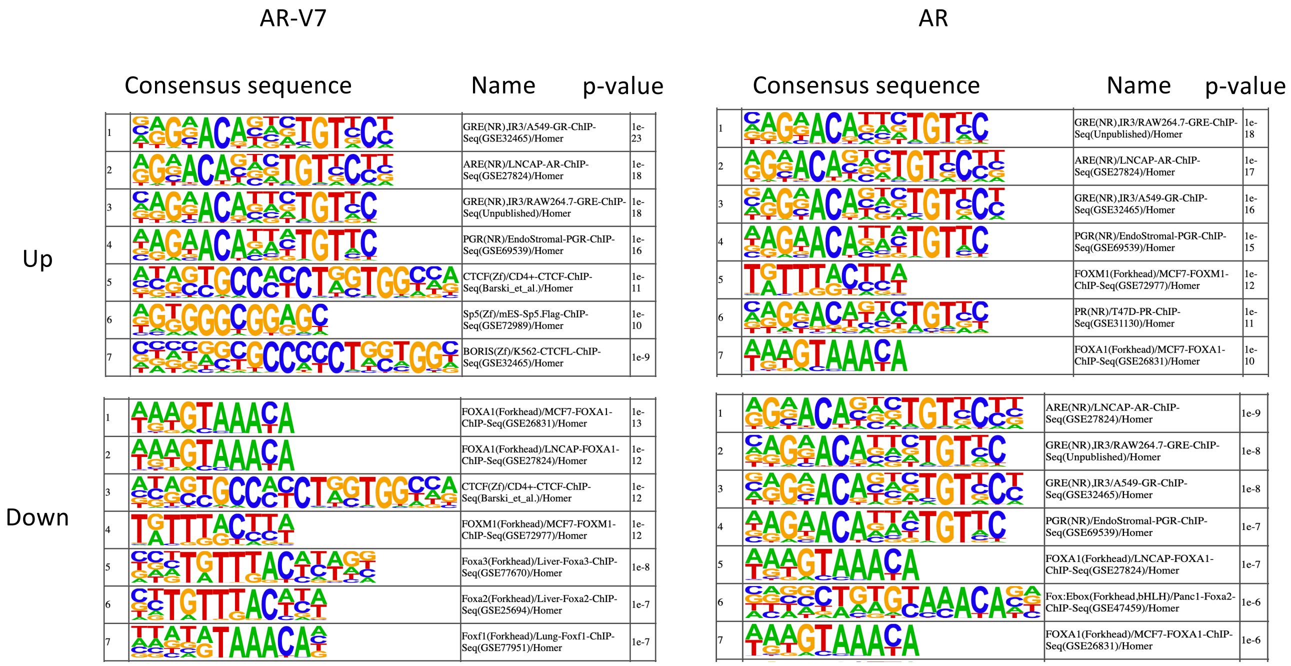

Supplementary Figure 13. Consensus motifs enriched at AR and AR-V7 binding sites near TSS of DEGs. Consensus motif sequences identified in a motif analysis using ARBs and ARVBs within 100bpof TSS of up and down DEGs. Homer findmotifsGenome.pl, -factor and other default parameters (target peaks vs background $p < 0.05$) were used.

Supplementary Figure 14

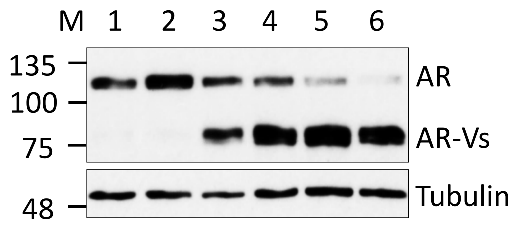

Supplementary Figure 14. Induction of AR-V7 by Doxycycline. LNCaP AR-V7 cells in CSS were treated with vehicle, 10 nM R1881, or the indicated doses of doxycycline for 24 hours, harvested, and AR isoforms detected by western blotting with AR 441 antibody and β Tubulin antibody. Note that the shorter exposure needed to portray the high levels of induction does not detect the low basal level of AR-V7 seen in the absence of Doxycyline. Lanes represent LNCaP AR-V7 cells treated with 1. Ethanol, 2. 10 nM R1881, 3-6. Doxycycline final concentration 3. Doxycycline 20 ng/mL, 4. Doxycycline 40 ng/mL, 5. Doxycycline 80 ng/mL, 6. Doxycycline 120 ng/mL.

Supplementary Figure 15

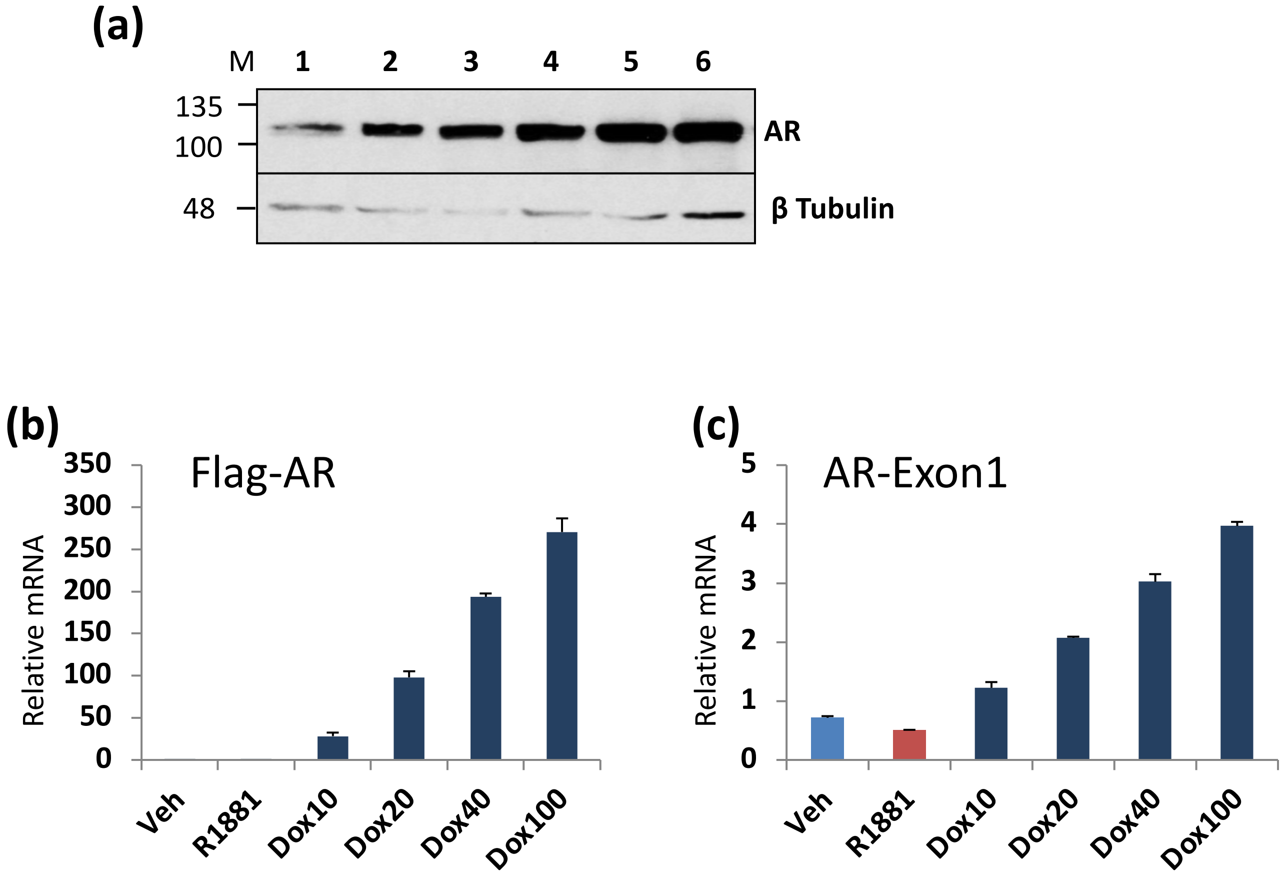

Supplementary Figure 15. Doxycycline induced overexpression of AR. The flag AR line in CSS was treated with vehicle (0.1% ethanol), 10 nM R1881 or the indicated doses of Doxycyclne for 24 hours, proteins isolated for western analyses, and RNA for mRNA expression. **a**. Western blot. Western blot using AR 441 antibody and β Tubulin antibody confirming increased expression of AR in response to different doses of Doxycycline. Lanes represent LNCaP AR cells treated with 1. Ethanol, 2. 10 nM R1881, 3-6. Doxycycline final concentration 3. Doxycycline 10 ng/mL, 4. Doxycycline 20 ng/mL, 5. Doxycycline 40 ng/mL, 6. Doxycycline 100 ng/mL. **b**. Samples prepared as described above were assayed using qPCR for the flag epitope. **c**. The same samples as in **b** were assayed by qPCR for AR-exon 1, a measure of total AR.

Supplementary Figure 16

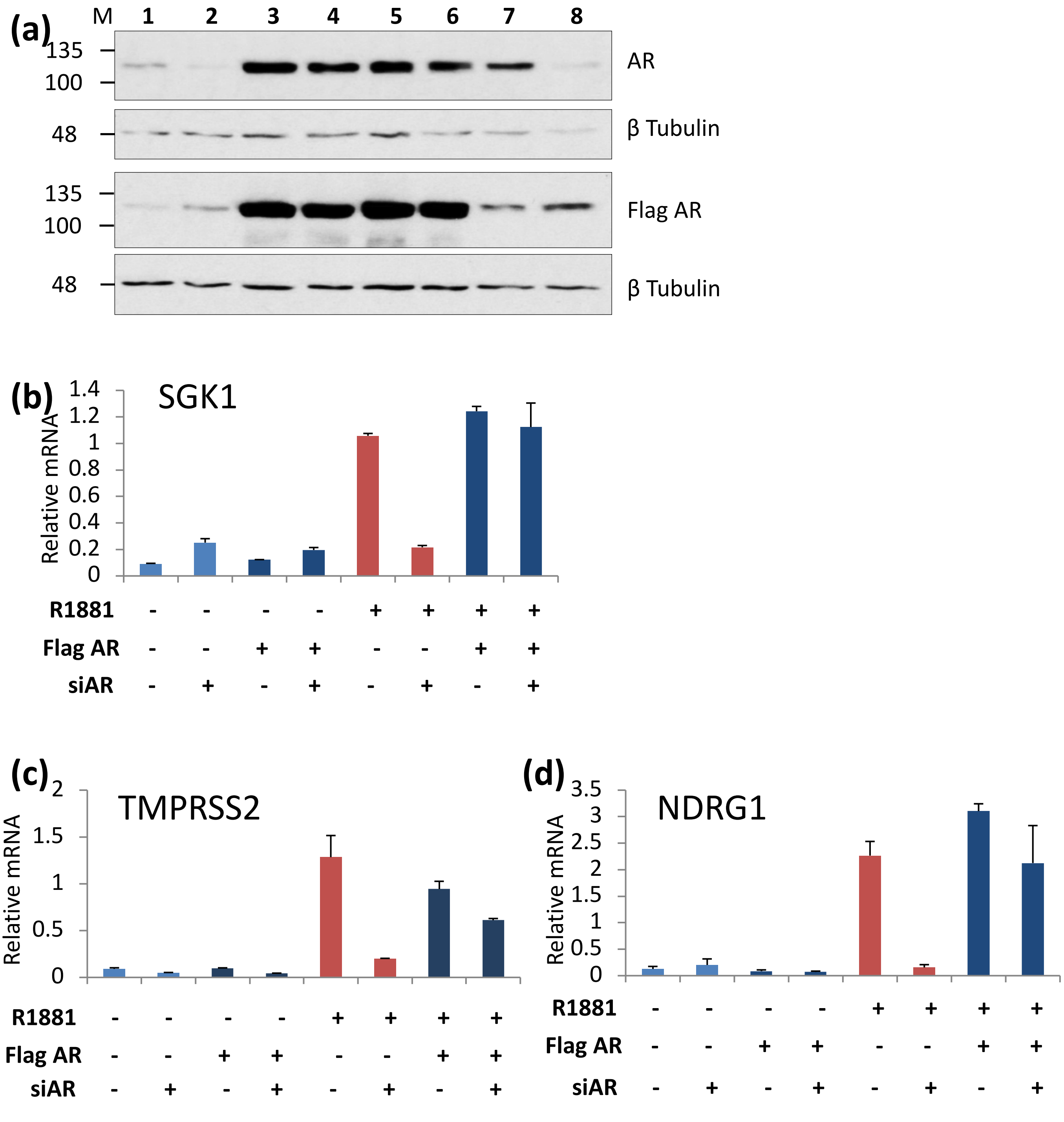

Supplementary Figure 16. Confirmation of flag AR activity. Flag AR cells in CSS were treated with si3'AR, or control siRNA for 48 hours and then treated with vehicle , R1881, or doxycycline (20 ng/ml) for 24 hours prior to harvest for analysis of protein and mRNA. **a**. Western blot using AR 441 antibody and β Tubulin (upper) and flag antibody and tubulin antibody (lower). Lanes represent LNCaP AR cells treated with 1. Vehicle, 2. Vehicle+si3'AR, 3. Dox, 4. Dox+si3'AR, 5. Dox+10 nM R1881, 6. Dox+10 nM R1881+si3'AR, 7. 10 nM R1881, 8. 10 nM R1881+si3'AR. **b-d**, qPCR analysis of **b**. SGK1, **c**, TMPRSS2 , and **d**, NDRG1 expression. Dox treatment was with 20ng/mL, si3'AR – represent cells treated with siControl.

Supplementary Figure 17

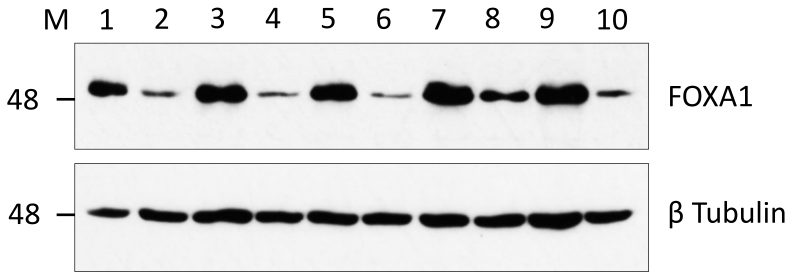

Supplementary Figure 17. Depletion of FOXA1 in LNCaP AR-V7 and LNCaP AR cells. Cells in CSS were treated with control siRNA or siFoXA1 for 24 hours, followed by treatment with vehicle, Doxycycline, or R1881 for another 24 hours.Western blot showing FOXA1 depletion using siRNA transfection in LNCaP AR-V7 (1 – 6) and LNCaP AR (7 – 10) cells 1. Vehicle, 2. Vehicle +siFOXA1, 3. 20 ng/mL final concentration Dox, 4. 20 ng/mL final concentration Doxy+siFOXA1, 5. 10 nM R1881, 6. 10 nM R1881+siFOXA1, 7. LNCaP AR Dox, 8. LNCaP AR Dox +siFOXA1, 9. LNCaP AR Dox+ 10 nM R1881, 10. LNCaP AR Dox +siFOXA1+ 10 nM R1881. Corresponding control cells that did not get siFOXA1 treatment were treated with siControl.

Supplementary Figure 18

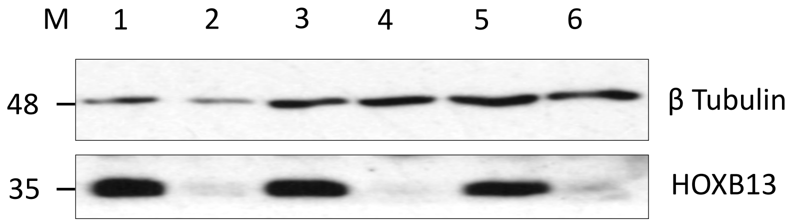

Supplementary Figure 18. Depletion of HOXB13. LNCaP cells in 10% CSS with treated with control or HOXB13 siRNA for 24 hours prior to addition of the indicated treatments for another 24 hours. HOXB13 was detected by western blotting.1. Ethanol, 2. Ethanol +siHOXB13, 3. 20ng/mL final concentration Dox, 4. 20ng/mL final concentration Dox+siHOXB13, 5. 10 nM R1881, 6. 10 nM R1881+siHOXB13. Corresponding control cells that didn't get siFOXA1 treatment were treated with siControl.

Supplementary Figure 19

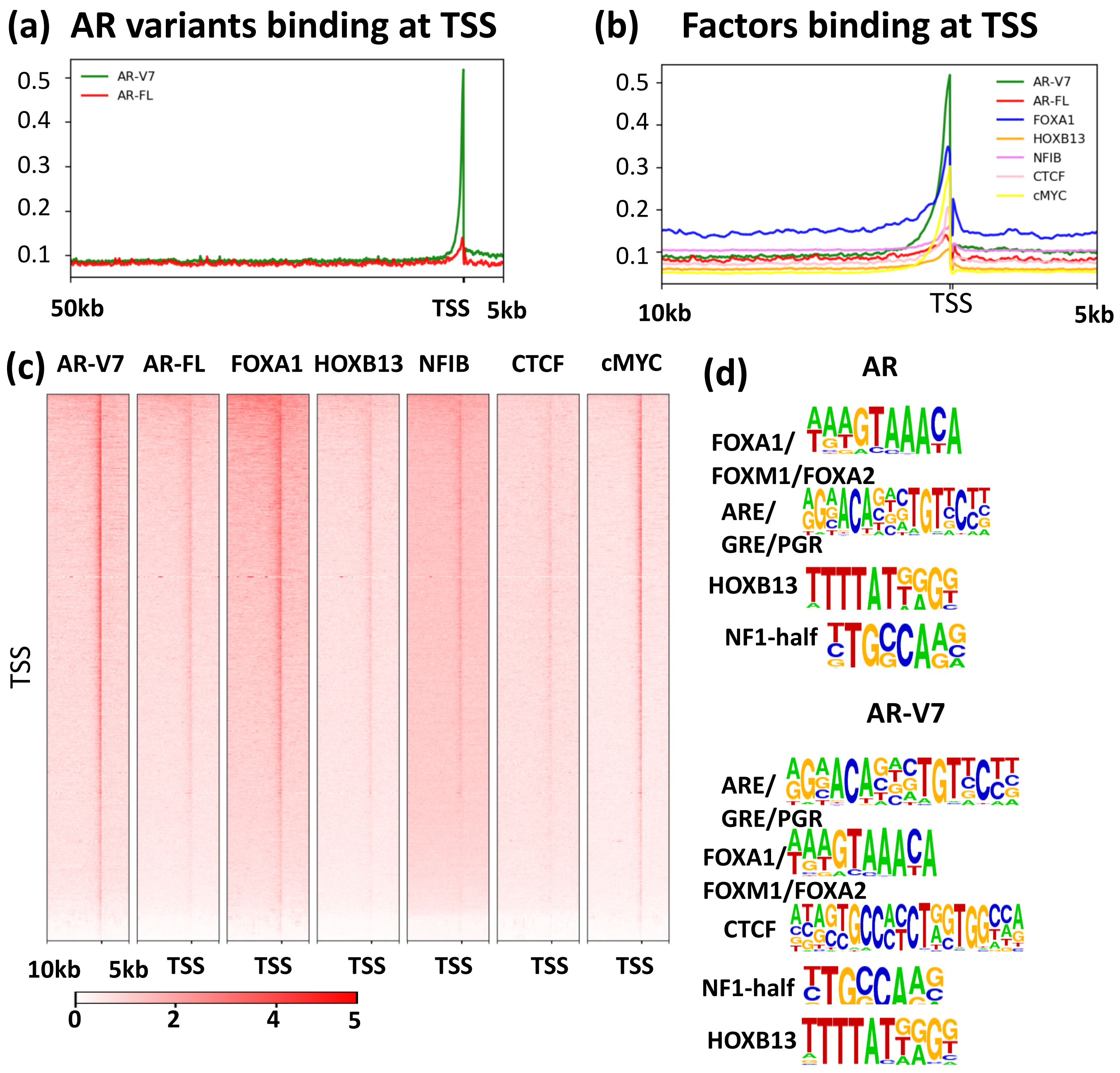

Supplementary Figure 19. Transcription factor binding profile at Transcription Start Sites of DEGs. a. Average ChIP-Exo signal for AR variants and **b.** ChIP signal for other factors at regulatory sites of DEGs, plotted as a profile within 1kb upstream and 1kb downstream. **c**. Heatmap visualizing average ChIP signal for AR variants and other factors at proximal promoter sites (ARBs and AR-V7 binding sites within 100bp to TSS) plotted within 1kb upstream to 1kb downstream of peak center. **d**. Consensus motif sequences identified in a motif analysis using Homer findmotifsGenome.pl, -factor and other default parameters (target peaks vs background $p < 0.05$) of AR and AR-V7 binding sites in Figure 3, panel A.

Supplementary Figure 20

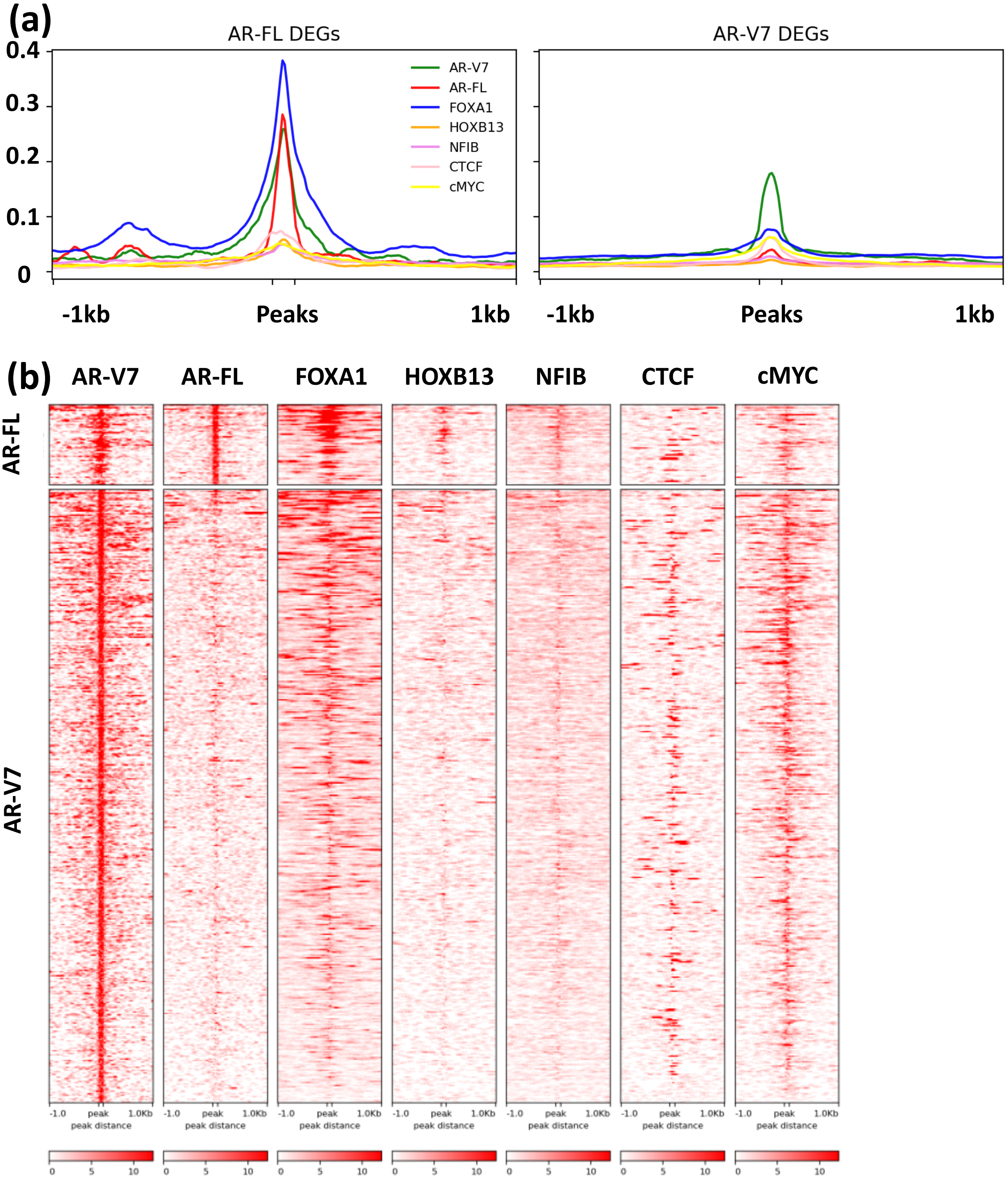

Supplementary Figure 20. Transcription factor binding profile at Transcription Start Sites of DEGs. **a**. Average ChIP signal for AR variants and other factors at regulatory sites of DEGs, plotted as a profile within 1 kb upstream and 1 kb downstream. **b**. Heatmap visualizing average ChIP signal for AR variants and other factors at proximal promoter sites (ARBs and AR-V7 binding sites within 100 bp to TSS) plotted within 1kb upstream to 1kb downstream of peak center.

Supplementary Figure 21

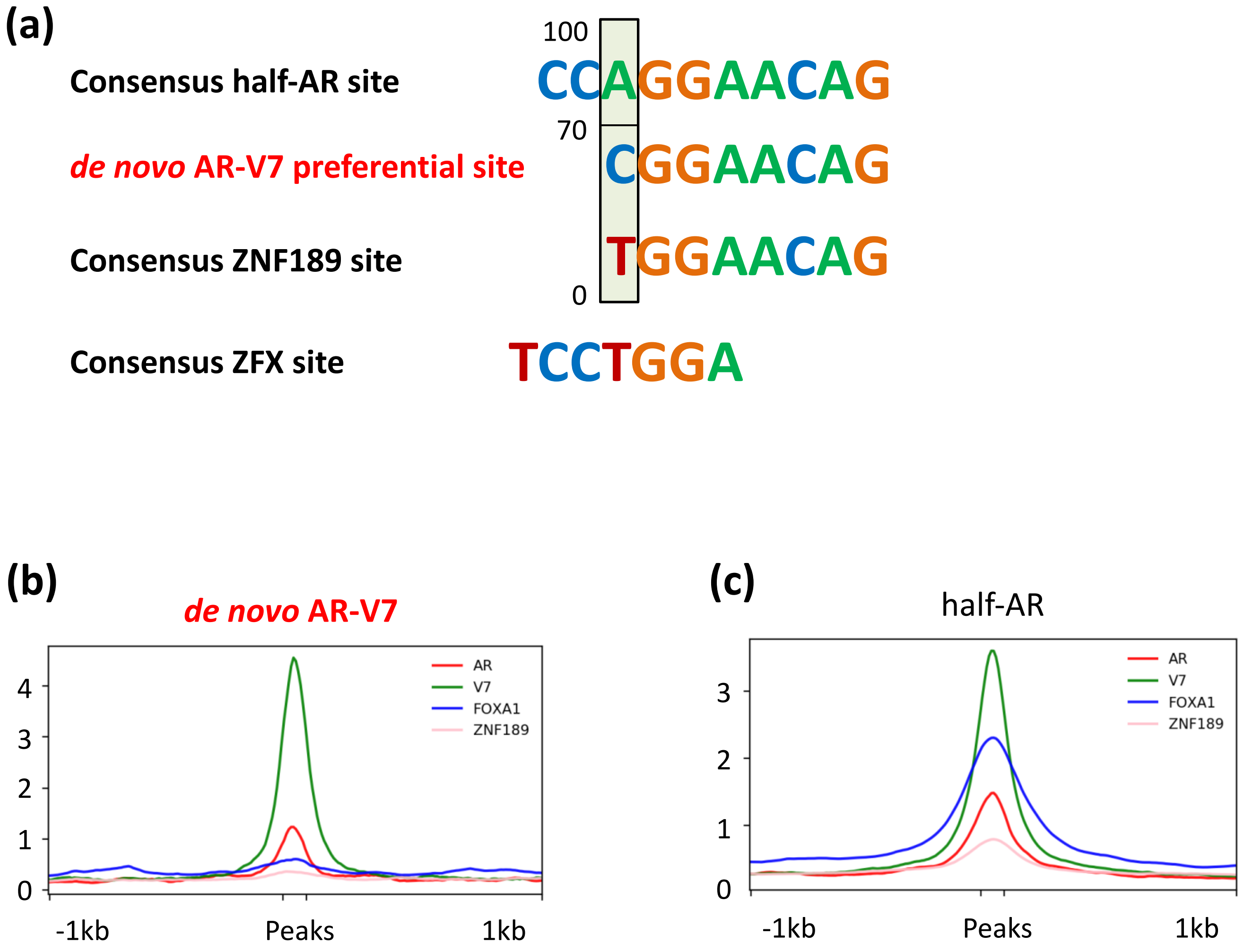

Supplementary Figure 21. AR-V7 *de novo* motifs enriched in a motif analysis within 20bp from peak center. **a**. Consensus half-AR motif, *de novo* AR-V7 motif identified, consensus ZNF189 motif and consensus ZFX motif showing sequence similarity (target peaks vs background $p < 0.05$). **b**. Average ChIP signal for AR variants and other factors at AR-V7 binding sites having *de novo* motif present (within 250bp from peak center). **c**. Average ChIP signal at AR-V7 binding sites with consensus half ARE motif (within 250bp from peak center) is plotted as a profile. b and c are plotted within 1 kb upstream to 1 kb downstream of peak center.

STable1. List of publicly available data sets used in this study.

	**ChIP-seq data**	**Data**
1	**AR ChIP-exo (Data from this Lab)**	**GSE143906**
2	**AR-V7 ChIP-exo (Data from this Lab)**	**GSE143906**
3	AR ChIP-exo	GSE43785
4	FOXA1 ChIP-exo	GSE43678
5	HOXB13 ChIP-seq	GSE96652
6	ZFX ChIP-seq	GSE94013
7	NFIB ChIP-seq	PRJNA276666
8	CTCF ChIP-seq	GSE38685
9	cMYC ChIP-seq	GSE73995

	**RNA-seq data**	
1	**LNCaP AR-V7 (Data from this Lab)**	**GSE143905**
2	**LN95 datasets (Data from this Lab)**	**GSE184676**
3	22RV1 AR-V7 signature (He)	GSE80743
4	22RV1 AR-V7 signature (Cai)	GSE94013
5	22RV1 AR-V7 signature (Kounatidou)	GSE126306
6	LNCaP ABL AR signature	GSE35126
7	C4-2B AR signature	GSE40050
8	FOXA1 KD	GSE27824

STable 2. Sequence information for Real-Time PCR Real-time PCR primer information for qPCR and ChIP-qPCR.

Orientation	Gene	Sequence	Comments
Sense	18S	ACCGCAGCTAGGAATAATGGA	qPCR
Antisense	18S	GCCTCAGTTCCGAAAACCA	qPCR
Sense	PSA (KLK3)	ACCTGCACCCGGAGAGCT	qPCR
Antisense	PSA (KLK3)	TCACGGACAGGGTGAGGAAG	qPCR
Sense	ELL2	GCTCAATTGCCTGGGATTTATAC	qPCR
Antisense	ELL2	CTCTGCCTGGGTCATTCTTT	qPCR
Sense	SGK1	CCTTGACGCTGGCTGTGA	qPCR
Antisense	SGK1	TGACCCCGAGTTTACCGA	qPCR
Sense	TMPRSS2	AGGATCGGTGTGTTCGCCTC	qPCR
Antisense	TMPRSS2	CTCGTTCCAGTCGTCTTGGC	qPCR
Sense	NRDG1	CATGAACCCCGGCAACC	qPCR
Antisense	NRDG1	GCTCAATCTCCAGGTCGCG	qPCR
Sense	OLAH	GAGAGAGGAGACCAACCTAAGA	qPCR
Antisense	OLAH	CAGGGAAAGCAAATCAGCTTAAA	qPCR
Sense	ChIP -ve	AACCTCACTTTCATTGTTACTAGCCATA	ChIP qPCR
Antisense	ChIP -ve	CGCTCAAGGATGTCAGTAGCAT	ChIP qPCR
Sense	TRPM8	CCCCTGGTGTGCATCTACATG	qPCR
Antisense	TRPM8	CCCACCGTGTAGCCAAACAT	qPCR
Sense	PAK1IP1	CCTCTTTGGGTTCGCTGTACAC	qPCR
Antisense	PAK1IP1	TGAAGTCAGCCACAAGAGTCCAT	qPCR
Sense	ELOVL7	TTTGCTGCAGGTGGTTTGGGAA	qPCR
Antisense	ELOVL7	AAATACTTCTGGTAGGCTGGCCC	qPCR
Sense	TRPM8 Enh1	AGCATCAGTGAAATTTGAGACA	ChIP qPCR
Antisense	TRPM8 Enh1	GAGTTTGGGAAATGCTGAAGTG	ChIP qPCR
Sense	EDN2	CTGCAGACGTGTTCCAGAC	qPCR
Antisense	EDN2	AGGCTCTTGACTGTGGAAATG	qPCR
Sense	FKBP5	GGATATACGCCAACATGTTCA	qPCR
Antisense	FKBP5	CCATTGCTTTATTGGCCTCT	qPCR
Sense	NDRG1	CATGAACCCCGGCAACC	qPCR
Antisense	NDRG1	GCTCAATCTCCAGGTCGCG	qPCR
Sense	FOXA1	GTGGCTCCAGGATGTTAGGA	qPCR
Antisense	FOXA1	GAGTAGGCCTCCTGCGTGT	qPCR
Sense	NKX3-1	GAGAGAGCCTTGGCCATATTC	ChIP qPCR
Antisense	NKX3-1	GAGAAAGGACAACCAGGATGAG	ChIP qPCR
Sense	PSA Enh	GACCTACTCTGGAGGAACATATTG	ChIP qPCR
Antisense	PSA Enh	GGCTTGCTTACTGTCCTAGATAA	ChIP qPCR
Sense	BIRC3	CGACAGGTGTCGCTTGAAA	ChIP qPCR
Antisense	BIRC3	ACACACACACACACACACA	ChIP qPCR
Sense	FKBP5	AGGTCAACTCTTGCTGGAAC	ChIP qPCR
Antisense	FKBP5	ACTGGGAGTTGTTTGGTCTG	ChIP qPCR

Sense	PSA Prom	TGCATCCAGGGTGATCTAGTA	ChIP qPCR
Antisense	PSA Prom	GCTGGAGGCTGGACAAC	ChIP qPCR
Sense	ORM1	GCATAATACAACCTGCTGTTCTG	qPCR
Antisense	ORM1	TGTTCCCAACACAAAGAAATGG	qPCR
Sense	HOXB13	TTGCCAGGGAGAACAGAAC	qPCR
Antisense	HOXB13	CTGTACGGAATGCGTTTCTTG	qPCR
Sense	AR-V7	GTCGTCTTCGGAAATGTTATGAA	qPCR
Antisense	AR-V7	TGCCAACCCGGAATTTTTCT	qPCR
Sense	AR-V9	AACAGAAGTACCTGTGCGCC	qPCR
Antisense	AR-V9	TCAGGGTCTGGTCATTTTGA	qPCR
Antisense	AR-V9	GCAAATGTCTCCAAAAAGCAGC	qPCR
Sense	AR Exon1	GGATAGCTACTCCGGACATTACG	qPCR
Antisense	AR Exon1	ATAGTCAATGGGGAAAAGATGGT	qPCR
Sense	AR Flag	ACAAAGACCATGACGGTGATTA	qPCR
Antisense	AR Flag	GAACAGATTCTGGAAAGCTCCT	qPCR

STable 3. The list of differentially expressed genes (DEGs).

LNCaP DEGs

Gene	log2FC R1881/Veh	log2FC Dox/Veh	Direction
NKX6-2	-2.31	7.94	Opposite
NR2F1	-1.93	6.52	Opposite
TNC	-0.62	6.38	Opposite
ZNF727	-1.60	6.18	Opposite
EPHA3	-0.84	5.62	Opposite
KCNJ3	-2.27	5.56	Opposite
ZNF98	-0.64	4.98	Opposite
ALB	-1.18	4.23	Opposite
SOHLH2	-1.36	4.20	Opposite
UBE2QL1	-2.74	3.91	Opposite
BIRC3	-0.62	3.79	Opposite
PKIA	-2.40	3.69	Opposite
TFCP2L1	-1.40	3.50	Opposite
ZNF729	-1.43	3.35	Opposite
CYP7A1	-3.17	3.34	Opposite
CP	-2.14	3.32	Opposite
ATOH8	-1.06	3.31	Opposite
ST6GALNAC2	-0.61	3.26	Opposite
ZNF99	-1.66	3.19	Opposite
KRT80	-1.63	2.96	Opposite
LUM	-1.73	2.93	Opposite
KCNQ5	-1.74	2.92	Opposite
COL27A1	-2.02	2.90	Opposite
INSL5	-1.39	2.86	Opposite
UGT8	-1.05	2.84	Opposite
ADGRD1	-0.77	2.72	Opposite
OVOS2	-1.38	2.70	Opposite
EPAS1	-0.76	2.64	Opposite
EDN2	-2.04	2.51	Opposite
GCG	-2.67	2.49	Opposite
ZNF676	-0.63	2.46	Opposite
KNG1	-1.82	2.38	Opposite
CCDC74A	-0.70	2.31	Opposite
ARHGAP23	-0.80	2.29	Opposite
GSTA1	-0.91	2.26	Opposite
PLPPR1	-3.95	2.25	Opposite
NSG1	-0.68	2.16	Opposite
P3H2	-1.05	2.12	Opposite
ZFHX4	-0.66	2.11	Opposite

VCaP DEGs

Gene	log2FC R1881/Veh	log2FC Dox/Veh	Direction
HSD17B2	3.32	-3.64	Opposite
VWF	0.72	-2.15	Opposite
SV2C	1.43	-1.92	Opposite
PALMD	0.94	-1.60	Opposite
TEAD2	2.73	-1.37	Opposite
LDLRAD4	0.68	-1.35	Opposite
PDE4B	0.72	-1.28	Opposite
ALPK3	1.15	-1.19	Opposite
ZIC3	0.72	-1.15	Opposite
RPH3A	3.44	-1.14	Opposite
SLC22A4	1.21	-1.10	Opposite
ALDH1A1	0.70	-1.07	Opposite
TMC7	0.61	-1.06	Opposite
UGT2B4	0.59	-1.01	Opposite
FPR3	0.93	-1.00	Opposite
GSDMC	3.47	-0.98	Opposite
GNB4	1.27	-0.96	Opposite
ANXA8L1	0.98	-0.94	Opposite
CACNA2D1	1.00	-0.94	Opposite
TRPM8	1.99	-0.93	Opposite
GLB1L3	1.63	-0.91	Opposite
PIK3AP1	1.34	-0.89	Opposite
TFF1	0.69	-0.87	Opposite
KCNK6	1.00	-0.87	Opposite
RGS4	0.87	-0.87	Opposite
ROPN1B	1.26	-0.86	Opposite
BOLA2B	3.48	-0.84	Opposite
SLIT3	0.62	-0.81	Opposite
LGALS3	1.55	-0.78	Opposite
CHST8	0.74	-0.78	Opposite
GDF15	1.09	-0.76	Opposite
CNTD2	0.82	-0.75	Opposite
CDH13	1.21	-0.74	Opposite
TP53INP2	1.05	-0.74	Opposite
ADH1C	1.80	-0.71	Opposite
PDE2A	1.54	-0.70	Opposite
IGFL1	1.57	-0.69	Opposite
PDLIM3	0.66	-0.69	Opposite
GRHL1	0.58	-0.66	Opposite

SDC1	-1.20	2.09	Opposite	MYOF	3.73	-0.64	Opposite
ASPH	-0.75	1.96	Opposite	SASH1	1.17	-0.64	Opposite
GMPR	-1.23	1.95	Opposite	CCDC53	0.74	-0.63	Opposite
XAGE1B	-1.71	1.95	Opposite	PLCB1	0.69	-0.63	Opposite
PDE4D	-1.34	1.94	Opposite	MAK	1.99	-0.63	Opposite
TENM2	-1.30	1.93	Opposite	CYP1A1	0.71	-0.63	Opposite
ZDHHC15	-1.02	1.91	Opposite	VSIG10L	1.89	-0.62	Opposite
GPR176	-0.74	1.87	Opposite	RNF152	1.25	-0.62	Opposite
C5orf46	-1.46	1.82	Opposite	SMPD3	1.43	-0.62	Opposite
ATP2B1	-0.99	1.79	Opposite	IQGAP2	0.72	-0.61	Opposite
BRDT	-1.75	1.77	Opposite	TG	2.30	-0.61	Opposite
KCNU1	-3.87	1.72	Opposite	CRABP2	2.29	-0.61	Opposite
USP2	-0.96	1.69	Opposite	ASB4	1.34	-0.61	Opposite
GRIK2	-1.73	1.61	Opposite	ZDHHC1	0.58	-0.60	Opposite
KIF17	-1.40	1.61	Opposite	SLC6A19	1.05	-0.59	Opposite
ADAMTS17	-0.70	1.61	Opposite	MESP2	1.14	-0.58	Opposite
GSTM4	-0.71	1.59	Opposite	ARID3A	0.70	-0.57	AR-FL
NPTX2	-0.98	1.54	Opposite	SEC14L2	1.44	-0.57	AR-FL
MT2A	-0.66	1.54	Opposite	UGT2B11	1.26	-0.57	AR-FL
RGMA	-0.78	1.49	Opposite	ANKH	0.73	-0.57	AR-FL
FSTL4	-1.45	1.46	Opposite	CYP4B1	1.38	-0.57	AR-FL
MICALCL	-0.84	1.44	Opposite	FAM212B	0.66	-0.55	AR-FL
ZNF257	-1.05	1.40	Opposite	GPR158	0.85	-0.55	AR-FL
MOV10L1	-1.52	1.37	Opposite	CA5B	0.99	-0.54	AR-FL
REEP2	-1.08	1.36	Opposite	SCGB2A1	0.89	-0.53	AR-FL
PITPNM3	-0.68	1.35	Opposite	CREB3L1	2.38	-0.53	AR-FL
SDC2	-1.11	1.34	Opposite	SUSD2	0.73	-0.51	AR-FL
CAMK2D	-0.67	1.33	Opposite	SHROOM1	0.68	-0.50	AR-FL
FCHO1	-1.33	1.31	Opposite	TRIM16	0.90	-0.49	AR-FL
OASL	-1.00	1.31	Opposite	SPON2	0.87	-0.49	AR-FL
ST3GAL5	-1.07	1.28	Opposite	FAM69A	1.09	-0.49	AR-FL
TNS1	-1.56	1.24	Opposite	DSC2	0.70	-0.49	AR-FL
VWDE	-0.86	1.22	Opposite	LGR5	1.38	-0.49	AR-FL
COL9A3	-1.02	1.21	Opposite	LRCH1	1.12	-0.49	AR-FL
PARP8	-1.09	1.18	Opposite	LCK	1.57	-0.48	AR-FL
CHAC2	-0.66	1.17	Opposite	EHBP1	0.70	-0.48	AR-FL
NOTCH2NL	-0.59	1.16	Opposite	SAT1	1.24	-0.45	AR-FL
LAMC3	-0.71	1.14	Opposite	ELOVL7	1.12	-0.45	AR-FL
CES4A	-0.66	1.13	Opposite	BEND4	0.60	-0.45	AR-FL
PRC1	-0.62	1.13	Opposite	VASH2	0.60	-0.43	AR-FL
IER5L	-1.17	1.02	Opposite	CHRM1	1.16	-0.43	AR-FL
FAM49A	-0.93	1.01	Opposite	RAB23	0.62	-0.42	AR-FL
BEX1	-1.35	1.01	Opposite	SOSTDC1	0.87	-0.42	AR-FL
DEGS2	-1.44	1.00	Opposite	CCDC71L	0.96	-0.42	AR-FL
PRIMA1	-1.96	1.00	Opposite	ENDOD1	1.96	-0.41	AR-FL
PDGFRB	-0.78	1.00	Opposite	ACER2	1.30	-0.41	AR-FL

MT1X	-0.74	0.98	Opposite	ANXA2	1.48	-0.41	AR-FL
ZNF492	-1.02	0.96	Opposite	SERPINE2	0.62	-0.41	AR-FL
PGM2L1	-1.54	0.95	Opposite	SLC22A23	0.62	-0.41	AR-FL
C16orf89	-2.30	0.95	Opposite	SNX25	0.80	-0.41	AR-FL
GPR161	-0.67	0.92	Opposite	EMILIN1	0.60	-0.41	AR-FL
CCDC109B	-0.63	0.92	Opposite	ANKRD37	2.57	-0.40	AR-FL
HAS3	-1.25	0.91	Opposite	ATP8A2	0.70	-0.39	AR-FL
SPATA6	-0.95	0.91	Opposite	MAP3K15	0.71	-0.39	AR-FL
CCDC74B	-0.81	0.90	Opposite	RARB	0.88	-0.38	AR-FL
EPB41L2	-0.69	0.89	Opposite	GPNMB	0.66	-0.37	AR-FL
NEU4	-1.01	0.89	Opposite	RASSF3	0.61	-0.37	AR-FL
LPO	-1.82	0.89	Opposite	POPDC3	1.10	-0.37	AR-FL
MSX2	-0.88	0.88	Opposite	SLC16A6	1.20	-0.36	AR-FL
TMEM59L	-1.48	0.88	Opposite	ZNF792	0.60	-0.35	AR-FL
CCNO	-2.41	0.86	Opposite	SH3RF3	0.82	-0.35	AR-FL
CEL	-1.09	0.83	Opposite	HCAR1	0.66	-0.35	AR-FL
SDC4	-1.67	0.83	Opposite	TIGD2	0.67	-0.34	AR-FL
TMPRSS9	-0.73	0.82	Opposite	F3	1.22	-0.34	AR-FL
DDIT4	-0.90	0.81	Opposite	GREM2	1.58	-0.34	AR-FL
CATSPERB	-0.69	0.79	Opposite	ZNF827	0.75	-0.33	AR-FL
TRPC1	-1.56	0.78	Opposite	GNA12	0.61	-0.33	AR-FL
C18orf54	-0.86	0.78	Opposite	DIRAS2	0.70	-0.33	AR-FL
LDOC1	-0.72	0.77	Opposite	SKIL	0.81	-0.32	AR-FL
CACNA1G	-2.99	0.77	Opposite	PHLDB3	1.11	-0.32	AR-FL
TIGD3	-0.87	0.75	Opposite	INSM1	0.99	-0.32	AR-FL
ACSF2	-1.20	0.75	Opposite	SHISA8	1.52	-0.32	AR-FL
SPAG8	-0.78	0.75	Opposite	ITPKB	1.06	-0.31	AR-FL
FBXL2	-0.73	0.72	Opposite	NOD2	1.78	-0.31	AR-FL
CCDC155	-0.97	0.71	Opposite	SMN1	1.61	-0.31	AR-FL
FEZ1	-0.69	0.70	Opposite	AFAP1	0.79	-0.31	AR-FL
TRIM29	-1.88	0.70	Opposite	RAPGEF5	0.67	-0.30	AR-FL
KLRG2	-1.33	0.70	Opposite	ZNF703	0.71	-0.30	AR-FL
GPER1	-0.69	0.69	Opposite	FAM3D	1.98	-0.29	AR-FL
TLR3	-0.63	0.69	Opposite	DDR2	0.86	-0.29	AR-FL
ZNF594	-0.61	0.69	Opposite	GMDS	0.84	-0.28	AR-FL
CFAP69	-0.63	0.69	Opposite	DEGS1	0.60	-0.28	AR-FL
SLC12A4	-0.71	0.69	Opposite	SLC2A10	1.08	-0.27	AR-FL
MECOM	-3.55	0.68	Opposite	SYTL1	0.75	-0.27	AR-FL
ASZ1	-4.49	0.68	Opposite	ADRB1	0.66	-0.27	AR-FL
RARRES3	-1.14	0.67	Opposite	OTUD7B	1.17	-0.27	AR-FL
LEKR1	-0.75	0.67	Opposite	ELOVL2	1.16	-0.26	AR-FL
VEPH1	-1.09	0.67	Opposite	SLC10A4	0.92	-0.26	AR-FL
ADAM23	-1.89	0.64	Opposite	ETV6	0.74	-0.25	AR-FL
IFIT1	-0.83	0.64	Opposite	LAMC2	1.15	-0.25	AR-FL
IFI27L2	-2.62	0.63	Opposite	MME	0.83	-0.25	AR-FL
TRIM54	-1.36	0.62	Opposite	TBC1D16	0.65	-0.25	AR-FL

FOSB	-0.60	0.60	Opposite
SMC6	-1.06	0.60	Opposite
PPFIA4	-0.80	0.59	Opposite
NHLRC4	-0.82	0.59	Opposite
SPOCK2	-0.74	0.58	Opposite
ZBBX	-0.79	0.58	AR-FL
SMIM3	-1.95	0.57	AR-FL
MAGED4B	-0.67	0.57	AR-FL
MYO15A	-0.94	0.57	AR-FL
CNBD1	-2.28	0.57	AR-FL
MET	-2.75	0.56	AR-FL
CA13	-0.90	0.56	AR-FL
ADRA1A	-1.91	0.55	AR-FL
NOS3	-0.60	0.55	AR-FL
AHNAK2	-0.93	0.52	AR-FL
ZNF711	-1.28	0.52	AR-FL
TMEM45A	-0.77	0.52	AR-FL
ASS1	-0.58	0.52	AR-FL
SERPINB5	-0.68	0.52	AR-FL
CES3	-0.96	0.51	AR-FL
PCP2	-0.85	0.51	AR-FL
GNG4	-1.24	0.50	AR-FL
QSOX1	-0.79	0.50	AR-FL
RCN3	-0.66	0.50	AR-FL
HSPA4L	-1.51	0.49	AR-FL
IRF1	-0.70	0.48	AR-FL
NFASC	-0.78	0.47	AR-FL
DYRK3	-0.73	0.47	AR-FL
LEF1	-0.79	0.47	AR-FL
UNC5CL	-0.75	0.47	AR-FL
EIF3C	-0.66	0.46	AR-FL
ANKRD2	-2.01	0.45	AR-FL
PIFO	-0.82	0.45	AR-FL
KCNH3	-1.22	0.45	AR-FL
SI	-4.83	0.45	AR-FL
IRX3	-1.09	0.45	AR-FL
SPTB	-0.62	0.44	AR-FL
LRP1	-0.76	0.44	AR-FL
RP5-1187M17.10	-0.61	0.44	AR-FL
THRA	-0.74	0.43	AR-FL
PLEKHA7	-0.88	0.43	AR-FL
ANKRD31	-1.13	0.42	AR-FL
NOVA1	-1.87	0.41	AR-FL
CAMK1D	-2.39	0.41	AR-FL
HNF4G	-0.82	0.40	AR-FL
RAB40A	-0.65	0.39	AR-FL
SERPINA11	0.64	-0.25	AR-FL
NBL1	1.27	-0.25	AR-FL
TM4SF1	0.70	-0.24	AR-FL
MTCL1	1.31	-0.24	AR-FL
BAIAP2L1	0.60	-0.24	AR-FL
B3GNT5	0.66	-0.23	AR-FL
CLDN8	1.81	-0.23	AR-FL
SEL1L3	0.68	-0.23	AR-FL
FMOD	1.31	-0.23	AR-FL
CCDC160	1.56	-0.22	AR-FL
LPAR2	0.83	-0.22	AR-FL
FAM105A	1.25	-0.22	AR-FL
NKX3-1	0.88	-0.21	AR-FL
C3orf14	1.35	-0.21	AR-FL
TMEM210	1.23	-0.21	AR-FL
EPS15	0.65	-0.21	AR-FL
C20orf96	1.22	-0.21	AR-FL
GRK5	0.90	-0.21	AR-FL
SGK223	0.96	-0.21	AR-FL
GUCY1A3	0.72	-0.21	AR-FL
UAP1	1.25	-0.20	AR-FL
ATP7B	0.84	-0.20	AR-FL
ALOX15	0.82	-0.19	AR-FL
PDE9A	0.64	-0.19	AR-FL
ARF6	0.71	-0.19	AR-FL
TJP2	1.03	-0.19	AR-FL
ILDR1	0.62	-0.18	AR-FL
TMEM135	0.68	-0.18	AR-FL
UCHL1	0.80	-0.18	AR-FL
SPECC1L	0.62	-0.18	AR-FL
POLB	0.66	-0.17	AR-FL
SEC14L1	0.59	-0.17	AR-FL
BCAR3	0.61	-0.17	AR-FL
KLHL29	1.24	-0.17	AR-FL
MMD	0.80	-0.16	AR-FL
CEBPA	0.75	-0.16	AR-FL
PCCA	0.66	-0.16	AR-FL
DNASE2B	0.87	-0.16	AR-FL
SEMG2	2.47	-0.16	AR-FL
CARD6	0.69	-0.15	AR-FL
PEX10	1.73	-0.15	AR-FL
ETS1	1.13	-0.14	AR-FL
TTC39A	0.60	-0.13	AR-FL
SPATC1L	0.85	-0.13	AR-FL
DLGAP1	0.82	-0.12	AR-FL
TP53I11	1.19	-0.12	AR-FL

IFIT3	-0.78	0.39	AR-FL	DMXL1	0.60	-0.12	AR-FL
TCF4	-0.71	0.39	AR-FL	HES2	2.49	-0.12	AR-FL
CDHR3	-0.68	0.37	AR-FL	LIMD2	1.22	-0.11	AR-FL
APBB1	-0.81	0.37	AR-FL	FMNL2	0.83	-0.11	AR-FL
FAM234B	-0.81	0.36	AR-FL	TMEM55A	0.69	-0.11	AR-FL
SLC1A1	-0.65	0.35	AR-FL	NKAIN1	0.58	-0.11	AR-FL
NAP1L5	-1.63	0.35	AR-FL	SOAT1	0.58	-0.11	AR-FL
MCTP1	-1.92	0.35	AR-FL	BHLHE40	1.31	-0.10	AR-FL
DIAPH2	-0.82	0.35	AR-FL	ABLIM2	0.80	-0.10	AR-FL
MICA	-1.34	0.35	AR-FL	MALL	1.27	-0.10	AR-FL
RIBC1	-0.83	0.35	AR-FL	BAZ1A	0.64	-0.10	AR-FL
NUAK2	-1.67	0.35	AR-FL	ASPHD2	1.50	-0.10	AR-FL
CCDC146	-0.94	0.34	AR-FL	CSGALNACT1	2.52	-0.09	AR-FL
GRIN1	-0.70	0.34	AR-FL	ANXA5	0.78	-0.09	AR-FL
RAD9B	-0.64	0.33	AR-FL	CORO2A	1.40	-0.09	AR-FL
PBX4	-0.75	0.32	AR-FL	CRACR2A	1.93	-0.09	AR-FL
F2R	-0.84	0.32	AR-FL	OSMR	0.76	-0.09	AR-FL
EPHX4	-1.58	0.31	AR-FL	MARVELD3	1.11	-0.08	AR-FL
SPATA18	-1.01	0.31	AR-FL	GJB2	0.93	-0.08	AR-FL
HLF	-0.80	0.30	AR-FL	INTS10	1.13	-0.08	AR-FL
ENPP3	-0.90	0.30	AR-FL	PTPRN2	0.63	-0.08	AR-FL
ABCC5	-0.79	0.29	AR-FL	C9orf152	0.66	-0.08	AR-FL
GYLTL1B	-0.69	0.29	AR-FL	ERBB2IP	0.89	-0.08	AR-FL
KCNG1	-1.06	0.28	AR-FL	TCTN2	0.86	-0.08	AR-FL
ZNF385C	-0.89	0.28	AR-FL	GLRX2	1.60	-0.08	AR-FL
ERBB2	-0.59	0.28	AR-FL	MEX3D	0.67	-0.08	AR-FL
ATP1B1	-1.07	0.28	AR-FL	PDZRN3	0.78	-0.07	AR-FL
TRIB1	-0.82	0.28	AR-FL	RIPK4	0.64	-0.07	AR-FL
FBLN2	-1.16	0.28	AR-FL	SLC8B1	0.65	-0.07	AR-FL
PLXDC1	-0.86	0.28	AR-FL	KAZALD1	1.25	-0.07	AR-FL
EPHB2	-0.89	0.27	AR-FL	C1orf116	0.61	-0.06	AR-FL
STON2	-1.17	0.27	AR-FL	TSPAN33	0.65	-0.06	AR-FL
LCAT	-0.60	0.27	AR-FL	AP1S2	0.58	-0.06	AR-FL
NLGN1	-1.72	0.27	AR-FL	PDGFA	0.91	-0.06	AR-FL
PLCXD1	-0.82	0.26	AR-FL	FAM214A	0.80	-0.04	AR-FL
MMP24	-0.78	0.26	AR-FL	PXDN	0.66	-0.04	AR-FL
FAM167B	-0.76	0.25	AR-FL	ZBTB24	0.69	-0.04	AR-FL
CIART	-0.82	0.24	AR-FL	LMAN1L	1.82	-0.04	AR-FL
NATD1	-1.66	0.24	AR-FL	STXBP1	0.64	-0.04	AR-FL
LYST	-0.64	0.24	AR-FL	CEP55	0.68	-0.04	AR-FL
AP006285.2	-0.70	0.24	AR-FL	MMP15	1.05	-0.04	AR-FL
PKDREJ	-0.63	0.24	AR-FL	GPCPD1	1.52	-0.03	AR-FL
ZIC2	-0.73	0.24	AR-FL	MALT1	1.06	-0.03	AR-FL
ANKS1B	-0.65	0.24	AR-FL	TRAM2	0.92	-0.03	AR-FL
HLA-DQB1	-0.79	0.24	AR-FL	SEC62	0.58	-0.03	AR-FL
C1orf145	-0.89	0.23	AR-FL	RAB19	0.59	-0.03	AR-FL

LRRC73	-1.13	0.22	AR-FL
EBF4	-0.71	0.22	AR-FL
TBX6	-0.87	0.22	AR-FL
AR	-0.63	0.21	AR-FL
KLHL13	-0.62	0.21	AR-FL
ALDH3B1	-0.67	0.21	AR-FL
MYLK	-0.94	0.20	AR-FL
CBLN2	-2.03	0.20	AR-FL
MAST1	-0.67	0.20	AR-FL
LFNG	-1.08	0.20	AR-FL
ANKRD16	-0.85	0.19	AR-FL
UGT2B17	-2.31	0.19	AR-FL
SCARF1	-0.95	0.19	AR-FL
KIAA1614	-0.78	0.19	AR-FL
LAMB1	-1.10	0.19	AR-FL
TPPP	-0.60	0.19	AR-FL
IQCC	-0.62	0.19	AR-FL
RSPH1	-0.64	0.18	AR-FL
SEMA6C	-0.64	0.18	AR-FL
COL16A1	-2.45	0.17	AR-FL
MFSD4	-1.01	0.17	AR-FL
DENND5B	-0.88	0.17	AR-FL
ST7	-1.40	0.17	AR-FL
NAB1	-0.79	0.17	AR-FL
PRKG2	-0.64	0.16	AR-FL
S1PR3	-0.77	0.15	AR-FL
CCDC69	-1.59	0.15	AR-FL
LASP1	-1.25	0.15	AR-FL
TCF7L2	-0.88	0.14	AR-FL
RMI1	-0.67	0.14	AR-FL
CABLES2	-0.61	0.14	AR-FL
HEG1	-0.58	0.14	AR-FL
ABCB9	-0.59	0.13	AR-FL
POLR3GL	-0.74	0.13	AR-FL
ASMTL	-0.66	0.13	AR-FL
STX16-NPEPL1	-0.64	0.13	AR-FL
TMEM38A	-0.58	0.13	AR-FL
PCSK6	-0.61	0.12	AR-FL
DHDH	-1.12	0.12	AR-FL
PNPLA3	-1.11	0.11	AR-FL
MAGEA2	-0.79	0.11	AR-FL
PDE6D	-0.60	0.11	AR-FL
ACOXL	-0.59	0.11	AR-FL
NUP93	-1.35	0.11	AR-FL
CLSTN3	-0.74	0.10	AR-FL
MACROD2	-0.61	0.10	AR-FL

ITSN2	0.82	-0.03	AR-FL
CRISP3	1.09	-0.02	AR-FL
TRAPPC5	0.72	-0.02	AR-FL
NDRG3	0.61	-0.02	AR-FL
OSTF1	0.83	-0.01	AR-FL
TJP1	0.68	-0.01	AR-FL
CEACAM1	1.43	-0.01	AR-FL
CD320	0.65	-0.01	AR-FL
ITGB5	0.67	-0.01	AR-FL
FBN2	1.29	-0.01	AR-FL
CD24	1.05	0.00	AR-FL
SH3RF2	1.41	0.00	AR-FL
CADM2	0.99	0.00	AR-FL
INCA1	1.26	0.00	AR-FL
MAP3K14	-0.59	0.00	AR-FL
CDK5R2	-0.82	0.00	AR-FL
C11orf70	-2.33	0.00	AR-FL
ADAM8	-1.14	0.00	AR-FL
TMEM67	-0.58	0.00	AR-FL
SNAPC1	-0.60	0.00	AR-FL
ERAP2	-1.08	0.01	AR-FL
ATP6V1C2	-0.82	0.01	AR-FL
PCSK6	-0.81	0.01	AR-FL
TOP2A	-0.60	0.01	AR-FL
CTGF	-1.94	0.01	AR-FL
EYS	-0.87	0.01	AR-FL
FOXO3	-1.05	0.02	AR-FL
PRR22	-1.07	0.02	AR-FL
KCNQ2	-0.59	0.02	AR-FL
TAS2R31	-1.01	0.03	AR-FL
WDR97	-0.69	0.03	AR-FL
RGS11	-0.91	0.03	AR-FL
IGSF9B	-0.68	0.03	AR-FL
C16orf45	-1.71	0.03	AR-FL
ENO2	-1.06	0.03	AR-FL
SMARCA2	-1.07	0.03	AR-FL
MTUS2	-0.92	0.04	AR-FL
PEG10	-2.04	0.04	AR-FL
CRIP1	-1.50	0.04	AR-FL
SLC4A8	-0.91	0.04	AR-FL
IRS1	-1.43	0.04	AR-FL
VWA8	-0.75	0.04	AR-FL
ARHGDIG	-0.80	0.04	AR-FL
SORBS1	-0.75	0.05	AR-FL
LDB3	-0.71	0.05	AR-FL
SYT12	-1.90	0.05	AR-FL

DDIT4L	-1.48	0.10	AR-FL
FANK1	-0.68	0.10	AR-FL
WEE1	-0.67	0.10	AR-FL
CYP2C18	-0.58	0.10	AR-FL
PAG1	-0.69	0.09	AR-FL
CCDC88B	-1.03	0.08	AR-FL
SFR1	-0.71	0.08	AR-FL
EPHX1	-0.65	0.08	AR-FL
VTA1	-0.59	0.08	AR-FL
QRICH2	-0.64	0.08	AR-FL
CD82	-0.76	0.07	AR-FL
C2CD4D	-0.64	0.07	AR-FL
MGAT5	-0.60	0.07	AR-FL
SUSD5	-0.90	0.07	AR-FL
PAQR8	-0.97	0.07	AR-FL
ARHGEF17	-1.04	0.06	AR-FL
KIAA1683	-0.91	0.06	AR-FL
CCDC148	-1.15	0.06	AR-FL
CHML	-0.62	0.06	AR-FL
TMEM91	-0.61	0.05	AR-FL
F10	-0.59	0.05	AR-FL
RP11-1212A22.4	-0.71	0.05	AR-FL
C17orf53	-0.94	0.05	AR-FL
MLKL	-0.91	0.05	AR-FL
IL17RD	-1.13	0.05	AR-FL
HMGB2	-0.77	0.05	AR-FL
SH3BP5	-0.68	0.05	AR-FL
EPS8	-1.06	0.04	AR-FL
SMARCD3	-1.24	0.04	AR-FL
HPCAL4	-1.21	0.04	AR-FL
ZNF695	-0.80	0.04	AR-FL
GPC1	-0.67	0.04	AR-FL
WDR31	-0.69	0.03	AR-FL
PLAUR	-0.90	0.03	AR-FL
SLC9A1	-0.59	0.03	AR-FL
BLM	-0.95	0.03	AR-FL
ASB13	-0.81	0.02	AR-FL
CLEC2D	-0.64	0.02	AR-FL
ZNF385A	-0.93	0.02	AR-FL
FAM83D	-0.64	0.02	AR-FL
ZFHX2	-0.69	0.02	AR-FL
KRTAP5-1	-0.78	0.02	AR-FL
PLK3	-0.62	0.02	AR-FL
ZNF519	-0.73	0.01	AR-FL
SEMA6A	-1.63	0.01	AR-FL
EMID1	-1.25	0.01	AR-FL
PYGO1	-1.42	0.05	AR-FL
TTC23L	-0.95	0.06	AR-FL
SYCE2	-1.68	0.06	AR-FL
SLC1A2	-0.95	0.06	AR-FL
ACRV1	-0.70	0.06	AR-FL
CARNS1	-0.99	0.06	AR-FL
CSAD	-0.58	0.06	AR-FL
PRUNE2	-1.35	0.06	AR-FL
RAPGEFL1	-1.09	0.06	AR-FL
ZNF76	-0.65	0.06	AR-FL
MAP3K12	-0.68	0.07	AR-FL
A2ML1	-1.28	0.07	AR-FL
DEPDC4	-0.77	0.07	AR-FL
AMH	-0.59	0.07	AR-FL
EMID1	-0.71	0.07	AR-FL
ZNF671	-0.58	0.07	AR-FL
CLDN1	-0.99	0.07	AR-FL
SRD5A1	-0.93	0.07	AR-FL
IFT172	-0.61	0.08	AR-FL
TOR4A	-1.17	0.08	AR-FL
UBE2L6	-0.81	0.08	AR-FL
LCA5L	-0.64	0.08	AR-FL
PHKG1	-0.77	0.08	AR-FL
NAV1	-0.88	0.08	AR-FL
IFITM10	-0.73	0.08	AR-FL
ACTA2	-0.68	0.08	AR-FL
CPNE7	-0.98	0.08	AR-FL
NFIL3	-1.29	0.09	AR-FL
EPB41L2	-0.59	0.09	AR-FL
ALDH3B1	-0.94	0.09	AR-FL
SLC9A5	-0.86	0.09	AR-FL
PLA2G7	-1.39	0.09	AR-FL
AKNA	-0.67	0.09	AR-FL
SNPH	-0.79	0.09	AR-FL
WBP2NL	-0.92	0.10	AR-FL
RAB6B	-0.95	0.10	AR-FL
PPFIBP2	-0.71	0.11	AR-FL
TMEM65	-0.86	0.11	AR-FL
ARSK	-0.84	0.11	AR-FL
CLIP3	-1.51	0.11	AR-FL
ZNF19	-1.31	0.11	AR-FL
ACBD7	-0.88	0.11	AR-FL
PDLIM1	-1.46	0.12	AR-FL
TFAP2E	-0.60	0.12	AR-FL
NME5	-0.76	0.12	AR-FL
ZNF132	-0.63	0.12	AR-FL

TCTEX1D4	-1.12	0.01	AR-FL
ARHGEF2	-0.74	0.01	AR-FL
CEP152	-0.76	0.00	AR-FL
SOWAHD	-1.12	0.00	AR-FL
TMEM132A	-0.78	0.00	AR-FL
MAN1A1	-3.29	0.00	AR-FL
SYT4	0.02	-7.26	AR-V7
KCNH8	0.08	-6.48	AR-V7
C5orf42	0.18	-6.22	AR-V7
OBSL1	0.01	-5.66	AR-V7
DSC3	0.02	-5.38	AR-V7
PDE10A	0.18	-3.81	AR-V7
EBF2	0.13	-3.70	AR-V7
MSI1	0.27	-3.41	AR-V7
PSMD5	0.37	-3.35	AR-V7
FAM213A	0.16	-3.30	AR-V7
SLC4A4	0.53	-3.05	AR-V7
GUCY1B3	0.06	-2.98	AR-V7
PHF11	0.21	-2.96	AR-V7
KCND3	0.14	-2.92	AR-V7
RLN1	0.11	-2.79	AR-V7
NDUFA4L2	0.06	-2.59	AR-V7
FOXD1	0.46	-2.58	AR-V7
SP140L	0.33	-2.49	AR-V7
DNALI1	0.27	-2.46	AR-V7
MDGA1	0.28	-2.37	AR-V7
PXDN	0.58	-2.35	AR-V7
PHTF1	0.41	-2.34	AR-V7
TMEM45B	0.51	-2.29	AR-V7
STOX2	0.02	-2.12	AR-V7
ECHDC1	0.02	-1.87	AR-V7
UTRN	0.53	-1.87	AR-V7
TMEM178B	0.39	-1.83	AR-V7
ADRA2A	0.09	-1.77	AR-V7
PIK3AP1	0.02	-1.76	AR-V7
PCBP3	0.04	-1.73	AR-V7
RNF152	0.40	-1.72	AR-V7
FAM110C	0.02	-1.71	AR-V7
RNF150	0.52	-1.71	AR-V7
PRPH	0.10	-1.68	AR-V7
DNAH8	0.05	-1.65	AR-V7
PLXDC2	0.55	-1.56	AR-V7
FAM129A	0.15	-1.53	AR-V7
SP100	0.17	-1.52	AR-V7
IFITM2	0.32	-1.52	AR-V7
MCTP2	0.58	-1.50	AR-V7
COL27A1	-2.08	0.12	AR-FL
PLPP2	-1.09	0.12	AR-FL
LETM2	-0.64	0.13	AR-FL
ENO3	-0.92	0.13	AR-FL
CYP3A5	-1.13	0.13	AR-FL
TMEM179	-1.36	0.13	AR-FL
CEP126	-2.73	0.13	AR-FL
TGFBR2	-0.86	0.14	AR-FL
ETS2	-1.41	0.14	AR-FL
DNAH1	-0.78	0.14	AR-FL
FBXL2	-0.77	0.14	AR-FL
ERC2	-1.14	0.14	AR-FL
SAXO2	-0.92	0.14	AR-FL
TANC2	-0.73	0.15	AR-FL
HYPK	-0.67	0.15	AR-FL
RNF207	-0.75	0.16	AR-FL
TMEM91	-1.02	0.16	AR-FL
FAM19A5	-0.89	0.16	AR-FL
IFT80	-0.68	0.16	AR-FL
IGFBP2	-0.68	0.16	AR-FL
CCDC93	-0.61	0.17	AR-FL
RERG	-2.06	0.18	AR-FL
SEMA7A	-1.19	0.18	AR-FL
PARP14	-1.11	0.18	AR-FL
PLEKHH2	-0.88	0.18	AR-FL
ZNF337	-0.66	0.19	AR-FL
ASPHD1	-0.76	0.20	AR-FL
STOX2	-0.76	0.20	AR-FL
FAM198A	-1.53	0.20	AR-FL
ADM5	-1.06	0.20	AR-FL
TREX2	-0.60	0.20	AR-FL
SDC2	-2.29	0.21	AR-FL
EIF4A1	-0.68	0.21	AR-FL
FERMT2	-1.14	0.21	AR-FL
PNMA3	-1.60	0.21	AR-FL
TMC3	-0.84	0.22	AR-FL
RAP1GDS1	-0.62	0.22	AR-FL
USP46	-0.63	0.23	AR-FL
SGSM2	-0.75	0.23	AR-FL
CDKN2A	-1.41	0.23	AR-FL
LRRC4	-2.09	0.24	AR-FL
FMNL3	-0.81	0.24	AR-FL
NAT8L	-0.93	0.24	AR-FL
RP11-849H4.2	-0.69	0.24	AR-FL
NGFRAP1	-0.76	0.24	AR-FL
MROH8	-1.03	0.25	AR-FL

SCARF2	0.08	-1.46	AR-V7
CLEC7A	0.42	-1.46	AR-V7
UST	0.52	-1.45	AR-V7
CACNA1D	0.11	-1.39	AR-V7
NAALADL2	0.07	-1.37	AR-V7
OSBPL10	0.25	-1.35	AR-V7
GYG2	0.02	-1.34	AR-V7
SLC16A3	0.54	-1.34	AR-V7
SLC1A5	0.31	-1.33	AR-V7
SMAGP	0.40	-1.33	AR-V7
RINL	0.02	-1.31	AR-V7
TP53I11	0.46	-1.30	AR-V7
HEY1	0.22	-1.29	AR-V7
GPC6	0.56	-1.26	AR-V7
ABHD17C	0.30	-1.24	AR-V7
OSBPL6	0.37	-1.24	AR-V7
SPIRE1	0.13	-1.23	AR-V7
MYO6	0.38	-1.22	AR-V7
CAB39L	0.19	-1.22	AR-V7
TANC1	0.05	-1.17	AR-V7
FAM135A	0.23	-1.16	AR-V7
CRIP2	0.53	-1.16	AR-V7
RNF24	0.18	-1.13	AR-V7
SPRY1	0.40	-1.11	AR-V7
CYP2E1	0.37	-1.08	AR-V7
DTNA	0.12	-1.08	AR-V7
CPT1A	0.11	-1.06	AR-V7
COLGALT2	0.38	-1.05	AR-V7
MED27	0.07	-1.05	AR-V7
SNAP91	0.41	-1.04	AR-V7
NEDD4	0.18	-1.03	AR-V7
RCBTB2	0.08	-1.02	AR-V7
SAPCD2	0.03	-1.02	AR-V7
NRIP1	0.22	-1.00	AR-V7
TTC7B	0.02	-0.98	AR-V7
DGKB	0.46	-0.97	AR-V7
PACSIN1	0.23	-0.94	AR-V7
HCN2	0.58	-0.94	AR-V7
CYP2J2	0.08	-0.94	AR-V7
ERGIC1	0.50	-0.94	AR-V7
TMC1	0.22	-0.90	AR-V7
SMAD9	0.11	-0.89	AR-V7
SPON2	0.32	-0.89	AR-V7
EPB41L4B	0.23	-0.87	AR-V7
RALGAPA2	0.32	-0.87	AR-V7
EFNB2	0.27	-0.85	AR-V7
RIPK2	-1.01	0.25	AR-FL
RCCD1	-1.10	0.25	AR-FL
NTSR1	-1.74	0.25	AR-FL
MAMDC4	-0.74	0.25	AR-FL
TUBE1	-0.67	0.26	AR-FL
ATOH1	-2.28	0.26	AR-FL
AGTR1	-2.14	0.26	AR-FL
AF011889.5	-1.10	0.27	AR-FL
FLT4	-1.00	0.27	AR-FL
FAM169A	-0.89	0.28	AR-FL
SACS	-0.81	0.28	AR-FL
PTGER4	-1.66	0.28	AR-FL
ITGB8	-1.48	0.28	AR-FL
SLC5A3	-0.72	0.29	AR-FL
C2orf15	-1.04	0.30	AR-FL
SYNGR1	-1.09	0.31	AR-FL
IQGAP3	-0.71	0.31	AR-FL
FAM102B	-1.47	0.31	AR-FL
WIF1	-0.70	0.31	AR-FL
NKD1	-0.73	0.31	AR-FL
PDE4A	-1.64	0.32	AR-FL
SUSD4	-1.11	0.32	AR-FL
MXRA7	-0.81	0.32	AR-FL
THRA	-1.22	0.32	AR-FL
ADD2	-0.84	0.33	AR-FL
DGAT2	-0.69	0.33	AR-FL
HS3ST1	-0.77	0.33	AR-FL
TLR5	-0.71	0.34	AR-FL
TMEM59L	-1.28	0.35	AR-FL
TPH1	-0.64	0.35	AR-FL
GJC1	-0.67	0.35	AR-FL
KCND1	-1.36	0.35	AR-FL
ITGA7	-1.02	0.35	AR-FL
MNS1	-0.65	0.35	AR-FL
TSSK4	-0.84	0.35	AR-FL
MYLK	-1.12	0.36	AR-FL
SNX22	-2.01	0.36	AR-FL
PCYOX1L	-0.60	0.36	AR-FL
FLRT1	-0.74	0.37	AR-FL
SEMA6D	-1.49	0.38	AR-FL
PIWIL4	-0.74	0.38	AR-FL
BIRC3	-2.16	0.39	AR-FL
ZNF23	-0.81	0.39	AR-FL
BCL6	-0.82	0.39	AR-FL
YAP1	-0.67	0.40	AR-FL
ALCAM	-1.50	0.41	AR-FL

ARID3A	0.01	-0.85	AR-V7
LAD1	0.11	-0.85	AR-V7
DNPEP	0.18	-0.84	AR-V7
SLC19A1	0.03	-0.82	AR-V7
NVL	0.04	-0.82	AR-V7
SLC25A48	0.05	-0.81	AR-V7
PVRL1	0.13	-0.81	AR-V7
RIMS1	0.30	-0.81	AR-V7
ADAMTS1	0.54	-0.81	AR-V7
R3HDM2	0.52	-0.81	AR-V7
DDAH1	0.20	-0.80	AR-V7
MARVELD1	0.03	-0.79	AR-V7
IMPG1	0.42	-0.79	AR-V7
ADARB1	0.27	-0.78	AR-V7
ME1	0.30	-0.78	AR-V7
ABCE1	0.01	-0.77	AR-V7
FAM222A	0.11	-0.76	AR-V7
DOCK9	0.05	-0.76	AR-V7
ZNF75D	0.02	-0.75	AR-V7
MID2	0.02	-0.75	AR-V7
CDK5R1	0.40	-0.75	AR-V7
GTF3A	0.07	-0.75	AR-V7
TOP2B	0.06	-0.74	AR-V7
AGAP1	0.24	-0.73	AR-V7
RPS6KA6	0.02	-0.72	AR-V7
KCTD1	0.38	-0.72	AR-V7
RELL2	0.46	-0.72	AR-V7
ERBB3	0.07	-0.71	AR-V7
UCK2	0.19	-0.71	AR-V7
TNFSF4	0.48	-0.71	AR-V7
GPRIN1	0.37	-0.71	AR-V7
ACTL10	0.16	-0.70	AR-V7
DDO	0.20	-0.69	AR-V7
ZSCAN31	0.11	-0.69	AR-V7
ZC3HAV1L	0.29	-0.69	AR-V7
LRP3	0.34	-0.69	AR-V7
LITAF	0.12	-0.68	AR-V7
SPTBN2	0.38	-0.68	AR-V7
TUBB6	0.24	-0.68	AR-V7
TNKS2	0.04	-0.68	AR-V7
OGDH	0.27	-0.67	AR-V7
UBE2E2	0.15	-0.67	AR-V7
GLO1	0.03	-0.66	AR-V7
MTCL1	0.36	-0.66	AR-V7
COTL1	0.33	-0.66	AR-V7
ZNF827	0.44	-0.66	AR-V7
CHAC1	-0.66	0.41	AR-FL
TNC	-4.93	0.41	AR-FL
CCDC30	-0.71	0.41	AR-FL
UACA	-0.66	0.42	AR-FL
NAV2	-1.36	0.43	AR-FL
PCBP3	-0.80	0.44	AR-FL
SNX33	-1.08	0.45	AR-FL
PLXNC1	-0.59	0.46	AR-FL
SCN3B	-0.62	0.46	AR-FL
TMEM63C	-1.42	0.47	AR-FL
FOXE1	-1.37	0.47	AR-FL
RADIL	-0.75	0.48	AR-FL
MECOM	-2.28	0.48	AR-FL
GFOD1	-0.79	0.50	AR-FL
JAZF1	-0.66	0.50	AR-FL
DHRSX	-1.10	0.51	AR-FL
TM7SF3	-0.67	0.51	AR-FL
RGS2	-1.09	0.54	AR-FL
ADAM12	-0.94	0.54	AR-FL
IGFBP1	-0.91	0.54	AR-FL
TBC1D32	-0.60	0.54	AR-FL
RNF122	-0.96	0.55	AR-FL
QSOX1	-1.46	0.57	AR-FL
PTGER3	-1.39	0.57	AR-FL
ARHGAP23	-1.32	0.57	AR-FL
FAM229B	-1.27	0.57	AR-FL
ST3GAL5	-0.60	0.59	Opposite
ADGRF1	-2.89	0.60	Opposite
LAMA4	-0.77	0.62	Opposite
PKIB	-1.19	0.62	Opposite
VEGFC	-1.80	0.67	Opposite
WNT11	-2.31	0.67	Opposite
SOX6	-1.41	0.67	Opposite
NEURL1	-1.11	0.67	Opposite
SCARF1	-0.66	0.71	Opposite
SCRN1	-0.70	0.73	Opposite
NPTX1	-0.74	0.74	Opposite
FAM46A	-1.18	0.78	Opposite
CRISPLD1	-1.66	0.78	Opposite
TEX9	-1.24	0.78	Opposite
SYN1	-0.65	0.81	Opposite
RSPH1	-0.76	0.81	Opposite
THBS1	-2.14	0.82	Opposite
MYH15	-1.08	0.83	Opposite
MDH1B	-1.49	0.85	Opposite
CHAC2	-0.71	0.87	Opposite

TUBG1	0.23	-0.65	AR-V7
FAM162A	0.00	-0.65	AR-V7
GMDS	0.25	-0.65	AR-V7
RYR3	0.06	-0.65	AR-V7
PPFIA2	0.01	-0.65	AR-V7
FBXL17	0.27	-0.65	AR-V7
BVES	0.54	-0.65	AR-V7
FAM84B	0.01	-0.64	AR-V7
SERPINB6	0.02	-0.64	AR-V7
IFNAR2	0.21	-0.62	AR-V7
ZNRF2	0.09	-0.61	AR-V7
TBL1XR1	0.07	-0.61	AR-V7
SLC25A5	0.25	-0.61	AR-V7
TXNRD1	0.11	-0.61	AR-V7
MPHOSPH6	0.28	-0.60	AR-V7
SERINC5	0.43	-0.60	AR-V7
DUSP2	0.46	-0.60	AR-V7
SETDB2	0.16	-0.60	AR-V7
NUDT3	0.02	-0.60	AR-V7
EHBP1	0.38	-0.60	AR-V7
GOLM1	0.43	-0.60	AR-V7
TRIM14	0.09	-0.59	AR-V7
WDR4	0.05	-0.59	AR-V7
WDR72	0.07	-0.59	AR-V7
ACTR3C	0.02	-0.59	AR-V7
DAAM1	0.31	-0.58	AR-V7
VAV2	0.02	-0.58	AR-V7
KIAA1107	-0.15	0.58	AR-V7
TCAP	-0.15	0.58	AR-V7
HEMK1	-0.05	0.58	AR-V7
ODF2L	-0.10	0.58	AR-V7
DUSP19	-0.24	0.58	AR-V7
KCNJ11	-0.27	0.59	AR-V7
KLHDC9	-0.11	0.59	AR-V7
LOXL2	-0.39	0.59	AR-V7
KIF6	-0.27	0.59	AR-V7
EIF4A2	-0.14	0.59	AR-V7
INTU	-0.14	0.59	AR-V7
CFAP61	-0.16	0.59	AR-V7
TMEM62	-0.40	0.59	AR-V7
MAP1B	-0.30	0.59	AR-V7
ZC2HC1C	-0.29	0.59	AR-V7
SEC61A2	-0.10	0.60	AR-V7
ARHGAP4	-0.19	0.60	AR-V7
ENKD1	-0.08	0.60	AR-V7
AAMDC	-0.13	0.60	AR-V7
TMEM74B	-0.59	0.87	Opposite
KCNQ3	-0.61	0.88	Opposite
THRB	-0.97	0.89	Opposite
DISC1	-1.58	0.93	Opposite
SLC1A1	-0.84	0.95	Opposite
LSAMP	-1.60	0.95	Opposite
SEMA4G	-0.99	0.95	Opposite
MT1G	-0.63	0.96	Opposite
NR3C2	-1.25	1.01	Opposite
AK8	-1.01	1.01	Opposite
PRIMA1	-0.94	1.03	Opposite
ZNF185	-1.33	1.04	Opposite
CXorf57	-0.62	1.05	Opposite
IGFBP5	-1.12	1.06	Opposite
BTG1	-0.83	1.10	Opposite
ETNK2	-1.05	1.10	Opposite
ABLIM3	-0.71	1.14	Opposite
PDK4	-7.12	1.16	Opposite
ARX	-1.01	1.23	Opposite
ANK2	-0.96	1.36	Opposite
ADRA2B	-1.35	1.40	Opposite
TMEFF2	-0.91	1.44	Opposite
TRPA1	-3.21	1.51	Opposite
OCA2	-1.50	1.53	Opposite
PDZD2	-1.17	1.59	Opposite
GATSL2	-1.06	1.60	Opposite
EPHA4	-1.58	1.64	Opposite
ETV5	-1.37	1.67	Opposite
USP2	-1.57	1.88	Opposite
DUOX1	-1.46	1.90	Opposite
GRIN3A	-0.86	1.91	Opposite
POU3F1	-1.95	1.91	Opposite
CCDC109B	-0.59	1.96	Opposite
MSX2	-0.88	2.07	Opposite
NR5A2	-0.95	2.24	Opposite
ADORA1	-0.81	2.38	Opposite
HOPX	-3.53	2.40	Opposite
NPY2R	-0.71	2.54	Opposite
MAATS1	-1.90	2.66	Opposite
ATP2B2	-2.61	2.69	Opposite
NGFR	-0.62	2.79	Opposite
RASL11B	-1.55	2.81	Opposite
DGKG	-2.38	2.82	Opposite
CHN2	-1.38	2.95	Opposite
KLHL31	-0.84	3.02	Opposite
WISP2	-1.68	3.12	Opposite

SSPO	-0.16	0.60	AR-V7
ULBP3	-0.27	0.60	AR-V7
GNPTAB	-0.31	0.60	AR-V7
STAU2	-0.07	0.61	AR-V7
CCDC78	-0.24	0.61	AR-V7
C14orf79	-0.05	0.61	AR-V7
DNAJA4	-0.01	0.61	AR-V7
CASC1	-0.46	0.61	AR-V7
CITED4	-0.30	0.62	AR-V7
DYX1C1	-0.31	0.62	AR-V7
TECTA	-0.43	0.63	AR-V7
COL1A1	-0.22	0.63	AR-V7
RECK	-0.02	0.63	AR-V7
IQSEC2	-0.03	0.64	AR-V7
VAMP5	-0.40	0.65	AR-V7
SDC3	-0.31	0.65	AR-V7
TRIM17	-0.35	0.65	AR-V7
ELK1	-0.03	0.65	AR-V7
TLE1	-0.20	0.66	AR-V7
ZC2HC1A	-0.13	0.66	AR-V7
RABL2B	-0.18	0.66	AR-V7
NBPF14	-0.30	0.67	AR-V7
TCIRG1	-0.09	0.67	AR-V7
REC8	-0.01	0.67	AR-V7
CEBPB	-0.06	0.67	AR-V7
ZNF66	-0.20	0.67	AR-V7
CCNB1IP1	-0.08	0.68	AR-V7
BBS12	0.00	0.68	AR-V7
C9orf173	-0.44	0.68	AR-V7
FSD1L	-0.25	0.68	AR-V7
ANKRD26	-0.41	0.68	AR-V7
CNPY4	-0.08	0.69	AR-V7
RASSF4	-0.30	0.69	AR-V7
PLXNA1	-0.06	0.69	AR-V7
VWA5A	-0.03	0.69	AR-V7
CPT1B	-0.02	0.70	AR-V7
MKL1	-0.06	0.70	AR-V7
CYSTM1	-0.25	0.70	AR-V7
CD37	-0.18	0.70	AR-V7
TNFAIP8L1	-0.09	0.70	AR-V7
LMNTD2	-0.18	0.71	AR-V7
TMEM64	-0.30	0.71	AR-V7
RBPMS	-0.15	0.71	AR-V7
GPT	-0.40	0.71	AR-V7
RABL2A	-0.29	0.71	AR-V7
SLC12A2	-0.20	0.72	AR-V7
NCMAP	-1.50	3.18	Opposite
TAGAP	-1.53	3.23	Opposite
UPP1	-0.72	3.24	Opposite
MYH7	-4.03	3.29	Opposite
PSTPIP2	-0.65	3.59	Opposite
TEK	-1.22	4.09	Opposite
UBE2QL1	-2.45	4.31	Opposite
ACVR1C	-1.78	4.44	Opposite
SULT1C2	-0.58	4.77	Opposite
PLXNA4	-1.13	5.04	Opposite
PROC	-0.84	5.25	Opposite
CABS1	-2.28	5.27	Opposite
UGT3A2	0.38	-3.24	AR-V7
SP8	0.09	-1.70	AR-V7
DNAJC22	0.29	-1.33	AR-V7
CSTA	0.25	-1.32	AR-V7
ABCG1	0.14	-1.32	AR-V7
MAGEB17	0.28	-1.31	AR-V7
DNAH8	0.46	-1.27	AR-V7
HAO1	0.27	-1.26	AR-V7
INPP4B	0.06	-1.25	AR-V7
FGFR2	0.22	-1.24	AR-V7
HGD	0.32	-1.23	AR-V7
FFAR2	0.03	-1.17	AR-V7
KCNH8	0.26	-1.14	AR-V7
SPTSSB	0.25	-1.14	AR-V7
TMC1	0.04	-1.08	AR-V7
PLA2R1	0.23	-1.07	AR-V7
FRMD5	0.50	-1.07	AR-V7
SERPINA1	0.33	-1.02	AR-V7
LCP1	0.23	-1.00	AR-V7
KCNK3	0.12	-0.97	AR-V7
MGAT4A	0.22	-0.96	AR-V7
LNX1	0.46	-0.94	AR-V7
LHFP	0.25	-0.93	AR-V7
RUNX2	0.05	-0.93	AR-V7
ITGA6	0.11	-0.92	AR-V7
LRP2BP	0.19	-0.89	AR-V7
REPS2	0.26	-0.89	AR-V7
NCALD	0.35	-0.88	AR-V7
SPRED1	0.22	-0.88	AR-V7
TMEM133	0.07	-0.87	AR-V7
STAP2	0.23	-0.87	AR-V7
CGNL1	0.17	-0.83	AR-V7
CMTM8	0.14	-0.82	AR-V7
IGSF21	0.50	-0.80	AR-V7

RANBP17	-0.17	0.72	AR-V7
HSPA12B	-0.09	0.72	AR-V7
CDH8	-0.40	0.73	AR-V7
GGT7	-0.21	0.73	AR-V7
ACYP1	-0.23	0.73	AR-V7
WDR19	-0.16	0.73	AR-V7
FRMD4B	-0.42	0.73	AR-V7
TMC2	-0.27	0.74	AR-V7
UACA	-0.43	0.74	AR-V7
ZNF467	-0.21	0.74	AR-V7
CCDC189	-0.22	0.74	AR-V7
C8G	-0.14	0.75	AR-V7
LIPH	-0.54	0.75	AR-V7
MOK	-0.07	0.75	AR-V7
PIANP	-0.26	0.76	AR-V7
ARNTL2	-0.38	0.77	AR-V7
FAM217B	-0.55	0.78	AR-V7
C1orf228	-0.53	0.78	AR-V7
TM7SF3	-0.51	0.78	AR-V7
PCSK4	-0.10	0.78	AR-V7
LCN12	-0.54	0.78	AR-V7
SBSPON	-0.28	0.78	AR-V7
CCDC62	-0.57	0.79	AR-V7
GOLT1A	-0.23	0.80	AR-V7
CDC14A	-0.53	0.80	AR-V7
NBN	-0.07	0.81	AR-V7
MIDN	-0.15	0.81	AR-V7
SLC16A9	-0.01	0.81	AR-V7
ALKBH6	-0.02	0.82	AR-V7
TJP3	-0.20	0.83	AR-V7
PDE4A	-0.51	0.83	AR-V7
AQP1	-0.21	0.83	AR-V7
NOXRED1	-0.26	0.83	AR-V7
ZNF502	-0.04	0.83	AR-V7
ZNF185	-0.38	0.83	AR-V7
PRICKLE4	-0.37	0.83	AR-V7
TSC22D3	-0.06	0.83	AR-V7
AIFM2	-0.34	0.83	AR-V7
ARX	-0.31	0.83	AR-V7
AKAP7	-0.11	0.84	AR-V7
RNF32	-0.21	0.84	AR-V7
GRAMD3	-0.10	0.84	AR-V7
CCDC30	-0.39	0.84	AR-V7
POGLUT1	-0.29	0.85	AR-V7
TMEM56-RWDD3	-0.11	0.85	AR-V7
TTC21A	-0.12	0.85	AR-V7
GPX2	0.15	-0.79	AR-V7
ACOX2	0.40	-0.78	AR-V7
SLA2	0.58	-0.77	AR-V7
THSD4	0.31	-0.77	AR-V7
OVGP1	0.22	-0.76	AR-V7
IGSF5	0.16	-0.76	AR-V7
TBX2	0.36	-0.76	AR-V7
B4GALNT3	0.39	-0.76	AR-V7
SRPX	0.06	-0.75	AR-V7
TACSTD2	0.43	-0.73	AR-V7
BBS4	0.52	-0.70	AR-V7
PLD2	0.03	-0.70	AR-V7
SH3KBP1	0.08	-0.69	AR-V7
GPR160	0.12	-0.68	AR-V7
CDK17	0.28	-0.66	AR-V7
SDK2	0.23	-0.66	AR-V7
PKP1	0.36	-0.65	AR-V7
LENG9	0.16	-0.65	AR-V7
FAM162A	0.07	-0.64	AR-V7
ENTPD5	0.58	-0.63	AR-V7
SMPDL3B	0.02	-0.63	AR-V7
RUNX1	0.05	-0.62	AR-V7
TPM4	0.26	-0.62	AR-V7
ONECUT2	0.07	-0.60	AR-V7
RTN2	0.17	-0.60	AR-V7
USP53	0.00	-0.59	AR-V7
EML6	0.22	-0.59	AR-V7
CARD9	0.28	-0.59	AR-V7
PREX1	0.24	-0.59	AR-V7
DNAH2	0.05	-0.58	AR-V7
NRTN	-0.28	0.58	AR-V7
SLC6A8	-0.09	0.58	AR-V7
DUSP9	-0.15	0.60	AR-V7
PER2	-0.25	0.60	AR-V7
MASP2	-0.08	0.60	AR-V7
QSER1	-0.41	0.60	AR-V7
C11orf54	-0.06	0.60	AR-V7
DGCR6	-0.07	0.61	AR-V7
PGAP2	-0.25	0.61	AR-V7
APOH	-0.31	0.62	AR-V7
FBXO21	-0.03	0.63	AR-V7
SRRM5	-0.05	0.63	AR-V7
EME2	-0.03	0.63	AR-V7
MXD3	-0.53	0.64	AR-V7
ELK1	-0.32	0.64	AR-V7
ITPR2	-0.30	0.64	AR-V7

RIPK2	-0.21	0.86	AR-V7
WDR66	-0.03	0.86	AR-V7
CATIP	-0.02	0.86	AR-V7
MAP2K6	-0.49	0.87	AR-V7
SLC6A16	-0.30	0.87	AR-V7
UNC45A	-0.09	0.88	AR-V7
HPCAL1	-0.02	0.89	AR-V7
SYNGR3	-0.03	0.89	AR-V7
PRUNE2	-0.19	0.89	AR-V7
CFAP43	-0.10	0.89	AR-V7
CFAP53	-0.10	0.89	AR-V7
LIX1L	-0.02	0.89	AR-V7
RASL11A	-0.08	0.89	AR-V7
KIF21B	-0.34	0.90	AR-V7
TSNAXIP1	-0.09	0.90	AR-V7
TAS1R3	-0.21	0.90	AR-V7
METRNL	-0.20	0.91	AR-V7
TNFSF15	-0.21	0.92	AR-V7
TRIP6	-0.12	0.93	AR-V7
RNF144B	-0.27	0.94	AR-V7
MAATS1	-0.39	0.94	AR-V7
AMH	-0.41	0.94	AR-V7
C17orf97	-0.14	0.94	AR-V7
DAB2IP	-0.56	0.94	AR-V7
SGSM1	-0.27	0.95	AR-V7
DKKL1	-0.07	0.95	AR-V7
EDA	-0.55	0.95	AR-V7
SLC7A7	-0.06	0.96	AR-V7
ARSK	-0.27	0.96	AR-V7
RAB31	-0.38	0.96	AR-V7
MSH4	-0.13	0.97	AR-V7
FUOM	-0.54	0.98	AR-V7
MORN3	-0.26	0.98	AR-V7
ITPR3	-0.01	0.98	AR-V7
ARHGAP28	-0.14	0.99	AR-V7
ID1	-0.20	0.99	AR-V7
APOF	-0.38	1.01	AR-V7
SLC41A2	-0.31	1.01	AR-V7
CHST3	-0.07	1.02	AR-V7
CDKN1A	-0.36	1.03	AR-V7
FUT3	-0.13	1.04	AR-V7
MFGE8	-0.18	1.04	AR-V7
ZSCAN18	-0.26	1.04	AR-V7
NR3C2	-0.09	1.04	AR-V7
ADAMTSL5	-0.27	1.04	AR-V7

C18orf54	-0.44	0.66	AR-V7
ANOS1	-0.43	0.66	AR-V7
C5orf34	-0.51	0.67	AR-V7
TUBGCP6	-0.12	0.67	AR-V7
GMPR	-0.24	0.68	AR-V7
PNRC2	-0.48	0.69	AR-V7
NPIPB11	-0.05	0.69	AR-V7
PHTF2	-0.10	0.70	AR-V7
VPS33B	-0.16	0.70	AR-V7
AHNAK	-0.27	0.71	AR-V7
ENC1	-0.45	0.71	AR-V7
DGKH	-0.02	0.71	AR-V7
WDR62	-0.02	0.71	AR-V7
SMC6	-0.28	0.71	AR-V7
LIG1	-0.20	0.72	AR-V7
NBN	-0.27	0.72	AR-V7
ITM2C	-0.16	0.73	AR-V7
DPP6	-0.35	0.74	AR-V7
PLEKHG4B	-0.14	0.74	AR-V7
C17orf97	-0.29	0.75	AR-V7
GLDC	-0.04	0.75	AR-V7
IL10RA	-0.14	0.75	AR-V7
CEP152	-0.13	0.76	AR-V7
POGLUT1	-0.42	0.76	AR-V7
NUAK2	-0.53	0.77	AR-V7
UNC5B	-0.33	0.77	AR-V7
RMI2	-0.05	0.78	AR-V7
HSPA4L	-0.34	0.80	AR-V7
CYP46A1	-0.07	0.80	AR-V7
KANK1	-0.35	0.80	AR-V7
APITD1	-0.11	0.81	AR-V7
CFAP69	-0.35	0.83	AR-V7
MRPS25	-0.41	0.83	AR-V7
SDC3	-0.33	0.84	AR-V7
ZNF287	-0.09	0.85	AR-V7
TECTA	-0.17	0.86	AR-V7
NETO1	-0.20	0.86	AR-V7
CASK	-0.25	0.88	AR-V7
HELZ2	-0.07	0.88	AR-V7
NRCAM	-0.55	0.89	AR-V7
RP5-1187M17.10	-0.47	0.90	AR-V7
CES2	-0.48	0.91	AR-V7
RAP2A	-0.44	0.93	AR-V7
PARP12	-0.40	0.94	AR-V7
BRCA2	-0.04	0.95	AR-V7

SPAG4	-0.14	1.04	AR-V7	UNC5A	-0.38	0.95	AR-V7
METTL7A	-0.22	1.04	AR-V7	PRDM1	-0.25	0.98	AR-V7
CNTNAP1	-0.20	1.04	AR-V7	MPV17L	-0.28	0.99	AR-V7
GADD45A	-0.30	1.04	AR-V7	ARNTL	-0.27	1.00	AR-V7
RCCD1	-0.51	1.05	AR-V7	FAM101B	-0.34	1.01	AR-V7
C12orf75	-0.20	1.05	AR-V7	IFT57	-0.58	1.02	AR-V7
MLLT3	-0.21	1.05	AR-V7	TGS1	-0.04	1.03	AR-V7
NHLRC1	-0.33	1.07	AR-V7	MYBL1	-0.22	1.03	AR-V7
GUCY1A2	-0.39	1.07	AR-V7	ZNF84	-0.35	1.05	AR-V7
NDUFAF5	-0.01	1.08	AR-V7	PRKAR2B	-0.11	1.05	AR-V7
IQCD	-0.32	1.09	AR-V7	IQUB	-0.02	1.05	AR-V7
IFIT2	-0.37	1.09	AR-V7	B4GALT6	-0.35	1.06	AR-V7
PTPRG	-0.34	1.10	AR-V7	ASIC3	-0.09	1.06	AR-V7
DLL1	-0.21	1.10	AR-V7	CADPS2	-0.13	1.06	AR-V7
JADE1	-0.09	1.11	AR-V7	ARMCX4	-0.20	1.10	AR-V7
UVRAG	-0.19	1.11	AR-V7	CSPG5	-0.14	1.11	AR-V7
SELO	-0.12	1.11	AR-V7	TSNAXIP1	-0.36	1.12	AR-V7
COL4A2	-0.29	1.12	AR-V7	MAMLD1	-0.46	1.15	AR-V7
PAQR5	-0.07	1.12	AR-V7	C3orf58	-0.22	1.18	AR-V7
PARP14	-0.26	1.12	AR-V7	SPATA13	-0.16	1.20	AR-V7
FSIP1	-0.34	1.14	AR-V7	RND3	-0.33	1.20	AR-V7
TAF1L	-0.21	1.14	AR-V7	IER5L	-0.08	1.23	AR-V7
CCDC191	-0.01	1.15	AR-V7	ADRA2A	-0.23	1.25	AR-V7
KANK3	-0.40	1.15	AR-V7	HYDIN	-0.12	1.26	AR-V7
NUDT8	-0.13	1.16	AR-V7	PTPRG	-0.07	1.29	AR-V7
TUBE1	-0.46	1.16	AR-V7	TACC2	-0.08	1.34	AR-V7
MYO1F	-0.25	1.19	AR-V7	SELO	-0.21	1.35	AR-V7
TNFRSF12A	-0.48	1.21	AR-V7	ZNF189	-0.05	1.47	AR-V7
CCNJ	-0.03	1.22	AR-V7	HAUS7	-0.01	1.49	AR-V7
LINGO4	-0.26	1.23	AR-V7	GCLC	-0.49	1.54	AR-V7
PRKAR2B	-0.48	1.24	AR-V7	PDGFC	-0.10	1.56	AR-V7
HIST1H4E	-0.02	1.24	AR-V7	TNS1	-0.38	1.58	AR-V7
PHLDA1	-0.24	1.24	AR-V7	ABCC3	-0.54	1.72	AR-V7
IGF2	-0.27	1.27	AR-V7	PPP1R1B	-0.42	1.75	AR-V7
SCNN1A	-0.41	1.27	AR-V7	CYP4F3	-0.54	1.79	AR-V7
LRRIQ1	-0.03	1.31	AR-V7	FRMD4B	-0.03	1.89	AR-V7
FAM161B	-0.03	1.31	AR-V7	CACNA1G	-0.37	1.90	AR-V7
ATL1	-0.27	1.34	AR-V7	CADPS	-0.35	1.90	AR-V7
S100A10	-0.37	1.36	AR-V7	KCNG1	-0.22	1.91	AR-V7
PTGFR	-0.23	1.37	AR-V7	PHLPP2	-0.12	1.93	AR-V7
FABP6	-0.35	1.37	AR-V7	AUTS2	-0.40	1.94	AR-V7
LGI4	-0.29	1.38	AR-V7	DOK4	0.00	2.01	AR-V7
TTC16	-0.35	1.40	AR-V7	SGPP2	-0.32	2.02	AR-V7
HIST2H2AA4	-0.27	1.40	AR-V7	PMAIP1	-0.18	2.16	AR-V7
MICAL2	-0.06	1.40	AR-V7	SPSB1	-0.17	2.19	AR-V7
PDLIM1	-0.13	1.43	AR-V7	PAPPA2	-0.29	2.54	AR-V7

PHTF2	-0.36	1.43	AR-V7	ANO1	-0.19	2.57	AR-V7
HADH	-0.16	1.47	AR-V7	ADRA2C	-0.15	2.63	AR-V7
ALDH3B2	-0.37	1.49	AR-V7	GRIK4	-0.01	3.62	AR-V7
PLIN2	-0.14	1.51	AR-V7	AFF2	-0.20	3.96	AR-V7
RNF112	-0.05	1.53	AR-V7	SERPINA5	-0.05	4.52	AR-V7
PCDHB8	-0.21	1.55	AR-V7	SCNN1A	-0.26	4.71	AR-V7
MSRB3	-0.39	1.55	AR-V7	MS4A2	-0.34	4.73	AR-V7
MMP25	-0.42	1.57	AR-V7	SCG2	-0.17	4.92	AR-V7
TSPAN12	-0.07	1.61	AR-V7	NRG2	-0.53	4.98	AR-V7
P2RX2	-0.06	1.62	AR-V7	CFAP46	-0.06	5.12	AR-V7
DNAAF1	-0.17	1.67	AR-V7	LAPTM5	-0.05	5.21	AR-V7
ABCC8	-0.21	1.69	AR-V7	CD99	-0.28	5.97	AR-V7
PXYLP1	-0.30	1.74	AR-V7	TMC2	-0.06	6.32	AR-V7
AKNA	-0.09	1.75	AR-V7	NTS	-4.56	-4.28	Both Isoforms
TMEM74B	-0.31	1.80	AR-V7	ATF6B	-1.09	-4.08	Both Isoforms
IL32	-0.48	1.81	AR-V7	WDR46	-0.80	-3.87	Both Isoforms
ZNF208	-0.06	1.86	AR-V7	TDGF1	-4.03	-3.20	Both Isoforms
ARL4A	-0.29	1.87	AR-V7	ABCB5	-3.13	-3.02	Both Isoforms
SAMD4A	-0.12	1.88	AR-V7	TMPRSS11F	-1.53	-2.95	Both Isoforms
DYNC1I1	-0.27	1.88	AR-V7	LRRC2	-5.14	-2.93	Both Isoforms
OPRL1	-0.23	1.93	AR-V7	SMCO3	-3.96	-2.80	Both Isoforms
HBEGF	-0.01	1.98	AR-V7	SCUBE2	-3.31	-2.76	Both Isoforms
CACNA1H	-0.31	1.98	AR-V7	LMX1A	-2.62	-2.76	Both Isoforms
SYCP3	-0.44	2.04	AR-V7	ADGRL3	-9.02	-2.75	Both Isoforms
ALCAM	-0.18	2.05	AR-V7	C2CD4A	-4.24	-2.71	Both Isoforms
SSFA2	-0.32	2.07	AR-V7	ALS2CL	-2.50	-2.68	Both Isoforms
ABCA4	-0.09	2.11	AR-V7	SALL4	-3.05	-2.66	Both Isoforms
PPP1R1B	-0.35	2.15	AR-V7	COL14A1	-4.36	-2.65	Both Isoforms
STAB1	-0.31	2.16	AR-V7	ASZ1	-7.70	-2.64	Both Isoforms
CSPG4	-0.28	2.22	AR-V7	HABP2	-3.68	-2.59	Both Isoforms
FAM229B	-0.42	2.30	AR-V7	CPED1	-4.00	-2.58	Both Isoforms
HIST2H2AA3	-0.20	2.31	AR-V7	GCG	-3.81	-2.49	Both Isoforms
HSPB8	-0.26	2.33	AR-V7	B3GNT6	-4.31	-2.47	Both Isoforms
PDZK1IP1	-0.55	2.40	AR-V7	PPFIA2	-4.43	-2.41	Both Isoforms
EFHD1	-0.30	2.44	AR-V7	DCHS2	-5.38	-2.41	Both Isoforms
CYP3A5	-0.02	2.56	AR-V7	INHBA	-2.40	-2.39	Both Isoforms
ADD3	-0.31	2.60	AR-V7	CTTNBP2	-4.11	-2.38	Both Isoforms
CNTN5	-0.37	2.61	AR-V7	PTPRR	-2.77	-2.37	Both Isoforms
RDH10	-0.45	2.63	AR-V7	IFIT3	-1.85	-2.36	Both Isoforms
CACNA2D4	-0.03	2.79	AR-V7	CTNNA3	-4.59	-2.34	Both Isoforms
CDK18	-0.42	2.82	AR-V7	REG4	-3.53	-2.33	Both Isoforms
IRGM	-0.35	3.11	AR-V7	FOXQ1	-3.26	-2.33	Both Isoforms
NLGN4X	-0.28	3.11	AR-V7	SOX30	-4.04	-2.32	Both Isoforms
NPY1R	-0.28	3.15	AR-V7	CA8	-3.55	-2.32	Both Isoforms
PDZD2	-0.58	3.43	AR-V7	BEND5	-4.15	-2.31	Both Isoforms
PGF	-0.52	3.78	AR-V7	GPRIN3	-1.91	-2.30	Both Isoforms

CRISPLD1	-0.25	3.96	AR-V7	KRT2	-1.03	-2.30	Both Isoforms
CLDN1	-0.35	4.55	AR-V7	LUM	-4.94	-2.26	Both Isoforms
TRIM49	-0.56	4.77	AR-V7	NPIPA2	-0.85	-2.26	Both Isoforms
PAGE1	-0.30	4.90	AR-V7	SATB1	-4.46	-2.25	Both Isoforms
FIBCD1	-0.06	5.75	AR-V7	CAV1	-2.74	-2.22	Both Isoforms
TRIM49C	-0.51	5.87	AR-V7	SLC4A4	-1.29	-2.21	Both Isoforms
SIGLEC1	-0.11	6.45	AR-V7	ABCA1	-3.22	-2.20	Both Isoforms
PROC	-0.54	7.11	AR-V7	CACNA2D3	-5.03	-2.17	Both Isoforms
NTNG1	1.72	-5.43	Opposite	NAALAD2	-4.28	-2.16	Both Isoforms
RGS16	0.67	-4.49	Opposite	TNFSF10	-1.24	-2.15	Both Isoforms
KCNN2	1.63	-3.39	Opposite	LTF	-2.96	-2.14	Both Isoforms
COL12A1	2.40	-3.27	Opposite	NAV3	-1.49	-2.12	Both Isoforms
TWIST1	2.83	-3.26	Opposite	CFTR	-5.31	-2.11	Both Isoforms
PARM1	2.02	-2.97	Opposite	PGM5	-4.11	-2.11	Both Isoforms
FEV	1.28	-2.67	Opposite	PTPRB	-3.50	-2.10	Both Isoforms
MYOF	1.33	-2.64	Opposite	C12orf60	-2.13	-2.10	Both Isoforms
IL1RAPL1	0.59	-2.62	Opposite	C8orf4	-0.86	-2.10	Both Isoforms
PALMD	0.76	-2.53	Opposite	IL33	-7.33	-2.09	Both Isoforms
AIM1	1.06	-2.42	Opposite	TSPAN12	-2.48	-2.09	Both Isoforms
SDR9C7	3.18	-2.25	Opposite	SLC7A14	-4.29	-2.09	Both Isoforms
DUSP27	0.62	-2.17	Opposite	DLGAP2	-3.04	-2.08	Both Isoforms
LXN	2.13	-2.14	Opposite	LHX9	-4.23	-2.06	Both Isoforms
PDE3A	6.91	-2.13	Opposite	AOX1	-2.25	-2.05	Both Isoforms
TMEM150C	2.17	-1.97	Opposite	WNT2	-6.81	-2.04	Both Isoforms
FAM65B	1.35	-1.96	Opposite	APELA	-2.32	-2.04	Both Isoforms
ETV1	0.60	-1.83	Opposite	UGT2B15	-5.32	-2.03	Both Isoforms
APOL3	0.69	-1.79	Opposite	IL1RAPL1	-2.55	-2.03	Both Isoforms
CNTNAP2	1.66	-1.79	Opposite	FLT3	-3.85	-2.02	Both Isoforms
BEND4	1.53	-1.76	Opposite	GLIPR1	-3.00	-2.02	Both Isoforms
SLC22A10	1.25	-1.48	Opposite	RAPGEF4	-1.88	-1.99	Both Isoforms
TRPM8	3.96	-1.47	Opposite	WNT5B	-2.26	-1.99	Both Isoforms
CNGB3	1.64	-1.47	Opposite	CXXC4	-3.75	-1.99	Both Isoforms
KCNJ2	1.09	-1.43	Opposite	SNTB1	-4.62	-1.99	Both Isoforms
IGSF5	1.36	-1.34	Opposite	PI15	-2.81	-1.99	Both Isoforms
KREMEN2	0.73	-1.31	Opposite	IL2RG	-2.04	-1.99	Both Isoforms
MAGEA1	0.63	-1.28	Opposite	RNF125	-2.34	-1.98	Both Isoforms
FGFR3	1.05	-1.26	Opposite	CDCA7L	-2.69	-1.98	Both Isoforms
HHIPL2	3.54	-1.24	Opposite	TNFSF15	-4.08	-1.96	Both Isoforms
OSBPL7	1.06	-1.20	Opposite	TPK1	-1.01	-1.96	Both Isoforms
CYP1A1	0.67	-1.19	Opposite	NIPSNAP3B	-4.41	-1.96	Both Isoforms
SLC43A1	1.10	-1.19	Opposite	PCED1B	-2.74	-1.95	Both Isoforms
RAI2	0.85	-1.19	Opposite	CLEC3A	-7.84	-1.95	Both Isoforms
DEGS1	0.88	-1.18	Opposite	THEG	-2.24	-1.92	Both Isoforms
FAT1	0.89	-1.18	Opposite	FST	-6.77	-1.92	Both Isoforms
KLHL29	1.33	-1.10	Opposite	ERP27	-3.84	-1.92	Both Isoforms
DSC1	3.38	-1.10	Opposite	TPO	-0.87	-1.91	Both Isoforms

RANBP3L	1.14	-1.08	Opposite	MCC	-1.48	-1.90	Both Isoforms
IL20RA	1.16	-1.06	Opposite	LIX1	-3.32	-1.88	Both Isoforms
INHBE	0.63	-1.06	Opposite	WIPF1	-1.18	-1.88	Both Isoforms
CLDN8	1.27	-1.04	Opposite	ALPL	-2.75	-1.87	Both Isoforms
SERPINE2	0.64	-1.04	Opposite	MAN1A1	-2.55	-1.87	Both Isoforms
GCNT2	0.94	-1.03	Opposite	PDE7B	-2.75	-1.87	Both Isoforms
WNT5A	1.40	-1.03	Opposite	ESRRG	-1.08	-1.87	Both Isoforms
MAGEC2	0.77	-0.99	Opposite	NRIP3	-2.46	-1.87	Both Isoforms
ARFGEF3	0.92	-0.99	Opposite	GIMAP2	-2.55	-1.86	Both Isoforms
RFPL2	1.70	-0.98	Opposite	IGFBP3	-4.48	-1.86	Both Isoforms
LCP1	1.70	-0.97	Opposite	ARHGAP20	-4.24	-1.85	Both Isoforms
FMO5	0.67	-0.95	Opposite	PRKG2	-2.00	-1.85	Both Isoforms
PPM1E	0.66	-0.95	Opposite	FREM2	-1.93	-1.85	Both Isoforms
MESP1	0.99	-0.94	Opposite	IPCEF1	-2.03	-1.84	Both Isoforms
C9orf152	1.46	-0.91	Opposite	SULF1	-4.78	-1.84	Both Isoforms
TACSTD2	0.84	-0.90	Opposite	UPB1	-2.85	-1.83	Both Isoforms
GRPR	2.94	-0.90	Opposite	SYTL5	-2.09	-1.83	Both Isoforms
WNT7B	1.67	-0.87	Opposite	C1QTNF1	-2.29	-1.82	Both Isoforms
ADORA2A	2.21	-0.86	Opposite	FOLH1	-3.22	-1.82	Both Isoforms
GNB4	2.08	-0.86	Opposite	SYTL2	-3.72	-1.82	Both Isoforms
GADD45G	0.88	-0.86	Opposite	HS3ST3B1	-4.61	-1.82	Both Isoforms
SMAP1	1.03	-0.84	Opposite	C8orf74	-1.10	-1.82	Both Isoforms
RBM47	0.62	-0.81	Opposite	DIXDC1	-2.46	-1.81	Both Isoforms
RAP1GAP	1.09	-0.80	Opposite	DENND2A	-2.81	-1.81	Both Isoforms
AQP4	3.34	-0.79	Opposite	ROS1	-3.87	-1.81	Both Isoforms
SRSF12	1.54	-0.77	Opposite	SLC24A3	-2.19	-1.80	Both Isoforms
SASH1	1.94	-0.76	Opposite	EBF3	-2.26	-1.80	Both Isoforms
OAS1	0.82	-0.76	Opposite	CCDC83	-3.52	-1.80	Both Isoforms
RGMB	0.82	-0.74	Opposite	AC021106.1	-2.74	-1.80	Both Isoforms
CAMK2N2	0.96	-0.74	Opposite	MAT1A	-3.27	-1.80	Both Isoforms
MED12L	0.61	-0.73	Opposite	PEAK1	-2.23	-1.79	Both Isoforms
SEMA3C	1.75	-0.73	Opposite	BNC2	-3.04	-1.79	Both Isoforms
PKP1	3.97	-0.71	Opposite	UGT2B10	-7.56	-1.78	Both Isoforms
LRRC36	2.09	-0.70	Opposite	GPR85	-5.55	-1.78	Both Isoforms
MZB1	1.31	-0.70	Opposite	GRM4	-1.92	-1.78	Both Isoforms
HLA-C	0.76	-0.69	Opposite	CYBRD1	-2.61	-1.78	Both Isoforms
TJP1	0.83	-0.69	Opposite	SOX11	-2.33	-1.77	Both Isoforms
PROB1	0.99	-0.65	Opposite	LRRC31	-1.70	-1.77	Both Isoforms
TNS3	2.13	-0.65	Opposite	KLF5	-1.26	-1.77	Both Isoforms
SP9	3.14	-0.63	Opposite	DACH1	-3.10	-1.76	Both Isoforms
GALNT18	0.60	-0.62	Opposite	VAV3	-3.15	-1.76	Both Isoforms
CDC42BPA	0.62	-0.60	Opposite	RUNDC3B	-3.31	-1.76	Both Isoforms
OPHN1	1.03	-0.60	Opposite	ADAM19	-3.58	-1.76	Both Isoforms
DDR2	0.97	-0.58	Opposite	LURAP1L	-2.17	-1.75	Both Isoforms
RAPGEF5	0.70	-0.58	AR-FL	LRRIQ4	-1.32	-1.75	Both Isoforms
DEFB132	1.71	-0.55	AR-FL	EDARADD	-0.82	-1.75	Both Isoforms

LRCH1	2.49	-0.55	AR-FL
TTC39A	0.88	-0.55	AR-FL
CPEB2	0.70	-0.53	AR-FL
MMEL1	0.93	-0.52	AR-FL
MIA3	0.82	-0.50	AR-FL
ZNF385B	2.24	-0.50	AR-FL
SLC5A4	1.73	-0.49	AR-FL
TNK2	0.92	-0.49	AR-FL
NEO1	0.63	-0.48	AR-FL
LSM6	0.59	-0.47	AR-FL
JAM3	1.56	-0.47	AR-FL
KAT2B	0.96	-0.47	AR-FL
OLA1	0.76	-0.47	AR-FL
TTC39C	0.81	-0.46	AR-FL
CDC42EP2	0.87	-0.45	AR-FL
DPP4	1.72	-0.44	AR-FL
PLA2G4F	0.68	-0.44	AR-FL
HERC6	0.60	-0.44	AR-FL
USP10	0.87	-0.44	AR-FL
DNASE2B	0.78	-0.43	AR-FL
PRRT2	0.87	-0.42	AR-FL
LRP4	2.04	-0.42	AR-FL
RNF223	0.85	-0.42	AR-FL
SMARCA1	1.23	-0.40	AR-FL
ST6GAL1	0.91	-0.40	AR-FL
GRHL2	0.97	-0.39	AR-FL
VSIG10L	0.75	-0.39	AR-FL
NCAPD3	2.52	-0.37	AR-FL
GPR158	1.70	-0.37	AR-FL
KCTD3	1.15	-0.37	AR-FL
CHD7	0.85	-0.36	AR-FL
EXOC6	0.60	-0.35	AR-FL
SNX25	1.65	-0.35	AR-FL
ALDH1A3	2.89	-0.35	AR-FL
HID1	0.91	-0.34	AR-FL
ADRBK1	0.67	-0.33	AR-FL
MYCBP2	0.77	-0.33	AR-FL
GNA12	1.04	-0.33	AR-FL
GALK2	0.68	-0.32	AR-FL
PAPSS1	0.86	-0.32	AR-FL
TEX2	1.21	-0.32	AR-FL
NPDC1	0.78	-0.32	AR-FL
NSMAF	1.16	-0.32	AR-FL
TRIM33	1.00	-0.31	AR-FL
SPECC1L	1.40	-0.30	AR-FL
HMG20B	0.66	-0.30	AR-FL
COL5A2	-3.65	-1.75	Both Isoforms
OSBPL6	-0.81	-1.74	Both Isoforms
MUCL1	-2.32	-1.74	Both Isoforms
BMP2	-3.84	-1.74	Both Isoforms
C11orf45	-3.96	-1.74	Both Isoforms
SEMA3E	-1.77	-1.73	Both Isoforms
SLC13A2	-4.16	-1.73	Both Isoforms
HIST4H4	-3.68	-1.73	Both Isoforms
TFPI	-4.47	-1.72	Both Isoforms
PDGFRB	-3.21	-1.72	Both Isoforms
NUAK1	-1.10	-1.71	Both Isoforms
IL36RN	-2.85	-1.70	Both Isoforms
BCL11A	-2.97	-1.70	Both Isoforms
ZNF521	-3.51	-1.70	Both Isoforms
REEP1	-2.36	-1.69	Both Isoforms
GPR149	-3.83	-1.69	Both Isoforms
STXBP5L	-2.46	-1.69	Both Isoforms
PACRG	-2.73	-1.69	Both Isoforms
RBP4	-3.43	-1.69	Both Isoforms
UGT2B17	-9.65	-1.68	Both Isoforms
KIAA1210	-3.02	-1.68	Both Isoforms
SV2B	-0.65	-1.68	Both Isoforms
IHH	-1.69	-1.68	Both Isoforms
NAP1L3	-3.33	-1.67	Both Isoforms
CCDC80	-3.31	-1.67	Both Isoforms
SEMA5A	-0.75	-1.66	Both Isoforms
NTRK2	-2.74	-1.65	Both Isoforms
P2RY1	-2.07	-1.65	Both Isoforms
STON1	-1.17	-1.64	Both Isoforms
BACE2	-1.85	-1.64	Both Isoforms
C11orf85	-2.66	-1.64	Both Isoforms
TM4SF20	-3.86	-1.63	Both Isoforms
POF1B	-2.13	-1.63	Both Isoforms
CNGA1	-0.92	-1.63	Both Isoforms
NT5E	-2.50	-1.62	Both Isoforms
ANXA3	-1.11	-1.62	Both Isoforms
EPHA2	-1.25	-1.62	Both Isoforms
LEPR	-1.98	-1.62	Both Isoforms
POU2F3	-2.39	-1.62	Both Isoforms
KLRG1	-0.96	-1.61	Both Isoforms
TLR1	-4.15	-1.61	Both Isoforms
NEGR1	-3.55	-1.60	Both Isoforms
FZD10	-2.14	-1.60	Both Isoforms
MET	-4.25	-1.60	Both Isoforms
AKR1C3	-2.96	-1.60	Both Isoforms
FAM110C	-1.36	-1.60	Both Isoforms

ZNF608	1.16	-0.30	AR-FL	LYPD6B	-2.05	-1.59	Both Isoforms
DOCK5	0.80	-0.30	AR-FL	CSRP2	-2.15	-1.59	Both Isoforms
CNDP2	0.83	-0.29	AR-FL	FBN1	-2.22	-1.58	Both Isoforms
KIF22	0.75	-0.29	AR-FL	MGP	-2.64	-1.58	Both Isoforms
FNIP2	0.76	-0.29	AR-FL	CACNB2	-2.42	-1.58	Both Isoforms
RAB3D	0.79	-0.29	AR-FL	B3GALT5	-2.76	-1.58	Both Isoforms
GCFC2	0.88	-0.29	AR-FL	SHH	-4.69	-1.58	Both Isoforms
SLC2A4	1.21	-0.27	AR-FL	AMIGO2	-3.70	-1.58	Both Isoforms
SYTL1	0.67	-0.26	AR-FL	SERPINI1	-2.37	-1.57	Both Isoforms
MAP7	0.77	-0.26	AR-FL	SAMD13	-1.08	-1.57	Both Isoforms
IQGAP2	2.39	-0.26	AR-FL	FAM19A2	-1.87	-1.56	Both Isoforms
VPS26B	1.47	-0.25	AR-FL	TXNRD2	-1.74	-1.56	Both Isoforms
SYNGR2	0.83	-0.24	AR-FL	SLC30A10	-3.02	-1.56	Both Isoforms
FHIT	1.37	-0.24	AR-FL	EDNRA	-2.56	-1.56	Both Isoforms
EMC2	0.75	-0.24	AR-FL	ZNF711	-3.30	-1.55	Both Isoforms
EPS15	1.01	-0.24	AR-FL	APOL4	-0.81	-1.55	Both Isoforms
RUNX1	3.32	-0.23	AR-FL	NEB	-2.90	-1.55	Both Isoforms
COL4A3BP	1.24	-0.23	AR-FL	UGT2B7	-3.56	-1.54	Both Isoforms
ELOVL2	3.87	-0.23	AR-FL	FAM171B	-2.37	-1.53	Both Isoforms
ARFIP1	0.65	-0.23	AR-FL	TTLL2	-2.76	-1.53	Both Isoforms
RCBTB1	0.75	-0.23	AR-FL	ZNF624	-1.07	-1.53	Both Isoforms
ATP10A	1.10	-0.22	AR-FL	SYNPO	-2.99	-1.53	Both Isoforms
TDRD1	1.10	-0.22	AR-FL	SSTR1	-1.85	-1.53	Both Isoforms
PSD3	1.17	-0.21	AR-FL	SYT4	-0.60	-1.52	Both Isoforms
INTS10	1.21	-0.21	AR-FL	GTF2IRD2B	-1.42	-1.51	Both Isoforms
ABHD17B	0.73	-0.21	AR-FL	EPB41L4A	-2.96	-1.51	Both Isoforms
ATP8B1	0.66	-0.21	AR-FL	COL8A2	-2.54	-1.51	Both Isoforms
NHSL2	0.68	-0.21	AR-FL	PTGER2	-3.02	-1.51	Both Isoforms
LRRC16A	1.88	-0.20	AR-FL	MDGA1	-2.15	-1.51	Both Isoforms
TRPM2	0.73	-0.20	AR-FL	SORL1	-1.61	-1.51	Both Isoforms
ALDH4A1	0.90	-0.19	AR-FL	TMEM176B	-2.86	-1.50	Both Isoforms
GRHL1	0.64	-0.19	AR-FL	CDH26	-1.81	-1.50	Both Isoforms
PLXNB3	0.63	-0.19	AR-FL	HMGCS2	-0.77	-1.50	Both Isoforms
CHST11	0.66	-0.18	AR-FL	ZNF536	-3.28	-1.49	Both Isoforms
GPRC5B	1.00	-0.17	AR-FL	ATP8B4	-0.80	-1.48	Both Isoforms
SOAT1	1.44	-0.17	AR-FL	ADCY2	-0.96	-1.48	Both Isoforms
SGK1	3.25	-0.17	AR-FL	PLAGL1	-0.66	-1.48	Both Isoforms
CARTPT	0.82	-0.16	AR-FL	TRIB1	-3.04	-1.47	Both Isoforms
NUP58	0.76	-0.16	AR-FL	SNX10	-2.12	-1.47	Both Isoforms
CITED1	1.19	-0.16	AR-FL	CD200	-5.55	-1.47	Both Isoforms
ENDOD1	1.85	-0.16	AR-FL	UMODL1	-0.81	-1.47	Both Isoforms
PCDH1	1.62	-0.16	AR-FL	MAPK4	-0.92	-1.46	Both Isoforms
OAZ3	0.58	-0.16	AR-FL	FTCD	-2.02	-1.46	Both Isoforms
COPRS	0.61	-0.16	AR-FL	PAX1	-2.32	-1.46	Both Isoforms
GNAQ	1.03	-0.15	AR-FL	NOV	-4.29	-1.46	Both Isoforms
ALPK3	1.33	-0.15	AR-FL	TAL2	-2.16	-1.46	Both Isoforms

L3MBTL3	0.85	-0.15	AR-FL	CDH17	-2.72	-1.45	Both Isoforms
RBM45	0.58	-0.14	AR-FL	SYT2	-1.62	-1.45	Both Isoforms
PASK	0.78	-0.14	AR-FL	LOXL4	-3.20	-1.45	Both Isoforms
ACTR3	0.84	-0.14	AR-FL	ADAMTSL2	-1.89	-1.45	Both Isoforms
ANTXR2	0.58	-0.14	AR-FL	PITPNC1	-1.84	-1.45	Both Isoforms
SOD3	0.71	-0.13	AR-FL	ST7	-2.52	-1.44	Both Isoforms
BHLHA15	2.05	-0.12	AR-FL	BMX	-4.20	-1.44	Both Isoforms
PITHD1	0.62	-0.12	AR-FL	A1CF	-2.53	-1.44	Both Isoforms
AP2S1	0.68	-0.12	AR-FL	P2RX7	-3.70	-1.44	Both Isoforms
CARD10	0.63	-0.11	AR-FL	HGF	-3.77	-1.44	Both Isoforms
AF131216.1	1.31	-0.11	AR-FL	RGS17	-1.28	-1.43	Both Isoforms
OR5B2	5.20	-0.11	AR-FL	PGR	-2.65	-1.43	Both Isoforms
RHPN2	0.62	-0.10	AR-FL	FGL2	-3.67	-1.43	Both Isoforms
HIST2H4B	0.60	-0.10	AR-FL	PLEKHG7	-2.95	-1.42	Both Isoforms
SEC63	0.63	-0.09	AR-FL	PHEX	-1.54	-1.42	Both Isoforms
OTUD7B	1.43	-0.09	AR-FL	CDH10	-3.80	-1.42	Both Isoforms
C2orf72	0.83	-0.09	AR-FL	KCNB1	-2.39	-1.42	Both Isoforms
C6orf132	0.72	-0.09	AR-FL	ZYG11A	-1.33	-1.42	Both Isoforms
MTFP1	1.22	-0.08	AR-FL	TCP11L2	-1.64	-1.42	Both Isoforms
ZDHHC21	0.59	-0.08	AR-FL	LRRC9	-1.30	-1.41	Both Isoforms
SPATC1L	0.76	-0.07	AR-FL	PIK3IP1	-2.33	-1.41	Both Isoforms
BBS4	0.95	-0.07	AR-FL	SPTLC3	-1.92	-1.41	Both Isoforms
UHRF2	0.63	-0.07	AR-FL	CLDN11	-3.29	-1.40	Both Isoforms
UAP1	2.12	-0.07	AR-FL	BMF	-0.61	-1.40	Both Isoforms
PRKAR2A	0.58	-0.06	AR-FL	IGF2BP2	-0.77	-1.40	Both Isoforms
GSR	1.04	-0.06	AR-FL	LPAR1	-0.98	-1.40	Both Isoforms
ATP6V1C1	0.61	-0.06	AR-FL	CD8A	-2.68	-1.39	Both Isoforms
NUP98	0.67	-0.06	AR-FL	PKNOX2	-2.37	-1.39	Both Isoforms
WWC2	1.09	-0.06	AR-FL	GLI3	-2.34	-1.39	Both Isoforms
TEP1	0.67	-0.06	AR-FL	SLC13A3	-1.77	-1.39	Both Isoforms
CD9	0.77	-0.05	AR-FL	BCAT1	-2.91	-1.39	Both Isoforms
SEMA4A	0.60	-0.05	AR-FL	ZIC2	-3.78	-1.39	Both Isoforms
SNX16	0.99	-0.05	AR-FL	CAPRIN2	-1.86	-1.39	Both Isoforms
SPATA24	0.65	-0.05	AR-FL	NR1D2	-1.79	-1.39	Both Isoforms
WWP1	0.71	-0.05	AR-FL	C5	-2.42	-1.38	Both Isoforms
RUSC1	0.67	-0.05	AR-FL	PDE1B	-2.44	-1.38	Both Isoforms
TECPR1	1.14	-0.05	AR-FL	ANKRD66	-2.49	-1.38	Both Isoforms
PCMT1	0.76	-0.04	AR-FL	KLF7	-2.09	-1.37	Both Isoforms
IL1R1	0.85	-0.04	AR-FL	FAM89A	-1.63	-1.37	Both Isoforms
CSK	0.61	-0.04	AR-FL	PLPPR4	-2.25	-1.37	Both Isoforms
EPS8L1	1.78	-0.04	AR-FL	RHCE	-1.29	-1.37	Both Isoforms
SERINC2	1.07	-0.04	AR-FL	FZD7	-1.86	-1.36	Both Isoforms
UBE2D1	0.66	-0.04	AR-FL	CLIC5	-3.07	-1.36	Both Isoforms
UNC5C	0.59	-0.03	AR-FL	KLHL24	-1.35	-1.36	Both Isoforms
GSPT1	0.63	-0.03	AR-FL	CDON	-1.26	-1.36	Both Isoforms
ZNF615	0.76	-0.03	AR-FL	TIAM2	-1.35	-1.36	Both Isoforms

NFKBIE	0.58	-0.02	AR-FL	SKIDA1	-0.91	-1.36	Both Isoforms
SLAIN2	1.00	-0.02	AR-FL	TMEM266	-2.54	-1.36	Both Isoforms
TADA3	0.61	-0.02	AR-FL	KIAA1211	-0.86	-1.36	Both Isoforms
IPPK	0.64	-0.02	AR-FL	LAMB4	-2.28	-1.35	Both Isoforms
RAB6C	0.68	-0.01	AR-FL	C20orf195	-2.15	-1.35	Both Isoforms
MAZ	0.63	-0.01	AR-FL	NR3C1	-2.04	-1.35	Both Isoforms
HEBP2	1.24	-0.01	AR-FL	OPTN	-1.99	-1.35	Both Isoforms
SPATS2L	0.66	-0.01	AR-FL	SYN2	-2.46	-1.34	Both Isoforms
TIMM23	0.64	-0.01	AR-FL	EVX1	-2.90	-1.34	Both Isoforms
KIAA0355	0.86	-0.01	AR-FL	NATD1	-2.14	-1.34	Both Isoforms
EML1	1.25	0.00	AR-FL	ADH1B	-4.18	-1.34	Both Isoforms
RYBP	0.65	0.00	AR-FL	MEP1A	-7.48	-1.34	Both Isoforms
KCNG3	1.74	0.00	AR-FL	KCTD16	-2.84	-1.34	Both Isoforms
POLR3E	0.91	0.00	AR-FL	CDC14A	-2.81	-1.33	Both Isoforms
NRG3	-2.61	-6.90	Both Isoforms	SLC22A11	-0.98	-1.33	Both Isoforms
MBNL2	-0.91	-6.23	Both Isoforms	AKR1C1	-1.85	-1.33	Both Isoforms
LRRN1	-3.48	-6.20	Both Isoforms	SH3GL3	-2.90	-1.33	Both Isoforms
DAPK1	-5.53	-6.00	Both Isoforms	MMP10	-1.82	-1.32	Both Isoforms
CSRNP3	-1.81	-5.40	Both Isoforms	GPC5	-1.47	-1.32	Both Isoforms
VARS2	-3.00	-4.88	Both Isoforms	SH3BP5	-1.40	-1.32	Both Isoforms
EYA2	-1.05	-4.71	Both Isoforms	MR1	-1.68	-1.32	Both Isoforms
PRSS1	-1.14	-4.70	Both Isoforms	CCDC158	-2.68	-1.32	Both Isoforms
FRAS1	-2.26	-4.47	Both Isoforms	POU5F1B	-1.33	-1.31	Both Isoforms
GRIK1	-2.10	-4.46	Both Isoforms	LRRTM3	-6.20	-1.31	Both Isoforms
OR51E1	-3.00	-4.40	Both Isoforms	CDK18	-2.82	-1.31	Both Isoforms
ST6GALNAC5	-2.74	-4.39	Both Isoforms	CD44	-0.66	-1.31	Both Isoforms
SALL4	-4.77	-4.21	Both Isoforms	LRRC7	-2.17	-1.31	Both Isoforms
ADAM2	-1.60	-4.19	Both Isoforms	TSPAN8	-0.97	-1.31	Both Isoforms
IHH	-3.51	-4.13	Both Isoforms	CCDC67	-1.78	-1.31	Both Isoforms
FAM189A1	-1.73	-3.92	Both Isoforms	CDH3	-2.23	-1.31	Both Isoforms
ABCA12	-2.88	-3.78	Both Isoforms	TMEM159	-1.64	-1.31	Both Isoforms

ARHGDIG	-0.59	-3.76	Both Isoforms	AMN	-3.16	-1.31	Both Isoforms
RLN2	-1.12	-3.67	Both Isoforms	ADGRD1	-2.63	-1.30	Both Isoforms
NAV3	-1.94	-3.65	Both Isoforms	SYT13	-1.20	-1.30	Both Isoforms
PLA2G2F	-5.19	-3.61	Both Isoforms	LANCL3	-1.85	-1.30	Both Isoforms
COL3A1	-3.39	-3.61	Both Isoforms	PPP1R14C	-3.42	-1.30	Both Isoforms
COL5A2	-2.88	-3.60	Both Isoforms	PPP2R5A	-1.75	-1.30	Both Isoforms
LTF	-2.49	-3.50	Both Isoforms	ADGRG6	-3.90	-1.30	Both Isoforms
OR51E2	-0.82	-3.49	Both Isoforms	FAM184A	-2.87	-1.30	Both Isoforms
OPRK1	-4.43	-3.45	Both Isoforms	GATA5	-1.46	-1.30	Both Isoforms
SLC6A20	-5.80	-3.41	Both Isoforms	ACKR3	-2.01	-1.30	Both Isoforms
ACPP	-0.61	-3.37	Both Isoforms	TMEM200A	-5.49	-1.30	Both Isoforms
CCDC83	-4.81	-3.17	Both Isoforms	LTBP1	-2.95	-1.30	Both Isoforms
SLC5A1	-1.88	-3.15	Both Isoforms	CDH12	-1.93	-1.30	Both Isoforms
CERK	-2.81	-3.09	Both Isoforms	KIF26B	-1.15	-1.30	Both Isoforms
DLX3	-3.16	-3.02	Both Isoforms	AFP	-0.82	-1.30	Both Isoforms
AMIGO2	-5.06	-2.97	Both Isoforms	SLC4A10	-3.15	-1.29	Both Isoforms
SCN8A	-1.15	-2.96	Both Isoforms	P4HA3	-1.87	-1.29	Both Isoforms
SIRPA	-0.92	-2.95	Both Isoforms	PTGFR	-0.92	-1.29	Both Isoforms
GALNT14	-2.50	-2.93	Both Isoforms	CNIH3	-0.71	-1.29	Both Isoforms
ODAM	-1.56	-2.86	Both Isoforms	RSAD2	-1.59	-1.29	Both Isoforms
TLL1	-2.79	-2.86	Both Isoforms	NCAM2	-3.22	-1.29	Both Isoforms
IL1RN	-3.54	-2.85	Both Isoforms	SLC3A1	-2.85	-1.29	Both Isoforms
CXorf36	-5.63	-2.84	Both Isoforms	BNC1	-2.49	-1.29	Both Isoforms
CD274	-1.50	-2.83	Both Isoforms	PLCXD3	-2.58	-1.29	Both Isoforms
BCL11B	-1.20	-2.80	Both Isoforms	CSMD2	-1.08	-1.28	Both Isoforms

KIFC1	-2.52	-2.77	Both Isoforms	CHST11	-1.31	-1.28	Both Isoforms
VAV3	-3.01	-2.76	Both Isoforms	KLHDC8A	-5.81	-1.28	Both Isoforms
ACSS1	-1.46	-2.74	Both Isoforms	FRAS1	-2.51	-1.28	Both Isoforms
ELF5	-0.60	-2.70	Both Isoforms	GATA6	-0.89	-1.28	Both Isoforms
PHF1	-2.00	-2.69	Both Isoforms	SLC10A5	-1.72	-1.28	Both Isoforms
MYRIP	-1.82	-2.67	Both Isoforms	MUC13	-0.95	-1.28	Both Isoforms
PLD1	-4.56	-2.66	Both Isoforms	BLNK	-1.02	-1.27	Both Isoforms
TMC7	-0.85	-2.64	Both Isoforms	TMEM45B	-1.19	-1.27	Both Isoforms
RIMS4	-2.13	-2.64	Both Isoforms	IL16	-0.67	-1.27	Both Isoforms
TSHZ3	-1.90	-2.63	Both Isoforms	ACSL5	-0.80	-1.27	Both Isoforms
GBP3	-0.65	-2.63	Both Isoforms	KCNK13	-1.81	-1.27	Both Isoforms
LRRC31	-4.53	-2.63	Both Isoforms	PRTG	-1.86	-1.27	Both Isoforms
LYPD6B	-2.40	-2.63	Both Isoforms	CSRNP3	-2.11	-1.27	Both Isoforms
DPYSL3	-0.82	-2.61	Both Isoforms	ANO2	-1.93	-1.27	Both Isoforms
SUSD4	-2.01	-2.60	Both Isoforms	SLC9A9	-1.15	-1.27	Both Isoforms
CAMK2N1	-4.21	-2.60	Both Isoforms	CDH7	-1.06	-1.27	Both Isoforms
TMEM179	-1.15	-2.58	Both Isoforms	CECR2	-1.69	-1.27	Both Isoforms
EIF3CL	-1.69	-2.57	Both Isoforms	FAM175A	-2.74	-1.26	Both Isoforms
CACHD1	-2.36	-2.54	Both Isoforms	SOX5	-2.17	-1.26	Both Isoforms
LRRC4C	-2.41	-2.51	Both Isoforms	MYB	-2.32	-1.26	Both Isoforms
VWF	-1.03	-2.51	Both Isoforms	PLA2G2A	-3.26	-1.26	Both Isoforms
PCDH11Y	-3.43	-2.50	Both Isoforms	TRERF1	-1.81	-1.26	Both Isoforms
AIDA	-1.59	-2.49	Both Isoforms	ZNF385B	-2.18	-1.26	Both Isoforms
ABCG1	-0.98	-2.48	Both Isoforms	CEMIP	-1.57	-1.26	Both Isoforms
PLA2G2A	-3.71	-2.48	Both Isoforms	BRINP1	-1.38	-1.26	Both Isoforms

EFHC2	-1.95	-2.48	Both Isoforms	AKAP3	-2.66	-1.25	Both Isoforms
RELN	-1.13	-2.43	Both Isoforms	TBX6	-1.90	-1.25	Both Isoforms
FN1	-0.92	-2.42	Both Isoforms	ADAMTSL3	-2.98	-1.25	Both Isoforms
IL36RN	-1.17	-2.40	Both Isoforms	MID1	-1.73	-1.25	Both Isoforms
MYB	-2.65	-2.39	Both Isoforms	MEF2C	-2.60	-1.25	Both Isoforms
DDC	-2.20	-2.39	Both Isoforms	HEPACAM2	-1.25	-1.25	Both Isoforms
PCED1B	-3.99	-2.37	Both Isoforms	CMBL	-2.45	-1.25	Both Isoforms
LYPD6	-1.06	-2.36	Both Isoforms	CDK5R1	-1.17	-1.25	Both Isoforms
TTC9	-0.84	-2.35	Both Isoforms	GPR146	-0.81	-1.25	Both Isoforms
MMP16	-1.83	-2.33	Both Isoforms	SCGB1D2	-1.67	-1.25	Both Isoforms
SLC7A5	-2.61	-2.31	Both Isoforms	PPARG	-1.18	-1.25	Both Isoforms
DCBLD2	-1.20	-2.30	Both Isoforms	ITGA9	-3.76	-1.25	Both Isoforms
LRRC8C	-1.08	-2.30	Both Isoforms	ALX4	-2.96	-1.24	Both Isoforms
REEP1	-3.16	-2.29	Both Isoforms	DAPK1	-1.61	-1.24	Both Isoforms
GLP1R	-1.14	-2.27	Both Isoforms	CD83	-2.19	-1.24	Both Isoforms
CDH3	-2.67	-2.23	Both Isoforms	FCGBP	-0.61	-1.23	Both Isoforms
RTN4RL1	-1.83	-2.23	Both Isoforms	SLC6A4	-1.58	-1.23	Both Isoforms
FFAR2	-3.17	-2.19	Both Isoforms	SLC30A4	-1.12	-1.23	Both Isoforms
CAMK2B	-1.43	-2.19	Both Isoforms	KIAA0922	-1.98	-1.23	Both Isoforms
MAPK4	-1.34	-2.19	Both Isoforms	SUPT3H	-1.86	-1.23	Both Isoforms
KCNK5	-1.37	-2.14	Both Isoforms	ALB	-3.52	-1.23	Both Isoforms
KIAA1211L	-1.43	-2.12	Both Isoforms	CD109	-2.35	-1.23	Both Isoforms
SMCO3	-1.14	-2.11	Both Isoforms	DUSP5	-1.01	-1.23	Both Isoforms
HOXB9	-0.67	-2.10	Both Isoforms	NOVA1	-2.86	-1.22	Both Isoforms
ETNPPL	-1.97	-2.09	Both Isoforms	HRH1	-1.05	-1.22	Both Isoforms

PLCH1	-2.11	-2.09	Both Isoforms	SRGAP3	-1.15	-1.22	Both Isoforms
P2RX7	-1.06	-2.09	Both Isoforms	FAM101A	-1.57	-1.22	Both Isoforms
PTPRK	-1.14	-2.08	Both Isoforms	WNK4	-2.89	-1.22	Both Isoforms
MATN2	-4.39	-2.06	Both Isoforms	CCT6B	-1.31	-1.22	Both Isoforms
TULP4	-1.58	-2.04	Both Isoforms	GBGT1	-1.85	-1.22	Both Isoforms
IGFBP3	-0.61	-2.03	Both Isoforms	SCN4B	-2.34	-1.21	Both Isoforms
FLT4	-2.10	-2.03	Both Isoforms	NDUFA4L2	-1.69	-1.21	Both Isoforms
MANEA	-2.05	-2.01	Both Isoforms	POU6F1	-2.14	-1.21	Both Isoforms
TOX3	-0.98	-2.01	Both Isoforms	FAM47E	-1.76	-1.21	Both Isoforms
C1orf95	-2.12	-1.99	Both Isoforms	NPNT	-2.10	-1.21	Both Isoforms
OLFM2	-1.01	-1.98	Both Isoforms	C4orf32	-0.93	-1.21	Both Isoforms
CDK8	-1.78	-1.97	Both Isoforms	CAPN13	-2.41	-1.21	Both Isoforms
CUTA	-2.15	-1.97	Both Isoforms	NFATC4	-1.45	-1.21	Both Isoforms
TMOD1	-1.12	-1.96	Both Isoforms	IER5	-2.50	-1.21	Both Isoforms
NEB	-2.03	-1.96	Both Isoforms	SALL2	-2.04	-1.21	Both Isoforms
LRRC7	-1.94	-1.96	Both Isoforms	LRRN1	-3.26	-1.21	Both Isoforms
SCD5	-0.73	-1.96	Both Isoforms	ACRBP	-2.96	-1.21	Both Isoforms
TGM6	-1.41	-1.94	Both Isoforms	APLF	-2.38	-1.20	Both Isoforms
LGI2	-2.20	-1.93	Both Isoforms	RP11-166B2.1	-2.01	-1.20	Both Isoforms
DNAJC6	-0.71	-1.87	Both Isoforms	CHRNA7	-1.72	-1.20	Both Isoforms
FAM198A	-2.21	-1.87	Both Isoforms	PCOLCE2	-2.21	-1.20	Both Isoforms
BARD1	-2.09	-1.86	Both Isoforms	GNG2	-2.59	-1.20	Both Isoforms
ABHD1	-0.72	-1.86	Both Isoforms	MFSD4	-2.20	-1.20	Both Isoforms
TARSL2	-1.12	-1.85	Both Isoforms	FYN	-3.05	-1.19	Both Isoforms
ZSWIM5	-1.60	-1.85	Both Isoforms	POLR2M	-2.20	-1.19	Both Isoforms

TMEM163	-1.76	-1.84	Both Isoforms	EIF3C	-1.25	-1.19	Both Isoforms
POLR3G	-1.51	-1.84	Both Isoforms	KCNJ3	-3.59	-1.19	Both Isoforms
TGFA	-1.42	-1.84	Both Isoforms	GAB1	-0.98	-1.19	Both Isoforms
ANTXR1	-3.16	-1.83	Both Isoforms	VILL	-1.18	-1.19	Both Isoforms
LUZP2	-1.43	-1.83	Both Isoforms	SLC7A5	-1.76	-1.19	Both Isoforms
AKR1C2	-0.86	-1.83	Both Isoforms	ANXA10	-1.21	-1.19	Both Isoforms
CMPK2	-1.24	-1.82	Both Isoforms	SLC24A2	-1.88	-1.19	Both Isoforms
SESN1	-0.92	-1.81	Both Isoforms	STK32A	-2.76	-1.18	Both Isoforms
PCDH11X	-4.12	-1.80	Both Isoforms	SPRY1	-1.15	-1.18	Both Isoforms
BCL2	-1.14	-1.80	Both Isoforms	TMEM220	-1.19	-1.18	Both Isoforms
ARHGAP22	-1.75	-1.79	Both Isoforms	GLS2	-1.51	-1.18	Both Isoforms
CA12	-0.88	-1.79	Both Isoforms	ZSCAN31	-0.78	-1.18	Both Isoforms
SLIT1	-2.08	-1.79	Both Isoforms	CMPK2	-1.12	-1.18	Both Isoforms
DEPTOR	-1.38	-1.78	Both Isoforms	TRPC1	-1.66	-1.18	Both Isoforms
HTRA1	-1.85	-1.77	Both Isoforms	RBFOX3	-3.25	-1.18	Both Isoforms
IL27RA	-3.53	-1.77	Both Isoforms	FOXN3	-0.82	-1.18	Both Isoforms
ADCY1	-1.74	-1.76	Both Isoforms	PRKD1	-1.39	-1.18	Both Isoforms
CHAC1	-1.40	-1.76	Both Isoforms	YPEL1	-1.61	-1.17	Both Isoforms
FAM184B	-1.27	-1.76	Both Isoforms	TSHZ1	-1.75	-1.17	Both Isoforms
GPR63	-1.72	-1.75	Both Isoforms	DPY19L2	-2.70	-1.17	Both Isoforms
SLITRK3	-3.64	-1.74	Both Isoforms	FGL1	-0.74	-1.17	Both Isoforms
PAK3	-0.67	-1.73	Both Isoforms	COL16A1	-2.67	-1.17	Both Isoforms
BEST1	-2.20	-1.73	Both Isoforms	TNNT1	-1.25	-1.17	Both Isoforms
ARHGAP9	-1.10	-1.73	Both Isoforms	FRRS1L	-2.78	-1.17	Both Isoforms
C8orf34	-0.59	-1.72	Both Isoforms	NELL2	-1.27	-1.17	Both Isoforms

JAKMIP1	-1.24	-1.72	Both Isoforms	TMPRSS4	-0.59	-1.17	Both Isoforms
CHRD	-1.46	-1.71	Both Isoforms	GDA	-0.58	-1.17	Both Isoforms
NOV	-3.78	-1.71	Both Isoforms	NIPAL2	-0.67	-1.16	Both Isoforms
FOXO6	-1.89	-1.71	Both Isoforms	FAM189A1	-0.72	-1.16	Both Isoforms
SLC6A9	-0.87	-1.71	Both Isoforms	CHST15	-1.46	-1.16	Both Isoforms
TMEM158	-1.29	-1.69	Both Isoforms	BEND7	-1.95	-1.16	Both Isoforms
ABCB11	-9.44	-1.69	Both Isoforms	IZUMO1R	-2.72	-1.16	Both Isoforms
ENO2	-1.65	-1.68	Both Isoforms	ZMYND15	-2.11	-1.16	Both Isoforms
CERS1	-1.41	-1.68	Both Isoforms	LTA4H	-1.86	-1.16	Both Isoforms
AIM1L	-1.76	-1.68	Both Isoforms	HHAT	-1.02	-1.16	Both Isoforms
CD200	-4.86	-1.68	Both Isoforms	INSM2	-2.47	-1.16	Both Isoforms
UBE2L6	-1.29	-1.68	Both Isoforms	N4BP2L1	-2.19	-1.15	Both Isoforms
CCDC80	-3.79	-1.67	Both Isoforms	NSG1	-2.75	-1.15	Both Isoforms
ARL15	-0.80	-1.66	Both Isoforms	HNF4G	-2.28	-1.15	Both Isoforms
FRMD3	-1.29	-1.66	Both Isoforms	RASSF10	-2.47	-1.15	Both Isoforms
GPR27	-0.74	-1.66	Both Isoforms	NEURL3	-2.68	-1.15	Both Isoforms
SCARA3	-0.93	-1.65	Both Isoforms	PPM1N	-1.96	-1.15	Both Isoforms
TMEM173	-0.66	-1.65	Both Isoforms	RARRES2	-2.42	-1.15	Both Isoforms
SQRDL	-0.65	-1.64	Both Isoforms	FZD3	-1.15	-1.15	Both Isoforms
CSTA	-1.08	-1.64	Both Isoforms	HLX	-2.08	-1.15	Both Isoforms
NINL	-2.33	-1.63	Both Isoforms	CX3CL1	-3.34	-1.15	Both Isoforms
FRMPD1	-0.92	-1.62	Both Isoforms	SEMA6C	-2.20	-1.14	Both Isoforms
HOXC4	-1.24	-1.61	Both Isoforms	PLSCR4	-2.10	-1.14	Both Isoforms
UNC5B	-0.76	-1.59	Both Isoforms	IYD	-1.36	-1.14	Both Isoforms
RGPD5	-1.58	-1.59	Both Isoforms	IL17RD	-2.55	-1.14	Both Isoforms

PDZD4	-0.76	-1.59	Both Isoforms	NOL4	-2.11	-1.14	Both Isoforms
MAGED4	-1.66	-1.58	Both Isoforms	GPD1L	-0.64	-1.14	Both Isoforms
SH3PXD2A	-0.92	-1.58	Both Isoforms	GDF9	-1.30	-1.14	Both Isoforms
SIDT1	-1.61	-1.56	Both Isoforms	ASAH2	-1.19	-1.14	Both Isoforms
NAALAD2	-1.44	-1.55	Both Isoforms	LRRC4C	-2.53	-1.14	Both Isoforms
PKNOX2	-1.29	-1.54	Both Isoforms	LPCAT4	-2.42	-1.13	Both Isoforms
PARK2	-1.77	-1.54	Both Isoforms	TMEM26	-0.82	-1.13	Both Isoforms
CSF1	-0.61	-1.54	Both Isoforms	PCGF5	-1.77	-1.13	Both Isoforms
TEX19	-0.96	-1.53	Both Isoforms	ITGB6	-0.85	-1.13	Both Isoforms
AUTS2	-1.09	-1.53	Both Isoforms	ITM2A	-1.91	-1.13	Both Isoforms
SOX8	-1.37	-1.52	Both Isoforms	RTN4RL1	-1.41	-1.13	Both Isoforms
PHGDH	-0.86	-1.52	Both Isoforms	FGGY	-1.45	-1.13	Both Isoforms
PCK2	-0.69	-1.52	Both Isoforms	CDX2	-1.03	-1.13	Both Isoforms
STON1	-0.94	-1.51	Both Isoforms	NET1	-0.79	-1.12	Both Isoforms
ACKR3	-2.80	-1.51	Both Isoforms	PBX1	-1.54	-1.12	Both Isoforms
PDX1	-0.83	-1.50	Both Isoforms	STBD1	-0.99	-1.12	Both Isoforms
ZNF84	-1.79	-1.50	Both Isoforms	IL6ST	-1.17	-1.12	Both Isoforms
REG4	-2.27	-1.49	Both Isoforms	ELFN1	-1.40	-1.12	Both Isoforms
TSPAN5	-1.42	-1.49	Both Isoforms	SHISA6	-0.75	-1.12	Both Isoforms
SVIP	-0.90	-1.49	Both Isoforms	PPM1L	-1.06	-1.12	Both Isoforms
FGF13	-1.62	-1.48	Both Isoforms	CCDC169	-1.82	-1.12	Both Isoforms
DIO1	-1.86	-1.48	Both Isoforms	CLEC2D	-1.19	-1.11	Both Isoforms
ZMAT1	-1.38	-1.48	Both Isoforms	KIF5A	-2.92	-1.11	Both Isoforms
FRMD6	-0.73	-1.47	Both Isoforms	MYZAP	-2.67	-1.11	Both Isoforms
OSR2	-1.92	-1.46	Both Isoforms	IGSF3	-0.65	-1.11	Both Isoforms

FAM171A2	-1.04	-1.46	Both Isoforms	CCNG2	-1.47	-1.11	Both Isoforms
ST3GAL1	-0.77	-1.46	Both Isoforms	FBLN2	-1.88	-1.11	Both Isoforms
KAZN	-1.12	-1.45	Both Isoforms	ENOX1	-1.31	-1.11	Both Isoforms
RNF165	-1.49	-1.44	Both Isoforms	PPM1J	-0.80	-1.11	Both Isoforms
PTPN13	-0.66	-1.44	Both Isoforms	PPP1R3C	-0.68	-1.11	Both Isoforms
KLHL1	-0.69	-1.44	Both Isoforms	HSD17B6	-1.95	-1.11	Both Isoforms
BAHCC1	-1.00	-1.43	Both Isoforms	ADAMTS3	-1.30	-1.11	Both Isoforms
PKIB	-2.81	-1.43	Both Isoforms	EFNB2	-0.82	-1.11	Both Isoforms
CPT1C	-0.87	-1.42	Both Isoforms	RASGEF1B	-1.21	-1.11	Both Isoforms
EMCN	-2.61	-1.42	Both Isoforms	SLC17A4	-2.52	-1.11	Both Isoforms
NRTN	-0.75	-1.42	Both Isoforms	ROR2	-2.51	-1.11	Both Isoforms
NANOS1	-1.38	-1.42	Both Isoforms	C1QTNF6	-1.21	-1.10	Both Isoforms
NLRC5	-1.96	-1.41	Both Isoforms	HOXD8	-0.98	-1.10	Both Isoforms
FAM20C	-1.80	-1.41	Both Isoforms	ENPP5	-2.13	-1.10	Both Isoforms
PRKAR1B	-1.44	-1.41	Both Isoforms	NOX1	-2.15	-1.10	Both Isoforms
ACTRT3	-2.63	-1.41	Both Isoforms	NRXN1	-3.47	-1.10	Both Isoforms
INHBB	-1.66	-1.41	Both Isoforms	HOXB6	-1.23	-1.10	Both Isoforms
SOWAHB	-1.20	-1.41	Both Isoforms	MTMR11	-0.79	-1.10	Both Isoforms
COLEC12	-2.61	-1.40	Both Isoforms	PPP2R2B	-2.72	-1.10	Both Isoforms
UBA7	-0.60	-1.39	Both Isoforms	ZNF334	-1.90	-1.09	Both Isoforms
SOBP	-1.55	-1.39	Both Isoforms	ARHGAP9	-2.20	-1.09	Both Isoforms
KSR2	-1.41	-1.39	Both Isoforms	EPHB6	-0.79	-1.09	Both Isoforms
IKZF3	-1.08	-1.38	Both Isoforms	GPR37	-0.85	-1.09	Both Isoforms
ATP2C2	-0.64	-1.37	Both Isoforms	VIL1	-2.52	-1.09	Both Isoforms
EML6	-2.60	-1.37	Both Isoforms	AKR7A3	-1.55	-1.09	Both Isoforms

HAP1	-1.72	-1.37	Both Isoforms	PADI2	-1.73	-1.09	Both Isoforms
MT1G	-1.20	-1.36	Both Isoforms	HNMT	-1.25	-1.09	Both Isoforms
SRCIN1	-3.02	-1.36	Both Isoforms	FHOD3	-1.02	-1.09	Both Isoforms
CAPN5	-0.88	-1.36	Both Isoforms	ZNF491	-0.79	-1.09	Both Isoforms
SVIL	-1.47	-1.36	Both Isoforms	KCNIP2	-2.73	-1.09	Both Isoforms
C1QTNF3	-1.27	-1.35	Both Isoforms	ZNF699	-0.81	-1.08	Both Isoforms
AIF1L	-0.85	-1.35	Both Isoforms	ZNF385C	-2.78	-1.08	Both Isoforms
TMEM51	-1.00	-1.34	Both Isoforms	SELENBP1	-1.89	-1.08	Both Isoforms
RAI14	-1.28	-1.34	Both Isoforms	AKAP2	-2.30	-1.08	Both Isoforms
PSPH	-0.62	-1.32	Both Isoforms	GFI1	-1.75	-1.08	Both Isoforms
TMEM181	-0.59	-1.32	Both Isoforms	HTRA1	-2.74	-1.08	Both Isoforms
KNDC1	-0.66	-1.32	Both Isoforms	COL17A1	-1.59	-1.08	Both Isoforms
PARD3B	-0.92	-1.31	Both Isoforms	FOXF2	-1.77	-1.08	Both Isoforms
CNTN2	-3.06	-1.31	Both Isoforms	FRMD6	-1.36	-1.07	Both Isoforms
PACRG	-1.46	-1.31	Both Isoforms	NID1	-1.56	-1.07	Both Isoforms
OAS3	-0.77	-1.31	Both Isoforms	SCIN	-0.82	-1.07	Both Isoforms
SH3KBP1	-0.80	-1.30	Both Isoforms	PTH1R	-5.08	-1.07	Both Isoforms
SLC16A7	-1.01	-1.30	Both Isoforms	KIRREL	-3.78	-1.07	Both Isoforms
SLCO5A1	-1.13	-1.30	Both Isoforms	TNFRSF11A	-0.80	-1.07	Both Isoforms
ASNS	-0.73	-1.29	Both Isoforms	AIM1L	-1.62	-1.07	Both Isoforms
LHFPL2	-0.74	-1.28	Both Isoforms	ROBO3	-0.68	-1.07	Both Isoforms
ARNT2	-0.74	-1.27	Both Isoforms	NEU4	-3.34	-1.07	Both Isoforms
ALOXE3	-0.75	-1.27	Both Isoforms	SEMA6A	-0.99	-1.06	Both Isoforms
SAMD11	-1.05	-1.27	Both Isoforms	NEFL	-2.00	-1.06	Both Isoforms
SLC30A10	-2.40	-1.27	Both Isoforms	GCOM1	-4.05	-1.06	Both Isoforms

TUBA4A	-0.89	-1.27	Both Isoforms	ADGRL2	-1.07	-1.06	Both Isoforms
PSAT1	-1.04	-1.26	Both Isoforms	MXI1	-3.07	-1.06	Both Isoforms
PLGRKT	-0.64	-1.25	Both Isoforms	ITGA2	-1.33	-1.06	Both Isoforms
TINAGL1	-1.90	-1.25	Both Isoforms	TMEM158	-0.95	-1.06	Both Isoforms
MARK1	-1.48	-1.24	Both Isoforms	2-Mar	-0.94	-1.06	Both Isoforms
SRGAP3	-1.25	-1.24	Both Isoforms	LASP1	-1.72	-1.06	Both Isoforms
ZYG11A	-1.63	-1.24	Both Isoforms	ERBB3	-0.59	-1.06	Both Isoforms
STK3	-0.83	-1.24	Both Isoforms	SSPN	-2.05	-1.06	Both Isoforms
MAPKAPK3	-1.11	-1.22	Both Isoforms	NLGN1	-2.70	-1.05	Both Isoforms
LRRC34	-2.37	-1.22	Both Isoforms	OXTR	-2.02	-1.05	Both Isoforms
ANO9	-0.87	-1.22	Both Isoforms	CEP68	-2.85	-1.05	Both Isoforms
PTRF	-1.09	-1.22	Both Isoforms	RAP2B	-2.58	-1.05	Both Isoforms
CWH43	-0.76	-1.21	Both Isoforms	XYLT1	-0.76	-1.05	Both Isoforms
CLDND2	-1.07	-1.21	Both Isoforms	DSC3	-0.64	-1.05	Both Isoforms
APBA1	-0.70	-1.21	Both Isoforms	CABYR	-1.54	-1.05	Both Isoforms
SLC29A1	-1.22	-1.21	Both Isoforms	HOXC13	-0.86	-1.05	Both Isoforms
FRMPD3	-0.94	-1.20	Both Isoforms	ADM	-1.44	-1.05	Both Isoforms
SLC9A3	-2.15	-1.20	Both Isoforms	BCO1	-1.43	-1.05	Both Isoforms
POU5F1B	-0.76	-1.20	Both Isoforms	SERPINB9	-1.97	-1.05	Both Isoforms
DMGDH	-0.97	-1.19	Both Isoforms	PARP8	-2.29	-1.05	Both Isoforms
SLC16A14	-1.25	-1.19	Both Isoforms	PCMTD1	-0.67	-1.04	Both Isoforms
NCOA7	-1.32	-1.18	Both Isoforms	SGTB	-1.38	-1.04	Both Isoforms
MYT1	-1.29	-1.18	Both Isoforms	MCTP1	-2.82	-1.04	Both Isoforms
ADAM11	-0.60	-1.18	Both Isoforms	FAM131B	-0.94	-1.04	Both Isoforms
EFNB3	-0.99	-1.16	Both Isoforms	KLHDC1	-1.22	-1.04	Both Isoforms

ZDHHC14		-0.68	-1.16	Both Isoforms	GJA3	-1.83	-1.04	Both Isoforms
ARHGAP27		-1.48	-1.16	Both Isoforms	TNFSF4	-0.94	-1.04	Both Isoforms
NEDD9		-1.02	-1.16	Both Isoforms	SLC12A6	-0.67	-1.04	Both Isoforms
PKP4		-1.24	-1.15	Both Isoforms	TSHZ3	-1.99	-1.04	Both Isoforms
AGBL3		-0.96	-1.15	Both Isoforms	PAPSS1	-0.81	-1.04	Both Isoforms
MYNN		-1.58	-1.15	Both Isoforms	ATP8A1	-1.46	-1.04	Both Isoforms
EXPH5		-0.86	-1.14	Both Isoforms	DLX3	-1.01	-1.04	Both Isoforms
SLC16A10		-0.78	-1.14	Both Isoforms	SI	-4.20	-1.03	Both Isoforms
CPNE3		-0.81	-1.14	Both Isoforms	MTURN	-2.33	-1.03	Both Isoforms
ASIC1		-1.09	-1.14	Both Isoforms	CCDC122	-1.38	-1.03	Both Isoforms
	3-Mar	-0.75	-1.14	Both Isoforms	ACOXL	-1.33	-1.03	Both Isoforms
C16orf45		-2.28	-1.14	Both Isoforms	HS3ST3A1	-3.07	-1.03	Both Isoforms
MAPT		-1.34	-1.12	Both Isoforms	TMEM27	-1.16	-1.03	Both Isoforms
GPR160		-0.90	-1.12	Both Isoforms	HSPA12B	-1.26	-1.03	Both Isoforms
GSAP		-0.99	-1.12	Both Isoforms	SGF29	-1.37	-1.03	Both Isoforms
CABLES1		-0.81	-1.11	Both Isoforms	SPATA17	-1.39	-1.03	Both Isoforms
PCDHGA12		-0.67	-1.11	Both Isoforms	PLEKHA2	-1.19	-1.02	Both Isoforms
TBL1Y		-0.93	-1.11	Both Isoforms	BACH2	-2.17	-1.02	Both Isoforms
SLC44A1		-1.26	-1.11	Both Isoforms	HOXC12	-1.09	-1.02	Both Isoforms
C5orf30		-1.45	-1.11	Both Isoforms	RORB	-2.08	-1.02	Both Isoforms
LRRC16B		-0.98	-1.11	Both Isoforms	C11orf95	-1.36	-1.02	Both Isoforms
SRMS		-2.05	-1.11	Both Isoforms	CALHM2	-1.74	-1.02	Both Isoforms
C3orf70		-1.13	-1.10	Both Isoforms	COL28A1	-1.09	-1.02	Both Isoforms
NWD1		-1.74	-1.10	Both Isoforms	LIAS	-1.54	-1.02	Both Isoforms
	3-Sep	-0.85	-1.10	Both Isoforms	ESYT3	-1.40	-1.02	Both Isoforms

SYNGAP1	-0.66	-1.09	Both Isoforms	MIA3	-1.12	-1.01	Both Isoforms
DOCK3	-0.82	-1.09	Both Isoforms	LGR4	-1.06	-1.01	Both Isoforms
ZNF629	-0.97	-1.09	Both Isoforms	DDX25	-1.76	-1.01	Both Isoforms
PABPC5	-1.08	-1.09	Both Isoforms	C7orf31	-1.26	-1.01	Both Isoforms
SERPINI1	-2.09	-1.09	Both Isoforms	DBP	-2.17	-1.01	Both Isoforms
KCNAB2	-1.13	-1.09	Both Isoforms	FRY	-1.23	-1.01	Both Isoforms
B4GALNT1	-1.05	-1.08	Both Isoforms	YPEL2	-0.70	-1.01	Both Isoforms
NDUFAF4	-0.82	-1.07	Both Isoforms	C15orf48	-2.40	-1.01	Both Isoforms
ZNF367	-0.95	-1.07	Both Isoforms	NKX2-1	-0.83	-1.01	Both Isoforms
SLC2A9	-1.54	-1.06	Both Isoforms	CDH4	-1.80	-1.01	Both Isoforms
STMN3	-0.62	-1.06	Both Isoforms	AGO4	-1.11	-1.01	Both Isoforms
IL17D	-0.89	-1.06	Both Isoforms	RINL	-1.37	-1.01	Both Isoforms
SELENBP1	-1.74	-1.05	Both Isoforms	HFM1	-2.65	-1.01	Both Isoforms
TNNT2	-0.97	-1.05	Both Isoforms	DNAH5	-1.64	-1.01	Both Isoforms
ASAP2	-1.43	-1.05	Both Isoforms	FOXD3	-0.96	-1.00	Both Isoforms
MX1	-0.60	-1.04	Both Isoforms	TBX19	-0.58	-1.00	Both Isoforms
PLCD3	-0.84	-1.04	Both Isoforms	CA13	-0.95	-1.00	Both Isoforms
GPR143	-0.98	-1.03	Both Isoforms	BTN3A2	-2.19	-1.00	Both Isoforms
GABRA3	-0.88	-1.03	Both Isoforms	GSTO2	-2.46	-1.00	Both Isoforms
RALB	-0.83	-1.03	Both Isoforms	PAK1	-1.32	-1.00	Both Isoforms
CRYL1	-0.61	-1.03	Both Isoforms	SYT15	-1.50	-1.00	Both Isoforms
GLI3	-1.50	-1.03	Both Isoforms	SCN2A	-1.63	-1.00	Both Isoforms
EBF3	-1.07	-1.03	Both Isoforms	ELFN2	-1.32	-1.00	Both Isoforms
KIAA1211	-1.12	-1.02	Both Isoforms	SLC8A2	-1.61	-1.00	Both Isoforms
SYTL2	-2.27	-1.02	Both Isoforms	MX1	-2.18	-1.00	Both Isoforms

FBLN7		-1.46	-1.02	Both Isoforms
WNT2B		-1.27	-1.02	Both Isoforms
VANGL2		-0.74	-1.02	Both Isoforms
TRERF1		-0.90	-1.02	Both Isoforms
EFNA3		-0.68	-1.01	Both Isoforms
FRMD5		-1.83	-1.01	Both Isoforms
GLCCI1		-1.03	-1.01	Both Isoforms
CCDC14		-0.95	-1.01	Both Isoforms
RNF43		-1.41	-1.01	Both Isoforms
NR1D1		-1.15	-1.01	Both Isoforms
ALX4		-1.68	-1.01	Both Isoforms
KIF7		-0.95	-1.01	Both Isoforms
LRRN2		-1.84	-1.01	Both Isoforms
ZNF365		-1.17	-1.01	Both Isoforms
SKA3		-0.73	-1.01	Both Isoforms
RGS10		-0.84	-1.00	Both Isoforms
FAM81A		-0.81	-1.00	Both Isoforms
CCNJL		-1.61	-1.00	Both Isoforms
ADAP1		-1.83	-1.00	Both Isoforms
	11-Sep	-0.74	-1.00	Both Isoforms
ABCG2		-2.65	-1.00	Both Isoforms
PCDHGB4		-0.79	-1.00	Both Isoforms
CSMD1		-1.38	-1.00	Both Isoforms
PNMA3		-0.67	-1.00	Both Isoforms
FZD3		-1.76	-1.00	Both Isoforms

EIF4EBP3	-1.88	-1.00	Both Isoforms
LRIG3	-1.47	-1.00	Both Isoforms
GARNL3	-1.18	-1.00	Both Isoforms
MLLT3	-1.17	-1.00	Both Isoforms
STOM	-1.67	-1.00	Both Isoforms
GLCCI1	-0.65	-1.00	Both Isoforms
PYGL	-2.35	-0.99	Both Isoforms
ARHGAP22	-2.77	-0.99	Both Isoforms
PARK2	-2.55	-0.99	Both Isoforms
LCA5	-1.76	-0.99	Both Isoforms
TMPRSS13	-1.47	-0.99	Both Isoforms
BEX2	-1.55	-0.99	Both Isoforms
ZCCHC7	-0.87	-0.99	Both Isoforms
EXPH5	-1.57	-0.99	Both Isoforms
ICAM5	-2.46	-0.99	Both Isoforms
C1QTNF3	-1.40	-0.99	Both Isoforms
REXO2	-1.33	-0.99	Both Isoforms
SMAD3	-2.40	-0.99	Both Isoforms
PRR26	-1.97	-0.99	Both Isoforms
DUSP16	-0.68	-0.99	Both Isoforms
PLK2	-2.80	-0.98	Both Isoforms
SERPINA10	-3.36	-0.98	Both Isoforms
BDH2	-1.30	-0.98	Both Isoforms
RNF19A	-1.18	-0.98	Both Isoforms
AKAP6	-1.31	-0.98	Both Isoforms

DTX3	-1.01	-0.99	Both Isoforms
CA11	-0.86	-0.99	Both Isoforms
HOXC13	-1.37	-0.99	Both Isoforms
RPP25	-1.23	-0.99	Both Isoforms
SCRN1	-0.93	-0.99	Both Isoforms
GOLGA6L10	-2.45	-0.99	Both Isoforms
COL5A1	-2.01	-0.99	Both Isoforms
ETS1	-0.74	-0.98	Both Isoforms
SBK1	-0.61	-0.98	Both Isoforms
ZNF117	-1.05	-0.98	Both Isoforms
SMAD1	-0.78	-0.98	Both Isoforms
CNIH3	-1.06	-0.97	Both Isoforms
MCU	-0.81	-0.97	Both Isoforms
SH3BGRL2	-0.88	-0.97	Both Isoforms
SHC4	-1.13	-0.97	Both Isoforms
ISM2	-1.37	-0.97	Both Isoforms
CNGA1	-1.53	-0.97	Both Isoforms
PLEKHA2	-0.77	-0.97	Both Isoforms
BMX	-1.52	-0.97	Both Isoforms
CTB-55O6.8	-1.17	-0.96	Both Isoforms
CDCA7L	-1.54	-0.96	Both Isoforms
WASF1	-0.70	-0.96	Both Isoforms
ACRV1	-0.88	-0.96	Both Isoforms
GRB10	-1.85	-0.96	Both Isoforms
MID1	-0.88	-0.95	Both Isoforms
ATXN7L1	-2.01	-0.98	Both Isoforms
LRRC16B	-1.60	-0.98	Both Isoforms
FRMPD2	-0.68	-0.98	Both Isoforms
MANBA	-1.45	-0.98	Both Isoforms
ADGRA2	-2.83	-0.98	Both Isoforms
PARD3B	-0.83	-0.98	Both Isoforms
MRAS	-0.64	-0.97	Both Isoforms
GJC3	-1.43	-0.97	Both Isoforms
TNIK	-0.70	-0.97	Both Isoforms
RASAL1	-1.16	-0.97	Both Isoforms
CAMK1D	-1.46	-0.97	Both Isoforms
PCDHB12	-1.41	-0.97	Both Isoforms
KIAA1462	-0.97	-0.97	Both Isoforms
STAT6	-1.22	-0.97	Both Isoforms
ZNF608	-0.60	-0.97	Both Isoforms
PRSS27	-1.11	-0.97	Both Isoforms
CABLES1	-1.41	-0.97	Both Isoforms
RASSF5	-1.88	-0.97	Both Isoforms
ALDH1L2	-2.75	-0.96	Both Isoforms
BTC	-1.50	-0.96	Both Isoforms
DENND2C	-1.83	-0.96	Both Isoforms
FAM227B	-0.97	-0.96	Both Isoforms
FAM155A	-1.19	-0.96	Both Isoforms
SMTNL2	-2.20	-0.96	Both Isoforms
NEO1	-0.84	-0.96	Both Isoforms

CFAP45	-1.03	-0.95	Both Isoforms	KCTD12	-2.49	-0.96	Both Isoforms
AC138969.4	-0.95	-0.95	Both Isoforms	GAL3ST1	-3.22	-0.96	Both Isoforms
PLEKHG5	-0.73	-0.95	Both Isoforms	ZNF345	-1.32	-0.96	Both Isoforms
EGLN3	-1.88	-0.95	Both Isoforms	CPA6	-2.11	-0.96	Both Isoforms
ADRBK2	-0.59	-0.95	Both Isoforms	BCL2L10	-1.71	-0.96	Both Isoforms
GSDMD	-1.34	-0.94	Both Isoforms	NADK2	-1.30	-0.95	Both Isoforms
NUP54	-0.81	-0.94	Both Isoforms	RAI2	-1.01	-0.95	Both Isoforms
ADAMTSL4	-1.06	-0.94	Both Isoforms	NR4A2	-2.91	-0.95	Both Isoforms
PRKD1	-1.36	-0.94	Both Isoforms	CDKL5	-1.13	-0.95	Both Isoforms
CORO1A	-1.57	-0.94	Both Isoforms	TMEM173	-1.60	-0.95	Both Isoforms
DTX4	-1.26	-0.94	Both Isoforms	RPS18	-1.10	-0.95	Both Isoforms
TRPS1	-1.22	-0.94	Both Isoforms	CNKSR3	-1.95	-0.95	Both Isoforms
STOX1	-2.34	-0.94	Both Isoforms	C4orf36	-1.40	-0.95	Both Isoforms
IFIT5	-0.79	-0.94	Both Isoforms	PRICKLE1	-1.42	-0.95	Both Isoforms
E2F2	-1.30	-0.93	Both Isoforms	DAPK2	-1.71	-0.95	Both Isoforms
IKZF2	-0.93	-0.93	Both Isoforms	ZNF594	-1.42	-0.95	Both Isoforms
FZD7	-0.78	-0.92	Both Isoforms	WNT8B	-2.17	-0.95	Both Isoforms
IGIP	-1.10	-0.92	Both Isoforms	PCDHGB7	-2.20	-0.95	Both Isoforms
PCNA	-0.81	-0.92	Both Isoforms	SAMD4A	-1.93	-0.94	Both Isoforms
FOLH1	-0.79	-0.92	Both Isoforms	SYNGAP1	-1.49	-0.94	Both Isoforms
MCM5	-0.97	-0.92	Both Isoforms	ZFP92	-1.73	-0.94	Both Isoforms
HELLS	-1.32	-0.92	Both Isoforms	MKX	-2.51	-0.94	Both Isoforms
C1QTNF6	-1.85	-0.91	Both Isoforms	SGMS1	-0.95	-0.94	Both Isoforms
APBA2	-0.80	-0.91	Both Isoforms	NUDT4	-1.06	-0.94	Both Isoforms
CCDC85B	-0.73	-0.91	Both Isoforms	ZNF33B	-1.24	-0.94	Both Isoforms

HOXA10	-1.35	-0.91	Both Isoforms	NOB1	-1.27	-0.94	Both Isoforms
ANKRD46	-0.62	-0.91	Both Isoforms	QKI	-1.70	-0.94	Both Isoforms
ENKUR	-0.75	-0.91	Both Isoforms	PAX2	-1.59	-0.94	Both Isoforms
IL36G	-3.21	-0.91	Both Isoforms	ABTB2	-1.14	-0.94	Both Isoforms
BLNK	-1.07	-0.91	Both Isoforms	ZNF660	-2.88	-0.94	Both Isoforms
VASH1	-1.09	-0.91	Both Isoforms	MAP3K7CL	-1.14	-0.94	Both Isoforms
DPYSL5	-2.22	-0.91	Both Isoforms	ASPH	-2.07	-0.93	Both Isoforms
RHBG	-1.75	-0.90	Both Isoforms	DSG2	-0.71	-0.93	Both Isoforms
WDR62	-1.01	-0.90	Both Isoforms	LIPM	-1.27	-0.93	Both Isoforms
LMX1B	-1.08	-0.90	Both Isoforms	CCSER1	-1.28	-0.93	Both Isoforms
E2F1	-0.94	-0.90	Both Isoforms	TRIM7	-1.24	-0.93	Both Isoforms
SAMHD1	-0.71	-0.90	Both Isoforms	SPINK1	-1.51	-0.93	Both Isoforms
TEAD4	-0.71	-0.90	Both Isoforms	CLRN3	-2.13	-0.93	Both Isoforms
ABHD18	-1.11	-0.90	Both Isoforms	MTSS1	-1.07	-0.93	Both Isoforms
ATP11C	-1.33	-0.89	Both Isoforms	BASP1	-1.39	-0.93	Both Isoforms
FBXL7	-1.69	-0.89	Both Isoforms	CYP27B1	-1.65	-0.93	Both Isoforms
BROX	-0.60	-0.89	Both Isoforms	CD55	-1.74	-0.93	Both Isoforms
KIAA1324	-2.13	-0.89	Both Isoforms	NCAM1	-1.62	-0.93	Both Isoforms
PBX1	-1.65	-0.89	Both Isoforms	ZIC5	-1.87	-0.93	Both Isoforms
MREG	-1.20	-0.89	Both Isoforms	TGM3	-2.42	-0.93	Both Isoforms
ANXA9	-1.02	-0.89	Both Isoforms	ULBP1	-2.23	-0.93	Both Isoforms
SLC6A11	-0.73	-0.88	Both Isoforms	CACNB4	-1.75	-0.93	Both Isoforms
SLFN5	-1.28	-0.88	Both Isoforms	CASP4	-1.60	-0.93	Both Isoforms
PIK3IP1	-1.43	-0.88	Both Isoforms	MS4A8	-0.66	-0.93	Both Isoforms
CUEDC1	-0.61	-0.88	Both Isoforms	LGALS4	-0.72	-0.93	Both Isoforms

VOPP1	-0.93	-0.88	Both Isoforms	BFSP1	-1.15	-0.93	Both Isoforms
RGS17	-0.88	-0.88	Both Isoforms	PRSS3	-1.32	-0.93	Both Isoforms
MND1	-1.09	-0.88	Both Isoforms	CELF5	-1.84	-0.93	Both Isoforms
BCHE	-2.70	-0.88	Both Isoforms	INHBB	-1.20	-0.92	Both Isoforms
RFX2	-1.03	-0.88	Both Isoforms	ITGA2B	-1.88	-0.92	Both Isoforms
RASGRP1	-0.99	-0.87	Both Isoforms	ACCS	-1.59	-0.92	Both Isoforms
IGSF9	-0.83	-0.87	Both Isoforms	SCN8A	-1.75	-0.92	Both Isoforms
STC2	-1.13	-0.87	Both Isoforms	CPNE4	-0.70	-0.92	Both Isoforms
B4GALNT4	-0.91	-0.87	Both Isoforms	CADM1	-0.62	-0.92	Both Isoforms
FYN	-2.15	-0.87	Both Isoforms	ADD3	-2.13	-0.92	Both Isoforms
ARHGAP42	-0.83	-0.87	Both Isoforms	DCLK2	-1.23	-0.92	Both Isoforms
TNFRSF25	-0.92	-0.86	Both Isoforms	SNX24	-0.66	-0.92	Both Isoforms
GRK5	-1.19	-0.86	Both Isoforms	ISX	-0.81	-0.91	Both Isoforms
CBR3	-0.96	-0.86	Both Isoforms	PPP1R12B	-1.26	-0.91	Both Isoforms
COL9A2	-1.04	-0.86	Both Isoforms	TMPRSS9	-1.96	-0.91	Both Isoforms
AVPI1	-0.78	-0.86	Both Isoforms	TAF4B	-1.62	-0.91	Both Isoforms
SUSD6	-1.23	-0.85	Both Isoforms	MB21D2	-3.08	-0.91	Both Isoforms
HIBADH	-0.97	-0.85	Both Isoforms	PRX	-1.49	-0.91	Both Isoforms
TMEM133	-0.83	-0.85	Both Isoforms	FAM234B	-0.92	-0.91	Both Isoforms
KHDRBS3	-0.95	-0.85	Both Isoforms	KHDRBS3	-1.85	-0.91	Both Isoforms
JDP2	-1.29	-0.85	Both Isoforms	BBC3	-2.38	-0.91	Both Isoforms
SYTL3	-0.65	-0.85	Both Isoforms	KLC3	-1.38	-0.91	Both Isoforms
GXYLT2	-1.01	-0.85	Both Isoforms	CREB5	-1.94	-0.91	Both Isoforms
TIMP2	-1.12	-0.85	Both Isoforms	NABP1	-1.50	-0.91	Both Isoforms
NKD1	-0.91	-0.85	Both Isoforms	IMPA1	-0.84	-0.91	Both Isoforms

RAD54L	-1.15	-0.84	Both Isoforms	LRRIQ3	-1.36	-0.91	Both Isoforms
ZBTB43	-1.25	-0.84	Both Isoforms	HOXD13	-1.51	-0.91	Both Isoforms
TMEM121	-0.93	-0.84	Both Isoforms	SLC18B1	-2.67	-0.91	Both Isoforms
CDCA7	-0.97	-0.84	Both Isoforms	RAVER2	-1.52	-0.91	Both Isoforms
FA2H	-0.99	-0.84	Both Isoforms	DEPTOR	-1.68	-0.90	Both Isoforms
CECR1	-1.24	-0.84	Both Isoforms	ODF3B	-0.97	-0.90	Both Isoforms
CDON	-1.03	-0.84	Both Isoforms	CHMP4C	-1.16	-0.90	Both Isoforms
CDC45	-1.11	-0.83	Both Isoforms	ZRANB3	-1.77	-0.90	Both Isoforms
GINS2	-0.71	-0.83	Both Isoforms	TTC21B	-0.80	-0.90	Both Isoforms
SLC27A2	-1.10	-0.83	Both Isoforms	GAD1	-1.86	-0.90	Both Isoforms
XXYLT1	-0.58	-0.83	Both Isoforms	NMNAT3	-1.97	-0.90	Both Isoforms
EFR3B	-1.40	-0.83	Both Isoforms	ANKRD36	-1.78	-0.90	Both Isoforms
NEIL3	-0.59	-0.83	Both Isoforms	TSPAN5	-1.27	-0.90	Both Isoforms
CENPU	-0.88	-0.83	Both Isoforms	HS2ST1	-0.71	-0.90	Both Isoforms
C4orf32	-1.21	-0.83	Both Isoforms	JMY	-1.02	-0.90	Both Isoforms
KCNQ1	-1.05	-0.83	Both Isoforms	MCAM	-3.89	-0.90	Both Isoforms
ZNF624	-0.76	-0.82	Both Isoforms	RARG	-2.10	-0.90	Both Isoforms
ABCA1	-4.16	-0.82	Both Isoforms	SP100	-1.40	-0.90	Both Isoforms
FMNL3	-1.26	-0.82	Both Isoforms	CCDC110	-1.06	-0.90	Both Isoforms
PRELID3A	-0.76	-0.82	Both Isoforms	CCDC28A	-1.37	-0.90	Both Isoforms
RABL3	-0.61	-0.81	Both Isoforms	ARL4C	-0.67	-0.90	Both Isoforms
GNAZ	-1.19	-0.81	Both Isoforms	RYR2	-1.74	-0.90	Both Isoforms
SLC22A11	-0.71	-0.81	Both Isoforms	LAMB1	-1.35	-0.90	Both Isoforms
MCM10	-1.30	-0.81	Both Isoforms	MBTD1	-1.35	-0.89	Both Isoforms
CCNE2	-1.29	-0.81	Both Isoforms	KRCC1	-1.19	-0.89	Both Isoforms

IGFBP2	-0.82	-0.80	Both Isoforms	VPS37D	-0.82	-0.89	Both Isoforms
CHEK2	-0.71	-0.80	Both Isoforms	SYNPO2	-3.48	-0.89	Both Isoforms
TGIF2	-0.62	-0.80	Both Isoforms	GCNT1	-1.00	-0.89	Both Isoforms
RAVER2	-0.87	-0.80	Both Isoforms	DTX4	-2.32	-0.89	Both Isoforms
TYMS	-0.76	-0.80	Both Isoforms	BAG2	-1.09	-0.89	Both Isoforms
HOXC12	-0.90	-0.80	Both Isoforms	ANKRD30B	-2.29	-0.89	Both Isoforms
KHK	-1.20	-0.80	Both Isoforms	FNDC4	-1.42	-0.89	Both Isoforms
MCM3	-0.94	-0.80	Both Isoforms	POU2F2	-0.83	-0.89	Both Isoforms
GRAMD1C	-1.06	-0.79	Both Isoforms	MATN2	-1.01	-0.89	Both Isoforms
TONSL	-0.95	-0.79	Both Isoforms	PIGR	-2.44	-0.89	Both Isoforms
PIPOX	-0.70	-0.78	Both Isoforms	RASSF2	-0.59	-0.89	Both Isoforms
C4orf46	-1.17	-0.78	Both Isoforms	JAK3	-1.80	-0.89	Both Isoforms
DNAH2	-0.88	-0.78	Both Isoforms	HHIPL1	-1.16	-0.88	Both Isoforms
EEF1A2	-0.72	-0.78	Both Isoforms	RLTPR	-1.66	-0.88	Both Isoforms
LPCAT4	-1.58	-0.78	Both Isoforms	C20orf194	-1.54	-0.88	Both Isoforms
WNT10A	-0.67	-0.78	Both Isoforms	SLIT1	-1.85	-0.88	Both Isoforms
SLC48A1	-0.97	-0.78	Both Isoforms	TMEM38B	-0.96	-0.88	Both Isoforms
CTSH	-1.03	-0.77	Both Isoforms	MAML2	-1.08	-0.88	Both Isoforms
ADGRV1	-1.23	-0.77	Both Isoforms	ZNF425	-1.04	-0.88	Both Isoforms
RCC1	-0.76	-0.77	Both Isoforms	ABHD14B	-1.58	-0.88	Both Isoforms
POLA2	-0.88	-0.77	Both Isoforms	HECTD2	-0.64	-0.88	Both Isoforms
C20orf27	-0.74	-0.77	Both Isoforms	ARL10	-2.31	-0.88	Both Isoforms
TMEM117	-1.08	-0.77	Both Isoforms	SH3BGR	-1.21	-0.88	Both Isoforms
TERT	-1.43	-0.77	Both Isoforms	OTUD1	-1.90	-0.88	Both Isoforms
TRIM16L	-0.59	-0.77	Both Isoforms	GPRASP2	-1.09	-0.88	Both Isoforms

FAM134B	-1.78	-0.77	Both Isoforms	POTEI	-0.71	-0.87	Both Isoforms
KLHL23	-0.60	-0.76	Both Isoforms	FOXD2	-1.70	-0.87	Both Isoforms
XRCC2	-1.20	-0.76	Both Isoforms	DYRK3	-1.99	-0.87	Both Isoforms
CPAMD8	-2.37	-0.76	Both Isoforms	NCOA7	-2.32	-0.87	Both Isoforms
MAP3K8	-1.44	-0.75	Both Isoforms	FERMT1	-1.34	-0.87	Both Isoforms
DPYSL2	-0.98	-0.75	Both Isoforms	ZSCAN12	-0.59	-0.87	Both Isoforms
GATA2	-0.68	-0.75	Both Isoforms	ITGB2	-1.64	-0.87	Both Isoforms
PRAC1	-1.08	-0.75	Both Isoforms	RELB	-1.54	-0.87	Both Isoforms
C11orf95	-1.10	-0.74	Both Isoforms	CBR4	-1.50	-0.87	Both Isoforms
MCM6	-0.76	-0.74	Both Isoforms	R3HDM2	-0.87	-0.87	Both Isoforms
MAGI1	-0.63	-0.74	Both Isoforms	DNAJC18	-1.89	-0.87	Both Isoforms
SLC10A5	-0.69	-0.74	Both Isoforms	C8orf58	-0.97	-0.87	Both Isoforms
PURA	-0.85	-0.74	Both Isoforms	ANKRD26	-1.49	-0.87	Both Isoforms
MYBL2	-0.92	-0.74	Both Isoforms	WDR88	-1.62	-0.87	Both Isoforms
HACL1	-0.88	-0.74	Both Isoforms	LRRC37B	-1.08	-0.87	Both Isoforms
WDR91	-0.92	-0.73	Both Isoforms	DSE	-0.70	-0.87	Both Isoforms
GVQW2	-1.32	-0.73	Both Isoforms	MPP1	-1.52	-0.87	Both Isoforms
EXO1	-1.38	-0.73	Both Isoforms	ADAM22	-1.61	-0.87	Both Isoforms
TBC1D31	-0.74	-0.73	Both Isoforms	NR2F1	-3.05	-0.87	Both Isoforms
SLC1A3	-1.10	-0.73	Both Isoforms	BTN3A3	-1.19	-0.87	Both Isoforms
SMKR1	-0.93	-0.73	Both Isoforms	TGFBR3L	-1.49	-0.87	Both Isoforms
HOXC9	-1.89	-0.73	Both Isoforms	SH3BGRL2	-1.12	-0.87	Both Isoforms
IL4R	-0.60	-0.73	Both Isoforms	PROCR	-1.29	-0.86	Both Isoforms
HOXB5	-0.73	-0.73	Both Isoforms	TFCP2L1	-1.62	-0.86	Both Isoforms
CLSPN	-1.06	-0.73	Both Isoforms	ZNF853	-2.00	-0.86	Both Isoforms

KATNAL2	-1.00	-0.72	Both Isoforms	C4orf50	-1.86	-0.86	Both Isoforms
DNAH10	-1.43	-0.72	Both Isoforms	LAMA3	-0.97	-0.86	Both Isoforms
RECQL4	-0.86	-0.72	Both Isoforms	PAX6	-1.15	-0.86	Both Isoforms
SLC34A3	-0.61	-0.72	Both Isoforms	BHLHE41	-1.05	-0.86	Both Isoforms
OIP5	-0.85	-0.72	Both Isoforms	NLGN3	-0.72	-0.86	Both Isoforms
CYFIP2	-0.91	-0.72	Both Isoforms	PELI2	-1.87	-0.86	Both Isoforms
BEND3	-0.60	-0.72	Both Isoforms	N4BP2	-0.71	-0.86	Both Isoforms
CRMP1	-0.97	-0.71	Both Isoforms	NFU1	-1.20	-0.86	Both Isoforms
RCAN3	-0.90	-0.71	Both Isoforms	GRB10	-1.69	-0.86	Both Isoforms
ORC1	-1.12	-0.71	Both Isoforms	PDE10A	-1.32	-0.86	Both Isoforms
RIMKLA	-0.65	-0.71	Both Isoforms	TAS1R3	-1.64	-0.86	Both Isoforms
GALNT10	-0.62	-0.71	Both Isoforms	EFCC1	-1.54	-0.86	Both Isoforms
FERMT1	-0.67	-0.71	Both Isoforms	FBXO2	-1.71	-0.86	Both Isoforms
TP53	-1.12	-0.71	Both Isoforms	FAM49A	-1.45	-0.86	Both Isoforms
ZC3H6	-0.78	-0.71	Both Isoforms	GSC	-0.86	-0.85	Both Isoforms
TMEM38B	-1.23	-0.71	Both Isoforms	DPY19L1	-0.81	-0.85	Both Isoforms
ANKRD6	-1.12	-0.70	Both Isoforms	ZNF503	-1.33	-0.85	Both Isoforms
DENND1B	-1.16	-0.70	Both Isoforms	RDH16	-1.03	-0.85	Both Isoforms
EPM2A	-1.13	-0.70	Both Isoforms	DNAJC12	-0.96	-0.85	Both Isoforms
BID	-1.06	-0.70	Both Isoforms	THEM5	-1.90	-0.85	Both Isoforms
DSCC1	-0.88	-0.70	Both Isoforms	ZCWPW1	-1.15	-0.85	Both Isoforms
ITGB4	-1.46	-0.70	Both Isoforms	TMEM45A	-2.15	-0.85	Both Isoforms
POLD1	-0.77	-0.70	Both Isoforms	MYRF	-1.66	-0.85	Both Isoforms
NIPSNAP3A	-1.19	-0.70	Both Isoforms	ZMYND12	-1.52	-0.85	Both Isoforms
SSTR5	-2.85	-0.70	Both Isoforms	TPSG1	-0.76	-0.85	Both Isoforms

DAPK2	-1.44	-0.69	Both Isoforms	LIX1L	-1.68	-0.85	Both Isoforms
MVB12B	-0.88	-0.69	Both Isoforms	DRC7	-0.85	-0.85	Both Isoforms
RCC2	-0.63	-0.69	Both Isoforms	SYNE1	-0.83	-0.85	Both Isoforms
BMF	-1.96	-0.69	Both Isoforms	CPT1B	-0.87	-0.84	Both Isoforms
VASN	-0.63	-0.69	Both Isoforms	H2AFJ	-0.82	-0.84	Both Isoforms
MCM2	-1.23	-0.69	Both Isoforms	GSTA1	-1.08	-0.84	Both Isoforms
MGAT4A	-0.66	-0.69	Both Isoforms	PLIN1	-1.47	-0.84	Both Isoforms
TP53INP1	-1.47	-0.69	Both Isoforms	RCAN3	-0.87	-0.84	Both Isoforms
MANBA	-1.08	-0.69	Both Isoforms	UBA7	-0.84	-0.84	Both Isoforms
MB	-1.22	-0.69	Both Isoforms	CDK14	-0.85	-0.84	Both Isoforms
NFIX	-0.72	-0.69	Both Isoforms	GID4	-0.98	-0.84	Both Isoforms
TMEM229B	-1.31	-0.69	Both Isoforms	ZNF572	-1.38	-0.84	Both Isoforms
CLCN5	-0.63	-0.69	Both Isoforms	SETSIP	-0.66	-0.84	Both Isoforms
MCM4	-1.02	-0.69	Both Isoforms	FABP5	-2.23	-0.84	Both Isoforms
SLC25A19	-1.04	-0.68	Both Isoforms	RNF212	-2.33	-0.84	Both Isoforms
EVC	-0.81	-0.68	Both Isoforms	DZIP3	-1.43	-0.84	Both Isoforms
PPP1R14C	-2.44	-0.68	Both Isoforms	TARSL2	-1.15	-0.84	Both Isoforms
BBC3	-1.17	-0.68	Both Isoforms	HECA	-1.28	-0.84	Both Isoforms
GPC4	-0.69	-0.68	Both Isoforms	PPM1M	-1.17	-0.84	Both Isoforms
OAF	-0.58	-0.68	Both Isoforms	BEX4	-1.40	-0.84	Both Isoforms
ITPR1	-0.64	-0.68	Both Isoforms	TEX15	-1.71	-0.84	Both Isoforms
APOBEC3B	-0.69	-0.68	Both Isoforms	LTBP2	-1.96	-0.83	Both Isoforms
GAS6	-0.87	-0.68	Both Isoforms	ZNF391	-1.69	-0.83	Both Isoforms
GAREML	-0.73	-0.68	Both Isoforms	STARD9	-1.66	-0.83	Both Isoforms
CRAT	-0.83	-0.68	Both Isoforms	C12orf56	-0.58	-0.83	Both Isoforms

6-Sep	-1.13	-0.68	Both Isoforms
MTURN	-0.97	-0.68	Both Isoforms
SLC9A5	-0.66	-0.68	Both Isoforms
TIPIN	-0.77	-0.68	Both Isoforms
ARHGAP20	-2.35	-0.68	Both Isoforms
TUBB4A	-1.48	-0.68	Both Isoforms
CDT1	-1.11	-0.67	Both Isoforms
FANCD2	-0.84	-0.67	Both Isoforms
FIGNL1	-1.14	-0.67	Both Isoforms
GATS	-0.63	-0.67	Both Isoforms
GJC1	-0.80	-0.67	Both Isoforms
BTG2	-1.08	-0.67	Both Isoforms
LRIG3	-1.30	-0.66	Both Isoforms
PIGZ	-1.08	-0.66	Both Isoforms
POLQ	-1.07	-0.66	Both Isoforms
F12	-0.61	-0.66	Both Isoforms
BUB1B	-0.94	-0.66	Both Isoforms
PKN3	-0.73	-0.66	Both Isoforms
APCDD1	-1.18	-0.66	Both Isoforms
GALNT3	-1.27	-0.66	Both Isoforms
ESCO2	-0.96	-0.66	Both Isoforms
PAK1	-1.29	-0.65	Both Isoforms
OCLN	-0.96	-0.65	Both Isoforms
NKD2	-1.37	-0.65	Both Isoforms
NDUFAF6	-0.83	-0.65	Both Isoforms
GSN	-0.67	-0.83	Both Isoforms
RGL3	-2.15	-0.83	Both Isoforms
KIAA1257	-1.28	-0.83	Both Isoforms
TRIM29	-1.57	-0.83	Both Isoforms
SESN3	-2.38	-0.83	Both Isoforms
DOCK2	-1.34	-0.83	Both Isoforms
PTPN14	-0.80	-0.83	Both Isoforms
RNF180	-1.01	-0.83	Both Isoforms
TTLL6	-0.83	-0.83	Both Isoforms
PCDHB11	-0.90	-0.83	Both Isoforms
SLC9B2	-2.03	-0.83	Both Isoforms
RGS10	-0.78	-0.83	Both Isoforms
PLCE1	-1.17	-0.82	Both Isoforms
FOXB2	-2.21	-0.82	Both Isoforms
THG1L	-1.39	-0.82	Both Isoforms
AUH	-0.86	-0.82	Both Isoforms
AIDA	-1.68	-0.82	Both Isoforms
NLRP1	-2.43	-0.82	Both Isoforms
SMIM10L2A	-2.03	-0.82	Both Isoforms
MBOAT1	-1.85	-0.82	Both Isoforms
SVIL	-1.51	-0.82	Both Isoforms
PCDHA13	-1.36	-0.82	Both Isoforms
MYC	-2.21	-0.82	Both Isoforms
RBM7	-0.59	-0.82	Both Isoforms
ZNF230	-0.89	-0.82	Both Isoforms

SNRK	-0.64	-0.65	Both Isoforms	RYR1	-2.70	-0.82	Both Isoforms
FBXO2	-2.16	-0.65	Both Isoforms	CAMLG	-1.01	-0.82	Both Isoforms
FAM184A	-1.10	-0.65	Both Isoforms	GPR27	-2.28	-0.82	Both Isoforms
E2F8	-0.99	-0.65	Both Isoforms	SLC6A11	-2.17	-0.81	Both Isoforms
PARD6G	-0.85	-0.65	Both Isoforms	JDP2	-1.58	-0.81	Both Isoforms
MCM7	-0.85	-0.65	Both Isoforms	EIF3CL	-1.30	-0.81	Both Isoforms
PHC1	-0.88	-0.65	Both Isoforms	NMNAT2	-1.23	-0.81	Both Isoforms
C14orf80	-0.89	-0.64	Both Isoforms	PRR18	-2.26	-0.81	Both Isoforms
PTPRR	-1.45	-0.64	Both Isoforms	ITGA1	-1.04	-0.81	Both Isoforms
FBXO24	-1.01	-0.64	Both Isoforms	NMT2	-1.62	-0.81	Both Isoforms
ASF1B	-1.20	-0.64	Both Isoforms	AR	-2.83	-0.81	Both Isoforms
CPNE1	-0.60	-0.64	Both Isoforms	SLC25A18	-0.86	-0.81	Both Isoforms
COL18A1	-0.87	-0.64	Both Isoforms	MAP3K8	-0.64	-0.81	Both Isoforms
MYC	-0.66	-0.64	Both Isoforms	CNNM2	-0.91	-0.81	Both Isoforms
DTL	-1.16	-0.64	Both Isoforms	ATP11C	-0.82	-0.81	Both Isoforms
LMNB1	-0.88	-0.64	Both Isoforms	NLN	-1.16	-0.81	Both Isoforms
FHOD3	-1.80	-0.63	Both Isoforms	SIX1	-1.09	-0.81	Both Isoforms
GRB14	-0.71	-0.63	Both Isoforms	RAI14	-0.60	-0.80	Both Isoforms
RBL1	-0.99	-0.63	Both Isoforms	MYO7A	-2.69	-0.80	Both Isoforms
ADORA1	-2.40	-0.63	Both Isoforms	SCNN1G	-1.31	-0.80	Both Isoforms
SLC6A19	-1.55	-0.63	Both Isoforms	VWDE	-1.79	-0.80	Both Isoforms
TMPRSS13	-1.43	-0.63	Both Isoforms	RILPL2	-0.97	-0.80	Both Isoforms
CELSR2	-1.08	-0.63	Both Isoforms	PCP4	-0.59	-0.80	Both Isoforms
CENPM	-0.69	-0.63	Both Isoforms	PTCH1	-1.33	-0.80	Both Isoforms
DNMT1	-0.64	-0.63	Both Isoforms	ASB14	-0.62	-0.80	Both Isoforms

LRRC37A3	-1.00	-0.63	Both Isoforms	CYBB	-1.05	-0.80	Both Isoforms
PRR36	-0.66	-0.63	Both Isoforms	MREG	-1.09	-0.80	Both Isoforms
GLIS2	-1.00	-0.63	Both Isoforms	SNX30	-1.56	-0.80	Both Isoforms
PER3	-0.70	-0.62	Both Isoforms	ZNF296	-1.58	-0.80	Both Isoforms
RAB30	-0.64	-0.62	Both Isoforms	PLCG2	-1.49	-0.80	Both Isoforms
CHST15	-0.65	-0.62	Both Isoforms	ABTB1	-1.45	-0.80	Both Isoforms
WDR76	-1.02	-0.62	Both Isoforms	PROZ	-1.42	-0.80	Both Isoforms
SKA1	-0.92	-0.62	Both Isoforms	PDP1	-0.84	-0.79	Both Isoforms
TRNP1	-1.28	-0.61	Both Isoforms	DOCK10	-2.74	-0.79	Both Isoforms
TUB	-0.66	-0.61	Both Isoforms	ZNF284	-1.72	-0.79	Both Isoforms
ST6GALNAC4	-1.05	-0.61	Both Isoforms	DOCK3	-1.09	-0.79	Both Isoforms
DDB2	-1.28	-0.61	Both Isoforms	MAPRE2	-1.11	-0.79	Both Isoforms
E2F7	-1.05	-0.61	Both Isoforms	PIFO	-0.68	-0.79	Both Isoforms
ADAM22	-0.77	-0.61	Both Isoforms	BAHCC1	-2.31	-0.79	Both Isoforms
ZNF680	-0.74	-0.61	Both Isoforms	GSTA4	-1.39	-0.79	Both Isoforms
ARHGAP29	-0.97	-0.61	Both Isoforms	SAMD11	-0.71	-0.79	Both Isoforms
N4BP3	-1.01	-0.61	Both Isoforms	PCDHB10	-0.85	-0.79	Both Isoforms
UHRF1	-1.13	-0.60	Both Isoforms	KLK14	-1.17	-0.79	Both Isoforms
TMCC2	-0.72	-0.60	Both Isoforms	NOA1	-0.69	-0.79	Both Isoforms
CHAF1A	-0.88	-0.60	Both Isoforms	TMOD2	-1.72	-0.79	Both Isoforms
CHAF1B	-0.95	-0.60	Both Isoforms	GABRD	-0.74	-0.79	Both Isoforms
EPHA10	-0.58	-0.60	Both Isoforms	GRAMD1C	-0.86	-0.79	Both Isoforms
GNG7	-0.81	-0.60	Both Isoforms	IL22RA1	-1.50	-0.79	Both Isoforms
RAD51	-0.96	-0.60	Both Isoforms	CREBRF	-1.51	-0.79	Both Isoforms
FAM182B	-0.83	-0.60	Both Isoforms	RAC2	-1.35	-0.78	Both Isoforms

TBX19	-1.08	-0.60	Both Isoforms	CDK7	-0.90	-0.78	Both Isoforms
ASCL5	-0.76	-0.60	Both Isoforms	CCL2	-2.13	-0.78	Both Isoforms
FBXO5	-0.63	-0.60	Both Isoforms	SIDT1	-1.36	-0.78	Both Isoforms
FANCA	-0.77	-0.60	Both Isoforms	SMAD1	-1.24	-0.78	Both Isoforms
CENPH	-0.68	-0.60	Both Isoforms	SCAI	-0.73	-0.78	Both Isoforms
WNT4	-1.36	-0.59	Both Isoforms	ADORA2B	-1.30	-0.78	Both Isoforms
BEND7	-1.10	-0.59	Both Isoforms	PROSER2	-0.68	-0.78	Both Isoforms
ABTB2	-1.22	-0.59	Both Isoforms	SPATS2L	-0.87	-0.78	Both Isoforms
GLCE	-0.61	-0.59	Both Isoforms	RPS6KA6	-1.20	-0.78	Both Isoforms
SOX11	-1.06	-0.59	Both Isoforms	ACVR2B	-0.66	-0.78	Both Isoforms
PCDHB11	-0.60	-0.59	Both Isoforms	TGIF2	-1.19	-0.78	Both Isoforms
FOXM1	-0.66	-0.59	Both Isoforms	POU3F3	-1.82	-0.78	Both Isoforms
ADGRL1	-0.62	-0.59	Both Isoforms	NT5C	-1.11	-0.78	Both Isoforms
WDR54	-0.65	-0.58	Both Isoforms	GLI2	-1.97	-0.78	Both Isoforms
VWA2	-3.56	-0.58	Both Isoforms	PTK7	-1.32	-0.78	Both Isoforms
SORL1	-1.71	-0.58	Both Isoforms	ADPRHL1	-1.12	-0.78	Both Isoforms
LNX2	-0.73	-0.58	Both Isoforms	DMGDH	-1.44	-0.78	Both Isoforms
CBX6	-0.80	-0.58	AR-FL	AGA	-0.83	-0.78	Both Isoforms
LAPTM4B	-0.98	-0.58	AR-FL	EIF4B	-1.03	-0.78	Both Isoforms
AC013461.1	-0.61	-0.58	AR-FL	ARRDC3	-1.44	-0.78	Both Isoforms
ADD2	-2.39	-0.57	AR-FL	PGM2	-1.15	-0.78	Both Isoforms
HMGA1	-0.81	-0.57	AR-FL	MAP2K5	-1.00	-0.78	Both Isoforms
ARMCX4	-0.74	-0.57	AR-FL	OSR2	-1.29	-0.78	Both Isoforms
NCAPH	-0.99	-0.57	AR-FL	LNPEP	-0.86	-0.78	Both Isoforms
DPY19L1	-0.98	-0.57	AR-FL	CERK	-2.01	-0.77	Both Isoforms
DACT3	-1.08	-0.57	AR-FL	ZNF658	-1.13	-0.77	Both Isoforms
BMP8B	-0.70	-0.57	AR-FL	FAAH2	-0.97	-0.77	Both Isoforms
ADCY3	-0.65	-0.57	AR-FL	TMCO6	-1.58	-0.77	Both Isoforms
GEMIN4	-0.64	-0.57	AR-FL	PARD6G	-1.17	-0.77	Both Isoforms
HOXB3	-0.67	-0.57	AR-FL	TMEM136	-1.44	-0.77	Both Isoforms
PODXL	-0.77	-0.57	AR-FL	PCNXL2	-0.62	-0.77	Both Isoforms
ME2	-0.86	-0.57	AR-FL	PRAC1	-1.09	-0.77	Both Isoforms

CENPV	-0.65	-0.57	AR-FL	THNSL1	-2.07	-0.77	Both Isoforms
XRCC6BP1	-0.59	-0.56	AR-FL	HTRA3	-1.34	-0.77	Both Isoforms
ENHO	-0.97	-0.56	AR-FL	CRTAM	-1.33	-0.77	Both Isoforms
GLDC	-1.14	-0.56	AR-FL	CTSK	-0.75	-0.77	Both Isoforms
TLL2	-1.08	-0.56	AR-FL	ZNF704	-1.26	-0.77	Both Isoforms
SLC9A7	-0.84	-0.56	AR-FL	PKHD1	-0.86	-0.77	Both Isoforms
CDCA5	-1.08	-0.56	AR-FL	IFRD1	-0.70	-0.77	Both Isoforms
PKMYT1	-1.04	-0.56	AR-FL	KCNJ2	-1.23	-0.77	Both Isoforms
MKI67	-0.63	-0.56	AR-FL	RAB6C	-0.77	-0.77	Both Isoforms
BRCA1	-1.01	-0.56	AR-FL	LSM11	-1.60	-0.76	Both Isoforms
FUT8	-0.84	-0.56	AR-FL	RBMS2	-0.97	-0.76	Both Isoforms
WNT10B	-0.79	-0.55	AR-FL	NCOA2	-1.03	-0.76	Both Isoforms
SESN2	-1.16	-0.55	AR-FL	ELOVL4	-0.78	-0.76	Both Isoforms
GTF2IRD2B	-1.16	-0.55	AR-FL	PCID2	-0.85	-0.76	Both Isoforms
EML4	-0.58	-0.55	AR-FL	C1orf145	-2.26	-0.76	Both Isoforms
CCNA2	-0.78	-0.55	AR-FL	LACE1	-0.59	-0.76	Both Isoforms
PIK3C2B	-0.73	-0.55	AR-FL	ACY3	-0.80	-0.76	Both Isoforms
CCDC102B	-1.29	-0.55	AR-FL	FN1	-0.62	-0.76	Both Isoforms
NOTCH1	-0.80	-0.55	AR-FL	TTC23	-1.37	-0.76	Both Isoforms
ADGRB2	-1.79	-0.55	AR-FL	POLR3C	-1.19	-0.76	Both Isoforms
ST5	-1.36	-0.55	AR-FL	DSEL	-2.24	-0.76	Both Isoforms
ITGA2	-0.87	-0.55	AR-FL	FAM83G	-0.66	-0.76	Both Isoforms
ESPN	-1.97	-0.55	AR-FL	RGS22	-1.35	-0.76	Both Isoforms
RALGPS1	-0.75	-0.55	AR-FL	ERICH2	-1.65	-0.76	Both Isoforms
ZNRF3	-0.59	-0.55	AR-FL	CTNND2	-0.87	-0.76	Both Isoforms
RNF208	-0.90	-0.55	AR-FL	SDCCAG8	-0.96	-0.76	Both Isoforms
ATXN7L2	-0.64	-0.55	AR-FL	EFCAB2	-0.88	-0.76	Both Isoforms
CACNA2D2	-0.82	-0.54	AR-FL	C14orf37	-2.24	-0.76	Both Isoforms
CD83	-1.52	-0.54	AR-FL	NBPF1	-1.74	-0.76	Both Isoforms
FANCB	-1.00	-0.54	AR-FL	ZNF667	-1.76	-0.76	Both Isoforms
ZMYND15	-1.06	-0.54	AR-FL	ARNT2	-0.93	-0.75	Both Isoforms
OTUD3	-0.62	-0.54	AR-FL	IMPDH2	-1.43	-0.75	Both Isoforms
LTA4H	-0.69	-0.54	AR-FL	CCDC191	-1.98	-0.75	Both Isoforms
RNASEH2A	-0.65	-0.54	AR-FL	WSCD1	-1.94	-0.75	Both Isoforms
TMEM144	-1.14	-0.54	AR-FL	RBBP8NL	-1.08	-0.75	Both Isoforms
EFCAB11	-1.02	-0.54	AR-FL	MBLAC2	-1.15	-0.75	Both Isoforms
MAP7D3	-1.13	-0.54	AR-FL	FAM134B	-1.57	-0.75	Both Isoforms
HOXB6	-0.77	-0.54	AR-FL	ACER3	-1.00	-0.75	Both Isoforms
CCDC151	-1.03	-0.54	AR-FL	BANK1	-0.66	-0.75	Both Isoforms
AGO4	-0.78	-0.54	AR-FL	CDH2	-2.95	-0.75	Both Isoforms
SPTLC3	-1.55	-0.53	AR-FL	TMEM123	-0.67	-0.75	Both Isoforms
CCDC92	-0.79	-0.53	AR-FL	KCNK15	-3.03	-0.75	Both Isoforms
STXBP6	-1.29	-0.53	AR-FL	CLIP4	-1.55	-0.75	Both Isoforms
NIPAL1	-1.00	-0.53	AR-FL	FEZ1	-1.69	-0.75	Both Isoforms
TNFRSF21	-0.93	-0.53	AR-FL	IMMP2L	-0.67	-0.75	Both Isoforms
FDX1	-0.64	-0.53	AR-FL	PHC1	-1.68	-0.75	Both Isoforms

TIAM2	-0.94	-0.53	AR-FL	ZNF182	-0.95	-0.75	Both Isoforms
MELK	-0.75	-0.53	AR-FL	LAMP3	-0.75	-0.75	Both Isoforms
LOXL3	-1.79	-0.53	AR-FL	KIZ	-1.18	-0.75	Both Isoforms
C11orf70	-1.89	-0.53	AR-FL	WNT2B	-2.00	-0.74	Both Isoforms
PLEKHS1	-1.51	-0.53	AR-FL	EYA3	-1.10	-0.74	Both Isoforms
KIAA0101	-0.63	-0.53	AR-FL	ZNF559-ZNF177	-1.08	-0.74	Both Isoforms
THG1L	-0.73	-0.53	AR-FL	PDLIM2	-0.64	-0.74	Both Isoforms
CDC6	-0.62	-0.53	AR-FL	CALCOCO1	-1.58	-0.74	Both Isoforms
BCAM	-0.79	-0.52	AR-FL	HIST1H2BF	-0.73	-0.74	Both Isoforms
CACNB2	-1.20	-0.52	AR-FL	PARP3	-1.30	-0.74	Both Isoforms
TTC23L	-1.22	-0.52	AR-FL	MPP7	-0.67	-0.74	Both Isoforms
INPP5J	-0.74	-0.52	AR-FL	ODAM	-0.85	-0.74	Both Isoforms
LIG1	-0.83	-0.52	AR-FL	HUNK	-0.95	-0.74	Both Isoforms
EPHB6	-0.80	-0.52	AR-FL	POLG2	-1.43	-0.74	Both Isoforms
IMPDH2	-0.60	-0.52	AR-FL	PEX11G	-0.73	-0.74	Both Isoforms
ZNF462	-0.71	-0.52	AR-FL	ADCY7	-2.54	-0.74	Both Isoforms
RAB17	-0.67	-0.52	AR-FL	CNOT6L	-0.99	-0.74	Both Isoforms
NT5M	-0.62	-0.52	AR-FL	PLCH1	-0.67	-0.73	Both Isoforms
GJB1	-0.59	-0.52	AR-FL	DPH5	-1.30	-0.73	Both Isoforms
TSPAN15	-1.05	-0.52	AR-FL	XRN2	-0.71	-0.73	Both Isoforms
SYNE2	-0.65	-0.52	AR-FL	DHRS7	-0.62	-0.73	Both Isoforms
LHX4	-0.67	-0.52	AR-FL	RFX3	-1.28	-0.73	Both Isoforms
RNF130	-0.59	-0.52	AR-FL	TRIQK	-0.91	-0.73	Both Isoforms
NPIPA7	-0.81	-0.52	AR-FL	CPNE1	-0.79	-0.73	Both Isoforms
ATXN7L1	-0.85	-0.52	AR-FL	PCMTD2	-1.28	-0.73	Both Isoforms
C1orf233	-0.58	-0.51	AR-FL	CFAP161	-0.96	-0.73	Both Isoforms
SDHAF3	-0.60	-0.51	AR-FL	IGFBP4	-1.19	-0.73	Both Isoforms
DGKH	-1.70	-0.51	AR-FL	NFKB1	-1.35	-0.73	Both Isoforms
GTF2IRD2	-1.45	-0.51	AR-FL	RPL22L1	-0.67	-0.73	Both Isoforms
ADORA2B	-0.85	-0.51	AR-FL	DPYSL3	-0.89	-0.73	Both Isoforms
C5orf34	-1.03	-0.51	AR-FL	KIF12	-1.24	-0.73	Both Isoforms
GSC	-0.67	-0.51	AR-FL	CDK19	-0.76	-0.73	Both Isoforms
C1orf53	-0.89	-0.51	AR-FL	DZANK1	-0.75	-0.73	Both Isoforms
FAM212B	-0.98	-0.51	AR-FL	STOX1	-3.08	-0.73	Both Isoforms
ASTN2	-0.94	-0.51	AR-FL	DOK7	-0.84	-0.73	Both Isoforms
GARNL3	-1.05	-0.51	AR-FL	NHSL2	-1.40	-0.72	Both Isoforms
DOK1	-1.57	-0.51	AR-FL	PRELID3A	-1.00	-0.72	Both Isoforms
FANCI	-0.60	-0.50	AR-FL	IPMK	-0.96	-0.72	Both Isoforms
MLXIPL	-1.62	-0.50	AR-FL	SMAGP	-0.65	-0.72	Both Isoforms
STRBP	-0.98	-0.50	AR-FL	ANKRD46	-0.63	-0.72	Both Isoforms
TCOF1	-0.78	-0.50	AR-FL	ANKRD16	-1.37	-0.72	Both Isoforms
GAS2L3	-0.81	-0.50	AR-FL	CAMK2N1	-1.60	-0.72	Both Isoforms
PMS2	-0.86	-0.50	AR-FL	UPK3A	-1.96	-0.72	Both Isoforms
RFX3	-1.61	-0.50	AR-FL	MYT1	-0.83	-0.72	Both Isoforms
ISYNA1	-0.79	-0.50	AR-FL	IFIT2	-0.91	-0.72	Both Isoforms
HSD17B6	-0.69	-0.50	AR-FL	MRM1	-1.15	-0.72	Both Isoforms

SH3RF1	-1.07	-0.50	AR-FL	TMEM243	-1.01	-0.72	Both Isoforms
DCPS	-0.61	-0.49	AR-FL	KLHL35	-1.49	-0.72	Both Isoforms
HRSP12	-0.89	-0.49	AR-FL	UGT8	-1.47	-0.72	Both Isoforms
ERV3-1	-0.63	-0.49	AR-FL	NR1D1	-1.37	-0.72	Both Isoforms
RELL1	-1.06	-0.49	AR-FL	ATG2B	-1.15	-0.72	Both Isoforms
KIF18B	-1.03	-0.49	AR-FL	CCDC14	-1.16	-0.72	Both Isoforms
LIN9	-1.13	-0.49	AR-FL	PAQR8	-1.25	-0.72	Both Isoforms
NFAT5	-0.90	-0.49	AR-FL	BAZ2B	-0.97	-0.71	Both Isoforms
NAT8L	-1.69	-0.48	AR-FL	CYTH3	-1.05	-0.71	Both Isoforms
EZH2	-0.73	-0.48	AR-FL	RNLS	-0.70	-0.71	Both Isoforms
KIAA1958	-0.79	-0.48	AR-FL	RNF217	-2.15	-0.71	Both Isoforms
AJUBA	-1.22	-0.48	AR-FL	GRB14	-1.77	-0.71	Both Isoforms
HECTD2	-0.77	-0.48	AR-FL	STON2	-1.09	-0.71	Both Isoforms
RFC2	-0.68	-0.48	AR-FL	IRX2	-0.61	-0.71	Both Isoforms
CYB5RL	-0.69	-0.48	AR-FL	ADA	-1.18	-0.71	Both Isoforms
FAM149A	-0.71	-0.48	AR-FL	GPR156	-1.34	-0.71	Both Isoforms
MACROD1	-0.58	-0.48	AR-FL	RBMX	-0.74	-0.71	Both Isoforms
RNF144A	-0.69	-0.48	AR-FL	PIWIL2	-5.30	-0.71	Both Isoforms
RTKN2	-0.65	-0.48	AR-FL	PTPRU	-1.74	-0.71	Both Isoforms
ARHGAP11A	-0.61	-0.47	AR-FL	NT5C3A	-0.66	-0.71	Both Isoforms
SALL2	-1.31	-0.47	AR-FL	ZC3H8	-0.85	-0.71	Both Isoforms
PGAP1	-1.20	-0.47	AR-FL	ARMC4	-2.99	-0.71	Both Isoforms
RGL1	-0.74	-0.47	AR-FL	SETDB2	-0.70	-0.71	Both Isoforms
TPX2	-0.71	-0.47	AR-FL	GLTSCR1L	-0.82	-0.71	Both Isoforms
KANK2	-0.76	-0.47	AR-FL	CCDC171	-1.44	-0.71	Both Isoforms
NFKB1	-0.90	-0.47	AR-FL	EIF4E3	-2.13	-0.70	Both Isoforms
H2AFY2	-0.71	-0.47	AR-FL	PLCB4	-1.04	-0.70	Both Isoforms
CENPA	-0.66	-0.47	AR-FL	FKBP7	-0.70	-0.70	Both Isoforms
INSIG2	-0.70	-0.47	AR-FL	BID	-0.64	-0.70	Both Isoforms
PIK3R3	-1.06	-0.47	AR-FL	IRF2BP2	-1.07	-0.70	Both Isoforms
RTN4R	-0.94	-0.47	AR-FL	ELN	-0.78	-0.70	Both Isoforms
LRP5L	-0.67	-0.47	AR-FL	PROS1	-0.77	-0.70	Both Isoforms
PLK1	-0.64	-0.46	AR-FL	IGBP1	-1.02	-0.70	Both Isoforms
ATP11A	-0.93	-0.46	AR-FL	SAMD5	-1.49	-0.70	Both Isoforms
RBBP8	-0.73	-0.46	AR-FL	TRAM1L1	-0.83	-0.70	Both Isoforms
SLC22A2	-1.41	-0.46	AR-FL	KCNJ14	-1.32	-0.70	Both Isoforms
RNF19A	-0.59	-0.46	AR-FL	CCDC177	-0.91	-0.70	Both Isoforms
MAD2L1	-0.69	-0.46	AR-FL	METTL20	-0.72	-0.70	Both Isoforms
PROZ	-0.71	-0.46	AR-FL	SH2D2A	-1.95	-0.69	Both Isoforms
KIF15	-0.88	-0.46	AR-FL	FOXN2	-0.61	-0.69	Both Isoforms
IRX2	-0.66	-0.46	AR-FL	CAMK2D	-1.46	-0.69	Both Isoforms
KIF11	-0.78	-0.46	AR-FL	NTM	-1.31	-0.69	Both Isoforms
BZRAP1	-0.70	-0.46	AR-FL	PRR5L	-1.53	-0.69	Both Isoforms
CALD1	-0.76	-0.45	AR-FL	HAUS4	-1.27	-0.69	Both Isoforms
IRF2BPL	-0.80	-0.45	AR-FL	HOXB9	-1.01	-0.69	Both Isoforms
CKAP2L	-0.83	-0.45	AR-FL	POLR3G	-1.05	-0.69	Both Isoforms

KCTD17	-0.73	-0.45	AR-FL
ZBTB34	-0.74	-0.45	AR-FL
TDP1	-0.59	-0.45	AR-FL
IL15RA	-0.64	-0.45	AR-FL
NAIP	-0.58	-0.45	AR-FL
ZBTB47	-0.65	-0.45	AR-FL
CEP131	-0.65	-0.45	AR-FL
DBP	-1.09	-0.44	AR-FL
AURKB	-1.07	-0.44	AR-FL
GPD1	-0.83	-0.44	AR-FL
HFE	-0.95	-0.44	AR-FL
KALRN	-1.58	-0.44	AR-FL
BMP1	-1.17	-0.44	AR-FL
WDR90	-0.62	-0.44	AR-FL
MAP1A	-1.39	-0.44	AR-FL
NDC80	-0.76	-0.44	AR-FL
THAP10	-0.88	-0.44	AR-FL
ARHGEF40	-1.00	-0.44	AR-FL
SMIM10L2A	-0.95	-0.44	AR-FL
LRRC20	-0.75	-0.44	AR-FL
WDR88	-0.72	-0.44	AR-FL
TM7SF2	-0.74	-0.44	AR-FL
GLS2	-1.07	-0.43	AR-FL
H2AFX	-0.79	-0.43	AR-FL
TGM3	-3.69	-0.43	AR-FL
ELOVL6	-1.02	-0.43	AR-FL
HSPA12A	-0.85	-0.43	AR-FL
9-Sep	-0.62	-0.43	AR-FL
MCC	-2.27	-0.43	AR-FL
ZNF736	-0.86	-0.42	AR-FL
SESN3	-1.21	-0.42	AR-FL
C16orf59	-0.77	-0.42	AR-FL
SLC13A4	-1.55	-0.42	AR-FL
TLR5	-0.98	-0.42	AR-FL
C21orf58	-0.72	-0.42	AR-FL
LGALS3BP	-0.59	-0.42	AR-FL
NUDT11	-0.70	-0.42	AR-FL
NET1	-0.74	-0.42	AR-FL
CLYBL	-1.25	-0.42	AR-FL
RDM1	-0.94	-0.42	AR-FL
DMBX1	-1.62	-0.42	AR-FL
KITLG	-1.05	-0.42	AR-FL
SSBP2	-2.36	-0.41	AR-FL
TICRR	-0.92	-0.41	AR-FL
FAM177B	-1.79	-0.41	AR-FL
ADCY7	-2.53	-0.41	AR-FL
SDC4	-1.30	-0.69	Both Isoforms
SP110	-1.30	-0.69	Both Isoforms
SOX1	-1.51	-0.69	Both Isoforms
TCAIM	-0.66	-0.69	Both Isoforms
ZNF485	-0.75	-0.69	Both Isoforms
ANKRD22	-1.26	-0.69	Both Isoforms
ATG9B	-1.38	-0.69	Both Isoforms
SIK2	-0.80	-0.69	Both Isoforms
IKZF2	-0.95	-0.69	Both Isoforms
ANKRD50	-2.19	-0.69	Both Isoforms
RASAL2	-1.85	-0.69	Both Isoforms
TEC	-0.88	-0.69	Both Isoforms
LMCD1	-0.61	-0.69	Both Isoforms
SPINK5	-1.31	-0.69	Both Isoforms
DCLK1	-1.03	-0.69	Both Isoforms
IL7	-1.91	-0.69	Both Isoforms
DHX40	-1.04	-0.69	Both Isoforms
ALKBH2	-1.23	-0.69	Both Isoforms
ARHGEF10	-0.72	-0.68	Both Isoforms
RASD2	-0.71	-0.68	Both Isoforms
PPP1R3B	-0.68	-0.68	Both Isoforms
STX7	-1.16	-0.68	Both Isoforms
FXYD5	-1.47	-0.68	Both Isoforms
RAB39B	-1.01	-0.68	Both Isoforms
GNG4	-1.92	-0.68	Both Isoforms
METAP2	-0.69	-0.68	Both Isoforms
OBSCN	-2.69	-0.68	Both Isoforms
SPTBN5	-1.14	-0.68	Both Isoforms
ERICH5	-1.30	-0.68	Both Isoforms
CYP4V2	-0.99	-0.68	Both Isoforms
SPRY2	-1.31	-0.68	Both Isoforms
EFHC2	-1.34	-0.68	Both Isoforms
ITGB1BP2	-1.22	-0.68	Both Isoforms
AFF1	-0.80	-0.68	Both Isoforms
ANTXR1	-1.76	-0.68	Both Isoforms
SLC25A26	-1.09	-0.68	Both Isoforms
HBP1	-0.99	-0.68	Both Isoforms
GOLIM4	-0.94	-0.68	Both Isoforms
PCDHGA1	-0.78	-0.68	Both Isoforms
NHLRC4	-1.89	-0.68	Both Isoforms
UBASH3B	-1.74	-0.68	Both Isoforms
DDX60	-1.06	-0.68	Both Isoforms
NPM1	-0.99	-0.68	Both Isoforms
FMO5	-1.39	-0.67	Both Isoforms
EFCAB11	-1.17	-0.67	Both Isoforms
TMC8	-1.02	-0.67	Both Isoforms

RP11-723O4.6	-0.87	-0.41	AR-FL
FRMPD2	-1.62	-0.41	AR-FL
KIF20B	-0.82	-0.41	AR-FL
HSD3B7	-0.66	-0.41	AR-FL
MFI2	-0.76	-0.41	AR-FL
HAUS8	-0.90	-0.41	AR-FL
DEPDC1B	-0.59	-0.41	AR-FL
PSRC1	-0.73	-0.41	AR-FL
PVRL4	-0.59	-0.41	AR-FL
PATZ1	-0.77	-0.41	AR-FL
CCDC28B	-0.98	-0.41	AR-FL
GINS4	-0.84	-0.41	AR-FL
RASSF5	-1.54	-0.41	AR-FL
C16orf74	-0.93	-0.40	AR-FL
PTPRB	-1.94	-0.40	AR-FL
SLC16A2	-0.73	-0.40	AR-FL
ITGB1BP1	-0.62	-0.40	AR-FL
TNFAIP2	-1.43	-0.40	AR-FL
SLC18B1	-1.25	-0.40	AR-FL
DOC2A	-0.94	-0.39	AR-FL
DSEL	-1.72	-0.39	AR-FL
CEP78	-0.72	-0.39	AR-FL
PRIM1	-1.01	-0.39	AR-FL
IRF5	-0.69	-0.39	AR-FL
FHAD1	-1.05	-0.39	AR-FL
VPS37D	-0.87	-0.39	AR-FL
IL1RAP	-1.07	-0.39	AR-FL
WLS	-1.46	-0.39	AR-FL
FAM102A	-0.76	-0.39	AR-FL
SLC20A2	-0.62	-0.39	AR-FL
RFXAP	-0.65	-0.39	AR-FL
TK1	-0.96	-0.39	AR-FL
CMTM1	-0.66	-0.39	AR-FL
SPC24	-0.98	-0.39	AR-FL
FANCG	-0.66	-0.39	AR-FL
SYNM	-0.74	-0.39	AR-FL
KNTC1	-0.83	-0.39	AR-FL
SYBU	-1.01	-0.39	AR-FL
RBP5	-1.27	-0.39	AR-FL
GINS1	-0.89	-0.38	AR-FL
GPR19	-0.71	-0.38	AR-FL
CENPP	-0.65	-0.38	AR-FL
SCLT1	-0.84	-0.38	AR-FL
RAPGEF6	-0.71	-0.38	AR-FL
GLTPD2	-0.67	-0.38	AR-FL
FAM53B	-0.60	-0.38	AR-FL
APP	-0.90	-0.67	Both Isoforms
FMN2	-1.75	-0.67	Both Isoforms
GPR150	-1.55	-0.67	Both Isoforms
FAM71F2	-1.40	-0.67	Both Isoforms
ZADH2	-1.07	-0.67	Both Isoforms
TATDN3	-0.87	-0.67	Both Isoforms
CBLN3	-0.94	-0.67	Both Isoforms
RAMP2	-3.08	-0.67	Both Isoforms
FAM216A	-1.40	-0.67	Both Isoforms
ZNF581	-1.48	-0.67	Both Isoforms
ZNF277	-0.91	-0.67	Both Isoforms
GTPBP8	-0.68	-0.67	Both Isoforms
ZNF354C	-0.84	-0.67	Both Isoforms
ZDBF2	-1.06	-0.67	Both Isoforms
NEDD9	-0.63	-0.67	Both Isoforms
BMP2K	-1.13	-0.67	Both Isoforms
ARHGEF25	-1.49	-0.67	Both Isoforms
CC2D2A	-1.00	-0.67	Both Isoforms
DSP	-0.59	-0.66	Both Isoforms
BTN2A2	-1.29	-0.66	Both Isoforms
RORC	-1.78	-0.66	Both Isoforms
RCBTB2	-1.05	-0.66	Both Isoforms
OGFRL1	-0.98	-0.66	Both Isoforms
VWA5B1	-1.74	-0.66	Both Isoforms
SLC25A43	-0.66	-0.66	Both Isoforms
COLCA2	-2.28	-0.66	Both Isoforms
MAP7D2	-1.35	-0.66	Both Isoforms
AGPAT4	-1.80	-0.66	Both Isoforms
MSRB3	-1.37	-0.66	Both Isoforms
SHE	-0.98	-0.66	Both Isoforms
SAMD9	-1.54	-0.66	Both Isoforms
SFTPA2	-1.13	-0.66	Both Isoforms
GPR176	-1.19	-0.66	Both Isoforms
FAM110B	-1.56	-0.66	Both Isoforms
RAB40B	-1.06	-0.66	Both Isoforms
CDNF	-1.76	-0.66	Both Isoforms
GAS2	-1.46	-0.65	Both Isoforms
POLR1D	-1.08	-0.65	Both Isoforms
ADGRE5	-0.64	-0.65	Both Isoforms
STAG2	-0.87	-0.65	Both Isoforms
ZC3H6	-0.97	-0.65	Both Isoforms
RP11-111M22.2	-1.27	-0.65	Both Isoforms
RAB17	-0.83	-0.65	Both Isoforms
CMYA5	-1.67	-0.65	Both Isoforms
ANXA9	-0.68	-0.65	Both Isoforms
PLEKHG4	-0.82	-0.65	Both Isoforms

ORC6	-1.02	-0.38	AR-FL	KANK3	-1.29	-0.65	Both Isoforms
UPK3A	-1.23	-0.37	AR-FL	LETMD1	-1.31	-0.65	Both Isoforms
COLQ	-1.33	-0.37	AR-FL	SESN1	-1.30	-0.65	Both Isoforms
RFC3	-0.81	-0.37	AR-FL	PTPRT	-4.55	-0.65	Both Isoforms
PLS1	-0.92	-0.37	AR-FL	NKX2-3	-1.19	-0.65	Both Isoforms
HSPG2	-1.22	-0.37	AR-FL	OLFM2	-1.03	-0.65	Both Isoforms
PBK	-0.66	-0.37	AR-FL	FBXL4	-0.79	-0.65	Both Isoforms
ENOSF1	-0.65	-0.36	AR-FL	PCNXL4	-1.11	-0.65	Both Isoforms
LRRCC1	-0.59	-0.36	AR-FL	SIX2	-1.39	-0.65	Both Isoforms
ARRB2	-0.74	-0.36	AR-FL	BEND6	-1.76	-0.65	Both Isoforms
ESPL1	-1.19	-0.36	AR-FL	SIAE	-1.09	-0.65	Both Isoforms
UBE2T	-0.61	-0.36	AR-FL	OARD1	-0.89	-0.64	Both Isoforms
NPIPA8	-0.70	-0.36	AR-FL	CDKL2	-2.42	-0.64	Both Isoforms
RAD51AP1	-0.74	-0.36	AR-FL	PRSS23	-1.40	-0.64	Both Isoforms
RAB37	-0.95	-0.36	AR-FL	WASF1	-0.81	-0.64	Both Isoforms
TEF	-0.60	-0.36	AR-FL	LRRK1	-0.96	-0.64	Both Isoforms
BRCA2	-1.05	-0.36	AR-FL	E2F5	-0.90	-0.64	Both Isoforms
YPEL1	-1.03	-0.35	AR-FL	C5orf56	-0.75	-0.64	Both Isoforms
STARD9	-0.67	-0.35	AR-FL	TPM2	-3.19	-0.64	Both Isoforms
ARHGAP11B	-0.65	-0.35	AR-FL	LTBP4	-1.29	-0.64	Both Isoforms
TAPT1	-0.77	-0.35	AR-FL	MYO15B	-1.12	-0.64	Both Isoforms
HAUS5	-0.71	-0.35	AR-FL	TGIF1	-0.68	-0.64	Both Isoforms
PKIG	-0.85	-0.35	AR-FL	SIRPA	-1.52	-0.64	Both Isoforms
TMEM116	-0.61	-0.35	AR-FL	MRPL10	-0.82	-0.64	Both Isoforms
DGAT2	-1.18	-0.35	AR-FL	NPR2	-0.89	-0.64	Both Isoforms
TMEM106C	-0.65	-0.34	AR-FL	ATXN1	-0.83	-0.64	Both Isoforms
CARNS1	-0.71	-0.34	AR-FL	CHGB	-1.60	-0.64	Both Isoforms
LIF	-1.54	-0.34	AR-FL	ANKAR	-0.65	-0.64	Both Isoforms
GSG2	-1.01	-0.34	AR-FL	ZNF224	-1.43	-0.64	Both Isoforms
NUSAP1	-0.67	-0.34	AR-FL	APEX1	-0.81	-0.64	Both Isoforms
ATAD5	-0.88	-0.34	AR-FL	ARHGAP24	-1.18	-0.63	Both Isoforms
SULT2B1	-0.97	-0.34	AR-FL	AMZ1	-0.87	-0.63	Both Isoforms
FEN1	-0.59	-0.34	AR-FL	NPTN	-0.67	-0.63	Both Isoforms
OXTR	-0.85	-0.34	AR-FL	EXOSC5	-1.03	-0.63	Both Isoforms
TSPAN11	-1.42	-0.34	AR-FL	FAM92A1	-1.19	-0.63	Both Isoforms
CASC5	-0.83	-0.34	AR-FL	OPRK1	-4.69	-0.63	Both Isoforms
KCNS3	-0.89	-0.33	AR-FL	RAD9B	-1.26	-0.63	Both Isoforms
VDR	-0.70	-0.33	AR-FL	PGM2L1	-1.29	-0.63	Both Isoforms
MAN1C1	-1.13	-0.33	AR-FL	CAV2	-1.84	-0.63	Both Isoforms
DNMT3B	-0.59	-0.33	AR-FL	RBM43	-0.95	-0.63	Both Isoforms
MNS1	-0.86	-0.33	AR-FL	CALN1	-3.20	-0.63	Both Isoforms
NRGN	-0.88	-0.33	AR-FL	CA1	-1.48	-0.63	Both Isoforms
AUNIP	-1.38	-0.33	AR-FL	SCG5	-2.95	-0.63	Both Isoforms
POLN	-0.71	-0.33	AR-FL	ATAD3C	-1.63	-0.63	Both Isoforms
DNA2	-0.87	-0.33	AR-FL	OSBPL8	-1.07	-0.63	Both Isoforms
FAM19A2	-1.46	-0.33	AR-FL	TFAP4	-1.28	-0.63	Both Isoforms

SCAPER	-0.65	-0.32	AR-FL	EFNB3	-1.12	-0.63	Both Isoforms
UGT2B15	-3.34	-0.32	AR-FL	PLEKHH1	-0.63	-0.62	Both Isoforms
CCNF	-0.70	-0.32	AR-FL	FDXR	-0.79	-0.62	Both Isoforms
MAPRE2	-1.08	-0.32	AR-FL	CACNA1B	-1.42	-0.62	Both Isoforms
SFI1	-0.69	-0.32	AR-FL	ZNF286A	-0.90	-0.62	Both Isoforms
C1R	-1.23	-0.32	AR-FL	ABHD1	-1.35	-0.62	Both Isoforms
GAREM	-0.85	-0.32	AR-FL	DIEXF	-1.08	-0.62	Both Isoforms
NBPF26	-1.81	-0.32	AR-FL	BZW2	-0.91	-0.62	Both Isoforms
CLMN	-0.64	-0.32	AR-FL	CAMKMT	-0.94	-0.62	Both Isoforms
QDPR	-0.60	-0.32	AR-FL	ATG14	-1.01	-0.62	Both Isoforms
GFOD1	-0.80	-0.32	AR-FL	POLR1E	-1.40	-0.62	Both Isoforms
PIF1	-0.80	-0.32	AR-FL	SLC6A9	-1.24	-0.62	Both Isoforms
CDH26	-2.25	-0.32	AR-FL	ING3	-0.95	-0.62	Both Isoforms
RNF217	-1.28	-0.32	AR-FL	PSD	-1.97	-0.62	Both Isoforms
STXBP5L	-2.52	-0.32	AR-FL	KCNH1	-1.38	-0.62	Both Isoforms
GRAMD4	-0.92	-0.32	AR-FL	ANKRD63	-2.36	-0.62	Both Isoforms
RFC5	-0.78	-0.32	AR-FL	WFIKKN1	-1.26	-0.62	Both Isoforms
PRMT9	-0.61	-0.32	AR-FL	CTSF	-1.69	-0.62	Both Isoforms
PROM2	-0.78	-0.32	AR-FL	TAF1D	-1.25	-0.62	Both Isoforms
RARG	-0.63	-0.31	AR-FL	SLC16A10	-0.60	-0.62	Both Isoforms
SH3BP1	-0.82	-0.31	AR-FL	RARRES3	-0.77	-0.62	Both Isoforms
DIS3L	-0.82	-0.31	AR-FL	FAM117B	-0.89	-0.62	Both Isoforms
PSMC3IP	-0.69	-0.31	AR-FL	CCDC112	-0.73	-0.62	Both Isoforms
SGOL1	-0.62	-0.31	AR-FL	XK	-0.89	-0.62	Both Isoforms
CEP68	-0.89	-0.31	AR-FL	POLL	-1.14	-0.62	Both Isoforms
ANP32E	-0.90	-0.31	AR-FL	WDR35	-1.16	-0.62	Both Isoforms
RMI2	-1.02	-0.31	AR-FL	FSD1	-2.50	-0.62	Both Isoforms
ELFN2	-1.59	-0.31	AR-FL	CROCC	-1.31	-0.62	Both Isoforms
GFPT2	-1.06	-0.30	AR-FL	HOXA13	-1.64	-0.61	Both Isoforms
NPAS1	-0.75	-0.30	AR-FL	ANKRD65	-1.02	-0.61	Both Isoforms
TOX2	-1.08	-0.30	AR-FL	DDX60L	-0.65	-0.61	Both Isoforms
CDCA4	-0.84	-0.30	AR-FL	SEPSECS	-1.12	-0.61	Both Isoforms
RNF128	-0.99	-0.30	AR-FL	MAPK15	-2.12	-0.61	Both Isoforms
NCAPG	-0.67	-0.30	AR-FL	ATG16L2	-1.81	-0.61	Both Isoforms
RELT	-1.32	-0.30	AR-FL	NMB	-1.00	-0.61	Both Isoforms
CENPF	-0.63	-0.30	AR-FL	HDAC5	-0.90	-0.61	Both Isoforms
FAM198B	-2.85	-0.30	AR-FL	ADCK3	-0.71	-0.61	Both Isoforms
CEP128	-0.69	-0.29	AR-FL	HIST1H2AC	-0.91	-0.61	Both Isoforms
EGFL7	-0.70	-0.29	AR-FL	KIAA0040	-0.85	-0.61	Both Isoforms
THOC3	-0.64	-0.29	AR-FL	FOXO4	-0.95	-0.61	Both Isoforms
DNM3	-1.48	-0.29	AR-FL	ZNF763	-1.02	-0.61	Both Isoforms
CENPK	-0.71	-0.29	AR-FL	C1orf106	-0.70	-0.61	Both Isoforms
RAB36	-0.74	-0.29	AR-FL	PIM1	-0.71	-0.61	Both Isoforms
RRM2	-0.70	-0.29	AR-FL	ZNF239	-1.18	-0.61	Both Isoforms
PHLDA3	-0.76	-0.28	AR-FL	CDKL1	-0.97	-0.61	Both Isoforms
LRRC37B	-0.65	-0.28	AR-FL	ADAM11	-1.78	-0.61	Both Isoforms

CDR2L	-0.84	-0.28	AR-FL
STXBP1	-0.75	-0.28	AR-FL
GLMN	-0.77	-0.28	AR-FL
PTPRU	-0.59	-0.28	AR-FL
RAB19	-0.67	-0.27	AR-FL
ATP4B	-0.99	-0.27	AR-FL
BNIPL	-0.77	-0.27	AR-FL
CHEK1	-0.77	-0.27	AR-FL
RPAP2	-0.62	-0.27	AR-FL
TMEM266	-0.62	-0.27	AR-FL
WDHD1	-0.77	-0.27	AR-FL
STAG3	-0.69	-0.27	AR-FL
TMC8	-0.64	-0.27	AR-FL
ZNF596	-0.69	-0.27	AR-FL
HS6ST2	-2.51	-0.27	AR-FL
BOC	-1.00	-0.26	AR-FL
ULBP1	-0.98	-0.26	AR-FL
TRIM45	-1.15	-0.26	AR-FL
NDUFB4	-0.96	-0.26	AR-FL
PLIN4	-1.56	-0.26	AR-FL
TLN2	-0.91	-0.26	AR-FL
TRIP13	-0.60	-0.26	AR-FL
CIT	-0.74	-0.26	AR-FL
CISH	-1.17	-0.26	AR-FL
CCNG2	-0.90	-0.26	AR-FL
IL17RB	-0.73	-0.25	AR-FL
STK32A	-0.76	-0.25	AR-FL
EME1	-0.99	-0.25	AR-FL
NEK1	-0.69	-0.25	AR-FL
CLIP3	-0.90	-0.25	AR-FL
ARHGEF39	-0.79	-0.25	AR-FL
SCNN1D	-0.74	-0.25	AR-FL
NFATC2	-1.37	-0.25	AR-FL
DZIP1L	-1.86	-0.24	AR-FL
CAMKMT	-0.65	-0.24	AR-FL
FDXR	-0.98	-0.24	AR-FL
ANKRD36C	-0.65	-0.24	AR-FL
PALM	-0.99	-0.24	AR-FL
TOP2A	-1.01	-0.24	AR-FL
RFC4	-0.70	-0.24	AR-FL
NUF2	-0.75	-0.23	AR-FL
DONSON	-0.59	-0.23	AR-FL
SNAI1	-1.36	-0.23	AR-FL
ELF3	-0.65	-0.23	AR-FL
PTP4A3	-0.59	-0.23	AR-FL
EIF4E3	-1.22	-0.23	AR-FL

CARF	-1.36	-0.61	Both Isoforms
AVPR1B	-0.99	-0.61	Both Isoforms
GALNT14	-1.82	-0.61	Both Isoforms
N4BP3	-1.85	-0.60	Both Isoforms
BCL2	-0.63	-0.60	Both Isoforms
COL4A4	-2.57	-0.60	Both Isoforms
DIAPH2	-0.89	-0.60	Both Isoforms
CCNG1	-0.64	-0.60	Both Isoforms
KNG1	-2.16	-0.60	Both Isoforms
BMP7	-1.54	-0.60	Both Isoforms
NFATC1	-0.79	-0.60	Both Isoforms
SRI	-0.68	-0.60	Both Isoforms
RFESD	-0.82	-0.60	Both Isoforms
RPUSD4	-0.96	-0.60	Both Isoforms
AMER3	-0.71	-0.60	Both Isoforms
STK26	-0.70	-0.60	Both Isoforms
DMBX1	-1.37	-0.60	Both Isoforms
LMBR1L	-1.28	-0.60	Both Isoforms
C8orf34	-1.41	-0.60	Both Isoforms
HOXC9	-1.23	-0.60	Both Isoforms
GCSAM	-1.47	-0.60	Both Isoforms
MAP3K5	-1.08	-0.60	Both Isoforms
DLX6	-1.01	-0.60	Both Isoforms
RAB11FIP4	-0.60	-0.60	Both Isoforms
MTIF2	-0.63	-0.60	Both Isoforms
AGTPBP1	-0.62	-0.60	Both Isoforms
CD302	-1.59	-0.60	Both Isoforms
CLHC1	-0.77	-0.60	Both Isoforms
TNFRSF18	-1.68	-0.60	Both Isoforms
RNF144B	-0.82	-0.60	Both Isoforms
ACAA2	-1.22	-0.60	Both Isoforms
MAP7D3	-1.51	-0.59	Both Isoforms
TSEN2	-0.84	-0.59	Both Isoforms
TRAK1	-0.79	-0.59	Both Isoforms
SIRT3	-0.75	-0.59	Both Isoforms
SLC7A11	-1.22	-0.59	Both Isoforms
COL4A3	-1.88	-0.59	Both Isoforms
TRIP6	-1.00	-0.59	Both Isoforms
LHX6	-1.51	-0.59	Both Isoforms
SLC25A24	-0.60	-0.59	Both Isoforms
FHL3	-1.02	-0.59	Both Isoforms
LARP6	-0.62	-0.59	Both Isoforms
CRBN	-0.86	-0.59	Both Isoforms
CCNDBP1	-0.76	-0.59	Both Isoforms
NPM2	-1.41	-0.59	Both Isoforms
TRIM59	-0.77	-0.59	Both Isoforms

FUS	-0.60	-0.23	AR-FL
ZGRF1	-0.92	-0.23	AR-FL
AGPAT2	-0.60	-0.23	AR-FL
PLK4	-0.99	-0.23	AR-FL
FGFR1	-0.62	-0.23	AR-FL
KIF23	-0.82	-0.23	AR-FL
MNX1	-0.93	-0.23	AR-FL
ZC3H12B	-0.86	-0.23	AR-FL
ARL6IP6	-0.67	-0.22	AR-FL
CCDC150	-0.65	-0.22	AR-FL
LRRC75A	-1.44	-0.22	AR-FL
ELN	-0.94	-0.22	AR-FL
KIF14	-0.69	-0.22	AR-FL
TCTEX1D2	-0.82	-0.22	AR-FL
SPATA33	-0.63	-0.22	AR-FL
CEP72	-0.79	-0.21	AR-FL
TMPO	-0.58	-0.21	AR-FL
ZNF724P	-0.80	-0.21	AR-FL
PEG3	-2.46	-0.21	AR-FL
TMOD2	-0.99	-0.21	AR-FL
WNK2	-1.21	-0.21	AR-FL
TRAIP	-0.81	-0.21	AR-FL
NT5DC2	-0.65	-0.21	AR-FL
NOTCH3	-1.08	-0.20	AR-FL
VMO1	-0.92	-0.20	AR-FL
NBEA	-0.66	-0.20	AR-FL
C9orf43	-0.59	-0.20	AR-FL
UBE2C	-0.67	-0.20	AR-FL
KIF2C	-0.89	-0.20	AR-FL
FAM86B2	-1.47	-0.20	AR-FL
ADRA2C	-1.42	-0.20	AR-FL
PAX1	-2.13	-0.20	AR-FL
FES	-1.45	-0.19	AR-FL
DPF3	-0.91	-0.19	AR-FL
ATG16L2	-1.34	-0.19	AR-FL
GNG13	-0.68	-0.19	AR-FL
BAMBI	-1.77	-0.19	AR-FL
MXRA8	-0.62	-0.19	AR-FL
SMO	-0.67	-0.19	AR-FL
CDH24	-1.29	-0.19	AR-FL
ZPLD1	-0.65	-0.19	AR-FL
IER5	-1.12	-0.19	AR-FL
OR2B6	-0.84	-0.18	AR-FL
MXD3	-0.68	-0.18	AR-FL
TARBP1	-0.76	-0.18	AR-FL
PITX1	-0.92	-0.18	AR-FL

F8	-1.48	-0.59	Both Isoforms
DCBLD2	-1.52	-0.59	Both Isoforms
OGDHL	-1.21	-0.59	Both Isoforms
NINL	-0.87	-0.59	Both Isoforms
GPC6	-1.92	-0.59	Both Isoforms
ABHD18	-0.74	-0.59	Both Isoforms
NPAS1	-2.26	-0.59	Both Isoforms
HS6ST2	-1.49	-0.59	Both Isoforms
TSNARE1	-1.43	-0.58	Both Isoforms
MYO5B	-0.61	-0.58	Both Isoforms
OTX1	-0.74	-0.58	Both Isoforms
ZKSCAN7	-1.29	-0.58	Both Isoforms
KIAA1328	-0.99	-0.58	Both Isoforms
SUMF2	-0.94	-0.58	Both Isoforms
MOB3C	-0.80	-0.58	Both Isoforms
ZC2HC1A	-0.83	-0.58	Both Isoforms
RBBP9	-0.99	-0.58	AR-FL
FAM60A	-0.69	-0.58	AR-FL
CCDC102A	-1.19	-0.58	AR-FL
CBLB	-0.60	-0.58	AR-FL
SMAD7	-0.83	-0.58	AR-FL
ATM	-1.10	-0.58	AR-FL
IRS2	-1.53	-0.58	AR-FL
ARG2	-1.13	-0.58	AR-FL
TSPAN7	-1.09	-0.58	AR-FL
HDAC9	-1.27	-0.58	AR-FL
CCDC77	-0.80	-0.58	AR-FL
ANKRD6	-0.79	-0.58	AR-FL
PDPR	-0.91	-0.58	AR-FL
FGD3	-0.98	-0.58	AR-FL
CHRNB1	-0.87	-0.58	AR-FL
TPPP	-1.35	-0.58	AR-FL
PRR16	-2.68	-0.58	AR-FL
RSL24D1	-0.85	-0.57	AR-FL
VSTM5	-0.87	-0.57	AR-FL
PLTP	-1.43	-0.57	AR-FL
RPAP2	-0.68	-0.57	AR-FL
ADAT2	-1.40	-0.57	AR-FL
GKAP1	-0.64	-0.57	AR-FL
TSPAN2	-2.09	-0.57	AR-FL
ZNF251	-1.05	-0.57	AR-FL
FAS	-1.23	-0.57	AR-FL
NOS3	-1.00	-0.57	AR-FL
RNF43	-0.83	-0.57	AR-FL
ZFP14	-0.94	-0.57	AR-FL
HIST3H2A	-0.93	-0.57	AR-FL

AK7	-0.66	-0.18	AR-FL	SERP2	-1.68	-0.57	AR-FL
DPF1	-0.85	-0.18	AR-FL	ZFP2	-1.04	-0.57	AR-FL
DNM1	-0.63	-0.18	AR-FL	EPS8	-1.01	-0.57	AR-FL
EXTL1	-2.21	-0.18	AR-FL	PPP1R9A	-0.99	-0.57	AR-FL
RPS17	-1.35	-0.18	AR-FL	C2orf70	-1.72	-0.57	AR-FL
POU2F3	-0.78	-0.18	AR-FL	TMTC1	-1.84	-0.57	AR-FL
MGME1	-0.62	-0.17	AR-FL	EDA	-2.28	-0.57	AR-FL
SPG20	-1.67	-0.17	AR-FL	SEMA6B	-1.75	-0.56	AR-FL
KIAA0922	-0.59	-0.17	AR-FL	BTN3A1	-1.28	-0.56	AR-FL
HMGCS2	-0.62	-0.17	AR-FL	BCAS4	-1.59	-0.56	AR-FL
FSCN1	-1.13	-0.17	AR-FL	SOX13	-1.90	-0.56	AR-FL
KLLN	-0.89	-0.17	AR-FL	DDC	-2.01	-0.56	AR-FL
NABP1	-0.61	-0.17	AR-FL	C14orf159	-1.10	-0.56	AR-FL
FBXO44	-0.83	-0.17	AR-FL	MRPL45	-0.60	-0.56	AR-FL
NAP1L1	-0.80	-0.17	AR-FL	ZNF343	-1.05	-0.56	AR-FL
FCGBP	-0.76	-0.17	AR-FL	KIAA1147	-0.86	-0.56	AR-FL
MAN2A2	-0.73	-0.17	AR-FL	IMPACT	-0.76	-0.56	AR-FL
ATP8A1	-0.93	-0.17	AR-FL	ZDHHC17	-0.59	-0.56	AR-FL
ATP6AP1L	-0.88	-0.16	AR-FL	SPATA7	-0.95	-0.56	AR-FL
TNIK	-0.80	-0.16	AR-FL	TIMP2	-1.13	-0.56	AR-FL
MMP11	-0.66	-0.16	AR-FL	LANCL1	-0.82	-0.56	AR-FL
INPP5B	-0.62	-0.16	AR-FL	KCNK5	-0.94	-0.56	AR-FL
ARHGAP19	-0.60	-0.16	AR-FL	TTC36	-0.98	-0.56	AR-FL
KIF3C	-0.70	-0.16	AR-FL	BACH1	-0.73	-0.56	AR-FL
CDCA3	-0.73	-0.16	AR-FL	PSD4	-1.03	-0.56	AR-FL
CENPE	-0.68	-0.15	AR-FL	RIMBP2	-1.24	-0.56	AR-FL
CDCA8	-0.64	-0.15	AR-FL	TRIM23	-0.85	-0.56	AR-FL
NES	-1.03	-0.15	AR-FL	HTRA2	-0.62	-0.56	AR-FL
CCNB2	-0.70	-0.15	AR-FL	ZNF516	-0.90	-0.56	AR-FL
ROBO1	-0.95	-0.15	AR-FL	TMEM116	-0.89	-0.56	AR-FL
ZNF703	-0.82	-0.15	AR-FL	PRCP	-0.98	-0.56	AR-FL
FAM57B	-0.65	-0.15	AR-FL	RGAG4	-0.78	-0.56	AR-FL
C2orf81	-0.86	-0.15	AR-FL	AVPI1	-0.83	-0.56	AR-FL
DIAPH3	-0.79	-0.14	AR-FL	MATR3	-0.90	-0.56	AR-FL
SCAI	-0.59	-0.14	AR-FL	MATR3	-0.67	-0.56	AR-FL
MAGI3	-0.83	-0.14	AR-FL	GSAP	-0.97	-0.56	AR-FL
ACACB	-0.82	-0.14	AR-FL	ZMAT1	-2.47	-0.56	AR-FL
PSIP1	-0.58	-0.14	AR-FL	BCL2L13	-0.81	-0.56	AR-FL
RASL10B	-2.33	-0.14	AR-FL	EPHA8	-1.83	-0.56	AR-FL
CLMP	-0.90	-0.14	AR-FL	CTTNBP2NL	-1.01	-0.56	AR-FL
ALDH1L2	-0.96	-0.13	AR-FL	SNX5	-0.95	-0.56	AR-FL
FAM102B	-1.16	-0.13	AR-FL	ARHGAP29	-0.67	-0.56	AR-FL
ALDH5A1	-0.74	-0.13	AR-FL	CD101	-0.93	-0.55	AR-FL
SIPA1L1	-0.69	-0.13	AR-FL	PCYOX1	-0.74	-0.55	AR-FL
SPTBN1	-0.76	-0.13	AR-FL	APPL1	-0.79	-0.55	AR-FL
C19orf57	-0.80	-0.13	AR-FL	ELF5	-0.92	-0.55	AR-FL

CSAD	-0.65	-0.13	AR-FL
PDIK1L	-0.61	-0.13	AR-FL
BMP6	-0.97	-0.12	AR-FL
AMN1	-0.63	-0.12	AR-FL
PRSS23	-0.69	-0.12	AR-FL
CENPI	-0.61	-0.12	AR-FL
RCOR2	-1.30	-0.12	AR-FL
FAM101B	-1.12	-0.11	AR-FL
CCDC180	-1.35	-0.11	AR-FL
NINJ1	-0.97	-0.11	AR-FL
ENPP1	-0.66	-0.11	AR-FL
PLCE1	-0.68	-0.11	AR-FL
C3orf52	-0.66	-0.10	AR-FL
MTFR2	-0.67	-0.10	AR-FL
ZNF738	-0.92	-0.10	AR-FL
NEIL1	-0.96	-0.10	AR-FL
NAV1	-0.71	-0.10	AR-FL
RASAL2	-0.60	-0.10	AR-FL
MXI1	-0.66	-0.10	AR-FL
GDPD5	-1.07	-0.10	AR-FL
DBF4B	-0.68	-0.09	AR-FL
KLHL3	-0.75	-0.09	AR-FL
EFCC1	-1.14	-0.09	AR-FL
POLD3	-0.77	-0.09	AR-FL
TNFRSF10D	-0.77	-0.08	AR-FL
PELI2	-0.83	-0.08	AR-FL
EML5	-1.10	-0.08	AR-FL
PCOLCE	-1.03	-0.08	AR-FL
TCEA3	-0.64	-0.08	AR-FL
MYOM2	-0.66	-0.08	AR-FL
TTYH2	-0.88	-0.08	AR-FL
LRRC49	-0.78	-0.07	AR-FL
RTTN	-0.93	-0.07	AR-FL
PARD6A	-0.70	-0.07	AR-FL
OSBPL3	-0.62	-0.07	AR-FL
CLUL1	-1.12	-0.06	AR-FL
C14orf93	-0.63	-0.06	AR-FL
CBX2	-0.63	-0.06	AR-FL
KIAA0895	-0.66	-0.06	AR-FL
SORBS3	-0.59	-0.06	AR-FL
SLC2A11	-0.58	-0.06	AR-FL
GOLGA8A	-0.59	-0.06	AR-FL
PROCR	-0.93	-0.06	AR-FL
SYT15	-0.77	-0.05	AR-FL
THUMPD2	-0.59	-0.05	AR-FL
ANO9	-0.68	-0.55	AR-FL
RP11-1212A22.4	-0.66	-0.55	AR-FL
OPRL1	-0.92	-0.55	AR-FL
CATSPERG	-0.82	-0.55	AR-FL
ZNF546	-1.18	-0.55	AR-FL
JRKL	-1.11	-0.55	AR-FL
SLC25A36	-0.81	-0.55	AR-FL
GPR135	-0.99	-0.55	AR-FL
GHR	-1.58	-0.55	AR-FL
SNED1	-0.64	-0.55	AR-FL
ATP2C1	-0.69	-0.55	AR-FL
OAF	-1.13	-0.55	AR-FL
ZNF25	-1.18	-0.55	AR-FL
TBC1D1	-0.89	-0.55	AR-FL
C3orf17	-1.08	-0.55	AR-FL
CD6	-2.37	-0.55	AR-FL
ADAM7	-2.52	-0.55	AR-FL
PLEKHG1	-1.74	-0.55	AR-FL
PTCHD2	-0.67	-0.55	AR-FL
ZNF596	-0.68	-0.55	AR-FL
ZBTB41	-0.87	-0.55	AR-FL
RSBN1	-0.65	-0.54	AR-FL
METAP1D	-0.62	-0.54	AR-FL
ZFYVE9	-0.71	-0.54	AR-FL
HEG1	-2.17	-0.54	AR-FL
C15orf41	-0.64	-0.54	AR-FL
CBFA2T2	-0.58	-0.54	AR-FL
KCNIP4	-1.32	-0.54	AR-FL
MED14	-0.61	-0.54	AR-FL
PLEKHN1	-0.88	-0.54	AR-FL
IGDCC4	-1.53	-0.54	AR-FL
ITGB4	-0.83	-0.54	AR-FL
C14orf93	-1.61	-0.54	AR-FL
AKR1E2	-0.93	-0.54	AR-FL
TMEM62	-1.02	-0.54	AR-FL
ING4	-0.71	-0.54	AR-FL
LMO4	-1.34	-0.54	AR-FL
TOP1MT	-1.18	-0.54	AR-FL
MYH10	-0.85	-0.54	AR-FL
ZNF283	-0.76	-0.54	AR-FL
ATP6AP1L	-1.45	-0.54	AR-FL
SSBP2	-1.44	-0.53	AR-FL
METTL8	-0.81	-0.53	AR-FL
EIF3E	-0.98	-0.53	AR-FL
NLE1	-1.02	-0.53	AR-FL

IRX5	-1.53	-0.05	AR-FL	WDR59	-1.04	-0.53	AR-FL
RAB6B	-0.91	-0.05	AR-FL	LRRN2	-3.20	-0.53	AR-FL
C2orf15	-0.64	-0.05	AR-FL	UBE3D	-0.96	-0.53	AR-FL
SUGCT	-0.76	-0.05	AR-FL	L1CAM	-1.38	-0.53	AR-FL
C16orf96	-0.90	-0.05	AR-FL	LRPPRC	-0.88	-0.53	AR-FL
TSPAN3	-0.89	-0.04	AR-FL	THADA	-0.78	-0.53	AR-FL
DET1	-0.59	-0.04	AR-FL	KCTD18	-0.62	-0.53	AR-FL
SORBS1	-0.82	-0.04	AR-FL	PALM2	-1.75	-0.53	AR-FL
TMEM65	-0.59	-0.04	AR-FL	TEKT4	-1.84	-0.53	AR-FL
FBXO15	-0.85	-0.04	AR-FL	ASS1	-1.09	-0.53	AR-FL
PLEKHD1	-0.88	-0.04	AR-FL	PLEKHB1	-1.29	-0.53	AR-FL
ZCWPW1	-0.62	-0.04	AR-FL	TCEAL1	-0.68	-0.53	AR-FL
CEP126	-0.62	-0.04	AR-FL	C12orf66	-1.05	-0.53	AR-FL
KDM4D	-0.62	-0.03	AR-FL	SCARA3	-0.86	-0.53	AR-FL
FHL3	-0.61	-0.03	AR-FL	GCA	-1.01	-0.53	AR-FL
BRIP1	-0.99	-0.03	AR-FL	NR6A1	-1.15	-0.53	AR-FL
HJURP	-0.74	-0.02	AR-FL	PYGM	-1.51	-0.53	AR-FL
KCNIP3	-1.04	-0.02	AR-FL	USP54	-1.13	-0.53	AR-FL
HIST1H2BO	-0.58	-0.02	AR-FL	NPHP3	-0.68	-0.53	AR-FL
TCFL5	-0.63	-0.02	AR-FL	MLANA	-0.59	-0.53	AR-FL
KIF24	-0.76	-0.02	AR-FL	EIF3D	-0.73	-0.53	AR-FL
SSTR2	-0.63	-0.02	AR-FL	SOX18	-1.51	-0.52	AR-FL
FAM78A	-0.84	-0.02	AR-FL	UTP14A	-0.87	-0.52	AR-FL
LTBP1	-1.39	-0.02	AR-FL	ZFP36L2	-1.35	-0.52	AR-FL
ASPM	-0.62	-0.02	AR-FL	MCMDC2	-1.47	-0.52	AR-FL
CDK1	-0.83	-0.02	AR-FL	SOCS7	-0.59	-0.52	AR-FL
QSER1	-1.01	-0.02	AR-FL	FKTN	-0.83	-0.52	AR-FL
RASL11B	-2.21	-0.01	AR-FL	AMHR2	-1.66	-0.52	AR-FL
CYP2W1	-1.14	-0.01	AR-FL	SLC25A34	-1.16	-0.52	AR-FL
SUV420H2	-0.63	-0.01	AR-FL	EIF4A2	-0.69	-0.52	AR-FL
GALK1	-0.94	-0.01	AR-FL	UBXN7	-0.88	-0.52	AR-FL
C5orf45	-0.60	-0.01	AR-FL	BBS7	-0.93	-0.52	AR-FL
TEX22	-0.59	-0.01	AR-FL	PLCH2	-1.54	-0.52	AR-FL
VASH2	-0.65	-0.01	AR-FL	NRXN2	-1.37	-0.52	AR-FL
SMAD3	-1.28	0.00	AR-FL	ANXA2R	-1.10	-0.52	AR-FL
TXNIP	-0.93	0.00	AR-FL	BLOC1S6	-0.62	-0.52	AR-FL
SSBP3	-0.86	0.00	AR-FL	SNX15	-1.00	-0.52	AR-FL
OPN3	-0.58	0.00	AR-FL	TMEM117	-1.13	-0.52	AR-FL
SPAG16	-0.33	-11.04	AR-V7	PPOX	-1.06	-0.52	AR-FL
HENMT1	-0.22	-6.26	AR-V7	C5orf47	-0.76	-0.52	AR-FL
TMSB4X	-0.53	-4.04	AR-V7	ZP3	-0.94	-0.52	AR-FL
VIPR2	-0.22	-3.72	AR-V7	RHOBTB1	-1.99	-0.52	AR-FL
SAMD5	-0.38	-3.18	AR-V7	CCNB1IP1	-1.36	-0.52	AR-FL
SHC3	-0.55	-3.10	AR-V7	KCND3	-0.61	-0.52	AR-FL
ANKRD65	-0.43	-3.04	AR-V7	SMKR1	-1.80	-0.51	AR-FL
TNFRSF14	-0.01	-3.00	AR-V7	EIF2S3L	-0.72	-0.51	AR-FL

SLC40A1	-0.31	-2.98	AR-V7
SYNE1	-0.54	-2.97	AR-V7
CNNM1	-0.19	-2.90	AR-V7
CADM1	-0.47	-2.56	AR-V7
CNTN4	-0.35	-2.46	AR-V7
GDPD1	-0.58	-2.40	AR-V7
APOL6	-0.28	-2.20	AR-V7
SLC7A11	-0.22	-2.18	AR-V7
ATXN1	-0.23	-2.09	AR-V7
PGBD5	-0.24	-2.02	AR-V7
GPRIN2	-0.57	-2.00	AR-V7
CDH13	-0.20	-2.00	AR-V7
GPX2	-0.43	-1.99	AR-V7
PDGFA	-0.43	-1.97	AR-V7
LONRF2	-0.31	-1.91	AR-V7
SORBS2	-0.16	-1.89	AR-V7
GIMAP2	-0.42	-1.84	AR-V7
B4GALNT3	-0.08	-1.82	AR-V7
BANK1	-0.54	-1.81	AR-V7
MME	-0.17	-1.77	AR-V7
SLC30A4	-0.26	-1.77	AR-V7
QKI	-0.19	-1.76	AR-V7
LRFN2	-0.43	-1.73	AR-V7
PLPP2	-0.58	-1.71	AR-V7
PARP10	-0.43	-1.70	AR-V7
SYT7	-0.08	-1.60	AR-V7
CCDC122	0.00	-1.60	AR-V7
KIAA2022	-0.07	-1.59	AR-V7
RGS11	-0.45	-1.58	AR-V7
BCAS1	-0.34	-1.54	AR-V7
SMPDL3B	-0.46	-1.52	AR-V7
CDC42EP1	-0.30	-1.52	AR-V7
DSC2	-0.40	-1.52	AR-V7
TMEM40	-0.33	-1.51	AR-V7
PLCG2	-0.51	-1.49	AR-V7
ZC3H12C	-0.56	-1.49	AR-V7
SV2C	-0.37	-1.48	AR-V7
FOXN3	-0.11	-1.47	AR-V7
PDIA2	-0.57	-1.47	AR-V7
RTN1	-0.21	-1.45	AR-V7
LRBA	-0.52	-1.43	AR-V7
LARGE	-0.18	-1.41	AR-V7
RLTPR	-0.36	-1.37	AR-V7
PAM	-0.15	-1.37	AR-V7
CGNL1	-0.21	-1.37	AR-V7
SLC39A8	-0.55	-1.34	AR-V7

MARK1	-0.88	-0.51	AR-FL
PABPC4	-0.79	-0.51	AR-FL
MACROD2	-1.47	-0.51	AR-FL
TATDN1	-0.79	-0.51	AR-FL
ZFP90	-1.18	-0.51	AR-FL
SLC7A2	-0.65	-0.51	AR-FL
ARID5A	-1.21	-0.51	AR-FL
TRMT5	-1.17	-0.51	AR-FL
ZNF285	-0.78	-0.51	AR-FL
SPNS2	-1.19	-0.51	AR-FL
ZNF383	-1.19	-0.51	AR-FL
ABLIM1	-0.66	-0.51	AR-FL
IRAK1BP1	-1.38	-0.51	AR-FL
HTR6	-1.31	-0.51	AR-FL
RFX7	-0.67	-0.51	AR-FL
KIF3C	-0.72	-0.51	AR-FL
SLC25A6	-0.95	-0.51	AR-FL
KDSR	-0.93	-0.51	AR-FL
GOLGA8A	-1.52	-0.51	AR-FL
HSD17B11	-1.04	-0.51	AR-FL
DDN	-1.53	-0.51	AR-FL
TBC1D5	-0.77	-0.51	AR-FL
FBXO4	-0.99	-0.51	AR-FL
STK33	-1.22	-0.51	AR-FL
CRMP1	-1.37	-0.51	AR-FL
SLC12A8	-0.59	-0.51	AR-FL
ASB11	-1.01	-0.50	AR-FL
TGFBR3	-1.47	-0.50	AR-FL
CPNE3	-0.70	-0.50	AR-FL
SLC26A10	-2.38	-0.50	AR-FL
LRRC8D	-0.60	-0.50	AR-FL
UNC93B1	-1.18	-0.50	AR-FL
FLNB	-0.61	-0.50	AR-FL
NRIP2	-0.63	-0.50	AR-FL
PDE5A	-0.83	-0.50	AR-FL
SOBP	-2.54	-0.50	AR-FL
GABRA5	-1.90	-0.50	AR-FL
DENND5A	-0.73	-0.50	AR-FL
HRSP12	-1.26	-0.50	AR-FL
NUP133	-0.71	-0.50	AR-FL
LNP1	-0.77	-0.50	AR-FL
JAKMIP3	-0.67	-0.50	AR-FL
CCDC27	-2.17	-0.50	AR-FL
NFASC	-1.64	-0.50	AR-FL
NTPCR	-0.93	-0.50	AR-FL
SNAI1	-0.86	-0.50	AR-FL

MFSD6L	-0.30	-1.32	AR-V7
NXPH4	-0.16	-1.32	AR-V7
CDK14	-0.12	-1.32	AR-V7
DSE	-0.48	-1.31	AR-V7
SLC9A9	-0.14	-1.31	AR-V7
CBR1	-0.13	-1.31	AR-V7
SATB2	-0.57	-1.29	AR-V7
NQO1	-0.09	-1.28	AR-V7
ENOX1	-0.27	-1.27	AR-V7
BICD1	-0.12	-1.27	AR-V7
SLC35G1	-0.49	-1.27	AR-V7
PLXNA2	-0.08	-1.25	AR-V7
ASPHD1	-0.11	-1.25	AR-V7
PELI1	-0.36	-1.24	AR-V7
KIAA1024	-0.52	-1.23	AR-V7
KCNQ2	-0.47	-1.21	AR-V7
PPARG	-0.38	-1.21	AR-V7
CTSC	-0.29	-1.19	AR-V7
DCHS1	-0.28	-1.18	AR-V7
SLC13A3	-0.27	-1.18	AR-V7
RASEF	-0.42	-1.17	AR-V7
PITPNC1	-0.41	-1.17	AR-V7
CXXC5	-0.53	-1.14	AR-V7
KLF8	-0.35	-1.14	AR-V7
FAXC	-0.57	-1.13	AR-V7
SLC23A1	-0.10	-1.12	AR-V7
KIF1A	-0.54	-1.12	AR-V7
PLCB1	-0.43	-1.11	AR-V7
CNTNAP3	-0.23	-1.11	AR-V7
PPP2R2C	-0.47	-1.11	AR-V7
AEBP1	-0.25	-1.10	AR-V7
BLOC1S6	-0.31	-1.09	AR-V7
PLK2	-0.20	-1.09	AR-V7
NPTXR	-0.01	-1.08	AR-V7
SLC6A6	-0.52	-1.07	AR-V7
FTCDNL1	-0.34	-1.07	AR-V7
HIVEP3	-0.42	-1.06	AR-V7
SLC16A1	-0.09	-1.05	AR-V7
STAMBPL1	-0.18	-1.05	AR-V7
NETO1	-0.48	-1.04	AR-V7
COL26A1	-0.17	-1.03	AR-V7
PIM1	-0.33	-1.02	AR-V7
APBB2	-0.17	-1.02	AR-V7
SERAC1	-0.43	-1.02	AR-V7
VEGFB	-0.04	-1.02	AR-V7
AP3B2	-0.43	-1.01	AR-V7
TRIM21	-0.76	-0.50	AR-FL
ERBB2	-1.11	-0.50	AR-FL
VAT1	-0.68	-0.50	AR-FL
RBMX2	-0.72	-0.50	AR-FL
FAM120C	-1.18	-0.50	AR-FL
PTAR1	-0.73	-0.50	AR-FL
FAXDC2	-1.31	-0.50	AR-FL
PATZ1	-0.88	-0.50	AR-FL
MLXIPL	-1.41	-0.49	AR-FL
TMEM245	-0.83	-0.49	AR-FL
ZNF395	-1.21	-0.49	AR-FL
AIFM3	-0.75	-0.49	AR-FL
FAM198B	-1.07	-0.49	AR-FL
FSCN1	-1.50	-0.49	AR-FL
ZSCAN30	-0.80	-0.49	AR-FL
LRRC34	-0.62	-0.49	AR-FL
LPCAT2	-0.91	-0.49	AR-FL
C5orf45	-1.43	-0.49	AR-FL
DNAH10	-0.61	-0.49	AR-FL
DPY19L4	-0.78	-0.49	AR-FL
ZNF514	-1.03	-0.49	AR-FL
TMEM128	-0.77	-0.49	AR-FL
C1R	-0.98	-0.49	AR-FL
MMP11	-0.69	-0.49	AR-FL
RASGEF1C	-2.07	-0.49	AR-FL
CCDC88A	-1.74	-0.49	AR-FL
ANKRD13B	-1.06	-0.49	AR-FL
ATXN3	-0.64	-0.49	AR-FL
MEX3A	-0.69	-0.49	AR-FL
CA11	-2.53	-0.49	AR-FL
C16orf62	-0.94	-0.49	AR-FL
TP53	-1.03	-0.49	AR-FL
ZNRF1	-0.61	-0.49	AR-FL
BLOC1S5	-0.67	-0.49	AR-FL
LGI2	-1.92	-0.49	AR-FL
USP27X	-1.05	-0.49	AR-FL
ARRDC2	-0.87	-0.49	AR-FL
PCBP2	-0.69	-0.49	AR-FL
GAB2	-1.04	-0.49	AR-FL
AEBP2	-0.64	-0.48	AR-FL
ZNF362	-0.80	-0.48	AR-FL
LOXL3	-1.60	-0.48	AR-FL
WDR3	-0.69	-0.48	AR-FL
NUDT6	-1.24	-0.48	AR-FL
EIF2A	-0.68	-0.48	AR-FL
NFAT5	-1.04	-0.48	AR-FL

STEAP3	-0.27	-1.01	AR-V7	TTK	-0.97	-0.48	AR-FL
PPP2R2A	-0.57	-1.00	AR-V7	DYRK2	-0.72	-0.48	AR-FL
SLC9A3R1	-0.21	-1.00	AR-V7	DIS3L	-1.08	-0.48	AR-FL
ISX	-0.57	-1.00	AR-V7	HMG20A	-0.74	-0.48	AR-FL
UQCC1	-0.35	-1.00	AR-V7	MEIS1	-2.37	-0.48	AR-FL
STAC3	-0.29	-1.00	AR-V7	ZKSCAN8	-1.13	-0.48	AR-FL
RAB23	-0.25	-0.98	AR-V7	DNM1	-1.17	-0.48	AR-FL
C17orf51	-0.29	-0.98	AR-V7	HSPG2	-1.27	-0.48	AR-FL
MBP	-0.02	-0.97	AR-V7	PRIM1	-1.22	-0.48	AR-FL
CEBPA	-0.54	-0.97	AR-V7	BRWD3	-0.85	-0.48	AR-FL
TRIM7	-0.38	-0.97	AR-V7	ADAMTS13	-1.60	-0.48	AR-FL
CCDC67	-0.29	-0.96	AR-V7	KLC4	-0.69	-0.48	AR-FL
FLRT1	-0.48	-0.96	AR-V7	GABPB2	-1.15	-0.48	AR-FL
BOK	-0.26	-0.96	AR-V7	RMND1	-1.05	-0.48	AR-FL
XPO5	-0.20	-0.96	AR-V7	UNC13D	-1.96	-0.48	AR-FL
DHRS13	-0.23	-0.96	AR-V7	HEBP1	-0.83	-0.48	AR-FL
MTHFD2	-0.54	-0.95	AR-V7	EFCAB13	-1.63	-0.48	AR-FL
CDKN2C	-0.57	-0.95	AR-V7	CTH	-0.81	-0.48	AR-FL
EIF4EBP1	-0.32	-0.95	AR-V7	ZNF510	-0.68	-0.48	AR-FL
EPHB3	-0.17	-0.95	AR-V7	ADGRV1	-1.13	-0.48	AR-FL
NCAM2	-0.10	-0.94	AR-V7	NT5M	-1.97	-0.47	AR-FL
STBD1	-0.57	-0.94	AR-V7	VAMP7	-1.01	-0.47	AR-FL
ANKRD44	-0.45	-0.94	AR-V7	ZDHHC14	-0.60	-0.47	AR-FL
TPD52L1	-0.01	-0.94	AR-V7	DET1	-0.80	-0.47	AR-FL
TUSC1	-0.52	-0.93	AR-V7	LONRF2	-1.00	-0.47	AR-FL
SLC25A21	-0.39	-0.93	AR-V7	CLNS1A	-0.85	-0.47	AR-FL
TMTC4	-0.13	-0.93	AR-V7	DSTYK	-0.67	-0.47	AR-FL
ABLIM1	-0.54	-0.92	AR-V7	LYRM9	-1.37	-0.47	AR-FL
ITGA6	-0.06	-0.92	AR-V7	SHF	-1.26	-0.47	AR-FL
CBFA2T2	-0.08	-0.91	AR-V7	RSL1D1	-0.84	-0.47	AR-FL
TTC39B	-0.57	-0.91	AR-V7	CTSC	-0.92	-0.47	AR-FL
SNTA1	-0.35	-0.90	AR-V7	SETD9	-0.66	-0.47	AR-FL
SETBP1	-0.09	-0.90	AR-V7	GSPT2	-0.60	-0.47	AR-FL
MAPK13	-0.33	-0.90	AR-V7	LEF1	-0.68	-0.47	AR-FL
PDE3B	-0.12	-0.90	AR-V7	KIF17	-0.89	-0.47	AR-FL
AVEN	-0.17	-0.89	AR-V7	TDRD5	-0.66	-0.47	AR-FL
TTC34	-0.57	-0.89	AR-V7	EVC	-1.16	-0.47	AR-FL
PTK6	-0.47	-0.89	AR-V7	KLF11	-0.87	-0.47	AR-FL
ACOX2	-0.12	-0.88	AR-V7	PXK	-0.80	-0.47	AR-FL
ARHGAP18	-0.49	-0.88	AR-V7	POT1	-0.71	-0.47	AR-FL
GARS	-0.35	-0.88	AR-V7	MTR	-1.12	-0.47	AR-FL
AHI1	-0.15	-0.88	AR-V7	CLCN5	-0.66	-0.47	AR-FL
SLCO4A1	-0.28	-0.88	AR-V7	FAIM	-0.60	-0.47	AR-FL
GPHN	-0.23	-0.88	AR-V7	ZNF566	-0.75	-0.47	AR-FL
NIPAL2	-0.23	-0.88	AR-V7	GMCL1	-0.72	-0.47	AR-FL
IFI35	-0.57	-0.87	AR-V7	ALOX12B	-0.60	-0.47	AR-FL

SHMT2	-0.02	-0.87	AR-V7
FAM3B	-0.46	-0.86	AR-V7
CGN	-0.27	-0.86	AR-V7
UBE3D	-0.19	-0.86	AR-V7
REXO2	-0.39	-0.86	AR-V7
ITGA3	-0.57	-0.86	AR-V7
SHB	-0.02	-0.86	AR-V7
KDM6A	-0.47	-0.86	AR-V7
MAST4	-0.20	-0.85	AR-V7
PCDHGA9	-0.46	-0.84	AR-V7
YPEL2	-0.46	-0.84	AR-V7
DTNB	-0.38	-0.84	AR-V7
NCOR2	-0.41	-0.84	AR-V7
MDN1	-0.13	-0.84	AR-V7
RTEL1-TNFRSF6B	-0.35	-0.83	AR-V7
SH3RF3	-0.22	-0.83	AR-V7
IFIH1	-0.20	-0.82	AR-V7
IMMP2L	-0.39	-0.81	AR-V7
ASAP3	-0.42	-0.81	AR-V7
TMEM238	-0.48	-0.80	AR-V7
HGH1	-0.07	-0.80	AR-V7
SAMD13	-0.52	-0.80	AR-V7
PANK1	-0.46	-0.80	AR-V7
TUBA1B	-0.22	-0.80	AR-V7
TRIM25	-0.56	-0.80	AR-V7
DST	-0.53	-0.79	AR-V7
TRAPPC9	-0.35	-0.79	AR-V7
MTHFD1L	-0.29	-0.79	AR-V7
RAB9B	-0.25	-0.79	AR-V7
GSTA4	-0.26	-0.78	AR-V7
SLC4A3	-0.45	-0.78	AR-V7
ADK	-0.25	-0.78	AR-V7
NETO2	-0.52	-0.78	AR-V7
ATIC	-0.42	-0.78	AR-V7
PDSS2	-0.40	-0.77	AR-V7
ZMYND11	-0.45	-0.77	AR-V7
PFAS	-0.47	-0.77	AR-V7
FAM47E	-0.53	-0.77	AR-V7
RAB9A	-0.20	-0.76	AR-V7
RBPMS2	-0.33	-0.76	AR-V7
STAP2	-0.23	-0.76	AR-V7
THBS4	-0.52	-0.76	AR-V7
ARHGEF6	-0.51	-0.76	AR-V7
NMU	-0.13	-0.75	AR-V7
KCTD15	-0.47	-0.75	AR-V7
PNP	-0.22	-0.75	AR-V7

C12orf74	-2.58	-0.47	AR-FL
ZNF862	-1.13	-0.47	AR-FL
WASL	-0.64	-0.47	AR-FL
C2CD5	-0.87	-0.47	AR-FL
CFD	-0.69	-0.47	AR-FL
MGAT3	-1.88	-0.47	AR-FL
RASIP1	-1.26	-0.47	AR-FL
PRDM11	-0.68	-0.46	AR-FL
RGL1	-1.14	-0.46	AR-FL
MKS1	-0.83	-0.46	AR-FL
ZSWIM5	-0.82	-0.46	AR-FL
ZSCAN23	-1.56	-0.46	AR-FL
RCN3	-1.04	-0.46	AR-FL
PFKM	-0.92	-0.46	AR-FL
PTPN4	-0.75	-0.46	AR-FL
MRPS31	-0.73	-0.46	AR-FL
ATF4	-0.83	-0.46	AR-FL
LOXL1	-0.98	-0.46	AR-FL
BZRAP1	-2.00	-0.46	AR-FL
TXNRD3	-1.00	-0.46	AR-FL
IRF8	-2.65	-0.46	AR-FL
WDR12	-0.86	-0.46	AR-FL
FREM1	-1.90	-0.46	AR-FL
MRE11A	-0.94	-0.46	AR-FL
TRPC7	-0.83	-0.46	AR-FL
MYO1B	-0.82	-0.46	AR-FL
ANKRD36B	-1.31	-0.46	AR-FL
C3orf18	-0.86	-0.46	AR-FL
PDE6B	-1.76	-0.46	AR-FL
COCH	-0.80	-0.46	AR-FL
GAL3ST2	-1.38	-0.46	AR-FL
MFSD7	-1.25	-0.46	AR-FL
ALDH5A1	-1.19	-0.45	AR-FL
KIAA1551	-0.59	-0.45	AR-FL
NEIL1	-1.52	-0.45	AR-FL
ATP6V1E2	-0.84	-0.45	AR-FL
RARA	-1.02	-0.45	AR-FL
FTCDNL1	-0.86	-0.45	AR-FL
SHISA2	-0.85	-0.45	AR-FL
FAM228B	-1.57	-0.45	AR-FL
GRIK5	-1.01	-0.45	AR-FL
ZNF214	-1.43	-0.45	AR-FL
RHBDF2	-1.15	-0.45	AR-FL
SYDE1	-1.12	-0.45	AR-FL
CYB5RL	-1.09	-0.45	AR-FL
ENPP4	-0.62	-0.45	AR-FL

FAM213B	-0.50	-0.75	AR-V7
2-Mar	-0.37	-0.75	AR-V7
CTNNAL1	-0.38	-0.75	AR-V7
PRDX1	-0.51	-0.75	AR-V7
PHF12	-0.37	-0.75	AR-V7
TCF7	-0.37	-0.75	AR-V7
SCPEP1	-0.30	-0.75	AR-V7
CACYBP	-0.46	-0.75	AR-V7
WASF3	-0.40	-0.75	AR-V7
PDCD2	-0.17	-0.75	AR-V7
ARHGAP32	-0.14	-0.74	AR-V7
NFU1	-0.16	-0.74	AR-V7
CCDC154	-0.46	-0.74	AR-V7
TTC28	-0.28	-0.74	AR-V7
CMTM7	-0.53	-0.74	AR-V7
MBNL3	-0.41	-0.74	AR-V7
CEP57L1	-0.22	-0.74	AR-V7
USP45	-0.25	-0.74	AR-V7
SAMD1	-0.46	-0.74	AR-V7
C1orf115	-0.01	-0.73	AR-V7
PCMTD1	-0.25	-0.73	AR-V7
LEO1	-0.04	-0.73	AR-V7
RNF6	-0.25	-0.73	AR-V7
MSRA	-0.03	-0.73	AR-V7
G6PD	-0.19	-0.73	AR-V7
TRAF3	-0.26	-0.73	AR-V7
NPW	-0.45	-0.73	AR-V7
PIEZO1	-0.04	-0.72	AR-V7
UHRF1BP1L	-0.08	-0.72	AR-V7
DCAKD	-0.38	-0.72	AR-V7
CERS4	-0.52	-0.72	AR-V7
ERVMER34-1	-0.34	-0.72	AR-V7
HSP90AB1	-0.25	-0.72	AR-V7
RBL2	-0.22	-0.71	AR-V7
SMC2	-0.50	-0.71	AR-V7
MICU1	-0.49	-0.71	AR-V7
PCSK1N	-0.09	-0.71	AR-V7
LRP11	-0.41	-0.70	AR-V7
CAT	-0.38	-0.70	AR-V7
JAKMIP3	-0.07	-0.70	AR-V7
AKTIP	-0.38	-0.70	AR-V7
ATP10D	-0.50	-0.70	AR-V7
ELP4	-0.55	-0.70	AR-V7
SCFD2	-0.12	-0.69	AR-V7
A2ML1	-0.46	-0.69	AR-V7
TMSB15A	-0.09	-0.69	AR-V7
SKOR1	-0.89	-0.45	AR-FL
VIPAS39	-0.80	-0.45	AR-FL
FUZ	-0.68	-0.45	AR-FL
STX2	-0.85	-0.45	AR-FL
ZNF382	-0.97	-0.45	AR-FL
POU4F1	-1.45	-0.45	AR-FL
IKZF4	-0.79	-0.45	AR-FL
LGALS8	-0.93	-0.45	AR-FL
ATIC	-0.70	-0.45	AR-FL
GNB1L	-0.71	-0.45	AR-FL
SBF2	-0.86	-0.45	AR-FL
ZNF496	-0.91	-0.45	AR-FL
PLK3	-1.12	-0.45	AR-FL
BIVM	-0.68	-0.45	AR-FL
ZNF234	-0.81	-0.45	AR-FL
BCAS3	-0.75	-0.45	AR-FL
NAP1L1	-1.11	-0.45	AR-FL
RPSA	-0.66	-0.45	AR-FL
ACE	-1.53	-0.44	AR-FL
ADAM33	-0.66	-0.44	AR-FL
GRIP2	-1.61	-0.44	AR-FL
KIAA0895	-0.93	-0.44	AR-FL
TPP2	-0.69	-0.44	AR-FL
DNAJC27	-0.97	-0.44	AR-FL
PAX8	-2.04	-0.44	AR-FL
MKNK1	-0.67	-0.44	AR-FL
METTL21B	-1.09	-0.44	AR-FL
EPHB4	-1.06	-0.44	AR-FL
CHML	-1.25	-0.44	AR-FL
GRB7	-0.94	-0.44	AR-FL
OSER1	-0.71	-0.44	AR-FL
GREB1L	-1.42	-0.44	AR-FL
DECR1	-1.19	-0.44	AR-FL
CEP95	-1.26	-0.44	AR-FL
NR1H4	-1.31	-0.44	AR-FL
PMS2	-0.65	-0.44	AR-FL
ANAPC4	-1.01	-0.44	AR-FL
MC1R	-1.38	-0.44	AR-FL
BEND3	-0.98	-0.44	AR-FL
TMEM102	-0.68	-0.44	AR-FL
PCDHB15	-1.29	-0.44	AR-FL
NEFM	-1.44	-0.44	AR-FL
ZDHHC2	-0.71	-0.44	AR-FL
TTC33	-0.93	-0.44	AR-FL
YPEL3	-1.35	-0.44	AR-FL
ZNF133	-0.92	-0.44	AR-FL

ZFYVE9	-0.28	-0.69	AR-V7	C12orf76	-0.99	-0.44	AR-FL
ETFDH	-0.37	-0.69	AR-V7	C12orf29	-0.84	-0.44	AR-FL
PKHD1L1	-0.54	-0.69	AR-V7	SUSD5	-1.64	-0.44	AR-FL
PRSS27	-0.48	-0.69	AR-V7	ATP1A3	-1.95	-0.43	AR-FL
CCDC110	-0.10	-0.68	AR-V7	FBLL1	-1.05	-0.43	AR-FL
GATA6	-0.47	-0.68	AR-V7	YAE1D1	-0.68	-0.43	AR-FL
CFD	-0.57	-0.68	AR-V7	HIBADH	-0.70	-0.43	AR-FL
TSPEAR	-0.48	-0.68	AR-V7	PDK1	-0.73	-0.43	AR-FL
ZP3	-0.30	-0.68	AR-V7	NREP	-0.72	-0.43	AR-FL
NUAK1	-0.57	-0.68	AR-V7	RHOV	-0.75	-0.43	AR-FL
TMEM123	-0.57	-0.68	AR-V7	IKZF3	-1.07	-0.43	AR-FL
ARSB	-0.28	-0.68	AR-V7	BHLHB9	-1.01	-0.43	AR-FL
SCML2	-0.53	-0.68	AR-V7	EGR1	-0.78	-0.43	AR-FL
C2CD2	-0.21	-0.68	AR-V7	TTR	-2.46	-0.43	AR-FL
CCND1	-0.27	-0.68	AR-V7	ARHGAP5	-0.68	-0.43	AR-FL
STK24	-0.17	-0.68	AR-V7	KIAA1958	-0.90	-0.43	AR-FL
PTMA	-0.20	-0.68	AR-V7	KLB	-0.98	-0.43	AR-FL
TYRO3	-0.24	-0.67	AR-V7	EML5	-0.69	-0.43	AR-FL
POLD2	-0.23	-0.67	AR-V7	SGCE	-0.98	-0.43	AR-FL
PRR19	-0.15	-0.67	AR-V7	RAMP1	-0.98	-0.43	AR-FL
NQO2	-0.17	-0.67	AR-V7	SLC2A11	-0.93	-0.43	AR-FL
OSBP2	-0.51	-0.67	AR-V7	KALRN	-0.80	-0.43	AR-FL
DBNDD1	-0.19	-0.67	AR-V7	SETDB1	-0.77	-0.43	AR-FL
MRPL14	-0.16	-0.67	AR-V7	SLC35D2	-0.70	-0.43	AR-FL
C19orf66	-0.13	-0.67	AR-V7	SLC25A27	-1.76	-0.43	AR-FL
R3HDM1	-0.39	-0.67	AR-V7	TOMM20	-0.76	-0.43	AR-FL
ADAT2	-0.25	-0.67	AR-V7	DOC2A	-2.38	-0.43	AR-FL
VWA1	-0.37	-0.66	AR-V7	FAM171A1	-1.10	-0.43	AR-FL
IGSF3	-0.34	-0.66	AR-V7	PGAP1	-1.69	-0.43	AR-FL
CHTF18	-0.54	-0.66	AR-V7	KCNAB2	-2.01	-0.43	AR-FL
PKN1	-0.28	-0.66	AR-V7	ORC3	-0.81	-0.43	AR-FL
XPOT	-0.12	-0.66	AR-V7	HNRNPA1L2	-0.80	-0.42	AR-FL
CPS1	-0.43	-0.66	AR-V7	DTX3	-0.86	-0.42	AR-FL
CSPG5	-0.33	-0.66	AR-V7	GTF2IRD2	-0.79	-0.42	AR-FL
SGMS1	-0.50	-0.66	AR-V7	KCNJ12	-1.80	-0.42	AR-FL
POU2F1	-0.42	-0.66	AR-V7	SGCB	-1.16	-0.42	AR-FL
TBC1D5	-0.47	-0.66	AR-V7	KIAA1683	-1.67	-0.42	AR-FL
UHRF1BP1	-0.10	-0.66	AR-V7	FOXL2NB	-1.09	-0.42	AR-FL
RTN4RL2	-0.42	-0.66	AR-V7	ANKRD36C	-1.99	-0.42	AR-FL
SLC7A1	-0.15	-0.66	AR-V7	AKAP5	-0.89	-0.42	AR-FL
PAK2	-0.51	-0.65	AR-V7	CYP7B1	-0.99	-0.42	AR-FL
ZFYVE28	-0.53	-0.65	AR-V7	STRADB	-0.78	-0.42	AR-FL
EEFSEC	-0.52	-0.65	AR-V7	KIAA1324	-0.84	-0.42	AR-FL
PPP1R3E	-0.17	-0.65	AR-V7	HNRNPA1	-0.93	-0.42	AR-FL
APOBEC3F	-0.27	-0.65	AR-V7	TFDP2	-1.13	-0.42	AR-FL
SPHK1	-0.52	-0.65	AR-V7	GLMN	-0.63	-0.42	AR-FL

FAM216A	-0.58	-0.65	AR-V7
YARS	-0.09	-0.65	AR-V7
LRP8	-0.13	-0.65	AR-V7
MPP7	-0.27	-0.65	AR-V7
FKBP4	-0.28	-0.64	AR-V7
IGSF1	-0.21	-0.64	AR-V7
TUBA1A	-0.39	-0.64	AR-V7
RRP1B	-0.15	-0.64	AR-V7
DYNLT1	-0.34	-0.64	AR-V7
TFAP2E	-0.19	-0.64	AR-V7
FKBP10	-0.19	-0.64	AR-V7
C3orf33	-0.18	-0.63	AR-V7
TKT	-0.24	-0.63	AR-V7
FAM124A	-0.13	-0.63	AR-V7
RRP1	-0.14	-0.63	AR-V7
ZNF704	-0.56	-0.63	AR-V7
TLE2	-0.39	-0.63	AR-V7
TSNARE1	-0.34	-0.63	AR-V7
TMEM97	-0.38	-0.63	AR-V7
GRASP	-0.05	-0.62	AR-V7
SH3GLB2	-0.42	-0.62	AR-V7
SPTY2D1-AS1	-0.21	-0.62	AR-V7
NR1D2	-0.34	-0.62	AR-V7
PSMG4	-0.29	-0.62	AR-V7
TPK1	-0.17	-0.62	AR-V7
SPIN4	-0.30	-0.62	AR-V7
ARHGEF38	-0.50	-0.62	AR-V7
CC2D2A	-0.24	-0.62	AR-V7
RAP2A	-0.27	-0.62	AR-V7
FZD6	-0.26	-0.62	AR-V7
DIMT1	-0.25	-0.62	AR-V7
SNED1	-0.36	-0.61	AR-V7
LRRC8D	-0.56	-0.61	AR-V7
NBEAL2	-0.38	-0.61	AR-V7
IPO4	-0.21	-0.61	AR-V7
SYNJ2	-0.37	-0.61	AR-V7
PSME1	-0.37	-0.61	AR-V7
OXCT1	-0.19	-0.61	AR-V7
TMEM243	-0.37	-0.61	AR-V7
HPRT1	-0.24	-0.61	AR-V7
SERTAD2	-0.38	-0.61	AR-V7
PKD2	-0.28	-0.61	AR-V7
SEMA3F	-0.24	-0.61	AR-V7
ZCCHC24	-0.14	-0.61	AR-V7
CENPJ	-0.34	-0.61	AR-V7
PSME2	-0.04	-0.61	AR-V7
ZCCHC11	-0.72	-0.42	AR-FL
TMEM42	-0.61	-0.42	AR-FL
EIF2S3	-0.67	-0.42	AR-FL
DCAF4	-1.38	-0.42	AR-FL
PNCK	-2.09	-0.42	AR-FL
RNF144A	-0.61	-0.42	AR-FL
C11orf57	-0.68	-0.42	AR-FL
FJX1	-1.35	-0.42	AR-FL
IGFALS	-1.10	-0.42	AR-FL
OTUD7A	-1.50	-0.42	AR-FL
CDHR1	-3.83	-0.42	AR-FL
TMEM178A	-1.52	-0.42	AR-FL
CALD1	-0.60	-0.42	AR-FL
SLC44A1	-0.78	-0.42	AR-FL
CLIP2	-0.58	-0.41	AR-FL
MAST1	-1.44	-0.41	AR-FL
ZNF302	-0.67	-0.41	AR-FL
EFEMP2	-0.65	-0.41	AR-FL
FAM78B	-1.23	-0.41	AR-FL
LRIG2	-0.78	-0.41	AR-FL
NECAB2	-1.36	-0.41	AR-FL
ZNF501	-0.75	-0.41	AR-FL
ZNF558	-0.91	-0.41	AR-FL
TTC28	-0.95	-0.41	AR-FL
TTC34	-1.74	-0.41	AR-FL
COL6A1	-1.71	-0.41	AR-FL
C11orf53	-1.73	-0.41	AR-FL
AP1AR	-0.78	-0.41	AR-FL
NDRG2	-0.65	-0.41	AR-FL
ZBTB44	-0.93	-0.41	AR-FL
TBPL1	-0.95	-0.41	AR-FL
RFX2	-0.59	-0.41	AR-FL
TYMP	-0.99	-0.41	AR-FL
MCF2L2	-0.88	-0.41	AR-FL
RIOK2	-0.60	-0.41	AR-FL
SLC16A14	-0.72	-0.40	AR-FL
COG8	-0.59	-0.40	AR-FL
SLC24A1	-0.79	-0.40	AR-FL
ZNF662	-0.77	-0.40	AR-FL
WDSUB1	-0.83	-0.40	AR-FL
CCDC115	-0.89	-0.40	AR-FL
CGREF1	-0.85	-0.40	AR-FL
NEK9	-0.73	-0.40	AR-FL
AJUBA	-1.17	-0.40	AR-FL
YJEFN3	-1.47	-0.40	AR-FL
SLC16A9	-2.64	-0.40	AR-FL

ZRANB3	-0.48	-0.61	AR-V7
GTF2H2C	-0.28	-0.61	AR-V7
RNF34	-0.48	-0.61	AR-V7
PEX7	-0.16	-0.61	AR-V7
NOD1	-0.21	-0.61	AR-V7
USP25	-0.18	-0.61	AR-V7
RCN1	-0.02	-0.61	AR-V7
FAM156B	-0.03	-0.60	AR-V7
RFX5	-0.10	-0.60	AR-V7
CPNE7	-0.37	-0.60	AR-V7
PCDHB14	-0.57	-0.60	AR-V7
SMYD3	-0.47	-0.60	AR-V7
MYH10	-0.35	-0.60	AR-V7
KLF10	-0.58	-0.60	AR-V7
HNF1B	-0.43	-0.60	AR-V7
LRRC58	-0.19	-0.60	AR-V7
RGS3	-0.50	-0.60	AR-V7
NOS1AP	-0.23	-0.60	AR-V7
OVOL1	-0.53	-0.60	AR-V7
MBOAT7	-0.29	-0.60	AR-V7
IPO5	-0.17	-0.60	AR-V7
BCAT2	-0.51	-0.60	AR-V7
VPS54	-0.34	-0.60	AR-V7
ANGEL1	-0.29	-0.59	AR-V7
ZSCAN5A	-0.39	-0.59	AR-V7
MLLT1	-0.21	-0.59	AR-V7
BCL2L11	-0.17	-0.59	AR-V7
AK6	-0.01	-0.59	AR-V7
CMSS1	-0.56	-0.59	AR-V7
PAICS	-0.23	-0.59	AR-V7
TAF4B	-0.46	-0.59	AR-V7
TMEM159	-0.52	-0.58	AR-V7
PRMT3	-0.49	-0.58	AR-V7
PCNT	-0.17	-0.58	AR-V7
CLSTN1	-0.55	-0.58	AR-V7
TIMELESS	-0.48	-0.58	AR-V7
TEX30	-0.42	-0.58	AR-V7
SYNE3	-0.27	-0.58	AR-V7
EIF4EBP3	-0.24	-0.58	AR-V7
DNAJC13	-0.19	-0.58	AR-V7
DNAJC17	0.24	0.58	AR-V7
WDR13	0.34	0.58	AR-V7
ZMYND8	0.36	0.58	AR-V7
GPS2	0.31	0.58	AR-V7
ENDOG	0.13	0.58	AR-V7
HIC2	0.41	0.59	AR-V7

PALD1	-1.35	-0.40	AR-FL
ZBTB32	-0.59	-0.40	AR-FL
APC2	-0.85	-0.40	AR-FL
ST13	-0.66	-0.40	AR-FL
PUS10	-0.79	-0.40	AR-FL
NOL8	-0.97	-0.40	AR-FL
HACE1	-0.66	-0.40	AR-FL
ABCA4	-0.97	-0.39	AR-FL
CHKB-CPT1B	-1.00	-0.39	AR-FL
RMDN2	-0.82	-0.39	AR-FL
STC2	-1.02	-0.39	AR-FL
TRIM66	-1.41	-0.39	AR-FL
ZNF112	-0.61	-0.39	AR-FL
KLHL3	-1.64	-0.39	AR-FL
CLYBL	-1.53	-0.39	AR-FL
IRF6	-1.01	-0.39	AR-FL
ZNF404	-0.90	-0.39	AR-FL
TOX	-2.26	-0.39	AR-FL
TIGD1	-0.64	-0.39	AR-FL
ZCCHC14	-0.82	-0.39	AR-FL
RANBP17	-0.87	-0.39	AR-FL
NTAN1	-0.93	-0.39	AR-FL
FTO	-0.80	-0.39	AR-FL
L3HYPDH	-0.94	-0.39	AR-FL
ZNF34	-1.19	-0.39	AR-FL
DTWD1	-1.03	-0.39	AR-FL
SLC9A7	-0.60	-0.39	AR-FL
ACBD4	-0.71	-0.39	AR-FL
GPT	-0.79	-0.39	AR-FL
ACAT1	-0.59	-0.38	AR-FL
VPS13C	-0.67	-0.38	AR-FL
PANK1	-0.80	-0.38	AR-FL
ELAVL2	-0.90	-0.38	AR-FL
SPEF2	-1.03	-0.38	AR-FL
SLC19A2	-1.22	-0.38	AR-FL
ZNF844	-0.97	-0.38	AR-FL
LRRC8C	-1.21	-0.38	AR-FL
EBF1	-1.20	-0.38	AR-FL
NAB1	-1.23	-0.38	AR-FL
HEXDC	-0.75	-0.38	AR-FL
PNMAL1	-1.78	-0.38	AR-FL
PBLD	-0.92	-0.38	AR-FL
IFIT5	-0.58	-0.38	AR-FL
ANGEL1	-0.60	-0.38	AR-FL
TTC27	-0.80	-0.38	AR-FL
1-Sep	-1.00	-0.38	AR-FL

CELSR3	0.09	0.59	AR-V7	CCDC85B	-0.77	-0.38	AR-FL
EIF2B2	0.36	0.59	AR-V7	FSD1L	-0.93	-0.38	AR-FL
CIITA	0.06	0.59	AR-V7	OXCT1	-0.63	-0.38	AR-FL
SMN2	0.27	0.59	AR-V7	AHSA2	-1.06	-0.38	AR-FL
ANKRD24	0.05	0.59	AR-V7	APAF1	-0.60	-0.38	AR-FL
LAMB3	0.31	0.59	AR-V7	ZNF449	-0.71	-0.38	AR-FL
HILPDA	0.29	0.59	AR-V7	HPS4	-0.70	-0.38	AR-FL
HACD4	0.10	0.59	AR-V7	PTCD2	-1.24	-0.38	AR-FL
ANKIB1	0.18	0.59	AR-V7	PAK6	-0.76	-0.38	AR-FL
WDSUB1	0.02	0.59	AR-V7	KISS1R	-1.14	-0.38	AR-FL
RP11-468E2.4	0.29	0.59	AR-V7	PLXDC1	-2.09	-0.38	AR-FL
FAM63B	0.31	0.59	AR-V7	CLUL1	-0.67	-0.37	AR-FL
EHF	0.43	0.60	AR-V7	SENP8	-0.67	-0.37	AR-FL
TRIO	0.49	0.60	AR-V7	TMEM69	-0.62	-0.37	AR-FL
TUT1	0.43	0.60	AR-V7	WDR17	-1.53	-0.37	AR-FL
NUTM2D	0.05	0.60	AR-V7	PLXND1	-1.16	-0.37	AR-FL
WDR5B	0.44	0.60	AR-V7	CLTCL1	-0.95	-0.37	AR-FL
SLC36A4	0.53	0.60	AR-V7	TXNIP	-0.86	-0.37	AR-FL
KIF16B	0.03	0.60	AR-V7	EFNA2	-0.70	-0.37	AR-FL
VAPA	0.31	0.60	AR-V7	FAM161B	-1.27	-0.37	AR-FL
MED9	0.29	0.60	AR-V7	WWOX	-0.70	-0.37	AR-FL
ZNF622	0.47	0.60	AR-V7	PLCL2	-1.09	-0.37	AR-FL
AHR	0.13	0.60	AR-V7	MGEA5	-0.90	-0.37	AR-FL
LARP1B	0.56	0.60	AR-V7	SULT1A1	-1.34	-0.37	AR-FL
LRFN4	0.25	0.60	AR-V7	SLC27A1	-1.02	-0.37	AR-FL
MAGED1	0.37	0.61	AR-V7	PYROXD2	-0.83	-0.37	AR-FL
NARF	0.10	0.61	AR-V7	SMARCD3	-1.35	-0.37	AR-FL
MLEC	0.55	0.61	AR-V7	DENND6B	-0.95	-0.37	AR-FL
KCTD7	0.26	0.61	AR-V7	GLTSCR2	-1.04	-0.37	AR-FL
HYPK	0.18	0.61	AR-V7	LHX4	-1.00	-0.37	AR-FL
NAGK	0.14	0.61	AR-V7	ERMP1	-1.17	-0.37	AR-FL
SLF1	0.04	0.61	AR-V7	QARS	-0.66	-0.37	AR-FL
DIXDC1	0.33	0.61	AR-V7	CBX7	-1.10	-0.37	AR-FL
PNCK	0.20	0.61	AR-V7	SUMF1	-0.59	-0.37	AR-FL
HIVEP2	0.12	0.61	AR-V7	ETAA1	-0.73	-0.37	AR-FL
ARRDC3	0.25	0.61	AR-V7	GEMIN5	-0.77	-0.36	AR-FL
NME5	0.02	0.62	AR-V7	ARFGEF1	-0.65	-0.36	AR-FL
SLC39A1	0.49	0.62	AR-V7	RNF165	-0.71	-0.36	AR-FL
MTMR7	0.06	0.62	AR-V7	MXRA8	-0.74	-0.36	AR-FL
DYNC2LI1	0.12	0.62	AR-V7	NCKAP5L	-1.24	-0.36	AR-FL
CBLB	0.04	0.62	AR-V7	SEMA3B	-0.75	-0.36	AR-FL
DPH3	0.43	0.62	AR-V7	NLGN4Y	-0.66	-0.36	AR-FL
BRICD5	0.15	0.62	AR-V7	EIF3M	-0.63	-0.36	AR-FL
CREBRF	0.23	0.62	AR-V7	RP5-862P8.2	-0.71	-0.36	AR-FL
ZNF701	0.37	0.62	AR-V7	DARS	-0.58	-0.36	AR-FL
SLC27A3	0.06	0.62	AR-V7	EPHA10	-1.01	-0.36	AR-FL

ENAH	0.37	0.62	AR-V7
MIIP	0.36	0.62	AR-V7
FERMT3	0.26	0.62	AR-V7
VPS37B	0.31	0.62	AR-V7
TSPAN6	0.24	0.62	AR-V7
MGST3	0.41	0.63	AR-V7
KIFAP3	0.41	0.63	AR-V7
IL6R	0.50	0.63	AR-V7
SMTN	0.32	0.63	AR-V7
ARL1	0.52	0.63	AR-V7
IQCB1	0.43	0.63	AR-V7
ANKFY1	0.58	0.63	AR-V7
C4orf33	0.11	0.63	AR-V7
TBC1D20	0.23	0.63	AR-V7
ZNF781	0.10	0.63	AR-V7
ACBD7	0.29	0.63	AR-V7
ASCC1	0.22	0.64	AR-V7
C4orf48	0.04	0.64	AR-V7
TBC1D10A	0.50	0.64	AR-V7
PKP2	0.51	0.64	AR-V7
SAFB2	0.20	0.64	AR-V7
AP4E1	0.53	0.64	AR-V7
NDUFV2	0.25	0.64	AR-V7
DDTL	0.51	0.64	AR-V7
CPEB4	0.25	0.64	AR-V7
SLC26A6	0.21	0.64	AR-V7
MED10	0.28	0.65	AR-V7
PSENEN	0.57	0.65	AR-V7
PRSS8	0.05	0.65	AR-V7
SNX1	0.17	0.65	AR-V7
SLC52A3	0.12	0.65	AR-V7
P4HA1	0.07	0.65	AR-V7
ZNF586	0.36	0.65	AR-V7
TAX1BP3	0.18	0.65	AR-V7
SORT1	0.34	0.65	AR-V7
TGFBR3	0.11	0.65	AR-V7
C15orf62	0.02	0.65	AR-V7
LDLRAD4	0.35	0.65	AR-V7
JUN	0.49	0.65	AR-V7
MAP3K12	0.20	0.65	AR-V7
DOK4	0.01	0.65	AR-V7
MAP1LC3B	0.30	0.66	AR-V7
PLXNA3	0.28	0.66	AR-V7
HPS3	0.11	0.66	AR-V7
DDN	0.20	0.66	AR-V7
KIAA0040	0.33	0.66	AR-V7

MTA3	-0.60	-0.36	AR-FL
KCNMB3	-1.15	-0.36	AR-FL
SMO	-0.81	-0.36	AR-FL
GDAP1L1	-1.61	-0.36	AR-FL
ZMIZ1	-0.87	-0.36	AR-FL
SPIN3	-0.75	-0.36	AR-FL
ZBED8	-0.60	-0.36	AR-FL
ZNF835	-1.20	-0.36	AR-FL
GSTO1	-0.67	-0.36	AR-FL
ACADSB	-0.89	-0.36	AR-FL
ZBTB47	-0.87	-0.36	AR-FL
EMX1	-1.32	-0.36	AR-FL
ZNF286B	-1.38	-0.36	AR-FL
DOK1	-0.63	-0.36	AR-FL
KBTBD3	-1.32	-0.35	AR-FL
C5orf42	-0.65	-0.35	AR-FL
ELP4	-0.62	-0.35	AR-FL
TNKS	-0.68	-0.35	AR-FL
CASP8	-0.69	-0.35	AR-FL
TGFA	-1.07	-0.35	AR-FL
SYTL3	-1.28	-0.35	AR-FL
MAOA	-1.50	-0.35	AR-FL
KIAA1107	-1.49	-0.35	AR-FL
CERS1	-1.75	-0.35	AR-FL
AHNAK2	-1.16	-0.35	AR-FL
MRPS27	-0.67	-0.35	AR-FL
KLRG2	-1.30	-0.35	AR-FL
ULK2	-0.65	-0.35	AR-FL
IFFO2	-0.70	-0.35	AR-FL
EXT1	-0.91	-0.35	AR-FL
ENPP3	-0.90	-0.35	AR-FL
C19orf44	-0.98	-0.35	AR-FL
HEXIM2	-0.82	-0.35	AR-FL
DDX50	-0.63	-0.35	AR-FL
CEP85L	-0.87	-0.35	AR-FL
ALOXE3	-1.17	-0.35	AR-FL
INSR	-0.62	-0.35	AR-FL
CRYZL1	-0.75	-0.35	AR-FL
MRRF	-0.59	-0.35	AR-FL
ZNF846	-0.82	-0.35	AR-FL
ARHGAP44	-0.99	-0.35	AR-FL
SP4	-0.79	-0.34	AR-FL
GNG12	-0.59	-0.34	AR-FL
GGT7	-1.24	-0.34	AR-FL
DYNC2H1	-1.75	-0.34	AR-FL
FAM149A	-1.00	-0.34	AR-FL

UBE2V1	0.19	0.66	AR-V7	NAT6	-0.67	-0.34	AR-FL
RAMP1	0.20	0.66	AR-V7	BACE1	-0.95	-0.34	AR-FL
COMMD6	0.32	0.66	AR-V7	FCHSD2	-0.76	-0.34	AR-FL
FAM104A	0.37	0.66	AR-V7	TAPT1	-0.71	-0.34	AR-FL
DSTN	0.33	0.66	AR-V7	ZNF605	-0.66	-0.34	AR-FL
HIST1H1B	0.43	0.67	AR-V7	THBS2	-1.17	-0.34	AR-FL
SYS1	0.12	0.67	AR-V7	RPRD2	-0.62	-0.34	AR-FL
DHCR7	0.43	0.67	AR-V7	IL17D	-1.31	-0.34	AR-FL
HBP1	0.55	0.67	AR-V7	LRBA	-0.59	-0.34	AR-FL
POFUT2	0.18	0.67	AR-V7	NAPEPLD	-0.92	-0.34	AR-FL
CHKB	0.12	0.67	AR-V7	SLC36A4	-1.34	-0.34	AR-FL
CRADD	0.37	0.67	AR-V7	MROH6	-0.74	-0.34	AR-FL
SPRED3	0.25	0.68	AR-V7	KANSL1	-0.63	-0.34	AR-FL
MSN	0.29	0.68	AR-V7	WDR74	-0.77	-0.34	AR-FL
GRIN2D	0.04	0.68	AR-V7	FOXK1	-0.65	-0.34	AR-FL
CYP51A1	0.40	0.68	AR-V7	ZNF790	-0.70	-0.34	AR-FL
MIER1	0.34	0.68	AR-V7	APBB1	-1.22	-0.34	AR-FL
NR6A1	0.20	0.68	AR-V7	BRWD1	-0.65	-0.33	AR-FL
OR2A7	0.19	0.68	AR-V7	HEMK1	-1.06	-0.33	AR-FL
KCNAB3	0.52	0.68	AR-V7	MTA1	-0.90	-0.33	AR-FL
TSSK6	0.21	0.68	AR-V7	GPX7	-0.95	-0.33	AR-FL
TMEM234	0.18	0.68	AR-V7	ZNRF3	-0.72	-0.33	AR-FL
ADAM10	0.22	0.68	AR-V7	PPIL6	-1.14	-0.33	AR-FL
ECM1	0.04	0.68	AR-V7	BCHE	-2.05	-0.33	AR-FL
SH3BGRL3	0.04	0.68	AR-V7	XDH	-0.64	-0.33	AR-FL
TUBB2A	0.05	0.68	AR-V7	NBPF3	-0.59	-0.33	AR-FL
SAXO2	0.14	0.69	AR-V7	KLHL21	-0.78	-0.33	AR-FL
NDUFC2	0.27	0.69	AR-V7	ZNF502	-0.75	-0.33	AR-FL
HSH2D	0.48	0.69	AR-V7	ZNF740	-0.65	-0.33	AR-FL
S100A2	0.31	0.69	AR-V7	FBXO46	-0.73	-0.33	AR-FL
C10orf10	0.20	0.69	AR-V7	CLUAP1	-0.95	-0.33	AR-FL
MSL3	0.12	0.69	AR-V7	ZNF233	-0.77	-0.33	AR-FL
CES2	0.24	0.69	AR-V7	PAK3	-2.03	-0.33	AR-FL
APAF1	0.18	0.69	AR-V7	PREX2	-1.02	-0.33	AR-FL
ARL6IP4	0.08	0.69	AR-V7	KIAA1586	-0.59	-0.33	AR-FL
EPOR	0.29	0.69	AR-V7	FES	-0.88	-0.33	AR-FL
GPCPD1	0.19	0.69	AR-V7	RTN1	-0.59	-0.33	AR-FL
ESF1	0.07	0.70	AR-V7	SPG20	-1.40	-0.33	AR-FL
OSCP1	0.29	0.70	AR-V7	IFT74	-0.61	-0.33	AR-FL
ORAI2	0.02	0.70	AR-V7	TET1	-0.83	-0.33	AR-FL
YAF2	0.16	0.70	AR-V7	ZNF788	-0.84	-0.33	AR-FL
WDR60	0.10	0.70	AR-V7	TSPAN10	-0.72	-0.33	AR-FL
CCDC115	0.25	0.70	AR-V7	ZNF45	-0.59	-0.33	AR-FL
CBX4	0.34	0.70	AR-V7	P2RY11	-0.63	-0.33	AR-FL
TBCEL	0.45	0.70	AR-V7	PIGZ	-1.30	-0.33	AR-FL
SAMD15	0.04	0.70	AR-V7	ZNF554	-0.94	-0.32	AR-FL

ZNF625	0.01	0.70	AR-V7
SIRT1	0.54	0.70	AR-V7
NSUN3	0.24	0.70	AR-V7
SMPDL3A	0.44	0.70	AR-V7
CFLAR	0.36	0.70	AR-V7
HIST1H2BJ	0.32	0.70	AR-V7
WBP5	0.55	0.70	AR-V7
UNC13B	0.25	0.70	AR-V7
TSPAN31	0.07	0.71	AR-V7
ARPC1B	0.52	0.71	AR-V7
CIB1	0.37	0.71	AR-V7
ETAA1	0.05	0.71	AR-V7
STX19	0.52	0.71	AR-V7
FLVCR2	0.02	0.71	AR-V7
ZNF281	0.16	0.71	AR-V7
COX17	0.50	0.71	AR-V7
THBS3	0.24	0.71	AR-V7
MAFF	0.57	0.71	AR-V7
DNAJC12	0.43	0.72	AR-V7
MAT1A	0.39	0.72	AR-V7
WBP1L	0.47	0.72	AR-V7
NOTCH2	0.18	0.72	AR-V7
RP11-505K9.4	0.36	0.72	AR-V7
MZT2A	0.15	0.72	AR-V7
FRS3	0.04	0.72	AR-V7
TTC13	0.39	0.72	AR-V7
HOXA1	0.26	0.72	AR-V7
VPS36	0.52	0.73	AR-V7
PARD3	0.32	0.73	AR-V7
BTBD8	0.18	0.73	AR-V7
HIST2H4A	0.11	0.73	AR-V7
CRY1	0.51	0.73	AR-V7
CHST12	0.25	0.73	AR-V7
ADAMTS15	0.30	0.73	AR-V7
ZNF274	0.54	0.73	AR-V7
CCDC107	0.23	0.73	AR-V7
PYGO1	0.31	0.73	AR-V7
SLC29A2	0.56	0.74	AR-V7
HIST1H2BC	0.39	0.74	AR-V7
KBTBD3	0.28	0.74	AR-V7
HDAC8	0.42	0.74	AR-V7
PPP1R3B	0.54	0.74	AR-V7
MEMO1	0.42	0.74	AR-V7
OTUD6B	0.54	0.74	AR-V7
RIT1	0.57	0.74	AR-V7
DERL3	0.57	0.74	AR-V7
ENTPD1	-0.99	-0.32	AR-FL
P3H3	-1.90	-0.32	AR-FL
XYLB	-0.90	-0.32	AR-FL
RENBP	-0.61	-0.32	AR-FL
ABCD4	-0.70	-0.32	AR-FL
ANO6	-0.63	-0.32	AR-FL
MSANTD4	-0.62	-0.32	AR-FL
TMEM231	-1.09	-0.32	AR-FL
MED13L	-0.65	-0.32	AR-FL
C17orf75	-0.64	-0.32	AR-FL
MAPT	-0.76	-0.32	AR-FL
SFRP4	-2.16	-0.32	AR-FL
RPS29	-0.70	-0.32	AR-FL
ASTL	-0.79	-0.32	AR-FL
TRAPPC6B	-0.61	-0.32	AR-FL
SRCIN1	-1.73	-0.32	AR-FL
MTX3	-0.70	-0.32	AR-FL
CLDN15	-1.54	-0.32	AR-FL
ARHGEF17	-1.09	-0.32	AR-FL
C10orf2	-0.82	-0.32	AR-FL
ERLIN2	-0.63	-0.32	AR-FL
MMP24	-1.51	-0.32	AR-FL
CPS1	-1.96	-0.32	AR-FL
LRRC3	-1.14	-0.32	AR-FL
GPALPP1	-0.83	-0.32	AR-FL
FBXW8	-0.78	-0.32	AR-FL
C9orf43	-0.91	-0.31	AR-FL
RSPO4	-0.84	-0.31	AR-FL
RIN3	-1.40	-0.31	AR-FL
QRICH2	-1.32	-0.31	AR-FL
LINGO1	-0.81	-0.31	AR-FL
MACROD1	-0.84	-0.31	AR-FL
OTOGL	-2.35	-0.31	AR-FL
RECQL5	-0.78	-0.31	AR-FL
ENDOV	-0.96	-0.31	AR-FL
SYNE3	-1.04	-0.31	AR-FL
ANP32B	-0.72	-0.31	AR-FL
ANKRD24	-1.40	-0.31	AR-FL
NACA	-0.62	-0.31	AR-FL
CCSAP	-0.81	-0.31	AR-FL
PIK3C3	-0.59	-0.31	AR-FL
NLRC3	-1.06	-0.31	AR-FL
SIRT5	-0.86	-0.31	AR-FL
MLLT10	-0.60	-0.31	AR-FL
NSA2	-0.61	-0.31	AR-FL
PCBD2	-0.69	-0.31	AR-FL

AOC2	0.28	0.75	AR-V7	USP13	-0.81	-0.31	AR-FL
UNC13A	0.42	0.75	AR-V7	TUB	-0.62	-0.31	AR-FL
TRAPPC2P1	0.58	0.75	AR-V7	PAN2	-0.98	-0.31	AR-FL
AC004381.6	0.51	0.75	AR-V7	BTBD2	-0.66	-0.31	AR-FL
EOGT	0.15	0.75	AR-V7	TRIM25	-0.73	-0.30	AR-FL
CYB5R2	0.20	0.75	AR-V7	TRPV1	-1.03	-0.30	AR-FL
HS1BP3	0.47	0.75	AR-V7	ST6GALNAC2	-1.26	-0.30	AR-FL
WDFY1	0.47	0.75	AR-V7	ADGRF5	-3.32	-0.30	AR-FL
SH3GLB1	0.52	0.75	AR-V7	PHGDH	-0.67	-0.30	AR-FL
NXPE3	0.46	0.75	AR-V7	MBNL2	-0.85	-0.30	AR-FL
CCDC87	0.19	0.76	AR-V7	ZNF573	-0.85	-0.30	AR-FL
ZNF682	0.08	0.76	AR-V7	CRTAP	-0.60	-0.30	AR-FL
SLC7A8	0.57	0.76	AR-V7	DNPH1	-0.59	-0.30	AR-FL
AGFG1	0.42	0.76	AR-V7	C19orf68	-0.98	-0.30	AR-FL
WDR78	0.08	0.76	AR-V7	VASH1	-1.01	-0.30	AR-FL
CCDC186	0.54	0.76	AR-V7	RNPC3	-0.76	-0.30	AR-FL
ZBTB25	0.35	0.77	AR-V7	QTRT1	-0.61	-0.30	AR-FL
LRP12	0.20	0.77	AR-V7	NKX2-4	-1.45	-0.30	AR-FL
STARD4	0.49	0.77	AR-V7	TFB1M	-0.87	-0.30	AR-FL
UGDH	0.52	0.77	AR-V7	RPGRIP1L	-0.97	-0.30	AR-FL
LMTK3	0.21	0.77	AR-V7	RPL5	-0.83	-0.30	AR-FL
CCDC24	0.32	0.77	AR-V7	ASB13	-0.75	-0.30	AR-FL
STOML1	0.34	0.77	AR-V7	USP36	-0.73	-0.30	AR-FL
HIST1H2BF	0.33	0.77	AR-V7	TMEM182	-1.15	-0.30	AR-FL
APLP2	0.52	0.78	AR-V7	NOC3L	-0.75	-0.30	AR-FL
CCBL1	0.47	0.78	AR-V7	GSTM4	-1.05	-0.30	AR-FL
SPSB3	0.23	0.78	AR-V7	CNTFR	-2.07	-0.30	AR-FL
NICN1	0.27	0.78	AR-V7	GPC4	-0.72	-0.30	AR-FL
CHKB-CPT1B	0.11	0.78	AR-V7	KSR2	-0.96	-0.30	AR-FL
GYG1	0.39	0.78	AR-V7	ZCCHC8	-0.82	-0.29	AR-FL
FAS	0.15	0.79	AR-V7	MGME1	-1.25	-0.29	AR-FL
CUTC	0.28	0.79	AR-V7	BBS9	-0.92	-0.29	AR-FL
POLI	0.27	0.79	AR-V7	ANKS6	-0.78	-0.29	AR-FL
GCC2	0.42	0.79	AR-V7	SMC1B	-1.13	-0.29	AR-FL
ACSS2	0.35	0.79	AR-V7	BRD8	-0.66	-0.29	AR-FL
TGS1	0.03	0.79	AR-V7	HNRNPDL	-0.62	-0.29	AR-FL
MVK	0.53	0.79	AR-V7	RASL10B	-3.19	-0.29	AR-FL
HEPH	0.44	0.80	AR-V7	LBHD1	-0.68	-0.29	AR-FL
LDHD	0.40	0.81	AR-V7	AP4S1	-1.37	-0.29	AR-FL
ARG2	0.52	0.81	AR-V7	GSDMB	-1.29	-0.29	AR-FL
FOSL2	0.48	0.81	AR-V7	SLX4IP	-0.86	-0.29	AR-FL
HPD	0.56	0.81	AR-V7	FOSL2	-1.34	-0.29	AR-FL
THOC5	0.30	0.81	AR-V7	SNRPA	-0.62	-0.29	AR-FL
PTPN18	0.43	0.81	AR-V7	ZFHX2	-0.89	-0.29	AR-FL
MYL6	0.22	0.82	AR-V7	TMEM39B	-0.75	-0.29	AR-FL
CASP10	0.28	0.82	AR-V7	KHK	-1.06	-0.29	AR-FL

ZNF165	0.41	0.82	AR-V7
PEBP4	0.20	0.83	AR-V7
ZNF527	0.19	0.83	AR-V7
FOS	0.38	0.84	AR-V7
SOX15	0.06	0.85	AR-V7
C1orf54	0.12	0.85	AR-V7
SSX2IP	0.36	0.85	AR-V7
SLC16A8	0.31	0.85	AR-V7
MMP13	0.24	0.85	AR-V7
NLGN3	0.18	0.85	AR-V7
STAT3	0.52	0.86	AR-V7
SRPK2	0.22	0.86	AR-V7
BIRC2	0.18	0.86	AR-V7
NARFL	0.34	0.86	AR-V7
SYCE3	0.15	0.86	AR-V7
RNF170	0.27	0.86	AR-V7
FBLL1	0.42	0.86	AR-V7
ASCC3	0.30	0.86	AR-V7
RASSF1	0.28	0.87	AR-V7
PPP1R15A	0.37	0.87	AR-V7
EIF2AK4	0.22	0.87	AR-V7
MVD	0.48	0.87	AR-V7
GPR179	0.03	0.87	AR-V7
PRRG4	0.46	0.87	AR-V7
PCLO	0.41	0.88	AR-V7
ARAF	0.25	0.88	AR-V7
HIST1H2AE	0.39	0.88	AR-V7
MSANTD4	0.26	0.88	AR-V7
C1S	0.08	0.88	AR-V7
TMEM262	0.43	0.88	AR-V7
RELB	0.38	0.88	AR-V7
CAPN9	0.24	0.88	AR-V7
KANK1	0.27	0.89	AR-V7
RP1-34B20.21	0.37	0.89	AR-V7
HS3ST1	0.26	0.89	AR-V7
NUDT18	0.31	0.89	AR-V7
RHBDL1	0.25	0.89	AR-V7
AK8	0.06	0.90	AR-V7
SPATA7	0.45	0.90	AR-V7
SLC22A31	0.44	0.90	AR-V7
C9orf3	0.18	0.90	AR-V7
PIGA	0.53	0.90	AR-V7
GDF15	0.04	0.90	AR-V7
MAP6D1	0.28	0.90	AR-V7
GALNT2	0.43	0.90	AR-V7
TSC2	0.41	0.91	AR-V7
METTL12	-0.58	-0.29	AR-FL
NPW	-1.03	-0.29	AR-FL
WDR83	-0.59	-0.29	AR-FL
DLL3	-0.65	-0.28	AR-FL
DENND3	-0.70	-0.28	AR-FL
DOK3	-0.76	-0.28	AR-FL
ESD	-0.73	-0.28	AR-FL
TRMT10B	-0.66	-0.28	AR-FL
PRKAR1B	-0.88	-0.28	AR-FL
LOXL2	-0.94	-0.28	AR-FL
PROCA1	-1.15	-0.28	AR-FL
IFT46	-0.64	-0.28	AR-FL
CEP57	-1.10	-0.28	AR-FL
RP11-511P7.5	-0.60	-0.28	AR-FL
IPO5	-0.72	-0.28	AR-FL
C19orf66	-0.78	-0.28	AR-FL
PLIN4	-0.83	-0.28	AR-FL
PLCD3	-0.89	-0.28	AR-FL
THBS3	-1.22	-0.28	AR-FL
ZDHHC11B	-1.75	-0.28	AR-FL
SULT4A1	-1.39	-0.28	AR-FL
DLL1	-1.26	-0.28	AR-FL
ARSJ	-1.06	-0.28	AR-FL
BOD1	-0.66	-0.28	AR-FL
PRNP	-0.78	-0.28	AR-FL
CRYM	-1.88	-0.28	AR-FL
PARS2	-0.77	-0.28	AR-FL
LTB4R	-1.38	-0.28	AR-FL
TCN2	-0.82	-0.28	AR-FL
ZNF225	-0.61	-0.27	AR-FL
RABGAP1L	-0.80	-0.27	AR-FL
ENOSF1	-1.08	-0.27	AR-FL
FKBP10	-0.79	-0.27	AR-FL
FBXO41	-0.67	-0.27	AR-FL
AMER1	-0.78	-0.27	AR-FL
NBPF11	-1.22	-0.27	AR-FL
RIMS4	-1.38	-0.27	AR-FL
ZNF221	-1.46	-0.27	AR-FL
PITPNM2	-1.43	-0.27	AR-FL
DAB1	-4.09	-0.27	AR-FL
NEU3	-1.01	-0.27	AR-FL
FAM135B	-2.80	-0.27	AR-FL
DUSP19	-0.89	-0.27	AR-FL
EIF3L	-0.85	-0.27	AR-FL
DLX4	-1.44	-0.27	AR-FL
EEF2	-0.77	-0.27	AR-FL

TXNL4B	0.41	0.91	AR-V7
PEAK1	0.20	0.91	AR-V7
ASIC3	0.04	0.92	AR-V7
CKAP2	0.15	0.92	AR-V7
FGF22	0.10	0.92	AR-V7
ASPG	0.27	0.92	AR-V7
BZW1	0.58	0.93	AR-V7
FAM166B	0.48	0.93	AR-V7
KIAA1161	0.11	0.93	AR-V7
AMT	0.03	0.93	AR-V7
PLEKHG3	0.31	0.94	AR-V7
CCDC82	0.31	0.94	AR-V7
GPLD1	0.19	0.94	AR-V7
EPHX2	0.36	0.94	AR-V7
VWA8	0.12	0.94	AR-V7
GDF9	0.39	0.94	AR-V7
SPATA17	0.20	0.94	AR-V7
AOC3	0.10	0.95	AR-V7
THSD4	0.30	0.95	AR-V7
DCAF4L1	0.27	0.96	AR-V7
SLC22A18	0.36	0.96	AR-V7
PIP5KL1	0.22	0.96	AR-V7
MAP4K5	0.47	0.96	AR-V7
COL7A1	0.12	0.96	AR-V7
PQLC1	0.07	0.96	AR-V7
XKR9	0.44	0.96	AR-V7
BDH2	0.06	0.97	AR-V7
GALR3	0.49	0.98	AR-V7
CRIM1	0.19	0.98	AR-V7
LIMS1	0.54	0.98	AR-V7
IFI27	0.10	0.98	AR-V7
TRIP10	0.33	0.99	AR-V7
C22orf15	0.37	0.99	AR-V7
DUSP15	0.11	0.99	AR-V7
ADAMTSL3	0.35	0.99	AR-V7
PEG10	0.37	0.99	AR-V7
ZNF783	0.08	0.99	AR-V7
SLC22A4	0.44	1.00	AR-V7
PEX2	0.51	1.00	AR-V7
RWDD3	0.07	1.00	AR-V7
DNAJB5	0.55	1.00	AR-V7
EVA1C	0.36	1.00	AR-V7
FAM129B	0.30	1.00	AR-V7
TP53TG5	0.23	1.00	AR-V7
CETN2	0.33	1.01	AR-V7
LZTFL1	0.38	1.01	AR-V7

LRRC49	-0.87	-0.27	AR-FL
RELN	-1.54	-0.27	AR-FL
WNT10B	-2.01	-0.27	AR-FL
ALPK1	-0.69	-0.27	AR-FL
RNF8	-0.60	-0.27	AR-FL
C5orf38	-0.83	-0.26	AR-FL
TMEM25	-0.68	-0.26	AR-FL
CEND1	-0.66	-0.26	AR-FL
FBXO44	-0.77	-0.26	AR-FL
ARHGEF40	-1.61	-0.26	AR-FL
TCAP	-0.62	-0.26	AR-FL
STXBP6	-0.96	-0.26	AR-FL
CMSS1	-0.75	-0.26	AR-FL
DHRS12	-0.62	-0.26	AR-FL
RPL15	-0.73	-0.26	AR-FL
PERM1	-0.70	-0.26	AR-FL
GNB2L1	-0.77	-0.26	AR-FL
STAC3	-1.21	-0.26	AR-FL
TPBGL	-2.39	-0.26	AR-FL
MPP6	-0.72	-0.26	AR-FL
MTAP	-0.84	-0.26	AR-FL
BTF3	-0.61	-0.26	AR-FL
ARID4A	-0.62	-0.26	AR-FL
FAN1	-0.62	-0.26	AR-FL
TRAPPC6A	-0.74	-0.26	AR-FL
KLF2	-0.93	-0.26	AR-FL
AATK	-1.27	-0.26	AR-FL
TMEM18	-0.70	-0.26	AR-FL
LYSMD1	-0.64	-0.26	AR-FL
CD274	-1.50	-0.26	AR-FL
TXLNG	-0.58	-0.26	AR-FL
GNG7	-1.24	-0.26	AR-FL
TPCN1	-0.60	-0.26	AR-FL
PPIL3	-0.69	-0.26	AR-FL
CDC25C	-0.63	-0.26	AR-FL
BCORL1	-1.20	-0.26	AR-FL
EIF3F	-0.63	-0.25	AR-FL
ESYT1	-0.73	-0.25	AR-FL
APOC1	-0.93	-0.25	AR-FL
HOXB7	-0.80	-0.25	AR-FL
C5orf30	-0.74	-0.25	AR-FL
TRNAU1AP	-0.73	-0.25	AR-FL
WDR31	-1.25	-0.25	AR-FL
MLLT6	-0.74	-0.25	AR-FL
ASIC1	-0.64	-0.25	AR-FL
TTYH2	-1.65	-0.25	AR-FL

KIAA1109	0.24	1.01	AR-V7
SUCO	0.54	1.01	AR-V7
TEX14	0.05	1.01	AR-V7
ZNF454	0.09	1.01	AR-V7
SPRY4	0.55	1.02	AR-V7
DUSP1	0.35	1.02	AR-V7
P4HA2	0.20	1.02	AR-V7
MYH9	0.50	1.02	AR-V7
CD24	0.11	1.02	AR-V7
SELM	0.28	1.02	AR-V7
RAB20	0.44	1.03	AR-V7
ITGA10	0.44	1.04	AR-V7
ZNF501	0.29	1.04	AR-V7
FAXDC2	0.09	1.05	AR-V7
SMCHD1	0.34	1.06	AR-V7
IRF7	0.28	1.06	AR-V7
ITGA7	0.15	1.07	AR-V7
SECTM1	0.00	1.07	AR-V7
TLCD2	0.49	1.07	AR-V7
WFDC3	0.27	1.08	AR-V7
LRGUK	0.14	1.08	AR-V7
STX18	0.54	1.08	AR-V7
CSRNP1	0.50	1.08	AR-V7
SMPD3	0.41	1.09	AR-V7
RAB32	0.43	1.09	AR-V7
PPP2R3A	0.30	1.09	AR-V7
ZNF547	0.36	1.09	AR-V7
MFSD7	0.11	1.10	AR-V7
MFAP4	0.39	1.10	AR-V7
MFSD6	0.15	1.10	AR-V7
SMIM1	0.47	1.12	AR-V7
ZNF135	0.06	1.12	AR-V7
AMOTL2	0.43	1.12	AR-V7
NCOR1	0.40	1.12	AR-V7
ENG	0.22	1.12	AR-V7
LMO7	0.56	1.13	AR-V7
RGPD2	0.31	1.14	AR-V7
PIH1D2	0.24	1.14	AR-V7
TYMP	0.45	1.15	AR-V7
HS6ST1	0.07	1.15	AR-V7
UBE2E3	0.14	1.15	AR-V7
GGN	0.37	1.16	AR-V7
PRR15L	0.19	1.16	AR-V7
GHDC	0.52	1.17	AR-V7
USP43	0.32	1.17	AR-V7
CLEC16A	0.24	1.17	AR-V7
DHFRL1	-0.81	-0.25	AR-FL
RORA	-0.86	-0.25	AR-FL
ITSN1	-0.70	-0.25	AR-FL
PDK2	-0.87	-0.25	AR-FL
ACAD10	-1.02	-0.25	AR-FL
GRAMD4	-1.01	-0.25	AR-FL
PALB2	-0.62	-0.25	AR-FL
NUFIP1	-0.75	-0.25	AR-FL
CLK4	-0.90	-0.25	AR-FL
FAM184B	-0.85	-0.25	AR-FL
USP11	-0.84	-0.25	AR-FL
B4GALNT4	-0.80	-0.24	AR-FL
CNNM1	-0.92	-0.24	AR-FL
IGSF22	-0.71	-0.24	AR-FL
AARSD1	-0.72	-0.24	AR-FL
NFKBIZ	-1.63	-0.24	AR-FL
DNMBP	-0.62	-0.24	AR-FL
WIPF3	-1.16	-0.24	AR-FL
PGLS	-0.59	-0.24	AR-FL
AL589743.1	-0.96	-0.24	AR-FL
VPS13A	-0.72	-0.24	AR-FL
COL18A1	-1.03	-0.24	AR-FL
FHL1	-2.73	-0.24	AR-FL
EIF3H	-0.64	-0.24	AR-FL
RNASET2	-1.23	-0.24	AR-FL
TSGA10	-1.09	-0.24	AR-FL
DENND5B	-1.16	-0.24	AR-FL
PPA1	-0.76	-0.24	AR-FL
TRMT1	-0.73	-0.24	AR-FL
TTLL3	-1.11	-0.24	AR-FL
WBSCR27	-1.02	-0.24	AR-FL
CCDC74B	-0.76	-0.23	AR-FL
TSPAN3	-0.64	-0.23	AR-FL
C1orf53	-0.81	-0.23	AR-FL
GAA	-0.67	-0.23	AR-FL
CAMKV	-1.75	-0.23	AR-FL
WDR19	-1.48	-0.23	AR-FL
PIM2	-0.89	-0.23	AR-FL
KLHL15	-0.99	-0.23	AR-FL
MDN1	-0.60	-0.23	AR-FL
SCNN1D	-1.24	-0.23	AR-FL
SMYD2	-0.63	-0.23	AR-FL
GP1BA	-0.82	-0.23	AR-FL
TARBP1	-0.87	-0.23	AR-FL
RPL4	-0.76	-0.23	AR-FL
TMEM64	-0.67	-0.23	AR-FL

SNX33	0.34	1.17	AR-V7
DDX53	0.10	1.17	AR-V7
ASB3	0.34	1.18	AR-V7
DNAJB4	0.28	1.20	AR-V7
DRC3	0.33	1.20	AR-V7
CBWD5	0.43	1.21	AR-V7
RP11-2C24.9	0.55	1.22	AR-V7
HELZ2	0.22	1.22	AR-V7
COX7B2	0.18	1.22	AR-V7
ADAMTS20	0.00	1.22	AR-V7
HIST1H1C	0.46	1.22	AR-V7
SLC47A2	0.36	1.23	AR-V7
ZBED3	0.28	1.24	AR-V7
MFSD2A	0.30	1.24	AR-V7
MAP3K14	0.20	1.24	AR-V7
POF1B	0.46	1.25	AR-V7
H2AFZ	0.19	1.25	AR-V7
HYDIN	0.12	1.26	AR-V7
C16orf86	0.48	1.26	AR-V7
BCL6	0.52	1.26	AR-V7
C4orf47	0.38	1.27	AR-V7
DNAH6	0.04	1.27	AR-V7
ERAP2	0.17	1.27	AR-V7
TFEB	0.56	1.28	AR-V7
PTK2B	0.34	1.28	AR-V7
AP3S1	0.18	1.32	AR-V7
FAM188B	0.23	1.34	AR-V7
ZMYND10	0.09	1.34	AR-V7
TUBB3	0.12	1.34	AR-V7
TBC1D8B	0.45	1.34	AR-V7
TMEM47	0.51	1.35	AR-V7
MARS2	0.50	1.36	AR-V7
PGAP2	0.36	1.36	AR-V7
DUSP5	0.02	1.38	AR-V7
DHRS3	0.33	1.38	AR-V7
CMTM8	0.52	1.39	AR-V7
VIM	0.47	1.40	AR-V7
TACC2	0.35	1.40	AR-V7
GCLC	0.27	1.40	AR-V7
SLC25A18	0.11	1.46	AR-V7
PSTPIP2	0.19	1.49	AR-V7
NREP	0.19	1.49	AR-V7
ARSA	0.36	1.51	AR-V7
IZUMO4	0.50	1.51	AR-V7
BBOF1	0.36	1.51	AR-V7
HSPB1	0.00	1.52	AR-V7
THEM4	-0.64	-0.22	AR-FL
WDR91	-1.08	-0.22	AR-FL
HEATR1	-0.62	-0.22	AR-FL
MATK	-1.44	-0.22	AR-FL
OSBPL1A	-0.70	-0.22	AR-FL
C2CD4C	-0.99	-0.22	AR-FL
WDR27	-1.06	-0.22	AR-FL
NUDT3	-0.59	-0.22	AR-FL
MEST	-0.73	-0.22	AR-FL
FSCN2	-0.73	-0.22	AR-FL
RPS6KA5	-1.00	-0.22	AR-FL
PFAS	-0.88	-0.22	AR-FL
RASGRF1	-0.85	-0.22	AR-FL
RPS4Y1	-0.73	-0.22	AR-FL
EMILIN3	-2.11	-0.22	AR-FL
PTPRS	-0.63	-0.22	AR-FL
PER3	-0.90	-0.22	AR-FL
LRRC37A3	-1.28	-0.21	AR-FL
LTB4R2	-1.10	-0.21	AR-FL
HADH	-0.62	-0.21	AR-FL
S100A13	-0.84	-0.21	AR-FL
NOD1	-1.22	-0.21	AR-FL
SFMBT2	-1.23	-0.21	AR-FL
ZNF141	-0.66	-0.21	AR-FL
C20orf27	-1.01	-0.21	AR-FL
ZNF585B	-0.59	-0.21	AR-FL
EPHX4	-1.87	-0.21	AR-FL
RAD52	-0.69	-0.21	AR-FL
KCTD15	-1.01	-0.21	AR-FL
RPL12	-0.89	-0.20	AR-FL
MYL9	-1.10	-0.20	AR-FL
ATP9A	-0.76	-0.20	AR-FL
SFI1	-1.16	-0.20	AR-FL
CSPP1	-0.67	-0.20	AR-FL
LYRM7	-0.65	-0.20	AR-FL
HOXA2	-1.09	-0.20	AR-FL
TBKBP1	-0.68	-0.20	AR-FL
TPR	-0.62	-0.20	AR-FL
ZNF780B	-0.59	-0.20	AR-FL
RPS25	-0.62	-0.20	AR-FL
RPS3	-0.73	-0.20	AR-FL
PLPP3	-0.97	-0.20	AR-FL
IPO4	-0.73	-0.20	AR-FL
ECHDC2	-0.60	-0.20	AR-FL
ICA1L	-0.68	-0.20	AR-FL
TDRD6	-1.90	-0.20	AR-FL

GAB2	0.41	1.53	AR-V7	TMC4	-0.69	-0.20	AR-FL
FLNA	0.45	1.54	AR-V7	TMEM121	-1.03	-0.20	AR-FL
ZNF728	0.09	1.54	AR-V7	ANKS1B	-1.42	-0.20	AR-FL
ALDH1A2	0.44	1.54	AR-V7	CCDC150	-0.94	-0.20	AR-FL
NFIL3	0.07	1.54	AR-V7	TDRD10	-0.81	-0.20	AR-FL
NRBP2	0.02	1.55	AR-V7	LRP5L	-0.58	-0.19	AR-FL
IGF2BP3	0.33	1.56	AR-V7	FBXO48	-0.69	-0.19	AR-FL
ZNF189	0.25	1.58	AR-V7	ENPP1	-0.97	-0.19	AR-FL
CERS6	0.47	1.60	AR-V7	ENGASE	-0.80	-0.19	AR-FL
TMEM60	0.18	1.61	AR-V7	NUDT13	-0.67	-0.19	AR-FL
CLU	0.17	1.61	AR-V7	FAHD2B	-1.03	-0.19	AR-FL
TNS2	0.01	1.62	AR-V7	GEMIN2	-0.83	-0.19	AR-FL
CEP120	0.21	1.62	AR-V7	CYFIP2	-0.83	-0.19	AR-FL
CHPT1	0.19	1.65	AR-V7	BMP8B	-0.90	-0.19	AR-FL
SNX22	0.29	1.65	AR-V7	ZNF619	-0.59	-0.19	AR-FL
IL17B	0.10	1.65	AR-V7	FAM185A	-0.63	-0.19	AR-FL
SMOX	0.17	1.65	AR-V7	CACNB1	-1.55	-0.19	AR-FL
KLHL31	0.45	1.66	AR-V7	C2orf74	-0.81	-0.19	AR-FL
CHRM3	0.22	1.68	AR-V7	TMCC2	-0.69	-0.19	AR-FL
PLAGL1	0.01	1.68	AR-V7	MYL5	-1.22	-0.19	AR-FL
KCNH2	0.34	1.69	AR-V7	ABCC5	-0.74	-0.19	AR-FL
UPP1	0.24	1.70	AR-V7	CLMN	-1.22	-0.19	AR-FL
S100P	0.45	1.70	AR-V7	GEMIN6	-0.58	-0.18	AR-FL
CNTD1	0.34	1.72	AR-V7	ANKRD61	-0.74	-0.18	AR-FL
RAG1	0.06	1.73	AR-V7	TCEA2	-1.00	-0.18	AR-FL
TASP1	0.41	1.77	AR-V7	RPL27A	-0.61	-0.18	AR-FL
CRYM	0.40	1.78	AR-V7	TNFRSF21	-0.81	-0.18	AR-FL
GBE1	0.17	1.78	AR-V7	C1GALT1	-0.86	-0.18	AR-FL
VPS33B	0.25	1.87	AR-V7	MMRN2	-1.07	-0.18	AR-FL
SPEF1	0.39	1.88	AR-V7	RPL34	-0.61	-0.18	AR-FL
SLC22A1	0.44	1.88	AR-V7	SYNGR3	-1.16	-0.18	AR-FL
SPATA4	0.39	1.89	AR-V7	L3MBTL1	-1.47	-0.18	AR-FL
CCDC108	0.55	1.92	AR-V7	ANKRD18B	-0.90	-0.18	AR-FL
MSX1	0.25	1.95	AR-V7	OSGEPL1	-0.73	-0.18	AR-FL
HIST1H2BG	0.19	1.96	AR-V7	CFAP70	-0.79	-0.17	AR-FL
RAET1L	0.43	1.96	AR-V7	TBC1D10C	-1.23	-0.17	AR-FL
FAM43A	0.32	1.97	AR-V7	TMEM43	-0.62	-0.17	AR-FL
TMSB10	0.17	1.99	AR-V7	INPP5B	-0.98	-0.17	AR-FL
KCND1	0.37	2.03	AR-V7	ERCC6	-0.65	-0.17	AR-FL
XDH	0.41	2.04	AR-V7	RPS15A	-0.72	-0.17	AR-FL
MAP1LC3A	0.27	2.06	AR-V7	CYP2D6	-0.70	-0.17	AR-FL
FAM151A	0.25	2.07	AR-V7	CEP250	-0.58	-0.17	AR-FL
TMEM56	0.56	2.08	AR-V7	HNF1A	-0.65	-0.17	AR-FL
GRIN2C	0.14	2.09	AR-V7	MKRN2OS	-0.61	-0.17	AR-FL
ARHGEF28	0.20	2.09	AR-V7	HSF4	-0.60	-0.17	AR-FL
IFI44	0.42	2.12	AR-V7	SLC44A3	-0.59	-0.17	AR-FL

SRD5A1	0.23	2.13	AR-V7	RPL23	-0.65	-0.17	AR-FL
EPHA7	0.52	2.19	AR-V7	CPQ	-0.96	-0.17	AR-FL
FCHSD1	0.35	2.29	AR-V7	RPS24	-0.64	-0.17	AR-FL
VILL	0.21	2.29	AR-V7	GNB5	-0.80	-0.17	AR-FL
PPM1B	0.54	2.33	AR-V7	PSMG4	-0.71	-0.16	AR-FL
ALOX15B	0.09	2.36	AR-V7	TIAF1	-0.65	-0.16	AR-FL
NAT2	0.25	2.39	AR-V7	PMP22	-1.59	-0.16	AR-FL
DUOX1	0.52	2.42	AR-V7	AZIN2	-1.03	-0.16	AR-FL
SCIN	0.30	2.46	AR-V7	GPM6B	-0.58	-0.16	AR-FL
ANXA1	0.03	2.47	AR-V7	BMP4	-1.15	-0.16	AR-FL
STMN4	0.46	2.53	AR-V7	TUBB4A	-0.72	-0.16	AR-FL
SLC45A1	0.19	2.57	AR-V7	SFR1	-0.78	-0.16	AR-FL
ZNF560	0.15	2.57	AR-V7	COL6A2	-0.92	-0.16	AR-FL
TBX10	0.51	2.62	AR-V7	RPL14	-0.72	-0.16	AR-FL
GLIPR1	0.43	2.67	AR-V7	HMP19	-1.02	-0.16	AR-FL
CYP26B1	0.10	2.73	AR-V7	DDX47	-0.73	-0.16	AR-FL
EPHA6	0.08	2.76	AR-V7	ADGRB1	-1.07	-0.16	AR-FL
GCH1	0.18	2.90	AR-V7	RPLP0	-0.65	-0.16	AR-FL
GPR153	0.55	3.00	AR-V7	RPL13A	-0.72	-0.16	AR-FL
MYO1G	0.04	3.07	AR-V7	SH3BGRL	-0.72	-0.16	AR-FL
MATN3	0.33	3.10	AR-V7	VSTM2L	-0.93	-0.16	AR-FL
JPH4	0.31	3.19	AR-V7	JUN	-1.00	-0.16	AR-FL
TMEM169	0.51	3.28	AR-V7	FAM132B	-1.36	-0.16	AR-FL
MXRA7	0.28	3.35	AR-V7	MAP3K1	-0.86	-0.15	AR-FL
ABLIM3	0.23	3.45	AR-V7	MICALCL	-0.59	-0.15	AR-FL
HRCT1	0.56	3.57	AR-V7	SP5	-1.06	-0.15	AR-FL
ETNK2	0.35	3.61	AR-V7	GPLD1	-1.28	-0.15	AR-FL
DDX4	0.48	3.75	AR-V7	RPL3	-0.81	-0.15	AR-FL
TDRD9	0.54	3.90	AR-V7	INO80B	-0.72	-0.15	AR-FL
WISP2	0.32	4.07	AR-V7	NRP2	-1.02	-0.15	AR-FL
ITIH3	0.23	4.54	AR-V7	INVS	-0.68	-0.15	AR-FL
GNAI1	0.39	4.75	AR-V7	ATHL1	-0.61	-0.15	AR-FL
LAPTM5	0.36	4.75	AR-V7	RPL10A	-0.67	-0.15	AR-FL
TRPC3	0.43	4.77	AR-V7	CD47	-0.70	-0.15	AR-FL
MAGEA4	0.04	4.81	AR-V7	DCAF16	-0.63	-0.15	AR-FL
SKIV2L	0.36	4.82	AR-V7	PODXL	-0.86	-0.15	AR-FL
RALYL	0.36	5.62	AR-V7	SIPA1L2	-0.97	-0.15	AR-FL
ABHD12	0.61	0.00	AR-FL	ZFYVE28	-1.06	-0.15	AR-FL
B3GAT1	1.16	0.00	AR-FL	RPS2	-0.66	-0.15	AR-FL
CHPF	0.89	0.00	AR-FL	ANKRD13A	-0.75	-0.15	AR-FL
TBC1D30	0.95	0.01	AR-FL	FBL	-0.67	-0.15	AR-FL
LYPLA2	0.64	0.01	AR-FL	ACTR3B	-0.63	-0.14	AR-FL
HIVEP1	0.72	0.01	AR-FL	ZNF10	-0.66	-0.14	AR-FL
ERBB2IP	0.59	0.01	AR-FL	AMT	-1.63	-0.14	AR-FL
PLCH2	0.80	0.01	AR-FL	ZNF551	-0.71	-0.14	AR-FL
PPP3CC	0.59	0.01	AR-FL	MBNL3	-0.72	-0.14	AR-FL

ADPGK	0.73	0.01	AR-FL	RPL7A	-0.64	-0.14	AR-FL
UCHL1	1.13	0.01	AR-FL	NME7	-0.61	-0.14	AR-FL
G3BP2	0.77	0.02	AR-FL	FUT10	-0.63	-0.14	AR-FL
TAOK3	1.62	0.02	AR-FL	NOXA1	-1.01	-0.14	AR-FL
ASPHD2	0.96	0.02	AR-FL	CUL4B	-0.73	-0.14	AR-FL
SEC14L2	2.43	0.02	AR-FL	FAM78A	-0.90	-0.14	AR-FL
TFPI	0.59	0.03	AR-FL	TENM2	-1.83	-0.14	AR-FL
TMEM79	2.36	0.03	AR-FL	KCNAB3	-1.50	-0.14	AR-FL
ZBTB24	1.95	0.03	AR-FL	RCL1	-0.62	-0.14	AR-FL
LRP2BP	1.21	0.03	AR-FL	H6PD	-0.58	-0.14	AR-FL
TMCC1	0.58	0.03	AR-FL	RPL22	-0.58	-0.14	AR-FL
REPS2	1.04	0.03	AR-FL	PAPLN	-2.17	-0.14	AR-FL
KDM4B	1.04	0.04	AR-FL	SPAG16	-0.68	-0.14	AR-FL
TMEM198	0.83	0.04	AR-FL	SGSM1	-1.62	-0.14	AR-FL
RNF185	0.58	0.05	AR-FL	RTTN	-0.72	-0.14	AR-FL
SRP68	0.59	0.05	AR-FL	ADSSL1	-0.61	-0.13	AR-FL
MESDC1	0.79	0.05	AR-FL	ACRC	-0.92	-0.13	AR-FL
TMEM50B	0.60	0.05	AR-FL	APBB3	-0.77	-0.13	AR-FL
CAPS	0.75	0.05	AR-FL	RASGRF2	-0.90	-0.13	AR-FL
ADRB1	1.73	0.06	AR-FL	F5	-0.94	-0.13	AR-FL
ACACA	1.04	0.06	AR-FL	LRRC61	-0.88	-0.13	AR-FL
MTMR3	0.68	0.06	AR-FL	RBFA	-0.65	-0.13	AR-FL
OSBP	0.75	0.06	AR-FL	KIF27	-1.06	-0.13	AR-FL
CANT1	0.83	0.06	AR-FL	PLSCR1	-1.66	-0.13	AR-FL
CSGALNACT2	0.69	0.06	AR-FL	RPL7	-0.63	-0.13	AR-FL
TRAPPC11	0.63	0.07	AR-FL	KATNAL2	-1.91	-0.13	AR-FL
SLC17A5	0.76	0.07	AR-FL	TYSND1	-0.59	-0.13	AR-FL
TBK1	0.59	0.07	AR-FL	C3orf67	-1.33	-0.13	AR-FL
GDF11	0.62	0.07	AR-FL	APCDD1	-0.90	-0.13	AR-FL
DOCK11	2.31	0.07	AR-FL	C2orf44	-0.59	-0.13	AR-FL
EIF2S1	0.65	0.07	AR-FL	AVIL	-0.67	-0.13	AR-FL
MXD1	0.70	0.07	AR-FL	ISCU	-0.62	-0.13	AR-FL
MAPK6	0.92	0.07	AR-FL	SPEG	-1.34	-0.13	AR-FL
MKLN1	1.12	0.08	AR-FL	ULBP3	-1.54	-0.12	AR-FL
CREB3L4	1.66	0.08	AR-FL	NOS1	-3.16	-0.12	AR-FL
FBXL8	0.82	0.08	AR-FL	ARRB2	-0.71	-0.12	AR-FL
NUDT9	1.26	0.08	AR-FL	FAM53A	-0.96	-0.12	AR-FL
GBP2	0.96	0.08	AR-FL	PRPF4	-0.59	-0.12	AR-FL
RIPK4	0.64	0.08	AR-FL	LAMC3	-2.94	-0.12	AR-FL
ATMIN	1.08	0.08	AR-FL	PDIA2	-2.36	-0.12	AR-FL
PLPP3	0.74	0.08	AR-FL	PRRT4	-1.21	-0.12	AR-FL
FAM214A	0.72	0.08	AR-FL	C1orf74	-0.65	-0.12	AR-FL
BTAF1	0.94	0.08	AR-FL	POLR2I	-0.61	-0.12	AR-FL
TRIM5	0.83	0.09	AR-FL	EEF1A1	-0.69	-0.12	AR-FL
PAK1IP1	2.70	0.09	AR-FL	SNX7	-0.69	-0.12	AR-FL
DLG2	2.47	0.09	AR-FL	ZNF438	-0.82	-0.12	AR-FL

HM13	0.78	0.09	AR-FL
EPN2	0.87	0.09	AR-FL
CCDC136	0.78	0.09	AR-FL
UBE2J1	1.44	0.09	AR-FL
RGPD6	1.26	0.09	AR-FL
IL10RB	1.10	0.09	AR-FL
PLEKHB2	0.66	0.09	AR-FL
PCTP	1.78	0.10	AR-FL
COG5	0.66	0.10	AR-FL
WSB2	0.65	0.10	AR-FL
COL4A5	0.84	0.10	AR-FL
FECH	0.89	0.10	AR-FL
DYNLL2	0.59	0.10	AR-FL
TAF1A	0.74	0.10	AR-FL
DNAH5	0.83	0.10	AR-FL
MFN1	0.74	0.10	AR-FL
LARP4	0.65	0.11	AR-FL
RALBP1	0.71	0.11	AR-FL
CCDC71L	0.69	0.11	AR-FL
GFM1	1.66	0.11	AR-FL
ERGIC2	1.12	0.11	AR-FL
ACOX3	1.17	0.11	AR-FL
PEX10	2.23	0.11	AR-FL
DHRS12	0.66	0.11	AR-FL
UCHL3	0.72	0.11	AR-FL
PRKCH	1.91	0.12	AR-FL
SAMD9	1.23	0.12	AR-FL
PRDX6	0.65	0.12	AR-FL
SEC16A	0.61	0.12	AR-FL
EFHC1	0.61	0.12	AR-FL
PPARA	0.74	0.13	AR-FL
TFPT	0.90	0.13	AR-FL
THYN1	0.59	0.13	AR-FL
ZNF761	0.64	0.13	AR-FL
APLN	0.59	0.14	AR-FL
GBA2	0.63	0.14	AR-FL
TUSC3	0.79	0.14	AR-FL
SPCS2	0.64	0.14	AR-FL
ACAA1	1.22	0.14	AR-FL
HK2	0.75	0.14	AR-FL
PSMD8	0.79	0.14	AR-FL
OSTM1	0.61	0.14	AR-FL
RGAG4	0.73	0.14	AR-FL
PERP	0.62	0.15	AR-FL
CDK6	2.02	0.15	AR-FL
TPD52	1.73	0.15	AR-FL
PHLDA1	-0.66	-0.12	AR-FL
ARL4D	-0.94	-0.12	AR-FL
TCEANC2	-0.69	-0.12	AR-FL
JAG2	-0.62	-0.12	AR-FL
CAMKK1	-0.87	-0.12	AR-FL
ALAD	-0.58	-0.12	AR-FL
IDNK	-0.67	-0.12	AR-FL
NFXL1	-0.59	-0.11	AR-FL
NPIPA1	-1.01	-0.11	AR-FL
HSPA12A	-1.42	-0.11	AR-FL
GRAMD1B	-1.37	-0.11	AR-FL
MAP6	-0.84	-0.11	AR-FL
PSIP1	-0.64	-0.11	AR-FL
ZNF165	-0.66	-0.11	AR-FL
RPS3A	-0.64	-0.11	AR-FL
ZNF497	-0.74	-0.11	AR-FL
RPL18	-0.62	-0.11	AR-FL
TMEM240	-0.70	-0.11	AR-FL
NNT	-1.57	-0.11	AR-FL
NLRP11	-0.61	-0.11	AR-FL
SYK	-0.77	-0.11	AR-FL
ARRB1	-0.76	-0.11	AR-FL
RPS14	-0.62	-0.10	AR-FL
CCDC82	-0.81	-0.10	AR-FL
PPP1R1C	-1.32	-0.10	AR-FL
PRKAB2	-1.05	-0.10	AR-FL
MAPK10	-0.58	-0.10	AR-FL
KBTBD11	-0.70	-0.10	AR-FL
KCNQ4	-1.00	-0.10	AR-FL
CENPV	-0.66	-0.10	AR-FL
NTN4	-1.98	-0.10	AR-FL
KCP	-1.53	-0.10	AR-FL
HIST1H2BC	-0.75	-0.10	AR-FL
NEIL2	-0.72	-0.10	AR-FL
EFCAB6	-1.03	-0.09	AR-FL
GATM	-0.74	-0.09	AR-FL
BCKDHB	-0.85	-0.09	AR-FL
GPR143	-0.68	-0.09	AR-FL
NTN3	-0.72	-0.09	AR-FL
TFR2	-1.02	-0.09	AR-FL
CEP72	-0.59	-0.09	AR-FL
KIAA1161	-0.63	-0.09	AR-FL
MYOT	-0.63	-0.09	AR-FL
ZHX3	-0.71	-0.08	AR-FL
PAIP2B	-0.71	-0.08	AR-FL
RCOR2	-0.99	-0.08	AR-FL

GTF2E2	1.01	0.15	AR-FL	ZNF529	-0.92	-0.08	AR-FL
USP33	0.99	0.15	AR-FL	SEC31B	-1.03	-0.08	AR-FL
SLC9A3R2	1.23	0.15	AR-FL	ZNF483	-0.75	-0.08	AR-FL
SMPD2	1.64	0.15	AR-FL	NOTCH1	-0.86	-0.08	AR-FL
SND1	0.59	0.16	AR-FL	SPRED3	-0.96	-0.08	AR-FL
SH3D19	0.72	0.16	AR-FL	MECR	-0.80	-0.08	AR-FL
CDC42EP3	2.56	0.16	AR-FL	AARD	-2.87	-0.08	AR-FL
ZFHX3	0.61	0.16	AR-FL	FCGRT	-0.85	-0.08	AR-FL
RAB5A	0.75	0.16	AR-FL	NIPAL3	-0.67	-0.07	AR-FL
SLC35C1	0.70	0.16	AR-FL	OGT	-1.01	-0.07	AR-FL
SNAPC2	0.92	0.16	AR-FL	ASB9	-1.50	-0.07	AR-FL
CD164	0.74	0.17	AR-FL	PRRG1	-0.63	-0.07	AR-FL
TES	0.92	0.17	AR-FL	ZFHX4	-1.04	-0.07	AR-FL
YIPF1	1.30	0.17	AR-FL	PABPC1L	-1.14	-0.07	AR-FL
KIAA1324L	0.73	0.17	AR-FL	RWDD3	-0.87	-0.07	AR-FL
PDE9A	1.31	0.17	AR-FL	ADAMTS17	-1.48	-0.07	AR-FL
MTMR2	1.03	0.17	AR-FL	PCSK4	-1.12	-0.07	AR-FL
TRIM52	1.26	0.18	AR-FL	NANOS1	-0.62	-0.06	AR-FL
TBC1D15	0.74	0.18	AR-FL	SKP2	-0.72	-0.06	AR-FL
GLRX2	1.35	0.18	AR-FL	JAK1	-0.59	-0.06	AR-FL
MTRNR2L8	0.86	0.18	AR-FL	EHHADH	-0.98	-0.06	AR-FL
TMEM125	0.92	0.18	AR-FL	C8orf44	-0.79	-0.06	AR-FL
GGPS1	0.72	0.18	AR-FL	MST1	-1.03	-0.05	AR-FL
PROSER2	1.07	0.18	AR-FL	DSCAM	-2.21	-0.05	AR-FL
SRPRB	0.68	0.18	AR-FL	TRAF1	-1.20	-0.05	AR-FL
GALNT7	1.06	0.18	AR-FL	FAM161A	-0.82	-0.05	AR-FL
DCAF6	0.93	0.19	AR-FL	ZNF665	-0.73	-0.05	AR-FL
FBXO8	0.66	0.19	AR-FL	BEST1	-2.01	-0.05	AR-FL
ATP6V0A2	1.09	0.19	AR-FL	EID2B	-0.92	-0.05	AR-FL
DGKA	1.24	0.19	AR-FL	ZSCAN18	-0.69	-0.04	AR-FL
EZR	0.87	0.19	AR-FL	GOLGA8B	-1.64	-0.04	AR-FL
YES1	0.66	0.19	AR-FL	DCHS1	-0.79	-0.04	AR-FL
S100A11	0.86	0.20	AR-FL	RILPL1	-0.74	-0.04	AR-FL
MEGF9	0.86	0.20	AR-FL	CAD	-0.98	-0.04	AR-FL
CBL	0.61	0.20	AR-FL	DDX51	-0.63	-0.04	AR-FL
BAIAP2	0.95	0.20	AR-FL	RCAN2	-1.04	-0.04	AR-FL
SCYL1	0.59	0.20	AR-FL	RCOR3	-0.60	-0.04	AR-FL
CYTH1	1.61	0.20	AR-FL	CA14	-0.71	-0.04	AR-FL
SH3TC1	0.71	0.20	AR-FL	LTBP3	-0.70	-0.03	AR-FL
PREB	0.66	0.20	AR-FL	OPN3	-0.89	-0.03	AR-FL
TMEM14C	0.62	0.20	AR-FL	HBQ1	-1.43	-0.03	AR-FL
ZNF155	0.65	0.20	AR-FL	CCDC188	-0.70	-0.03	AR-FL
SLC22A23	0.97	0.21	AR-FL	LDHD	-0.92	-0.03	AR-FL
SPATA2	0.62	0.21	AR-FL	UBE2V1	-0.59	-0.03	AR-FL
CTGLF11P	0.84	0.21	AR-FL	ZNF280C	-0.95	-0.03	AR-FL
JPH1	0.70	0.21	AR-FL	ADAM23	-2.10	-0.03	AR-FL

ZBTB37	0.64	0.21	AR-FL
SLC25A20	1.17	0.21	AR-FL
SLC35F2	2.07	0.21	AR-FL
IGBP1	0.66	0.21	AR-FL
ALG2	0.72	0.22	AR-FL
SLC45A2	1.37	0.22	AR-FL
WFS1	0.74	0.22	AR-FL
TNIP2	0.64	0.22	AR-FL
CRNKL1	0.71	0.22	AR-FL
SH3BGRL	1.43	0.22	AR-FL
TMEM2	1.01	0.22	AR-FL
PRODH	0.87	0.22	AR-FL
VMAC	1.00	0.22	AR-FL
SPRED2	0.77	0.22	AR-FL
JKAMP	0.61	0.23	AR-FL
ABHD3	1.17	0.23	AR-FL
COG3	0.61	0.23	AR-FL
MMAA	1.06	0.23	AR-FL
PMM2	0.66	0.23	AR-FL
PPM1D	0.89	0.23	AR-FL
GOLPH3	0.97	0.23	AR-FL
CLCN6	0.61	0.23	AR-FL
PSEN1	0.61	0.23	AR-FL
HN1L	0.62	0.23	AR-FL
SEC61A1	0.66	0.24	AR-FL
TMEM50A	0.89	0.24	AR-FL
SIAH2	0.76	0.24	AR-FL
ANKRD27	0.60	0.24	AR-FL
KIAA0513	0.59	0.24	AR-FL
CHM	0.66	0.24	AR-FL
IMMP1L	0.62	0.24	AR-FL
SLC25A13	0.65	0.24	AR-FL
PSMA6	0.98	0.24	AR-FL
ACLY	0.71	0.24	AR-FL
MYO1C	0.68	0.24	AR-FL
PRDM4	0.65	0.24	AR-FL
SMURF2	0.68	0.24	AR-FL
SACS	0.88	0.24	AR-FL
ARCN1	0.69	0.24	AR-FL
MON1B	0.79	0.25	AR-FL
ATP6V1D	0.75	0.25	AR-FL
TMEM87B	1.49	0.25	AR-FL
PGM3	1.51	0.25	AR-FL
GMNC	1.27	0.25	AR-FL
BCL2L1	0.81	0.25	AR-FL
ZG16B	1.02	0.26	AR-FL
C11orf96	-1.70	-0.02	AR-FL
TCFL5	-0.61	-0.02	AR-FL
DUT	-0.64	-0.02	AR-FL
RIPPLY3	-0.96	-0.02	AR-FL
COL7A1	-1.62	-0.02	AR-FL
OBSL1	-0.67	-0.02	AR-FL
UBAP1L	-0.98	-0.02	AR-FL
GOLT1A	-0.74	-0.02	AR-FL
DYRK4	-0.64	-0.01	AR-FL
AC092835.2	-1.15	-0.01	AR-FL
SOD2	-0.68	-0.01	AR-FL
MPP3	-1.20	-0.01	AR-FL
ZBTB48	-0.59	-0.01	AR-FL
ZNF550	-0.78	-0.01	AR-FL
FAM229A	-0.59	-0.01	AR-FL
TCTN1	-0.73	-0.01	AR-FL
FAM96A	-0.64	-0.01	AR-FL
ZFP36L1	-1.48	0.00	AR-FL
ACACB	-1.42	0.00	AR-FL
MYH7B	-0.87	0.00	AR-FL
MND1	-0.97	0.00	AR-FL
DNAJC19	-0.60	0.00	AR-FL
SLC22A10	-0.02	-2.25	AR-V7
MAB21L3	-0.03	-1.93	AR-V7
GPRC5A	-0.52	-1.70	AR-V7
RIMS1	-0.21	-1.68	AR-V7
TRDN	-0.45	-1.65	AR-V7
GBP3	-0.23	-1.58	AR-V7
ADAMTSL1	-0.27	-1.58	AR-V7
MYO18B	-0.38	-1.53	AR-V7
PTPRK	-0.28	-1.48	AR-V7
RPL21	-0.56	-1.44	AR-V7
BCAS1	-0.23	-1.40	AR-V7
PTPRE	-0.20	-1.38	AR-V7
WNT5A	-0.53	-1.35	AR-V7
IGFL2	-0.23	-1.34	AR-V7
LUZP2	-0.15	-1.32	AR-V7
RASSF6	-0.50	-1.31	AR-V7
ACPP	-0.03	-1.29	AR-V7
SPRY4	-0.57	-1.24	AR-V7
SSTR5	-0.48	-1.24	AR-V7
GDPD1	-0.12	-1.23	AR-V7
SERTAD4	-0.38	-1.22	AR-V7
C10orf10	-0.41	-1.22	AR-V7
C16orf70	-0.25	-1.21	AR-V7
TCERG1L	-0.53	-1.18	AR-V7

TTC12	0.60	0.26	AR-FL
FBXL3	0.64	0.26	AR-FL
TM2D2	0.58	0.26	AR-FL
KCNRG	0.63	0.26	AR-FL
COPB1	0.63	0.26	AR-FL
IMPDH1	1.01	0.27	AR-FL
TESK1	0.63	0.27	AR-FL
VAMP7	3.00	0.27	AR-FL
CASD1	0.63	0.27	AR-FL
MUC20	1.15	0.27	AR-FL
CCDC53	1.25	0.27	AR-FL
C20orf24	0.73	0.27	AR-FL
FZD8	1.58	0.27	AR-FL
SLC25A25	0.76	0.27	AR-FL
TMEM41B	0.75	0.27	AR-FL
SRPR	0.60	0.28	AR-FL
C15orf61	0.97	0.28	AR-FL
STAMBP	0.59	0.28	AR-FL
SLC33A1	0.87	0.28	AR-FL
TMED10	0.71	0.28	AR-FL
SERP1	1.11	0.28	AR-FL
LRRC59	1.02	0.28	AR-FL
MEGF6	1.35	0.28	AR-FL
ZHX1	0.62	0.28	AR-FL
ZNF697	1.13	0.29	AR-FL
ARHGAP26	1.20	0.29	AR-FL
PICALM	0.75	0.29	AR-FL
FMN1	1.63	0.29	AR-FL
LA16c-380H5.3	0.98	0.29	AR-FL
MTM1	0.90	0.29	AR-FL
GORASP2	0.72	0.29	AR-FL
FAM221A	0.71	0.30	AR-FL
ZNF540	0.98	0.30	AR-FL
GNPNAT1	1.05	0.30	AR-FL
EGFR	1.04	0.30	AR-FL
DNAJC3	1.23	0.30	AR-FL
THAP5	0.62	0.30	AR-FL
GOLGA5	0.83	0.30	AR-FL
RBMXL1	0.78	0.30	AR-FL
FHDC1	1.08	0.31	AR-FL
NUMB	0.59	0.31	AR-FL
SEC23B	0.83	0.31	AR-FL
CORO1B	1.26	0.31	AR-FL
PTPN1	0.72	0.31	AR-FL
MIPEP	1.17	0.31	AR-FL
CD2AP	0.65	0.31	AR-FL
DAB2	-0.54	-1.17	AR-V7
RELL1	-0.06	-1.17	AR-V7
TMOD1	-0.56	-1.16	AR-V7
MITF	-0.06	-1.16	AR-V7
DACT2	-0.31	-1.15	AR-V7
TP53I3	-0.30	-1.15	AR-V7
ADRB2	-0.44	-1.15	AR-V7
CCDC146	-0.38	-1.13	AR-V7
ELK3	-0.47	-1.10	AR-V7
PELI1	-0.50	-1.09	AR-V7
ANKRD33B	-0.26	-1.08	AR-V7
SMAD9	-0.49	-1.06	AR-V7
CTD-3088G3.8	-0.28	-1.06	AR-V7
TESK2	-0.32	-1.05	AR-V7
PVRL3	-0.01	-1.05	AR-V7
EYA2	0.00	-1.04	AR-V7
TRIM2	-0.35	-1.03	AR-V7
SLC2A13	-0.07	-1.01	AR-V7
CNTNAP2	-0.25	-1.01	AR-V7
DRAM1	-0.40	-1.00	AR-V7
SLC22A15	-0.36	-0.99	AR-V7
CACNA1D	-0.37	-0.98	AR-V7
NTF3	-0.22	-0.97	AR-V7
SRSF10	-0.24	-0.96	AR-V7
RGPD8	-0.03	-0.96	AR-V7
TRIM16L	-0.58	-0.96	AR-V7
UNC80	-0.50	-0.96	AR-V7
MGLL	-0.22	-0.95	AR-V7
NCEH1	-0.50	-0.95	AR-V7
PSD3	-0.22	-0.95	AR-V7
PAX5	-0.29	-0.95	AR-V7
TOX3	-0.05	-0.94	AR-V7
TTC9	-0.29	-0.94	AR-V7
NKX2-2	-0.49	-0.94	AR-V7
PDE11A	-0.27	-0.93	AR-V7
CABP4	-0.30	-0.93	AR-V7
C1orf115	-0.36	-0.93	AR-V7
TSHZ2	-0.34	-0.93	AR-V7
ADPRM	-0.53	-0.93	AR-V7
L3MBTL3	-0.33	-0.92	AR-V7
RWDD2A	-0.17	-0.91	AR-V7
KRBA2	-0.57	-0.91	AR-V7
KSR1	-0.05	-0.91	AR-V7
APBB2	-0.54	-0.90	AR-V7
APOL6	-0.43	-0.90	AR-V7
ARHGAP42	-0.05	-0.89	AR-V7

SEC22C	0.66	0.31	AR-FL	NRIP1	-0.44	-0.89	AR-V7
KLHDC2	0.60	0.31	AR-FL	SH3RF1	-0.54	-0.89	AR-V7
SLC4A7	0.59	0.31	AR-FL	LFNG	-0.26	-0.89	AR-V7
LRIG1	1.95	0.31	AR-FL	LIN7A	-0.57	-0.88	AR-V7
YOD1	0.75	0.31	AR-FL	C21orf91	-0.33	-0.88	AR-V7
BCAR3	1.30	0.31	AR-FL	GALNT3	-0.56	-0.87	AR-V7
ZNF350	2.27	0.31	AR-FL	PCDHGA8	-0.13	-0.87	AR-V7
MSMB	0.91	0.31	AR-FL	RAB25	-0.51	-0.86	AR-V7
C9orf91	0.98	0.31	AR-FL	ZNF860	-0.41	-0.85	AR-V7
PDIA6	0.77	0.31	AR-FL	UGT1A1	-0.44	-0.85	AR-V7
HCFC2	0.75	0.31	AR-FL	RALGAPA2	-0.15	-0.85	AR-V7
STX5	0.66	0.32	AR-FL	PPP2R3B	-0.56	-0.84	AR-V7
TXNDC11	0.86	0.32	AR-FL	DDX58	-0.48	-0.84	AR-V7
MUC4	0.80	0.32	AR-FL	ARHGAP6	-0.38	-0.84	AR-V7
SLC19A2	1.36	0.32	AR-FL	FAM171A2	-0.48	-0.84	AR-V7
SQLE	0.62	0.32	AR-FL	SLC25A21	-0.46	-0.84	AR-V7
TIGD6	0.80	0.32	AR-FL	SLCO5A1	-0.53	-0.84	AR-V7
SARAF	0.80	0.32	AR-FL	ZC3H12B	-0.51	-0.84	AR-V7
TRIM3	1.48	0.32	AR-FL	HSD17B14	-0.56	-0.83	AR-V7
SMURF1	0.74	0.32	AR-FL	MYO6	-0.15	-0.83	AR-V7
HYOU1	0.83	0.33	AR-FL	POLN	-0.49	-0.82	AR-V7
C6orf52	0.69	0.33	AR-FL	RNF186	-0.36	-0.82	AR-V7
C11orf54	0.65	0.33	AR-FL	NAALADL2	-0.53	-0.81	AR-V7
GUCY1A3	1.23	0.33	AR-FL	TSPAN6	-0.30	-0.81	AR-V7
MTOR	1.55	0.33	AR-FL	TMEM44	-0.44	-0.81	AR-V7
STRN	0.61	0.33	AR-FL	HOXC11	-0.45	-0.81	AR-V7
TM4SF1	2.10	0.33	AR-FL	TTC39C	-0.01	-0.81	AR-V7
TMEM8A	0.62	0.33	AR-FL	RNF128	-0.23	-0.80	AR-V7
SLC35A2	0.65	0.33	AR-FL	ADAMTS1	-0.47	-0.80	AR-V7
SEC22B	0.64	0.33	AR-FL	MEX3B	-0.55	-0.79	AR-V7
MAK	2.58	0.34	AR-FL	MIPOL1	-0.48	-0.78	AR-V7
ARFGAP3	0.97	0.34	AR-FL	C3orf80	-0.04	-0.78	AR-V7
CRISP3	1.91	0.34	AR-FL	CDR2L	-0.16	-0.78	AR-V7
NANS	1.09	0.34	AR-FL	ANKMY2	-0.02	-0.77	AR-V7
NOMO2	0.62	0.34	AR-FL	CLSTN2	-0.52	-0.77	AR-V7
HSPA13	0.98	0.34	AR-FL	MIOS	-0.56	-0.76	AR-V7
ACAD8	1.50	0.34	AR-FL	NBPF10	-0.54	-0.76	AR-V7
BICD2	1.66	0.34	AR-FL	TP53INP1	-0.52	-0.76	AR-V7
TIFA	0.76	0.34	AR-FL	AGMAT	-0.04	-0.76	AR-V7
FKBP2	0.70	0.35	AR-FL	ANKRD29	-0.56	-0.76	AR-V7
COL4A6	1.38	0.35	AR-FL	PRICKLE2	-0.41	-0.75	AR-V7
TIMM23B	0.59	0.35	AR-FL	TBL1Y	-0.39	-0.75	AR-V7
ZNF613	1.26	0.35	AR-FL	SORBS2	-0.22	-0.74	AR-V7
MPZL1	1.07	0.35	AR-FL	ICK	-0.47	-0.74	AR-V7
MESDC2	0.62	0.35	AR-FL	BPIFB1	-0.48	-0.73	AR-V7
SSR1	1.04	0.35	AR-FL	SFRP1	-0.28	-0.73	AR-V7

SEC24C	0.78	0.35	AR-FL	TMEM139	-0.28	-0.73	AR-V7
MYCL	0.99	0.35	AR-FL	FAM122C	-0.49	-0.73	AR-V7
PPP1R13B	0.61	0.35	AR-FL	FBXO25	-0.57	-0.72	AR-V7
C1orf210	0.75	0.35	AR-FL	DOCK4	-0.32	-0.72	AR-V7
PRRC1	0.81	0.35	AR-FL	ZNF280D	-0.58	-0.72	AR-V7
TMED2	0.68	0.35	AR-FL	ITGA5	-0.12	-0.72	AR-V7
HES4	0.64	0.36	AR-FL	SGMS2	-0.49	-0.72	AR-V7
SHROOM2	0.96	0.36	AR-FL	SMYD3	-0.24	-0.72	AR-V7
ABAT	0.59	0.36	AR-FL	MAP4K1	-0.58	-0.72	AR-V7
MRPS35	0.67	0.36	AR-FL	RBPMS	-0.26	-0.71	AR-V7
RSPH9	0.64	0.36	AR-FL	GRAMD1A	-0.49	-0.71	AR-V7
SPCS3	1.23	0.36	AR-FL	CYP4X1	-0.56	-0.71	AR-V7
KLB	1.07	0.36	AR-FL	KLF13	-0.19	-0.71	AR-V7
SPTBN4	0.97	0.36	AR-FL	SETBP1	-0.57	-0.71	AR-V7
SEPP1	2.25	0.36	AR-FL	FBXL7	-0.49	-0.71	AR-V7
UBE2G1	1.20	0.37	AR-FL	DIO1	-0.12	-0.71	AR-V7
MEAF6	1.22	0.37	AR-FL	MB	-0.05	-0.70	AR-V7
BCAP29	1.07	0.37	AR-FL	ADCY1	-0.04	-0.70	AR-V7
UBE2E1	0.59	0.37	AR-FL	ZNF117	-0.31	-0.70	AR-V7
RAB1A	0.66	0.37	AR-FL	UST	-0.38	-0.70	AR-V7
ZNF649	0.66	0.37	AR-FL	CTIF	-0.40	-0.70	AR-V7
MAP7D1	1.13	0.37	AR-FL	CXCR6	-0.53	-0.70	AR-V7
C17orf62	0.68	0.37	AR-FL	IGF2	-0.46	-0.70	AR-V7
STT3A	0.79	0.37	AR-FL	CNTN3	-0.42	-0.70	AR-V7
ZNF518B	0.96	0.37	AR-FL	PAM	-0.13	-0.70	AR-V7
PEA15	0.92	0.37	AR-FL	SNX16	-0.25	-0.70	AR-V7
SNAP23	0.89	0.37	AR-FL	ATP12A	-0.40	-0.69	AR-V7
SHOX2	0.71	0.37	AR-FL	AC013461.1	-0.50	-0.69	AR-V7
RREB1	0.75	0.37	AR-FL	COL4A6	-0.55	-0.69	AR-V7
NOMO3	0.70	0.37	AR-FL	COL9A2	-0.36	-0.69	AR-V7
PTPRJ	0.93	0.37	AR-FL	GDAP1	-0.44	-0.68	AR-V7
CMAS	0.81	0.38	AR-FL	PCDHB16	-0.30	-0.68	AR-V7
VEGFA	1.03	0.38	AR-FL	ARHGEF6	-0.41	-0.68	AR-V7
C18orf8	0.71	0.38	AR-FL	TMCO4	-0.30	-0.68	AR-V7
5-Mar	0.93	0.38	AR-FL	CNTN4	-0.40	-0.68	AR-V7
ELOVL7	3.20	0.38	AR-FL	ZNF532	-0.29	-0.68	AR-V7
GMPPB	1.00	0.38	AR-FL	NFKB2	-0.09	-0.67	AR-V7
ATG13	0.59	0.38	AR-FL	ADSS	-0.43	-0.67	AR-V7
SLC25A37	0.89	0.38	AR-FL	SERPINB6	-0.23	-0.67	AR-V7
ABCC11	1.13	0.38	AR-FL	HELB	-0.52	-0.67	AR-V7
TMEM214	0.73	0.39	AR-FL	RAP1GAP2	-0.49	-0.67	AR-V7
MCPH1	0.73	0.39	AR-FL	TRPV6	-0.15	-0.67	AR-V7
LATS2	0.99	0.39	AR-FL	HEY1	-0.31	-0.67	AR-V7
GLMP	1.63	0.39	AR-FL	ZNF680	-0.37	-0.67	AR-V7
ARF4	1.03	0.39	AR-FL	MCU	-0.21	-0.67	AR-V7
KDELR2	1.08	0.39	AR-FL	ISPD	-0.16	-0.67	AR-V7

DNAJC10	1.47	0.39	AR-FL	TLE2	-0.49	-0.67	AR-V7
POP1	0.62	0.39	AR-FL	ZSWIM4	-0.28	-0.66	AR-V7
OTULIN	1.11	0.39	AR-FL	CTNNAL1	-0.55	-0.66	AR-V7
C16orf46	0.78	0.40	AR-FL	PTPN13	-0.31	-0.66	AR-V7
APP	1.45	0.40	AR-FL	LPAR6	-0.45	-0.66	AR-V7
DERL2	0.85	0.40	AR-FL	6-Sep	-0.55	-0.66	AR-V7
THUMPD1	0.61	0.41	AR-FL	LRP4	-0.29	-0.66	AR-V7
MAP2K4	0.99	0.41	AR-FL	CAPN5	-0.56	-0.65	AR-V7
GOLGA3	0.67	0.41	AR-FL	NCKAP5	-0.47	-0.65	AR-V7
COPG1	0.99	0.41	AR-FL	NR1H3	-0.41	-0.65	AR-V7
CAP1	0.73	0.42	AR-FL	AAMDC	-0.48	-0.65	AR-V7
GREB1	2.93	0.42	AR-FL	GRHL2	-0.16	-0.65	AR-V7
SPRYD7	1.23	0.42	AR-FL	SCAMP1	-0.08	-0.65	AR-V7
SNX32	0.77	0.42	AR-FL	PLS1	-0.32	-0.65	AR-V7
TMED5	0.91	0.42	AR-FL	KIF16B	-0.43	-0.64	AR-V7
FOXO3	0.75	0.42	AR-FL	CEP170	-0.23	-0.64	AR-V7
SYVN1	0.86	0.43	AR-FL	RB1CC1	-0.55	-0.64	AR-V7
ATAD2	0.87	0.43	AR-FL	ACSS1	-0.50	-0.64	AR-V7
SLC25A42	0.68	0.43	AR-FL	SLC27A2	-0.51	-0.64	AR-V7
SLC35B1	0.65	0.43	AR-FL	FGF12	-0.35	-0.64	AR-V7
PDIA3	0.75	0.43	AR-FL	NEDD4L	-0.33	-0.64	AR-V7
TMEM87A	0.91	0.43	AR-FL	ZNF717	-0.53	-0.64	AR-V7
SELK	0.77	0.43	AR-FL	HOXC4	-0.55	-0.64	AR-V7
CYB561D2	0.73	0.43	AR-FL	LIPH	-0.01	-0.64	AR-V7
CDK2AP2	0.68	0.43	AR-FL	FOXP1	-0.35	-0.64	AR-V7
PNKD	0.96	0.43	AR-FL	STX16-NPEPL1	-0.51	-0.64	AR-V7
BET1	0.68	0.43	AR-FL	SCCPDH	-0.46	-0.64	AR-V7
NDFIP2	1.72	0.43	AR-FL	IRF1	-0.55	-0.63	AR-V7
AGAP4	1.63	0.43	AR-FL	B3GNT4	-0.53	-0.63	AR-V7
MARCKS	0.70	0.43	AR-FL	PCDHA10	-0.17	-0.63	AR-V7
TRADD	0.99	0.44	AR-FL	KLLN	-0.57	-0.63	AR-V7
GANC	0.66	0.44	AR-FL	BICC1	-0.44	-0.62	AR-V7
PHF8	0.85	0.44	AR-FL	PKIA	-0.53	-0.62	AR-V7
PARG	0.92	0.44	AR-FL	SSC4D	-0.55	-0.62	AR-V7
CORO2A	1.25	0.44	AR-FL	RAB27B	-0.42	-0.62	AR-V7
PTRH2	0.72	0.44	AR-FL	MIA2	-0.27	-0.62	AR-V7
ZSWIM8	0.59	0.44	AR-FL	GLIS2	-0.52	-0.62	AR-V7
CCPG1	0.79	0.44	AR-FL	SIAH1	-0.21	-0.61	AR-V7
SLC39A6	0.72	0.44	AR-FL	PLEKHF2	-0.14	-0.61	AR-V7
XPNPEP1	0.65	0.44	AR-FL	LRTM2	-0.52	-0.61	AR-V7
RCAN1	0.98	0.45	AR-FL	DOCK9	-0.17	-0.61	AR-V7
CNPY3	0.95	0.45	AR-FL	UTRN	-0.46	-0.61	AR-V7
ZNF532	0.74	0.45	AR-FL	ELF3	-0.06	-0.61	AR-V7
YWHAZ	0.63	0.45	AR-FL	KLHL7	-0.10	-0.61	AR-V7
GFPT1	0.97	0.45	AR-FL	MAP4K4	-0.51	-0.60	AR-V7
FNBP1L	1.11	0.45	AR-FL	HDAC11	-0.55	-0.60	AR-V7

CHP1		0.65	0.45	AR-FL
AFF4		0.75	0.45	AR-FL
OCRL		0.59	0.46	AR-FL
ARSG		1.56	0.46	AR-FL
CAMSAP2		0.88	0.46	AR-FL
RPS6KC1		0.74	0.46	AR-FL
NRG4		1.01	0.46	AR-FL
RNF103		0.70	0.46	AR-FL
PJA2		0.78	0.46	AR-FL
CYP46A1		0.68	0.46	AR-FL
MRPS18A		1.31	0.46	AR-FL
TRIM24		0.60	0.46	AR-FL
SEC61B		0.81	0.46	AR-FL
CCDC40		0.65	0.47	AR-FL
ITPKC		0.81	0.47	AR-FL
MCEE		0.90	0.47	AR-FL
DYNLRB2		0.88	0.47	AR-FL
PPM1K		1.18	0.47	AR-FL
ARID5B		1.32	0.47	AR-FL
ELMO2		1.22	0.47	AR-FL
SPDEF		2.30	0.47	AR-FL
B3GNT2		0.74	0.47	AR-FL
RAB43		0.70	0.47	AR-FL
ZDHHC9		1.05	0.48	AR-FL
ABCD3		0.65	0.48	AR-FL
FBXO42		0.63	0.48	AR-FL
ARHGAP1		0.80	0.48	AR-FL
CREB3		0.73	0.48	AR-FL
MTMR11		1.51	0.48	AR-FL
USO1		0.79	0.48	AR-FL
RAB27A		1.98	0.48	AR-FL
SEC24B		1.32	0.48	AR-FL
RDH11		0.60	0.49	AR-FL
TXNDC5		0.75	0.49	AR-FL
	2-Mar	0.68	0.49	AR-FL
CRACR2A		2.24	0.49	AR-FL
MORC4		0.78	0.49	AR-FL
CRISPLD2		1.46	0.50	AR-FL
COPB2		0.76	0.50	AR-FL
CAPZB		1.27	0.50	AR-FL
SURF4		0.85	0.50	AR-FL
SEC24A		1.01	0.50	AR-FL
CCDC90B		0.60	0.51	AR-FL
MPC2		1.70	0.51	AR-FL
UCN		0.68	0.51	AR-FL
TRAM1		1.02	0.51	AR-FL

EEFSEC	-0.54	-0.60	AR-V7
QPCT	-0.26	-0.60	AR-V7
NRN1	-0.51	-0.60	AR-V7
TMC5	-0.34	-0.60	AR-V7
LBH	-0.16	-0.60	AR-V7
CD2AP	-0.41	-0.60	AR-V7
RNF130	-0.51	-0.60	AR-V7
PERP	-0.52	-0.60	AR-V7
FYCO1	-0.31	-0.60	AR-V7
AMOTL2	-0.55	-0.60	AR-V7
RAB30	-0.33	-0.59	AR-V7
POTEJ	-0.51	-0.59	AR-V7
SYF2	-0.51	-0.59	AR-V7
DDI2	-0.43	-0.59	AR-V7
KCNT1	-0.42	-0.59	AR-V7
NYAP1	-0.01	-0.59	AR-V7
PCDHB13	-0.33	-0.59	AR-V7
PBXIP1	-0.15	-0.59	AR-V7
SIX4	-0.56	-0.59	AR-V7
GAREM	-0.49	-0.59	AR-V7
DENND4C	-0.22	-0.58	AR-V7
KRAS	-0.40	-0.58	AR-V7
PCDHB9	-0.31	-0.58	AR-V7
COQ10A	-0.57	-0.58	AR-V7
FRAT2	-0.15	-0.58	AR-V7
PIGQ	0.44	0.58	AR-V7
DHTKD1	0.44	0.58	AR-V7
PYGB	0.55	0.58	AR-V7
FBXL6	0.49	0.58	AR-V7
ACO2	0.56	0.58	AR-V7
CDCA3	0.13	0.58	AR-V7
NFE2L1	0.45	0.58	AR-V7
MMS22L	0.01	0.58	AR-V7
HIF1A	0.52	0.58	AR-V7
QPCTL	0.46	0.59	AR-V7
LMBR1	0.10	0.59	AR-V7
U2AF1L4	0.12	0.59	AR-V7
MYH14	0.11	0.59	AR-V7
CDK11B	0.50	0.59	AR-V7
BRI3BP	0.38	0.59	AR-V7
SH2D5	0.04	0.59	AR-V7
TST	0.18	0.59	AR-V7
PIP5K1C	0.49	0.59	AR-V7
TTC3	0.24	0.59	AR-V7
ABCD3	0.04	0.59	AR-V7
FAH	0.17	0.59	AR-V7

RALY	0.62	0.51	AR-FL	PLK4	0.09	0.60	AR-V7
MYDGF	0.92	0.51	AR-FL	ARAF	0.21	0.60	AR-V7
FRYL	1.35	0.51	AR-FL	GLUD2	0.37	0.60	AR-V7
CSNK1G3	0.61	0.51	AR-FL	WDFY1	0.44	0.60	AR-V7
ARMCX3	0.77	0.51	AR-FL	MDM1	0.18	0.60	AR-V7
TTN	1.19	0.51	AR-FL	ZNF778	0.56	0.60	AR-V7
AADAT	1.26	0.52	AR-FL	MED30	0.19	0.60	AR-V7
P4HB	0.65	0.52	AR-FL	VTN	0.18	0.60	AR-V7
MYL9	2.24	0.52	AR-FL	TADA2B	0.34	0.60	AR-V7
GBF1	0.69	0.52	AR-FL	KIF18B	0.07	0.60	AR-V7
C9orf72	1.17	0.52	AR-FL	ACAP3	0.43	0.60	AR-V7
PPP1R15B	0.72	0.52	AR-FL	TNFRSF10B	0.40	0.61	AR-V7
APIP	0.68	0.52	AR-FL	TMEM175	0.39	0.61	AR-V7
FBXW11	0.67	0.52	AR-FL	TM2D2	0.52	0.61	AR-V7
PAQR6	1.18	0.52	AR-FL	NCKIPSD	0.10	0.61	AR-V7
MAP3K6	0.92	0.52	AR-FL	ALKBH6	0.33	0.61	AR-V7
EDEM1	1.28	0.52	AR-FL	SLC2A4RG	0.40	0.61	AR-V7
AMACR	1.73	0.53	AR-FL	C9orf142	0.50	0.61	AR-V7
APPBP2	1.95	0.53	AR-FL	VWA5B2	0.47	0.61	AR-V7
NKX3-2	1.29	0.53	AR-FL	ANKFY1	0.46	0.62	AR-V7
C15orf65	1.06	0.53	AR-FL	BTG3	0.26	0.62	AR-V7
SRP19	1.00	0.53	AR-FL	NEMP2	0.01	0.62	AR-V7
RBM24	0.87	0.53	AR-FL	ZNF184	0.40	0.62	AR-V7
SLC30A7	0.91	0.53	AR-FL	ARL3	0.19	0.62	AR-V7
TMCO3	0.61	0.53	AR-FL	RBM47	0.54	0.62	AR-V7
IDH1	1.04	0.54	AR-FL	DCDC2	0.17	0.62	AR-V7
MAGT1	0.61	0.54	AR-FL	PAGR1	0.31	0.62	AR-V7
ANXA5	0.67	0.54	AR-FL	MROH1	0.48	0.62	AR-V7
PDIA4	0.84	0.54	AR-FL	PRAME	0.16	0.62	AR-V7
MAP3K5	0.66	0.54	AR-FL	CCDC86	0.40	0.62	AR-V7
PRKCA	2.58	0.54	AR-FL	TRAF4	0.52	0.62	AR-V7
STARD3NL	0.85	0.54	AR-FL	KLHL36	0.22	0.62	AR-V7
ERP44	0.91	0.54	AR-FL	GYLTL1B	0.07	0.63	AR-V7
GOLGB1	1.04	0.54	AR-FL	RHOC	0.33	0.63	AR-V7
LMAN1	1.18	0.55	AR-FL	ANKRD40	0.14	0.63	AR-V7
SDF2L1	0.89	0.55	AR-FL	TMEM164	0.15	0.63	AR-V7
MCFD2	0.84	0.55	AR-FL	FBXO9	0.22	0.63	AR-V7
ERN1	2.01	0.55	AR-FL	TMEM234	0.34	0.63	AR-V7
DHTKD1	0.66	0.55	AR-FL	WDR13	0.51	0.63	AR-V7
ZNF235	0.84	0.55	AR-FL	PPP4R3A	0.43	0.63	AR-V7
OSTC	0.98	0.55	AR-FL	IQCK	0.50	0.64	AR-V7
CSGALNACT1	5.08	0.55	AR-FL	CCDC157	0.47	0.64	AR-V7
MAGED2	0.59	0.55	AR-FL	ALDH3B2	0.02	0.64	AR-V7
CAMKK2	1.62	0.55	AR-FL	GRIPAP1	0.34	0.64	AR-V7
UFM1	0.60	0.55	AR-FL	TK2	0.48	0.64	AR-V7
SLC50A1	0.68	0.55	AR-FL	FIGNL1	0.44	0.64	AR-V7

FAR2	0.79	0.56	AR-FL	TP73	0.11	0.64	AR-V7
HSPA5	0.69	0.56	AR-FL	ZSCAN1	0.09	0.64	AR-V7
MGLL	0.99	0.56	AR-FL	SLC25A29	0.26	0.64	AR-V7
RPGRIP1	1.03	0.56	AR-FL	RFC2	0.55	0.64	AR-V7
CTTN	0.83	0.56	AR-FL	EZH2	0.44	0.64	AR-V7
SSR2	0.97	0.56	AR-FL	CENPP	0.07	0.64	AR-V7
DFNB31	0.69	0.56	AR-FL	NAIP	0.13	0.64	AR-V7
RNF225	0.85	0.56	AR-FL	NOS1AP	0.49	0.65	AR-V7
HERPUD1	1.05	0.56	AR-FL	PIGBOS1	0.41	0.65	AR-V7
EFNA5	1.31	0.57	AR-FL	DYNLL1	0.57	0.65	AR-V7
FAM118B	0.73	0.57	AR-FL	PPP5C	0.47	0.65	AR-V7
CCNYL1	0.74	0.57	AR-FL	VRK1	0.52	0.65	AR-V7
CSNK1A1	0.72	0.57	AR-FL	C4orf33	0.30	0.65	AR-V7
SEC61G	0.90	0.57	AR-FL	GALK1	0.12	0.65	AR-V7
FBXW2	1.16	0.57	AR-FL	SRPK2	0.11	0.66	AR-V7
FOXO1	1.16	0.57	AR-FL	AMOTL1	0.39	0.66	AR-V7
SEC11C	1.30	0.57	AR-FL	LMNB1	0.48	0.66	AR-V7
TMEM120A	0.62	0.57	AR-FL	STRADA	0.43	0.66	AR-V7
BTN3A1	1.04	0.58	AR-FL	ZNHIT1	0.53	0.66	AR-V7
YIPF5	0.66	0.58	AR-FL	GOT2	0.41	0.66	AR-V7
WDYHV1	1.22	0.58	AR-FL	ULK4	0.16	0.66	AR-V7
TMED7	0.88	0.58	Both Isoforms	PRKX	0.47	0.66	AR-V7
AKAP1	0.64	0.58	Both Isoforms	TMSB10	0.42	0.66	AR-V7
ADH1C	3.95	0.58	Both Isoforms	AADAT	0.52	0.66	AR-V7
CKMT1B	0.81	0.59	Both Isoforms	C12orf4	0.20	0.66	AR-V7
SEL1L	0.60	0.59	Both Isoforms	CHPT1	0.53	0.66	AR-V7
MGAT2	0.69	0.59	Both Isoforms	SKA1	0.27	0.66	AR-V7
BET1L	0.64	0.59	Both Isoforms	TMEM39A	0.53	0.67	AR-V7
COQ10B	0.70	0.59	Both Isoforms	CDKN1C	0.39	0.67	AR-V7
RIMBP3	0.96	0.59	Both Isoforms	IQCG	0.39	0.67	AR-V7
MAP9	1.23	0.59	Both Isoforms	TMEM144	0.47	0.67	AR-V7
GMPPA	1.10	0.59	Both Isoforms	KLHL26	0.27	0.67	AR-V7
ZNF432	0.78	0.60	Both Isoforms	GLUD1	0.56	0.67	AR-V7
OPN1SW	1.33	0.60	Both Isoforms	NCAPG2	0.40	0.67	AR-V7

GOSR2	0.63	0.60	Both Isoforms	C19orf71	0.15	0.67	AR-V7
OAT	0.89	0.60	Both Isoforms	LPIN1	0.27	0.67	AR-V7
KIF13B	1.23	0.60	Both Isoforms	PQLC1	0.27	0.68	AR-V7
SLC10A7	1.68	0.60	Both Isoforms	OPLAH	0.35	0.68	AR-V7
PIGH	0.66	0.60	Both Isoforms	SPA17	0.50	0.69	AR-V7
ARFIP2	0.97	0.60	Both Isoforms	SLC43A3	0.30	0.69	AR-V7
SDCBP	0.94	0.60	Both Isoforms	DUSP3	0.49	0.69	AR-V7
PTPRM	1.99	0.61	Both Isoforms	SUV39H1	0.45	0.70	AR-V7
KLF4	1.14	0.61	Both Isoforms	RBM34	0.06	0.70	AR-V7
TCAF1	1.56	0.61	Both Isoforms	TMSB4Y	0.30	0.70	AR-V7
MCCC2	0.99	0.61	Both Isoforms	MYCBP	0.56	0.70	AR-V7
HIST1H2BE	0.60	0.61	Both Isoforms	PXYLP1	0.00	0.70	AR-V7
ZNF587	0.75	0.61	Both Isoforms	TRMT2B	0.52	0.70	AR-V7
CALU	1.19	0.61	Both Isoforms	THOC2	0.55	0.71	AR-V7
ANXA4	0.59	0.61	Both Isoforms	CDC14B	0.29	0.71	AR-V7
CHPF2	0.61	0.61	Both Isoforms	TPM1	0.46	0.71	AR-V7
HES6	1.98	0.61	Both Isoforms	PPP1R16A	0.45	0.71	AR-V7
MAFK	0.71	0.61	Both Isoforms	MOK	0.18	0.72	AR-V7
TVP23C	0.84	0.61	Both Isoforms	CBS	0.20	0.72	AR-V7
LRG1	0.84	0.62	Both Isoforms	FAM217B	0.08	0.72	AR-V7
FKBP11	0.96	0.62	Both Isoforms	FLNC	0.26	0.72	AR-V7
WDR44	0.91	0.62	Both Isoforms	NCAPH2	0.38	0.72	AR-V7
SLC25A16	0.78	0.62	Both Isoforms	CAPN2	0.08	0.72	AR-V7
TRIM13	1.18	0.62	Both Isoforms	ZGRF1	0.11	0.72	AR-V7
GPR75	0.61	0.62	Both Isoforms	SRSF2	0.28	0.73	AR-V7

AKAP13	1.05	0.62	Both Isoforms	TRAIP	0.17	0.73	AR-V7
AP1S2	0.73	0.62	Both Isoforms	NASP	0.05	0.73	AR-V7
PDXDC1	0.64	0.62	Both Isoforms	TXNDC16	0.17	0.73	AR-V7
RRBP1	1.08	0.63	Both Isoforms	EIF4EBP2	0.29	0.73	AR-V7
RTN2	0.70	0.63	Both Isoforms	SPATA33	0.54	0.73	AR-V7
FABP3	0.64	0.63	Both Isoforms	C1orf226	0.57	0.73	AR-V7
FAM19A4	1.64	0.63	Both Isoforms	HIST1H1E	0.34	0.73	AR-V7
GOLGA2	0.88	0.63	Both Isoforms	HHEX	0.13	0.73	AR-V7
SEC13	0.69	0.63	Both Isoforms	NRGN	0.07	0.73	AR-V7
ELOVL1	0.70	0.64	Both Isoforms	FBXO36	0.48	0.74	AR-V7
GOLGA4	1.01	0.64	Both Isoforms	TRIP13	0.29	0.74	AR-V7
EXOC8	0.82	0.64	Both Isoforms	SERF1B	0.10	0.74	AR-V7
KLF5	2.88	0.64	Both Isoforms	STARD10	0.52	0.74	AR-V7
FAM46C	0.65	0.64	Both Isoforms	ENTPD3	0.32	0.74	AR-V7
DQX1	0.78	0.64	Both Isoforms	TMPPE	0.54	0.75	AR-V7
ID2	0.80	0.65	Both Isoforms	TBCEL	0.22	0.75	AR-V7
FEM1C	0.66	0.65	Both Isoforms	SIVA1	0.45	0.75	AR-V7
HARBI1	0.70	0.65	Both Isoforms	SPRYD7	0.35	0.75	AR-V7
ADPRM	1.33	0.65	Both Isoforms	RGMA	0.04	0.75	AR-V7
USP38	0.69	0.65	Both Isoforms	KREMEN2	0.40	0.75	AR-V7
PDS5B	0.84	0.65	Both Isoforms	WDHD1	0.53	0.76	AR-V7
INHBC	1.37	0.65	Both Isoforms	TICRR	0.04	0.76	AR-V7
LIG4	0.61	0.66	Both Isoforms	NRSN2	0.30	0.76	AR-V7
ADAM9	1.00	0.66	Both Isoforms	INPP1	0.12	0.76	AR-V7
GOLT1B	0.76	0.66	Both Isoforms	NARFL	0.47	0.77	AR-V7

USP35	0.59	0.66	Both Isoforms	NUDT1	0.57	0.77	AR-V7
MON2	0.64	0.66	Both Isoforms	ZMAT3	0.46	0.77	AR-V7
TCEAL3	0.70	0.66	Both Isoforms	KIF24	0.17	0.77	AR-V7
TRAFD1	0.82	0.66	Both Isoforms	AKAP1	0.49	0.77	AR-V7
KLF7	0.59	0.67	Both Isoforms	MBD2	0.52	0.77	AR-V7
PICK1	0.66	0.67	Both Isoforms	PXMP2	0.53	0.77	AR-V7
LMLN	0.60	0.67	Both Isoforms	ADGRB2	0.11	0.78	AR-V7
PPIB	0.78	0.67	Both Isoforms	RNASEH2A	0.37	0.78	AR-V7
C17orf107	0.62	0.67	Both Isoforms	TMEM201	0.26	0.78	AR-V7
TIMM10B	0.85	0.67	Both Isoforms	TBC1D20	0.48	0.78	AR-V7
C19orf48	0.94	0.67	Both Isoforms	C15orf59	0.11	0.78	AR-V7
TMED9	1.03	0.68	Both Isoforms	ZNF527	0.15	0.78	AR-V7
ENTPD7	1.08	0.69	Both Isoforms	CNN2	0.57	0.79	AR-V7
ORMDL2	0.73	0.69	Both Isoforms	RAB15	0.20	0.80	AR-V7
TRMT6	0.61	0.69	Both Isoforms	GPR137C	0.44	0.80	AR-V7
RHOBTB2	1.00	0.70	Both Isoforms	ASB16	0.47	0.80	AR-V7
HUS1	0.90	0.70	Both Isoforms	PMEPA1	0.48	0.80	AR-V7
CUL4B	0.84	0.70	Both Isoforms	DPCD	0.34	0.80	AR-V7
ENTPD4	0.61	0.70	Both Isoforms	NPHP1	0.15	0.80	AR-V7
FRY	1.56	0.70	Both Isoforms	POLQ	0.18	0.80	AR-V7
MPHOSPH9	1.66	0.70	Both Isoforms	SERPINF1	0.35	0.80	AR-V7
LYPLAL1	1.04	0.71	Both Isoforms	RFC4	0.27	0.80	AR-V7
FXYD3	1.10	0.71	Both Isoforms	PC	0.45	0.80	AR-V7
SYP	0.60	0.71	Both Isoforms	CDCA4	0.03	0.81	AR-V7
CMC2	1.26	0.71	Both Isoforms	DSN1	0.51	0.81	AR-V7

LIMCH1	1.46	0.71	Both Isoforms	RBL1	0.30	0.81	AR-V7
RPS6KA3	0.92	0.71	Both Isoforms	MCM2	0.42	0.81	AR-V7
FBXO16	0.65	0.71	Both Isoforms	RFC3	0.27	0.81	AR-V7
TMEM39A	0.85	0.71	Both Isoforms	CDCA5	0.18	0.81	AR-V7
PLPP5	1.39	0.71	Both Isoforms	CUX2	0.42	0.82	AR-V7
NPC1	1.51	0.71	Both Isoforms	ERVK3-1	0.07	0.82	AR-V7
FAM69A	1.47	0.71	Both Isoforms	FKBP1C	0.21	0.82	AR-V7
MANF	1.00	0.71	Both Isoforms	NOMO3	0.12	0.82	AR-V7
ZNF449	0.94	0.72	Both Isoforms	PITPNM3	0.14	0.82	AR-V7
PLEKHM3	0.87	0.72	Both Isoforms	DDTL	0.30	0.82	AR-V7
AFAP1L2	1.21	0.72	Both Isoforms	8-Sep	0.26	0.82	AR-V7
C2orf76	1.40	0.72	Both Isoforms	DNAH9	0.04	0.82	AR-V7
FZD5	2.48	0.72	Both Isoforms	POLD3	0.49	0.83	AR-V7
PFKFB2	1.08	0.73	Both Isoforms	TRABD	0.12	0.83	AR-V7
CPEB3	1.76	0.73	Both Isoforms	CENPU	0.40	0.83	AR-V7
FAM13A	1.31	0.73	Both Isoforms	HRH3	0.10	0.83	AR-V7
HSD17B4	0.65	0.73	Both Isoforms	UBXN11	0.17	0.83	AR-V7
SERF1B	0.93	0.74	Both Isoforms	EPAS1	0.07	0.84	AR-V7
WDFY2	0.83	0.74	Both Isoforms	ARHGAP31	0.34	0.84	AR-V7
SNAPC1	0.80	0.74	Both Isoforms	CCDC69	0.40	0.84	AR-V7
ANKRD37	2.13	0.74	Both Isoforms	KANK2	0.27	0.85	AR-V7
TG	4.73	0.74	Both Isoforms	TOE1	0.33	0.86	AR-V7
RAB29	0.59	0.74	Both Isoforms	NCOR1	0.21	0.86	AR-V7
KCTD21	0.85	0.74	Both Isoforms	SPC25	0.10	0.86	AR-V7
ALDH6A1	0.76	0.75	Both Isoforms	FANCI	0.47	0.87	AR-V7

ZBTB1	0.77	0.75	Both Isoforms	ZNF652	0.54	0.87	AR-V7
SGK3	1.25	0.75	Both Isoforms	RIT1	0.43	0.87	AR-V7
CREB3L2	1.08	0.75	Both Isoforms	RFC5	0.55	0.87	AR-V7
TBC1D8	2.05	0.75	Both Isoforms	STXBP5	0.42	0.87	AR-V7
ELF2	0.87	0.75	Both Isoforms	ATAD5	0.39	0.88	AR-V7
WWTR1	3.69	0.75	Both Isoforms	RGPD6	0.02	0.88	AR-V7
PPP2CB	1.08	0.75	Both Isoforms	CCND1	0.58	0.88	AR-V7
YY2	0.78	0.75	Both Isoforms	SHISA3	0.15	0.88	AR-V7
RASSF3	1.29	0.75	Both Isoforms	CENPK	0.39	0.89	AR-V7
PRR34	0.98	0.76	Both Isoforms	FANCD2	0.52	0.89	AR-V7
ZNF587B	0.91	0.76	Both Isoforms	BAIAP3	0.15	0.89	AR-V7
ALOX12B	2.09	0.76	Both Isoforms	TMEM60	0.08	0.89	AR-V7
POLD4	0.70	0.76	Both Isoforms	COMMD8	0.51	0.89	AR-V7
MTMR9	2.23	0.77	Both Isoforms	GTPBP2	0.49	0.90	AR-V7
DISP2	0.69	0.77	Both Isoforms	CTNNBIP1	0.25	0.90	AR-V7
HES2	2.84	0.77	Both Isoforms	RPS6KL1	0.39	0.91	AR-V7
WDR1	0.74	0.77	Both Isoforms	C17orf53	0.46	0.92	AR-V7
HGD	1.77	0.77	Both Isoforms	DNA2	0.23	0.93	AR-V7
TRO	0.58	0.77	Both Isoforms	ACADM	0.29	0.93	AR-V7
VIMP	1.25	0.77	Both Isoforms	CKAP2	0.39	0.93	AR-V7
CLDN12	0.69	0.78	Both Isoforms	BRIP1	0.27	0.93	AR-V7
GLYATL1	1.77	0.78	Both Isoforms	CENPI	0.42	0.93	AR-V7
HIST1H4H	0.72	0.78	Both Isoforms	CCBL1	0.46	0.94	AR-V7
PACSIN2	0.94	0.78	Both Isoforms	TONSL	0.51	0.94	AR-V7
GLYATL1P3	1.58	0.78	Both Isoforms	FAM46C	0.31	0.94	AR-V7

FRK	1.88	0.79	Both Isoforms	KRTCAP2	0.09	0.95	AR-V7
DNAJC1	0.66	0.79	Both Isoforms	GAMT	0.43	0.95	AR-V7
HERC4	0.73	0.79	Both Isoforms	VCL	0.42	0.95	AR-V7
FAM177A1	1.13	0.79	Both Isoforms	NPHP4	0.24	0.96	AR-V7
CDR2	0.60	0.80	Both Isoforms	MSX1	0.42	0.97	AR-V7
GCNT1	1.31	0.80	Both Isoforms	POLD1	0.29	0.97	AR-V7
GNRH1	0.85	0.80	Both Isoforms	XRCC2	0.33	0.97	AR-V7
IL17RA	0.60	0.81	Both Isoforms	KNTC1	0.07	0.98	AR-V7
ZNF589	0.97	0.81	Both Isoforms	MAGI2	0.11	0.98	AR-V7
KLHL36	0.84	0.81	Both Isoforms	ROR1	0.45	0.99	AR-V7
SCD	0.62	0.81	Both Isoforms	EYA1	0.18	0.99	AR-V7
HIST1H2BD	0.63	0.81	Both Isoforms	KCNC4	0.06	1.00	AR-V7
HMGCR	1.55	0.82	Both Isoforms	KIAA0101	0.42	1.00	AR-V7
CEP162	0.83	0.82	Both Isoforms	APOD	0.14	1.00	AR-V7
ZFP36	0.74	0.82	Both Isoforms	STARD13	0.31	1.00	AR-V7
HIBCH	0.90	0.82	Both Isoforms	CBX4	0.58	1.00	AR-V7
COX18	0.78	0.82	Both Isoforms	SPTB	0.40	1.01	AR-V7
WDR93	0.67	0.82	Both Isoforms	PANX2	0.39	1.01	AR-V7
BMPR1A	1.74	0.82	Both Isoforms	ACYP1	0.23	1.01	AR-V7
DUSP4	1.34	0.82	Both Isoforms	DHFR	0.43	1.02	AR-V7
MOB4	0.66	0.82	Both Isoforms	HMGA2	0.40	1.02	AR-V7
THRB	1.27	0.83	Both Isoforms	H2AFX	0.56	1.02	AR-V7
ECI2	1.54	0.83	Both Isoforms	SORT1	0.55	1.02	AR-V7
FAM174B	1.79	0.83	Both Isoforms	BRICD5	0.48	1.02	AR-V7
WTIP	0.67	0.84	Both Isoforms	IRF7	0.50	1.03	AR-V7

NEK3	0.96	0.84	Both Isoforms	PTK2B	0.03	1.04	AR-V7
SC5D	1.30	0.84	Both Isoforms	CHKB	0.35	1.05	AR-V7
RABAC1	0.81	0.84	Both Isoforms	SAMD15	0.22	1.06	AR-V7
DHCR24	2.18	0.84	Both Isoforms	NT5DC3	0.43	1.06	AR-V7
ILK	0.59	0.85	Both Isoforms	MLEC	0.57	1.06	AR-V7
LAMC1	2.29	0.85	Both Isoforms	EME1	0.27	1.06	AR-V7
ACOT9	0.65	0.85	Both Isoforms	DAGLA	0.10	1.06	AR-V7
EDEM2	0.95	0.85	Both Isoforms	MAT2A	0.26	1.07	AR-V7
KCNAB1	1.42	0.85	Both Isoforms	TRIM36	0.13	1.07	AR-V7
SSR3	1.35	0.85	Both Isoforms	RAD51AP1	0.30	1.07	AR-V7
LRRC63	1.36	0.85	Both Isoforms	SCD5	0.55	1.08	AR-V7
CRELD2	0.88	0.85	Both Isoforms	SPC24	0.32	1.08	AR-V7
PLEKHG2	1.55	0.86	Both Isoforms	HSD17B4	0.34	1.10	AR-V7
CD4	0.88	0.86	Both Isoforms	AKAP7	0.42	1.10	AR-V7
ABCA5	1.04	0.86	Both Isoforms	VANGL1	0.47	1.10	AR-V7
NOX5	0.98	0.86	Both Isoforms	RECQL4	0.53	1.10	AR-V7
EPPK1	1.20	0.86	Both Isoforms	GALNT2	0.35	1.11	AR-V7
WWC1	0.90	0.86	Both Isoforms	TMPO	0.18	1.11	AR-V7
ACADM	0.84	0.86	Both Isoforms	SMARCA1	0.26	1.12	AR-V7
NAAA	0.67	0.86	Both Isoforms	FSIP1	0.15	1.12	AR-V7
C14orf28	1.01	0.87	Both Isoforms	B3GALNT1	0.50	1.15	AR-V7
PDIA5	1.57	0.87	Both Isoforms	FTH1	0.43	1.15	AR-V7
SYNJ1	1.72	0.87	Both Isoforms	CSF1	0.54	1.16	AR-V7
MINPP1	0.78	0.88	Both Isoforms	TTC21A	0.50	1.17	AR-V7
VGF	1.50	0.88	Both Isoforms	DUSP2	0.32	1.18	AR-V7

RAB4A	1.69	0.88	Both Isoforms	MARS2	0.02	1.18	AR-V7
FBN1	0.86	0.89	Both Isoforms	PLOD2	0.52	1.19	AR-V7
ABCC1	1.91	0.89	Both Isoforms	E2F7	0.12	1.19	AR-V7
TNFAIP3	2.66	0.89	Both Isoforms	TASP1	0.28	1.19	AR-V7
DNAJB14	0.69	0.89	Both Isoforms	EP400NL	0.44	1.19	AR-V7
VMP1	1.17	0.90	Both Isoforms	LIMS1	0.38	1.26	AR-V7
DLEC1	1.16	0.90	Both Isoforms	THOC5	0.21	1.26	AR-V7
MKRN2OS	1.09	0.90	Both Isoforms	VLDLR	0.29	1.26	AR-V7
JMJD6	0.68	0.90	Both Isoforms	IFI6	0.36	1.27	AR-V7
KCTD9	1.21	0.90	Both Isoforms	AMOT	0.45	1.28	AR-V7
HIF1A	0.58	0.90	Both Isoforms	CBLL1	0.38	1.28	AR-V7
PPM1A	0.66	0.91	Both Isoforms	DMRT2	0.30	1.29	AR-V7
DNM1L	1.44	0.91	Both Isoforms	SLC44A5	0.37	1.30	AR-V7
ZNF841	1.11	0.91	Both Isoforms	C1orf21	0.29	1.31	AR-V7
KLK15	1.51	0.91	Both Isoforms	CYSTM1	0.03	1.31	AR-V7
ZCCHC6	1.92	0.91	Both Isoforms	OSGIN2	0.19	1.32	AR-V7
ASAH1	0.90	0.91	Both Isoforms	GPER1	0.37	1.32	AR-V7
PCYT2	1.01	0.91	Both Isoforms	LRRFIP2	0.55	1.33	AR-V7
PSME4	0.65	0.91	Both Isoforms	MAP1A	0.54	1.33	AR-V7
HIPK2	1.06	0.91	Both Isoforms	NRBP2	0.08	1.35	AR-V7
C16orf71	0.73	0.92	Both Isoforms	HELLS	0.55	1.35	AR-V7
HMGXB3	1.59	0.92	Both Isoforms	TYMS	0.47	1.36	AR-V7
TBC1D4	1.15	0.92	Both Isoforms	JAKMIP1	0.05	1.37	AR-V7
KIFC3	0.72	0.92	Both Isoforms	MT1E	0.06	1.38	AR-V7
ZC3H12A	0.60	0.92	Both Isoforms	RASL10A	0.16	1.40	AR-V7

PFKFB1	0.85	0.92	Both Isoforms	USP51	0.21	1.43	AR-V7
ACE2	0.96	0.93	Both Isoforms	IZUMO4	0.07	1.44	AR-V7
B3GNT9	0.81	0.93	Both Isoforms	SSX2IP	0.57	1.44	AR-V7
FNDC3B	0.80	0.93	Both Isoforms	BOC	0.46	1.45	AR-V7
SLCO2A1	1.06	0.93	Both Isoforms	PTP4A1	0.39	1.45	AR-V7
PPP1CB	1.16	0.95	Both Isoforms	VGF	0.06	1.46	AR-V7
PALLD	0.63	0.95	Both Isoforms	ESCO2	0.45	1.46	AR-V7
ATG101	0.99	0.95	Both Isoforms	CDH23	0.00	1.47	AR-V7
AMHR2	0.68	0.95	Both Isoforms	SMIM1	0.46	1.47	AR-V7
ZYX	0.84	0.95	Both Isoforms	TRIM35	0.38	1.48	AR-V7
CKAP4	0.66	0.95	Both Isoforms	TMEM56	0.10	1.48	AR-V7
TSPAN13	1.00	0.95	Both Isoforms	AKAP17A	0.06	1.50	AR-V7
MLPH	1.83	0.95	Both Isoforms	ATOH8	0.16	1.51	AR-V7
SMIM14	1.09	0.96	Both Isoforms	CEBPB	0.09	1.54	AR-V7
ZCCHC9	0.90	0.96	Both Isoforms	SBSPON	0.00	1.55	AR-V7
CAP2	1.37	0.96	Both Isoforms	MT1X	0.58	1.55	AR-V7
GOT2	0.66	0.96	Both Isoforms	HBEGF	0.10	1.56	AR-V7
FAM151B	1.14	0.96	Both Isoforms	CCNE1	0.58	1.66	AR-V7
ANKH	2.26	0.97	Both Isoforms	ZBTB8B	0.58	1.66	AR-V7
MBD2	0.59	0.97	Both Isoforms	PIM3	0.25	1.67	AR-V7
BMPR1B	1.64	0.97	Both Isoforms	ZNF589	0.53	1.68	AR-V7
SLC39A10	1.59	0.97	Both Isoforms	VEPH1	0.24	1.68	AR-V7
DNAJB9	2.01	0.98	Both Isoforms	TIGD3	0.29	1.69	AR-V7
SLC25A33	0.82	0.98	Both Isoforms	FOXD4	0.23	1.76	AR-V7
HK1	1.11	0.98	Both Isoforms	LRCH2	0.47	1.85	AR-V7

AGR2	0.63	0.99	Both Isoforms	GRAMD2	0.16	1.86	AR-V7
CYB5A	0.70	0.99	Both Isoforms	AC138969.4	0.51	1.87	AR-V7
ZNF652	0.93	0.99	Both Isoforms	CITED4	0.56	1.92	AR-V7
ZNF737	0.60	0.99	Both Isoforms	CDK6	0.12	1.93	AR-V7
SEC24D	1.87	0.99	Both Isoforms	SOWAHA	0.21	1.95	AR-V7
DAPK3	0.95	0.99	Both Isoforms	GALNT9	0.43	1.98	AR-V7
FICD	1.84	1.00	Both Isoforms	ZNF18	0.44	2.06	AR-V7
TMEM263	0.80	1.00	Both Isoforms	NPY	0.00	2.07	AR-V7
EXTL2	0.74	1.00	Both Isoforms	LRRC75A	0.52	2.08	AR-V7
SPAG17	2.70	1.00	Both Isoforms	AASS	0.37	2.10	AR-V7
EPDR1	1.42	1.00	Both Isoforms	UVRAG	0.14	2.13	AR-V7
SLC25A30	0.72	1.00	Both Isoforms	MT2A	0.56	2.16	AR-V7
LDLR	1.39	1.00	Both Isoforms	TCAF2	0.49	2.28	AR-V7
ZNF233	1.07	1.01	Both Isoforms	PPM1B	0.43	2.46	AR-V7
NMD3	0.64	1.01	Both Isoforms	RAB9B	0.13	2.54	AR-V7
MAPK8IP2	0.75	1.01	Both Isoforms	ZBED3	0.28	2.70	AR-V7
INPP4B	3.05	1.01	Both Isoforms	HPCAL4	0.38	2.83	AR-V7
ZNF552	1.11	1.01	Both Isoforms	SDC1	0.27	2.96	AR-V7
ERO1B	0.60	1.01	Both Isoforms	SERPINA7	0.30	3.16	AR-V7
HSP90B1	0.86	1.01	Both Isoforms	GTPBP6	0.44	4.00	AR-V7
FAM214B	0.88	1.01	Both Isoforms	PCDH7	0.55	4.35	AR-V7
CAPN13	0.76	1.02	Both Isoforms	NPTX2	0.00	4.40	AR-V7
FAM103A1	1.00	1.02	Both Isoforms	SMOC1	0.44	4.44	AR-V7
MERTK	2.43	1.02	Both Isoforms	NEXN	0.37	4.61	AR-V7
NFKBIA	1.47	1.02	Both Isoforms	ADAMTS8	0.43	6.34	AR-V7

VCL	1.30	1.02	Both Isoforms	HTATIP2	0.70	0.00	AR-FL
CCDC153	1.01	1.03	Both Isoforms	EXOC6	0.67	0.00	AR-FL
SOX9	2.88	1.03	Both Isoforms	ACOT9	0.85	0.01	AR-FL
TRPM4	1.11	1.03	Both Isoforms	MYADML2	1.04	0.01	AR-FL
SLA2	2.63	1.03	Both Isoforms	ITGA10	1.10	0.01	AR-FL
PLA1A	2.13	1.04	Both Isoforms	CPT1A	0.59	0.01	AR-FL
RGPD3	1.35	1.04	Both Isoforms	FRAT1	1.02	0.01	AR-FL
NFIB	1.44	1.04	Both Isoforms	FXYD3	0.61	0.01	AR-FL
ORC5	1.21	1.04	Both Isoforms	DLGAP4	0.62	0.01	AR-FL
CRLS1	1.20	1.04	Both Isoforms	CCDC126	0.94	0.02	AR-FL
EDEM3	1.49	1.04	Both Isoforms	TNFSF13	0.69	0.02	AR-FL
TMTC2	1.79	1.04	Both Isoforms	SPG21	0.69	0.02	AR-FL
BBX	1.01	1.04	Both Isoforms	TLN2	0.88	0.02	AR-FL
PPP1R13L	0.82	1.04	Both Isoforms	GLMP	1.20	0.02	AR-FL
CHRNB1	0.73	1.05	Both Isoforms	RHPN2	1.49	0.02	AR-FL
SMS	2.52	1.05	Both Isoforms	FLYWCH2	0.72	0.02	AR-FL
PHACTR2	1.01	1.05	Both Isoforms	BCL3	1.75	0.02	AR-FL
C1orf122	0.91	1.05	Both Isoforms	HDAC7	0.59	0.02	AR-FL
MYADM	1.08	1.06	Both Isoforms	SHB	0.95	0.03	AR-FL
ELK4	1.19	1.06	Both Isoforms	ACTL10	0.88	0.03	AR-FL
LSS	0.89	1.06	Both Isoforms	NOL3	0.92	0.03	AR-FL
LAMA3	1.13	1.07	Both Isoforms	STK10	0.80	0.03	AR-FL
PDZRN3	1.48	1.08	Both Isoforms	CD82	1.08	0.03	AR-FL
MYL12A	1.02	1.08	Both Isoforms	MYCL	1.05	0.03	AR-FL
RFTN2	0.65	1.08	Both Isoforms	C8orf33	0.63	0.03	AR-FL

AC021106.1	0.76	1.09	Both Isoforms	ST3GAL6	0.93	0.03	AR-FL
NAMPT	1.57	1.10	Both Isoforms	CYTH1	0.69	0.03	AR-FL
GPR37	1.35	1.10	Both Isoforms	ZNF614	0.72	0.03	AR-FL
TBKBP1	0.59	1.10	Both Isoforms	STS	0.75	0.04	AR-FL
ODC1	2.22	1.10	Both Isoforms	CALM2	0.64	0.04	AR-FL
EVA1B	1.03	1.11	Both Isoforms	ACBD3	0.60	0.04	AR-FL
NOSTRIN	1.48	1.11	Both Isoforms	SGPP1	0.83	0.04	AR-FL
LGMN	0.76	1.11	Both Isoforms	GLIPR2	0.96	0.04	AR-FL
TLX2	1.00	1.11	Both Isoforms	FAM53C	0.63	0.04	AR-FL
CFAP99	0.66	1.11	Both Isoforms	ANAPC15	0.69	0.04	AR-FL
CYR61	1.13	1.12	Both Isoforms	GNAI2	0.64	0.04	AR-FL
PHOSPHO1	0.88	1.12	Both Isoforms	ZNF217	1.04	0.04	AR-FL
CXCR4	4.12	1.12	Both Isoforms	PLOD1	0.66	0.04	AR-FL
OCEL1	0.59	1.12	Both Isoforms	EML1	0.76	0.04	AR-FL
SGK223	3.46	1.12	Both Isoforms	ADAT3	0.82	0.05	AR-FL
KDM7A	0.69	1.13	Both Isoforms	RABEP1	0.83	0.05	AR-FL
LRRC8A	0.78	1.13	Both Isoforms	RAP1GAP	1.22	0.05	AR-FL
NXF2	2.47	1.13	Both Isoforms	CDC42EP3	1.93	0.05	AR-FL
H1F0	0.88	1.13	Both Isoforms	STRN	0.82	0.05	AR-FL
ERO1A	1.03	1.14	Both Isoforms	ALOX5	1.21	0.05	AR-FL
HSD17B11	2.05	1.14	Both Isoforms	RGS9BP	1.06	0.05	AR-FL
TPM4	0.82	1.14	Both Isoforms	CCNJ	0.65	0.06	AR-FL
STARD13	0.92	1.14	Both Isoforms	MYL12B	0.65	0.06	AR-FL
RANBP2	0.71	1.14	Both Isoforms	FAM127C	0.72	0.06	AR-FL
ACTB	0.63	1.15	Both Isoforms	TTYH3	0.94	0.06	AR-FL

IDI1	1.13	1.16	Both Isoforms	PRDM10	0.71	0.06	AR-FL
RHBDF1	0.86	1.16	Both Isoforms	ZDHHC21	0.71	0.06	AR-FL
VLDLR	1.50	1.16	Both Isoforms	GALNT7	0.97	0.06	AR-FL
PRKCD	0.92	1.16	Both Isoforms	INTS8	0.59	0.06	AR-FL
CFAP221	2.28	1.16	Both Isoforms	PCTP	0.66	0.06	AR-FL
TSPYL2	0.62	1.16	Both Isoforms	EPHB3	1.28	0.06	AR-FL
CDYL2	2.48	1.17	Both Isoforms	BAG3	0.68	0.07	AR-FL
HIST4H4	0.98	1.17	Both Isoforms	NQO2	0.71	0.07	AR-FL
RAB3B	2.04	1.18	Both Isoforms	NPTXR	1.15	0.07	AR-FL
NEDD4L	1.23	1.19	Both Isoforms	P4HA1	0.84	0.07	AR-FL
MALT1	1.76	1.19	Both Isoforms	DYNC1LI1	0.62	0.07	AR-FL
WDR41	0.65	1.19	Both Isoforms	ITGA3	1.41	0.07	AR-FL
EEF2K	1.08	1.20	Both Isoforms	ZNF443	0.96	0.07	AR-FL
MAPK11	0.93	1.20	Both Isoforms	TMEM63A	0.91	0.07	AR-FL
SMN1	0.77	1.21	Both Isoforms	CYB5R4	0.67	0.07	AR-FL
ITGAV	1.70	1.21	Both Isoforms	ZBTB7C	0.78	0.07	AR-FL
PLPP6	1.60	1.21	Both Isoforms	LRTOMT	0.58	0.07	AR-FL
KRT18	1.89	1.21	Both Isoforms	SNAP91	1.40	0.08	AR-FL
EIF2AK3	1.13	1.21	Both Isoforms	GPR180	0.63	0.08	AR-FL
TNFRSF10B	1.49	1.21	Both Isoforms	CLEC11A	0.62	0.08	AR-FL
RRAS	1.04	1.22	Both Isoforms	WWTR1	1.15	0.08	AR-FL
PTGES3L	0.95	1.22	Both Isoforms	ALDH1A3	2.82	0.08	AR-FL
FMO4	1.09	1.22	Both Isoforms	MMEL1	0.68	0.08	AR-FL
SORD	2.21	1.22	Both Isoforms	SLC37A1	0.60	0.08	AR-FL
TCTN2	0.65	1.23	Both Isoforms	COL4A3BP	0.64	0.08	AR-FL

HERC3	2.75	1.23	Both Isoforms	LHFPL2	0.81	0.08	AR-FL
ARRDC1	1.29	1.23	Both Isoforms	KDM6B	0.95	0.08	AR-FL
LRRC46	0.74	1.23	Both Isoforms	FAM174B	0.68	0.08	AR-FL
ERLEC1	1.18	1.23	Both Isoforms	RNF24	0.83	0.08	AR-FL
PPFIBP2	2.02	1.24	Both Isoforms	CNDP2	0.75	0.08	AR-FL
GPR137C	0.73	1.24	Both Isoforms	ME1	0.99	0.08	AR-FL
TMEM139	0.77	1.24	Both Isoforms	CDS1	0.79	0.09	AR-FL
PREX2	1.08	1.24	Both Isoforms	TBK1	0.60	0.09	AR-FL
CDC14B	1.56	1.24	Both Isoforms	ZG16B	0.67	0.09	AR-FL
SLC16A6	3.54	1.24	Both Isoforms	ATP8B1	0.84	0.09	AR-FL
PGM2	1.30	1.25	Both Isoforms	TMEM206	0.89	0.09	AR-FL
SAR1B	1.02	1.25	Both Isoforms	PM20D2	0.76	0.09	AR-FL
PACS1	2.13	1.26	Both Isoforms	BVES	1.09	0.09	AR-FL
TP53INP2	0.62	1.26	Both Isoforms	S100P	2.51	0.09	AR-FL
C6orf201	1.65	1.26	Both Isoforms	ACAA1	0.79	0.09	AR-FL
NT5DC3	1.34	1.26	Both Isoforms	TRIM68	0.84	0.09	AR-FL
KDELR3	1.83	1.27	Both Isoforms	SCUBE1	1.51	0.09	AR-FL
RPN1	0.77	1.27	Both Isoforms	RPGR	0.58	0.10	AR-FL
SHROOM3	1.52	1.28	Both Isoforms	NFKBIE	1.09	0.10	AR-FL
TNFRSF19	2.24	1.28	Both Isoforms	MTHFR	0.61	0.10	AR-FL
DNAL1	0.60	1.29	Both Isoforms	SPTBN2	0.69	0.10	AR-FL
B4GALT1	0.76	1.29	Both Isoforms	ARMC5	0.67	0.10	AR-FL
DBI	1.71	1.30	Both Isoforms	ABCC4	1.60	0.10	AR-FL
LDB3	0.79	1.30	Both Isoforms	HSD11B2	0.64	0.10	AR-FL
SLC26A2	1.36	1.30	Both Isoforms	LARP4B	0.58	0.10	AR-FL

EFCAB12	2.48	1.30	Both Isoforms	TMED3	0.81	0.10	AR-FL
CEBPD	0.91	1.30	Both Isoforms	NEK5	0.99	0.10	AR-FL
KIF5C	0.93	1.30	Both Isoforms	TVP23B	1.37	0.10	AR-FL
MSMO1	0.90	1.31	Both Isoforms	NCOR2	0.61	0.10	AR-FL
CNKSR2	1.13	1.31	Both Isoforms	FSTL1	1.10	0.10	AR-FL
C1orf116	2.35	1.31	Both Isoforms	GFPT1	0.66	0.10	AR-FL
BHLHE40	1.14	1.31	Both Isoforms	TINF2	0.61	0.10	AR-FL
CFAP57	0.76	1.31	Both Isoforms	SNTB2	0.76	0.10	AR-FL
GRIN3A	1.82	1.31	Both Isoforms	ZNF765	0.73	0.11	AR-FL
IGF1	2.60	1.31	Both Isoforms	COPB1	0.67	0.11	AR-FL
AASS	1.16	1.32	Both Isoforms	CBLC	0.59	0.11	AR-FL
HIST1H4B	0.71	1.33	Both Isoforms	MLKL	1.36	0.11	AR-FL
FBXO38	1.43	1.33	Both Isoforms	KAT2B	1.24	0.11	AR-FL
PIAS1	1.38	1.33	Both Isoforms	UBE2G1	0.71	0.11	AR-FL
STK17B	2.44	1.33	Both Isoforms	FAM21A	0.86	0.11	AR-FL
ZNF18	1.39	1.34	Both Isoforms	CAMKK2	0.64	0.11	AR-FL
RARB	1.13	1.34	Both Isoforms	ZNF850	0.65	0.12	AR-FL
GATSL3	0.63	1.34	Both Isoforms	ZNF799	0.81	0.12	AR-FL
KLF15	1.57	1.34	Both Isoforms	NDFIP2	1.26	0.12	AR-FL
AGAP2	2.50	1.35	Both Isoforms	PCDHGB4	0.80	0.12	AR-FL
TRANK1	0.90	1.35	Both Isoforms	CCDC40	0.68	0.12	AR-FL
TNFAIP8	1.87	1.35	Both Isoforms	CXADR	0.88	0.12	AR-FL
PYGB	0.63	1.35	Both Isoforms	LRP3	1.13	0.12	AR-FL
DHRS2	0.82	1.36	Both Isoforms	PAQR5	0.89	0.12	AR-FL
IFT57	0.76	1.37	Both Isoforms	PARM1	2.22	0.12	AR-FL

TMEM100	8.24	1.37	Both Isoforms	EIF2B4	0.79	0.12	AR-FL
CYP3A43	1.45	1.37	Both Isoforms	PAK1IP1	0.60	0.12	AR-FL
FAM114A1	0.60	1.38	Both Isoforms	FAM120AOS	0.65	0.12	AR-FL
ZBTB10	2.27	1.38	Both Isoforms	TMEM79	1.94	0.13	AR-FL
POPDC2	0.64	1.38	Both Isoforms	PRR19	0.73	0.13	AR-FL
CHKA	0.99	1.38	Both Isoforms	RDH11	0.65	0.13	AR-FL
CNTN3	1.31	1.39	Both Isoforms	PRLR	0.74	0.13	AR-FL
ID4	1.37	1.39	Both Isoforms	DHDH	1.23	0.13	AR-FL
PNPLA8	1.17	1.40	Both Isoforms	RIMBP3	1.59	0.13	AR-FL
CDH15	1.05	1.40	Both Isoforms	LRRC8B	0.61	0.13	AR-FL
DISP1	1.71	1.42	Both Isoforms	SLC9A2	0.71	0.13	AR-FL
ERVK3-1	0.98	1.43	Both Isoforms	MAPK6	1.11	0.13	AR-FL
TBRG1	1.91	1.43	Both Isoforms	ZDHHC9	0.85	0.13	AR-FL
KLK4	1.53	1.45	Both Isoforms	MESP1	1.39	0.13	AR-FL
SCAP	1.33	1.45	Both Isoforms	PPP2R2D	0.63	0.13	AR-FL
ANGPTL4	0.58	1.48	Both Isoforms	GOLGA5	0.80	0.13	AR-FL
CCDC6	0.66	1.48	Both Isoforms	PSEN2	0.77	0.13	AR-FL
SLC38A2	0.99	1.48	Both Isoforms	SLC16A3	1.21	0.13	AR-FL
LIN7B	1.69	1.49	Both Isoforms	SLAIN2	0.88	0.13	AR-FL
PLPP1	2.27	1.49	Both Isoforms	PDCD10	0.67	0.13	AR-FL
COL23A1	0.84	1.50	Both Isoforms	MYBPC1	0.80	0.13	AR-FL
FERMT2	1.21	1.51	Both Isoforms	CAP1	0.70	0.14	AR-FL
LDLRAD3	1.58	1.52	Both Isoforms	C20orf196	1.09	0.14	AR-FL
SLC2A12	1.57	1.53	Both Isoforms	RANBP3L	1.53	0.14	AR-FL
ARAP2	1.44	1.55	Both Isoforms	USP6NL	0.74	0.14	AR-FL

PDLIM5	1.75	1.55	Both Isoforms	ZBTB42	0.69	0.14	AR-FL
GBP1	3.67	1.55	Both Isoforms	LRIG1	1.62	0.14	AR-FL
ATP1A1	1.24	1.55	Both Isoforms	CTC-534A2.2	0.63	0.14	AR-FL
UNC80	2.25	1.55	Both Isoforms	STRN3	0.60	0.14	AR-FL
CBLL1	1.60	1.56	Both Isoforms	TAX1BP3	0.89	0.14	AR-FL
FDFT1	1.14	1.56	Both Isoforms	ZNF143	0.60	0.14	AR-FL
RHOBTB3	0.86	1.58	Both Isoforms	TM9SF3	0.71	0.14	AR-FL
RALGPS2	1.06	1.59	Both Isoforms	CSK	0.79	0.14	AR-FL
KCNH6	2.41	1.60	Both Isoforms	RABEP2	1.47	0.14	AR-FL
CASC10	2.07	1.60	Both Isoforms	PGM3	0.82	0.14	AR-FL
ATP8B2	1.28	1.61	Both Isoforms	RAB43	1.39	0.14	AR-FL
PDE8B	0.62	1.61	Both Isoforms	ZMPSTE24	0.82	0.15	AR-FL
HIST2H2BE	0.72	1.62	Both Isoforms	PPM1E	1.01	0.15	AR-FL
TLR6	0.63	1.62	Both Isoforms	FZD9	2.01	0.15	AR-FL
CADPS2	2.87	1.63	Both Isoforms	CCDC149	0.64	0.15	AR-FL
C17orf58	0.59	1.64	Both Isoforms	TNFAIP3	1.20	0.15	AR-FL
SH3D21	2.59	1.64	Both Isoforms	DHX15	0.66	0.15	AR-FL
GLYATL2	2.40	1.64	Both Isoforms	EGF	1.69	0.15	AR-FL
ANG	1.97	1.64	Both Isoforms	CDCP1	0.63	0.15	AR-FL
UNC13D	0.79	1.66	Both Isoforms	ADARB2	0.96	0.15	AR-FL
WIPI1	2.80	1.67	Both Isoforms	PRKCH	1.58	0.15	AR-FL
ATF3	0.85	1.67	Both Isoforms	KMT2C	0.70	0.16	AR-FL
C1orf21	2.18	1.67	Both Isoforms	GGT1	0.91	0.16	AR-FL
PCSK5	2.62	1.68	Both Isoforms	MBLAC1	0.70	0.16	AR-FL
ATP8B3	1.52	1.68	Both Isoforms	RAB2A	0.61	0.16	AR-FL

TSC22D1	1.99	1.68	Both Isoforms	GLP2R	0.99	0.16	AR-FL
JAG1	1.39	1.68	Both Isoforms	BORCS6	0.89	0.16	AR-FL
OSBPL8	1.27	1.70	Both Isoforms	FZD5	2.03	0.16	AR-FL
KANSL1L	0.82	1.71	Both Isoforms	PRICKLE3	0.76	0.16	AR-FL
MYO1E	1.53	1.72	Both Isoforms	MAP9	0.58	0.16	AR-FL
SWT1	2.34	1.72	Both Isoforms	TMEM165	0.60	0.16	AR-FL
PLA2G5	2.21	1.73	Both Isoforms	VPS37B	0.61	0.16	AR-FL
CENPN	3.63	1.74	Both Isoforms	VANGL2	1.60	0.16	AR-FL
ZNF578	0.60	1.74	Both Isoforms	PROSER1	0.61	0.16	AR-FL
LPAR3	1.93	1.74	Both Isoforms	CDK2AP1	0.82	0.16	AR-FL
LRRFIP2	1.58	1.74	Both Isoforms	GALNT18	0.71	0.16	AR-FL
LAT2	3.01	1.74	Both Isoforms	MTMR9	1.32	0.16	AR-FL
HPX	0.97	1.76	Both Isoforms	REEP3	0.79	0.17	AR-FL
LPXN	1.35	1.76	Both Isoforms	OTULIN	0.69	0.17	AR-FL
CITED2	0.79	1.77	Both Isoforms	USP33	0.64	0.17	AR-FL
EPSTI1	1.98	1.78	Both Isoforms	MDFI	1.28	0.17	AR-FL
DLX2	1.46	1.78	Both Isoforms	SLC51A	0.65	0.17	AR-FL
HMGCS1	1.70	1.80	Both Isoforms	ESPN	0.69	0.17	AR-FL
NXF2B	3.42	1.80	Both Isoforms	WDR37	0.94	0.17	AR-FL
GJC3	0.85	1.81	Both Isoforms	TPD52	1.01	0.17	AR-FL
ITGA1	1.33	1.83	Both Isoforms	ACACA	0.66	0.17	AR-FL
GLUL	1.26	1.84	Both Isoforms	DHRS3	0.61	0.17	AR-FL
ROPN1L	0.71	1.84	Both Isoforms	AK6	0.58	0.17	AR-FL
ARHGEF37	0.68	1.85	Both Isoforms	ECH1	0.64	0.17	AR-FL
PSCA	0.71	1.86	Both Isoforms	SRMS	0.73	0.18	AR-FL

GSN	1.02	1.86	Both Isoforms	SEPHS2	0.67	0.18	AR-FL
HACD2	0.71	1.87	Both Isoforms	GALNT12	1.00	0.18	AR-FL
KCND2	1.86	1.87	Both Isoforms	ARHGEF16	0.61	0.18	AR-FL
CADM2	3.27	1.87	Both Isoforms	MANSC1	0.93	0.18	AR-FL
TBC1D1	1.89	1.87	Both Isoforms	ZNF579	0.65	0.18	AR-FL
NR4A1	2.17	1.88	Both Isoforms	LENG1	0.59	0.18	AR-FL
TMEM164	1.34	1.88	Both Isoforms	TRIB3	0.60	0.19	AR-FL
SPATA6L	1.47	1.88	Both Isoforms	GPR108	0.69	0.19	AR-FL
SDCBP2	1.41	1.88	Both Isoforms	ATP2A3	1.40	0.19	AR-FL
ANKRD29	1.74	1.89	Both Isoforms	GCLM	0.72	0.19	AR-FL
ABCC4	3.67	1.90	Both Isoforms	PSMD7	0.65	0.19	AR-FL
SLC41A1	2.44	1.90	Both Isoforms	CLDN7	0.95	0.19	AR-FL
MAOA	1.11	1.91	Both Isoforms	YIPF3	0.79	0.19	AR-FL
FAM189A2	1.88	1.92	Both Isoforms	IGF2R	0.62	0.19	AR-FL
NAT1	1.94	1.92	Both Isoforms	JPH1	1.10	0.19	AR-FL
DUSP10	0.77	1.93	Both Isoforms	PIN4	0.69	0.19	AR-FL
CLGN	1.41	1.96	Both Isoforms	HOXC6	0.96	0.20	AR-FL
FASN	1.27	1.96	Both Isoforms	PHACTR4	0.61	0.20	AR-FL
FGD4	2.47	1.98	Both Isoforms	NLK	0.64	0.20	AR-FL
PPIC	0.89	1.99	Both Isoforms	PDLIM4	2.85	0.20	AR-FL
FADS2	1.03	1.99	Both Isoforms	C9orf91	0.90	0.20	AR-FL
NKX3-1	3.25	1.99	Both Isoforms	RALGAPB	0.58	0.20	AR-FL
KLF6	1.62	1.99	Both Isoforms	SSPO	1.21	0.20	AR-FL
PPP2R3B	2.12	1.99	Both Isoforms	CCDC47	0.61	0.20	AR-FL
TRIM35	0.70	1.99	Both Isoforms	RELL2	2.11	0.20	AR-FL

RNF148	3.50	1.99	Both Isoforms	PSMA6	0.65	0.20	AR-FL
CYP2U1	2.83	2.00	Both Isoforms	RTN4R	0.60	0.20	AR-FL
TPM1	1.56	2.00	Both Isoforms	PPDPF	0.58	0.21	AR-FL
PVRL3	1.47	2.00	Both Isoforms	CRISP2	1.34	0.21	AR-FL
GLUD2	1.05	2.00	Both Isoforms	CRNKL1	0.87	0.21	AR-FL
ETS2	0.65	2.01	Both Isoforms	MAGEA12	0.69	0.21	AR-FL
ABHD2	2.51	2.03	Both Isoforms	TDG	0.60	0.21	AR-FL
DNAH7	1.50	2.04	Both Isoforms	CCDC22	0.61	0.21	AR-FL
PGLYRP2	2.89	2.04	Both Isoforms	TUBB6	0.84	0.21	AR-FL
PPFIBP1	1.99	2.04	Both Isoforms	ARFGAP3	0.69	0.21	AR-FL
CACNG4	4.95	2.05	Both Isoforms	BCCIP	0.71	0.21	AR-FL
NSDHL	0.63	2.05	Both Isoforms	SLC35F2	0.79	0.21	AR-FL
LPIN1	1.04	2.06	Both Isoforms	KIF21A	1.21	0.21	AR-FL
CNN2	1.50	2.06	Both Isoforms	MPHOSPH9	0.90	0.21	AR-FL
FAM110B	1.73	2.06	Both Isoforms	NAGA	0.58	0.22	AR-FL
IRS1	0.88	2.07	Both Isoforms	IQCD	0.86	0.22	AR-FL
INSIG1	1.45	2.08	Both Isoforms	PACS1	0.92	0.22	AR-FL
CHRNE	0.86	2.08	Both Isoforms	TMEM41B	0.81	0.22	AR-FL
C3orf58	2.80	2.08	Both Isoforms	PLPP5	0.69	0.22	AR-FL
TSKU	1.59	2.09	Both Isoforms	LRFN3	0.73	0.22	AR-FL
NPR2	2.28	2.09	Both Isoforms	AK4	0.72	0.22	AR-FL
MICAL1	4.37	2.10	Both Isoforms	HMOX2	0.88	0.22	AR-FL
TCAF2	2.95	2.11	Both Isoforms	SNAP23	0.59	0.22	AR-FL
AHNAK	1.41	2.11	Both Isoforms	MRPS24	0.75	0.22	AR-FL
CROT	1.08	2.11	Both Isoforms	UBE2J2	0.63	0.22	AR-FL

FOXD4L1	0.84	2.12	Both Isoforms	BMPR1A	0.62	0.22	AR-FL
CDKN1C	1.23	2.13	Both Isoforms	ATP1B3	0.80	0.22	AR-FL
PLOD2	1.13	2.13	Both Isoforms	EPHX1	0.77	0.23	AR-FL
TMEFF2	0.63	2.14	Both Isoforms	ACOT1	1.16	0.23	AR-FL
PARVA	1.27	2.14	Both Isoforms	SLC26A11	0.60	0.23	AR-FL
SP6	0.65	2.15	Both Isoforms	ARMC7	0.84	0.23	AR-FL
C1orf168	1.13	2.16	Both Isoforms	PDZD11	0.60	0.23	AR-FL
HOMER2	2.21	2.17	Both Isoforms	GSE1	0.66	0.23	AR-FL
ZBTB16	2.02	2.17	Both Isoforms	RNF185	0.74	0.23	AR-FL
TXNDC16	1.13	2.17	Both Isoforms	MYO5C	1.02	0.23	AR-FL
STYK1	1.32	2.18	Both Isoforms	TNFRSF10A	0.92	0.23	AR-FL
GLRX	0.61	2.19	Both Isoforms	ZBTB7A	0.61	0.23	AR-FL
MAF	5.07	2.20	Both Isoforms	DCAF6	0.94	0.23	AR-FL
PRICKLE2	1.34	2.22	Both Isoforms	FAM114A1	0.62	0.23	AR-FL
DNAH3	0.78	2.22	Both Isoforms	NQO1	1.13	0.23	AR-FL
ROR1	1.24	2.23	Both Isoforms	GALK2	0.64	0.23	AR-FL
LONRF1	2.33	2.23	Both Isoforms	ARAP3	1.11	0.23	AR-FL
FHL2	1.47	2.24	Both Isoforms	SLC17A5	1.67	0.23	AR-FL
HSPA2	1.63	2.24	Both Isoforms	BLOC1S3	0.69	0.23	AR-FL
HERC5	1.94	2.25	Both Isoforms	FBXL8	0.63	0.24	AR-FL
ASRGL1	2.20	2.26	Both Isoforms	NSUN5	0.58	0.24	AR-FL
KRT8	2.35	2.27	Both Isoforms	SLC2A9	1.22	0.24	AR-FL
FADS1	0.93	2.29	Both Isoforms	SMPD2	1.19	0.24	AR-FL
PTCRA	4.70	2.29	Both Isoforms	SELT	0.78	0.24	AR-FL
CDKL1	1.48	2.30	Both Isoforms	SPRYD3	0.60	0.24	AR-FL

SLC31A2	0.62	2.30	Both Isoforms	XRCC4	0.77	0.24	AR-FL
CD163L1	0.93	2.32	Both Isoforms	HN1L	0.89	0.24	AR-FL
ACSM1	2.56	2.33	Both Isoforms	NEBL	0.80	0.24	AR-FL
SH3TC2	1.02	2.34	Both Isoforms	NDEL1	0.74	0.24	AR-FL
ANXA2	2.97	2.34	Both Isoforms	NACAD	1.61	0.24	AR-FL
TRIM36	1.57	2.38	Both Isoforms	ALG3	0.60	0.24	AR-FL
MMRN2	3.37	2.39	Both Isoforms	SLC1A5	1.19	0.24	AR-FL
MYH15	1.51	2.44	Both Isoforms	WBP2	0.59	0.24	AR-FL
RHOU	3.32	2.47	Both Isoforms	GRHL3	3.07	0.24	AR-FL
NEK10	1.29	2.48	Both Isoforms	TARDBP	0.58	0.24	AR-FL
RHOB	0.73	2.50	Both Isoforms	KRT14	1.90	0.24	AR-FL
EGR1	1.02	2.50	Both Isoforms	ARTN	1.24	0.24	AR-FL
IQUB	2.04	2.51	Both Isoforms	HERC3	1.72	0.24	AR-FL
SDK1	4.17	2.51	Both Isoforms	HPCAL1	0.74	0.24	AR-FL
NTN4	0.98	2.52	Both Isoforms	ZFAND4	1.06	0.24	AR-FL
AKAP17A	2.47	2.56	Both Isoforms	NOP10	0.76	0.24	AR-FL
FAM83E	1.57	2.57	Both Isoforms	LMO7	0.58	0.25	AR-FL
KLK3	3.56	2.57	Both Isoforms	MOB4	0.74	0.25	AR-FL
PER1	1.77	2.59	Both Isoforms	PELO	0.64	0.25	AR-FL
SERPIND1	2.16	2.59	Both Isoforms	PCDHB2	0.66	0.25	AR-FL
ANK1	2.78	2.60	Both Isoforms	EFNA4	1.81	0.25	AR-FL
C16orf54	5.68	2.60	Both Isoforms	SEC31A	0.64	0.25	AR-FL
ARMC12	2.64	2.62	Both Isoforms	KDM5C	0.61	0.25	AR-FL
CCDC160	2.71	2.64	Both Isoforms	TPST2	1.43	0.25	AR-FL
MBOAT2	2.84	2.65	Both Isoforms	TEP1	1.08	0.25	AR-FL

SAT1	2.33	2.65	Both Isoforms	MARK2	0.66	0.25	AR-FL
CDKN2B	1.49	2.69	Both Isoforms	ORAI1	0.94	0.25	AR-FL
KRT6C	2.59	2.69	Both Isoforms	PAK4	0.66	0.25	AR-FL
EAF2	3.89	2.70	Both Isoforms	EFR3A	1.18	0.26	AR-FL
ITGA11	2.29	2.71	Both Isoforms	TMEM223	0.74	0.26	AR-FL
ASIP	1.78	2.71	Both Isoforms	MRPL27	0.69	0.26	AR-FL
SPSB1	0.96	2.72	Both Isoforms	5-Mar	0.81	0.26	AR-FL
GLUD1	1.74	2.72	Both Isoforms	AKT1S1	0.62	0.26	AR-FL
SH2D3A	0.92	2.72	Both Isoforms	LIMK2	0.59	0.26	AR-FL
PAPLN	0.77	2.73	Both Isoforms	DEXI	0.63	0.26	AR-FL
MYO1B	2.20	2.73	Both Isoforms	CACFD1	0.80	0.26	AR-FL
SLITRK6	2.29	2.75	Both Isoforms	S1PR3	1.90	0.26	AR-FL
PTPN21	2.53	2.76	Both Isoforms	MRPS18A	0.72	0.26	AR-FL
IGF1R	3.24	2.76	Both Isoforms	BCL2L12	1.02	0.26	AR-FL
PHLDB2	4.28	2.76	Both Isoforms	PRKCA	0.99	0.26	AR-FL
ALPK2	3.85	2.77	Both Isoforms	YRDC	0.64	0.26	AR-FL
PNMA1	2.04	2.77	Both Isoforms	CRELD1	0.86	0.26	AR-FL
BTG1	0.72	2.79	Both Isoforms	PSMD13	0.75	0.26	AR-FL
ELOVL5	2.34	2.79	Both Isoforms	FBXW10	1.68	0.26	AR-FL
GADD45B	1.05	2.82	Both Isoforms	SCAND1	1.09	0.26	AR-FL
SPATA13	1.16	2.84	Both Isoforms	ERH	0.59	0.26	AR-FL
CBWD1	2.40	2.85	Both Isoforms	WSB2	0.97	0.27	AR-FL
LOX	4.24	2.86	Both Isoforms	SIPA1L3	0.63	0.27	AR-FL
PMEPA1	3.39	2.86	Both Isoforms	RAB11A	0.94	0.27	AR-FL
CRYAB	0.78	2.88	Both Isoforms	DNAJA4	1.03	0.27	AR-FL

RASD1	2.66	2.89	Both Isoforms	APOF	0.71	0.27	AR-FL
SLC1A2	0.92	2.89	Both Isoforms	BPGM	0.84	0.27	AR-FL
FAM105A	4.44	2.92	Both Isoforms	MAP7D1	1.44	0.27	AR-FL
TMPRSS2	4.58	2.96	Both Isoforms	MTMR6	0.67	0.27	AR-FL
NPNT	1.57	2.97	Both Isoforms	NFIB	0.58	0.27	AR-FL
B2M	1.53	2.99	Both Isoforms	FAM173B	0.60	0.27	AR-FL
MAP2	1.92	2.99	Both Isoforms	HIPK1	0.61	0.27	AR-FL
ANGPT2	5.37	3.01	Both Isoforms	PNKD	1.28	0.27	AR-FL
POTEF	3.39	3.07	Both Isoforms	ERGIC2	0.98	0.27	AR-FL
LAMA1	3.65	3.09	Both Isoforms	MED8	0.60	0.27	AR-FL
CAPN2	0.95	3.09	Both Isoforms	SNRPA1	0.59	0.27	AR-FL
GNMT	3.59	3.20	Both Isoforms	APMAP	0.89	0.27	AR-FL
SLC45A3	3.71	3.29	Both Isoforms	BRD2	0.67	0.27	AR-FL
AZGP1	2.43	3.32	Both Isoforms	PIEZO1	0.60	0.27	AR-FL
WDR46	0.64	3.34	Both Isoforms	SLC7A6	0.68	0.27	AR-FL
POTEJ	2.26	3.35	Both Isoforms	VTI1A	0.62	0.27	AR-FL
APOD	1.27	3.35	Both Isoforms	LPCAT3	0.89	0.27	AR-FL
POTEI	1.76	3.35	Both Isoforms	BNIP3	0.90	0.28	AR-FL
SCNN1G	0.75	3.38	Both Isoforms	PSMD3	0.63	0.28	AR-FL
SNCG	3.53	3.39	Both Isoforms	COA6	0.60	0.28	AR-FL
TMPRSS11E	2.08	3.39	Both Isoforms	SLC10A7	0.66	0.28	AR-FL
NNMT	3.49	3.40	Both Isoforms	ZNF428	0.70	0.28	AR-FL
POTEE	3.34	3.40	Both Isoforms	ZDHHC16	1.00	0.28	AR-FL
PXDC1	2.49	3.45	Both Isoforms	TXNDC9	0.80	0.28	AR-FL
TIPARP	2.08	3.46	Both Isoforms	MAN1A2	0.67	0.28	AR-FL

F2RL1	1.18	3.46	Both Isoforms	RRAS	1.40	0.28	AR-FL
MEIS1	1.53	3.47	Both Isoforms	USP4	0.58	0.28	AR-FL
UGT2B11	3.31	3.49	Both Isoforms	EXOC3L1	0.75	0.28	AR-FL
SNAI2	5.73	3.49	Both Isoforms	ZFPL1	0.78	0.29	AR-FL
LIFR	3.35	3.49	Both Isoforms	MPG	0.63	0.29	AR-FL
KL	4.93	3.52	Both Isoforms	TTI2	0.73	0.29	AR-FL
CUX2	4.17	3.52	Both Isoforms	FAM89B	0.76	0.29	AR-FL
STK39	2.75	3.54	Both Isoforms	MYADM	0.64	0.29	AR-FL
SOCS2	2.60	3.55	Both Isoforms	GSR	0.88	0.29	AR-FL
TGFBR2	1.69	3.55	Both Isoforms	LAMC1	1.59	0.29	AR-FL
NDRG1	4.52	3.56	Both Isoforms	ANXA7	0.62	0.29	AR-FL
FOXG1	2.32	3.64	Both Isoforms	STK17B	0.93	0.29	AR-FL
KLK2	4.55	3.68	Both Isoforms	SRXN1	0.61	0.29	AR-FL
C8orf4	2.90	3.70	Both Isoforms	LPAR3	1.26	0.29	AR-FL
IRS2	2.80	3.70	Both Isoforms	ELOVL1	0.66	0.29	AR-FL
XKR5	3.94	3.80	Both Isoforms	WBP1L	0.70	0.29	AR-FL
NEXN	1.57	3.81	Both Isoforms	RNF26	0.66	0.29	AR-FL
ACSL3	3.64	3.84	Both Isoforms	SLC25A16	0.73	0.29	AR-FL
CYP11A1	3.28	3.88	Both Isoforms	KIAA0100	0.72	0.29	AR-FL
FAM107A	3.11	3.89	Both Isoforms	PEPD	0.74	0.29	AR-FL
AFF3	4.07	3.89	Both Isoforms	EIF4E	0.66	0.29	AR-FL
TRIM49D2	4.11	3.90	Both Isoforms	KDELR1	0.88	0.30	AR-FL
IL31RA	2.30	3.92	Both Isoforms	DTWD2	1.04	0.30	AR-FL
PTPRN2	2.17	3.94	Both Isoforms	DENND1C	0.63	0.30	AR-FL
KRT19	2.57	3.95	Both Isoforms	GABARAP	0.59	0.30	AR-FL

CECR6	3.94	3.96	Both Isoforms	CBR1	0.60	0.30	AR-FL
HES1	0.90	4.03	Both Isoforms	CCDC186	0.66	0.30	AR-FL
NPPC	5.58	4.04	Both Isoforms	RABIF	0.73	0.30	AR-FL
ITGB8	1.30	4.05	Both Isoforms	ASAH1	0.94	0.30	AR-FL
ACTA2	3.04	4.06	Both Isoforms	CCNYL1	1.57	0.30	AR-FL
ATRNL1	2.45	4.06	Both Isoforms	CKMT1A	1.07	0.30	AR-FL
KCNMA1	2.98	4.06	Both Isoforms	SLC25A32	0.65	0.30	AR-FL
HIST1H1E	3.24	4.10	Both Isoforms	PCMT1	0.85	0.30	AR-FL
CTNNA2	5.53	4.11	Both Isoforms	DCTPP1	0.75	0.30	AR-FL
MAGEA11	2.48	4.16	Both Isoforms	GPKOW	0.60	0.30	AR-FL
UGT2B28	5.82	4.20	Both Isoforms	SPARC	3.36	0.30	AR-FL
TNS4	3.66	4.23	Both Isoforms	PRAF2	0.58	0.30	AR-FL
DNER	1.44	4.23	Both Isoforms	TMEM19	0.61	0.30	AR-FL
MAOB	1.54	4.39	Both Isoforms	PCDHB3	1.02	0.30	AR-FL
FOXD4	3.06	4.42	Both Isoforms	AGTRAP	0.91	0.30	AR-FL
ACTG2	4.01	4.45	Both Isoforms	CMAS	0.61	0.30	AR-FL
FKBP5	4.72	4.51	Both Isoforms	TRMT12	0.80	0.31	AR-FL
SIPA1L2	1.91	4.56	Both Isoforms	CCDC159	0.90	0.31	AR-FL
CA4	1.86	4.61	Both Isoforms	KLHL25	0.79	0.31	AR-FL
TBX15	6.07	4.62	Both Isoforms	CREM	0.66	0.31	AR-FL
ASB9	1.67	4.64	Both Isoforms	RAB27A	1.51	0.31	AR-FL
ADAMTS8	0.96	4.70	Both Isoforms	GFM1	0.83	0.31	AR-FL
ST6GALNAC1	4.83	4.77	Both Isoforms	CHCHD1	0.62	0.31	AR-FL
MYH7	1.77	4.85	Both Isoforms	CRY1	1.18	0.31	AR-FL
SULT1B1	2.16	4.92	Both Isoforms	RHOU	1.35	0.31	AR-FL

EDN1	2.28	4.95	Both Isoforms	MED27	0.83	0.31	AR-FL
DLX1	2.77	5.01	Both Isoforms	UQCC3	0.83	0.31	AR-FL
ERRFI1	2.99	5.03	Both Isoforms	INPPL1	0.62	0.31	AR-FL
KRT73	6.95	5.03	Both Isoforms	LARP4	0.62	0.31	AR-FL
CHN2	2.47	5.04	Both Isoforms	ASAP3	0.78	0.31	AR-FL
MYH6	1.24	5.04	Both Isoforms	UBE2J1	0.99	0.31	AR-FL
ADAM7	3.87	5.06	Both Isoforms	ZNF44	0.65	0.31	AR-FL
PTGER4	3.90	5.07	Both Isoforms	PTPN1	0.82	0.31	AR-FL
ELL2	2.85	5.09	Both Isoforms	NSF	0.72	0.31	AR-FL
RCSD1	4.44	5.20	Both Isoforms	PROM2	0.62	0.31	AR-FL
KY	2.69	5.31	Both Isoforms	PCDHGB1	0.91	0.31	AR-FL
GP2	2.73	5.33	Both Isoforms	PGK1	0.83	0.31	AR-FL
SLC15A2	4.55	5.34	Both Isoforms	ACP2	0.85	0.32	AR-FL
TENM1	2.92	5.36	Both Isoforms	TM9SF2	0.74	0.32	AR-FL
TMCC3	5.06	5.45	Both Isoforms	HAT1	0.81	0.32	AR-FL
SPARC	2.32	5.48	Both Isoforms	ALDH16A1	0.63	0.32	AR-FL
IVL	2.11	5.56	Both Isoforms	SLC2A7	0.72	0.32	AR-FL
MYL7	2.99	5.69	Both Isoforms	PSMA3	0.64	0.32	AR-FL
TM4SF18	3.31	5.76	Both Isoforms	SLC30A7	0.90	0.32	AR-FL
F5	6.56	5.80	Both Isoforms	THUMPD1	0.86	0.32	AR-FL
PLA2G4E	5.81	5.94	Both Isoforms	CNOT3	0.64	0.32	AR-FL
EMP1	4.57	6.06	Both Isoforms	TIMM23	0.80	0.32	AR-FL
PGC	6.41	6.12	Both Isoforms	RSPH9	0.62	0.32	AR-FL
SLC6A3	2.12	6.16	Both Isoforms	LAMA5	0.64	0.32	AR-FL
TUBA3D	4.22	6.19	Both Isoforms	PSMA5	0.68	0.32	AR-FL

PNLIPRP3	3.29	6.20	Both Isoforms	PRKD2	1.11	0.32	AR-FL
SERPINF2	2.62	6.21	Both Isoforms	HCCS	0.60	0.32	AR-FL
STEAP4	10.11	6.36	Both Isoforms	TMEM11	0.76	0.32	AR-FL
NECAB1	0.70	6.44	Both Isoforms	PYCR1	0.82	0.32	AR-FL
MOGAT2	5.01	6.48	Both Isoforms	ODF2	0.59	0.32	AR-FL
TRIM49D1	5.86	6.58	Both Isoforms	C1GALT1C1	0.75	0.32	AR-FL
ZNF812	6.00	6.60	Both Isoforms	HN1	0.82	0.33	AR-FL
HOPX	0.92	6.66	Both Isoforms	PARK7	0.83	0.33	AR-FL
MYBPC1	5.03	6.81	Both Isoforms	ARL1	0.68	0.33	AR-FL
DOCK8	5.74	6.85	Both Isoforms	C21orf59	0.68	0.33	AR-FL
PAEP	3.31	6.95	Both Isoforms	CREB3	1.07	0.33	AR-FL
PDE6A	2.09	6.97	Both Isoforms	VAV2	0.80	0.33	AR-FL
SLC26A3	5.99	7.00	Both Isoforms	CLPB	0.61	0.33	AR-FL
SPOCK1	4.55	7.17	Both Isoforms	MTM1	0.66	0.33	AR-FL
NR5A2	1.28	7.27	Both Isoforms	APLP1	0.73	0.33	AR-FL
SLC2A3	10.76	7.46	Both Isoforms	SETX	0.60	0.33	AR-FL
NPR3	1.04	7.57	Both Isoforms	ROM1	0.90	0.33	AR-FL
TUBA3E	5.41	7.72	Both Isoforms	PPP2CB	0.69	0.33	AR-FL
NNT	4.40	7.72	Both Isoforms	DYNLL2	0.75	0.33	AR-FL
KRT6A	2.68	7.98	Both Isoforms	ACOX1	1.11	0.33	AR-FL
ANKRD1	2.21	8.06	Both Isoforms	ATP6V1E1	0.69	0.33	AR-FL
TGM2	5.15	8.13	Both Isoforms	CAMTA2	1.11	0.33	AR-FL
CCDC141	9.88	8.15	Both Isoforms	COMT	0.70	0.33	AR-FL
CHRNA2	6.63	8.36	Both Isoforms	GOLPH3	0.88	0.33	AR-FL
AKR1D1	6.08	8.36	Both Isoforms	TRAPPC3	0.69	0.33	AR-FL

GHRHR	5.90	8.40	Both Isoforms
SPINK13	1.90	8.40	Both Isoforms
PDK4	3.44	8.54	Both Isoforms
HPGD	6.77	8.54	Both Isoforms
IP6K3	3.75	8.56	Both Isoforms
AKAP12	6.04	8.71	Both Isoforms
OLAH	3.74	8.74	Both Isoforms
ACADL	6.25	8.89	Both Isoforms
FBLN5	1.51	9.70	Both Isoforms
DCN	3.29	9.77	Both Isoforms
CXCL13	7.06	9.88	Both Isoforms
SLC38A4	9.13	10.41	Both Isoforms
CTSE	4.78	10.46	Both Isoforms
ORM2	6.39	10.89	Both Isoforms
PNLIP	9.72	12.00	Both Isoforms
ORM1	6.65	12.38	Both Isoforms
PCDH7	2.43	12.43	Both Isoforms

H2AFY	0.65	0.33	AR-FL
NAPG	0.72	0.33	AR-FL
SMURF1	0.76	0.34	AR-FL
SORD	0.63	0.34	AR-FL
ZNF672	0.91	0.34	AR-FL
CLCC1	0.59	0.34	AR-FL
PVR	0.65	0.34	AR-FL
OPA3	0.80	0.34	AR-FL
SMIM4	0.72	0.34	AR-FL
VWA1	0.72	0.34	AR-FL
GRIN1	0.82	0.34	AR-FL
GSPT1	0.74	0.34	AR-FL
GSKIP	1.00	0.34	AR-FL
ARID1A	0.64	0.34	AR-FL
YWHAB	0.59	0.34	AR-FL
PSMC1	0.73	0.34	AR-FL
DDX3X	0.63	0.34	AR-FL
NDUFB1	0.66	0.34	AR-FL
ODC1	2.64	0.34	AR-FL
TMEM50A	1.08	0.34	AR-FL
SAP30	0.77	0.34	AR-FL
AUP1	0.78	0.34	AR-FL
ZBTB17	0.66	0.35	AR-FL
HIGD1A	0.77	0.35	AR-FL
PPCS	0.74	0.35	AR-FL
RREB1	0.59	0.35	AR-FL
IGF2BP3	0.72	0.35	AR-FL
SMDT1	0.77	0.35	AR-FL
FGFR3	1.64	0.35	AR-FL
ABCB6	0.60	0.35	AR-FL
KMT2D	0.80	0.35	AR-FL
GTF2A2	0.65	0.35	AR-FL

NOX5	0.90	0.35	AR-FL
MAP3K9	0.67	0.35	AR-FL
AP1G1	0.73	0.35	AR-FL
RPL36AL	0.63	0.35	AR-FL
ARPC2	0.63	0.35	AR-FL
XPNPEP1	0.65	0.35	AR-FL
PLIN3	0.91	0.35	AR-FL
LGALSL	1.05	0.35	AR-FL
TMEM87B	1.33	0.35	AR-FL
TMEM9B	0.59	0.35	AR-FL
ELP6	0.71	0.35	AR-FL
PPFIA3	1.13	0.35	AR-FL
CRB3	0.75	0.36	AR-FL
CTNS	0.66	0.36	AR-FL
C6orf1	0.73	0.36	AR-FL
ST3GAL4	1.26	0.36	AR-FL
HIST1H3E	0.64	0.36	AR-FL
KIAA1549	1.16	0.36	AR-FL
HMOX1	1.21	0.36	AR-FL
RAB8A	0.66	0.36	AR-FL
OTUB1	0.61	0.36	AR-FL
NDUFC1	0.75	0.36	AR-FL
TTC13	0.69	0.36	AR-FL
FAM98A	0.69	0.36	AR-FL
ZNF628	0.63	0.36	AR-FL
ZNF440	0.63	0.36	AR-FL
MICAL1	2.39	0.36	AR-FL
COG3	0.76	0.36	AR-FL
KIF5C	0.59	0.36	AR-FL
BTBD3	0.65	0.36	AR-FL
SLC19A1	0.65	0.36	AR-FL
ARCN1	0.79	0.36	AR-FL
CTDP1	0.65	0.36	AR-FL
PLPP6	1.00	0.36	AR-FL
CD9	1.35	0.36	AR-FL
RAB3D	1.31	0.36	AR-FL
BNIP1	0.60	0.36	AR-FL
COMMD7	0.61	0.36	AR-FL
DISP1	0.62	0.36	AR-FL
SLC25A45	0.96	0.37	AR-FL
ERCC4	0.62	0.37	AR-FL
MYO1C	1.01	0.37	AR-FL
C19orf24	0.70	0.37	AR-FL
MCOLN1	0.86	0.37	AR-FL
C3orf70	0.75	0.37	AR-FL
MTCH2	0.80	0.37	AR-FL

MRPL20	0.58	0.37	AR-FL
EPN3	0.82	0.37	AR-FL
SLC45A1	0.76	0.37	AR-FL
NARF	1.08	0.37	AR-FL
NDUFB3	0.65	0.37	AR-FL
TRADD	0.75	0.37	AR-FL
FUT11	0.95	0.37	AR-FL
PDXDC1	0.72	0.37	AR-FL
SMAP1	0.85	0.37	AR-FL
GOLGB1	0.79	0.37	AR-FL
SIGIRR	0.80	0.37	AR-FL
ABHD13	0.62	0.37	AR-FL
PMM2	0.67	0.37	AR-FL
MKL1	0.63	0.37	AR-FL
NDUFA8	0.64	0.37	AR-FL
NAA16	0.58	0.37	AR-FL
TBC1D7	0.74	0.37	AR-FL
GK5	0.67	0.37	AR-FL
TFPT	1.28	0.38	AR-FL
UQCRFS1	0.72	0.38	AR-FL
MLPH	0.65	0.38	AR-FL
CHCHD3	0.63	0.38	AR-FL
SLC52A3	0.65	0.38	AR-FL
FAM177A1	0.84	0.38	AR-FL
ECM1	1.15	0.38	AR-FL
LAGE3	0.78	0.38	AR-FL
OGDH	0.94	0.38	AR-FL
BAX	0.73	0.38	AR-FL
SPDEF	2.16	0.38	AR-FL
RPS19BP1	0.76	0.38	AR-FL
ATG101	0.94	0.38	AR-FL
TRPM4	0.99	0.38	AR-FL
RUSC1	1.49	0.38	AR-FL
TBXA2R	0.94	0.38	AR-FL
ALG11	0.66	0.38	AR-FL
ARHGAP26	2.12	0.38	AR-FL
PKM	0.77	0.38	AR-FL
POLD4	0.59	0.38	AR-FL
HIST1H2AE	0.79	0.38	AR-FL
NAPRT	0.90	0.38	AR-FL
MYH9	0.82	0.39	AR-FL
LRG1	1.46	0.39	AR-FL
CENPBD1	0.68	0.39	AR-FL
BEST3	0.77	0.39	AR-FL
VEGFA	0.70	0.39	AR-FL
SCAMP2	0.80	0.39	AR-FL

	ACTR2	0.73	0.39	AR-FL
	ANO10	0.64	0.39	AR-FL
	SLC30A5	0.60	0.39	AR-FL
	IGHMBP2	0.81	0.39	AR-FL
	SEC63	0.69	0.39	AR-FL
	GJB1	1.07	0.39	AR-FL
	STX3	0.82	0.39	AR-FL
	COPB2	0.98	0.39	AR-FL
	MESDC1	1.26	0.39	AR-FL
	DAPK3	0.73	0.39	AR-FL
	SSNA1	0.75	0.39	AR-FL
	TOR2A	0.86	0.39	AR-FL
	ZNF678	0.70	0.40	AR-FL
	CHUK	0.77	0.40	AR-FL
	FXR2	0.86	0.40	AR-FL
	GOLT1B	0.95	0.40	AR-FL
	TMEM222	0.60	0.40	AR-FL
	AES	0.68	0.40	AR-FL
	DTX1	1.86	0.40	AR-FL
	CARTPT	1.21	0.40	AR-FL
	EIF4G2	1.00	0.40	AR-FL
	TMC6	1.03	0.40	AR-FL
	PPP4C	0.63	0.40	AR-FL
	RAC3	0.60	0.40	AR-FL
	ARPC4	0.94	0.40	AR-FL
	USP38	0.75	0.40	AR-FL
	IDH2	0.84	0.40	AR-FL
	BCL2L2	0.89	0.40	AR-FL
	GNA11	0.60	0.40	AR-FL
	HAGH	0.70	0.40	AR-FL
	BAD	0.59	0.40	AR-FL
	DPM3	1.12	0.40	AR-FL
	PITPNA	1.04	0.40	AR-FL
	LAMTOR4	0.66	0.40	AR-FL
	FTSJ1	0.58	0.40	AR-FL
	AP3D1	0.63	0.41	AR-FL
	DUSP1	1.46	0.41	AR-FL
	SPATS2	0.68	0.41	AR-FL
	KCTD9	0.59	0.41	AR-FL
	RP11-111H13.1	1.17	0.41	AR-FL
	DOHH	0.61	0.41	AR-FL
	NIPA2	0.86	0.41	AR-FL
	SBNO2	0.61	0.41	AR-FL
	G3BP2	1.39	0.41	AR-FL
	ZDHHC5	0.65	0.41	AR-FL
	BLOC1S1	0.63	0.41	AR-FL

NEK3	1.35	0.41	AR-FL
FKBP14	0.61	0.41	AR-FL
TGFB2	1.41	0.41	AR-FL
ELP5	0.68	0.41	AR-FL
TNS3	0.70	0.41	AR-FL
NAPA	0.60	0.41	AR-FL
LYPLA2	1.03	0.41	AR-FL
ENO1	0.89	0.41	AR-FL
SNRNP40	0.63	0.41	AR-FL
CDC42SE1	1.01	0.41	AR-FL
PPM1H	1.48	0.41	AR-FL
SCO2	0.64	0.41	AR-FL
RANGRF	1.46	0.41	AR-FL
PPP6R1	0.66	0.41	AR-FL
COPRS	0.92	0.41	AR-FL
POR	0.66	0.41	AR-FL
KLK15	2.04	0.41	AR-FL
IRAK1	0.59	0.41	AR-FL
BCAP31	0.79	0.41	AR-FL
DYNLRB1	0.66	0.41	AR-FL
COX6B1	0.80	0.41	AR-FL
UNC13A	0.90	0.41	AR-FL
PLEKHF1	0.87	0.42	AR-FL
HNRNPUL2-BSCL2	0.75	0.42	AR-FL
ZNF410	1.12	0.42	AR-FL
MPZL1	1.10	0.42	AR-FL
MAPK7	0.84	0.42	AR-FL
STT3B	0.62	0.42	AR-FL
SNRPG	0.65	0.42	AR-FL
ZNHIT2	0.65	0.42	AR-FL
ARPC3	1.10	0.42	AR-FL
SLC2A1	0.82	0.42	AR-FL
AKT1	0.90	0.42	AR-FL
TIMM10B	0.72	0.42	AR-FL
RNGTT	0.65	0.42	AR-FL
RPS6KA1	1.09	0.42	AR-FL
CYP2U1	1.17	0.42	AR-FL
KLK2	1.03	0.42	AR-FL
SLC35A4	0.69	0.42	AR-FL
TRAPPC9	0.62	0.42	AR-FL
CLINT1	0.66	0.42	AR-FL
PDIA5	1.17	0.42	AR-FL
ADRBK1	1.40	0.42	AR-FL
NR2F6	0.73	0.43	AR-FL
SAMHD1	0.99	0.43	AR-FL

ERG	1.77	0.43	AR-FL
CANX	0.67	0.43	AR-FL
HCFC1	0.77	0.43	AR-FL
EIF4G1	0.72	0.43	AR-FL
TMPRSS2	3.28	0.43	AR-FL
PIAS3	1.00	0.43	AR-FL
IL17RC	0.59	0.43	AR-FL
RHOG	0.60	0.43	AR-FL
ROGDI	1.83	0.43	AR-FL
JKAMP	1.03	0.43	AR-FL
GORASP2	0.86	0.43	AR-FL
BCL7B	0.74	0.43	AR-FL
FRK	1.32	0.43	AR-FL
GNB2	0.98	0.43	AR-FL
NDUFA1	0.69	0.43	AR-FL
ALG5	0.69	0.43	AR-FL
S100A11	0.84	0.43	AR-FL
MIS12	0.68	0.43	AR-FL
SERF2	0.74	0.43	AR-FL
TSPAN17	0.69	0.43	AR-FL
POLR3E	1.07	0.43	AR-FL
SMG5	0.83	0.43	AR-FL
BCL2L1	1.05	0.44	AR-FL
NFATC2IP	0.81	0.44	AR-FL
MLLT11	0.87	0.44	AR-FL
WDR77	0.71	0.44	AR-FL
SLC5A6	0.80	0.44	AR-FL
EXOSC4	0.72	0.44	AR-FL
CISD2	0.62	0.44	AR-FL
PYCR2	0.63	0.44	AR-FL
STEAP4	4.52	0.44	AR-FL
RAB35	0.60	0.44	AR-FL
ZSWIM8	0.61	0.44	AR-FL
TLDC2	0.99	0.44	AR-FL
FAM222A	0.96	0.44	AR-FL
GDPD5	0.79	0.44	AR-FL
SELM	1.20	0.44	AR-FL
TRAM1	0.87	0.44	AR-FL
TMCO1	0.67	0.44	AR-FL
IL17RA	0.69	0.44	AR-FL
EIF4G3	0.75	0.44	AR-FL
WWC2	0.88	0.44	AR-FL
PROB1	0.72	0.44	AR-FL
SLC22A5	1.40	0.45	AR-FL
HIVEP3	0.77	0.45	AR-FL
UCHL3	0.73	0.45	AR-FL

SGSH	0.99	0.45	AR-FL
TMEM199	0.87	0.45	AR-FL
BOLA3	0.65	0.45	AR-FL
SPOCK3	0.85	0.45	AR-FL
RAVER1	0.64	0.45	AR-FL
RABGEF1	0.61	0.45	AR-FL
PPP1R15B	0.70	0.45	AR-FL
C11orf84	0.61	0.45	AR-FL
ATXN7L3	0.72	0.45	AR-FL
TMED5	0.91	0.45	AR-FL
SCAMP4	0.62	0.45	AR-FL
GRID2IP	0.64	0.45	AR-FL
SETD3	0.81	0.45	AR-FL
AIFM1	0.77	0.45	AR-FL
PSME4	1.05	0.45	AR-FL
RNF103	0.89	0.45	AR-FL
ATP5G3	0.68	0.45	AR-FL
HDLBP	0.67	0.45	AR-FL
COX18	0.79	0.45	AR-FL
LDHA	1.15	0.45	AR-FL
FAM126B	0.71	0.46	AR-FL
SLC9A3R2	1.76	0.46	AR-FL
FZD4	0.99	0.46	AR-FL
CDC42EP4	1.18	0.46	AR-FL
MFSD12	0.71	0.46	AR-FL
SNAPC5	0.63	0.46	AR-FL
SH3TC1	1.23	0.46	AR-FL
PUF60	0.60	0.46	AR-FL
MON1B	0.80	0.46	AR-FL
PACSIN1	1.10	0.46	AR-FL
ALAS1	1.07	0.46	AR-FL
GADD45B	0.81	0.46	AR-FL
DBNL	0.63	0.46	AR-FL
OLIG1	0.71	0.46	AR-FL
RPS6KA4	1.22	0.46	AR-FL
IRX4	1.19	0.46	AR-FL
GOLGA2	1.00	0.46	AR-FL
SERP1	0.85	0.46	AR-FL
ANKRD52	0.82	0.46	AR-FL
TMEM258	0.59	0.46	AR-FL
BCL9L	1.05	0.46	AR-FL
DPM2	0.74	0.46	AR-FL
CLN8	0.68	0.46	AR-FL
SMIM10L1	0.66	0.46	AR-FL
CALM1	0.73	0.46	AR-FL
MARVELD2	0.68	0.46	AR-FL

SNRPB	0.65	0.46	AR-FL
TECPR1	1.33	0.46	AR-FL
PSME2	0.90	0.46	AR-FL
HIST1H2BH	0.76	0.46	AR-FL
KPNA1	0.95	0.46	AR-FL
ZWILCH	0.58	0.46	AR-FL
MRPL52	0.66	0.46	AR-FL
DPP3	1.28	0.47	AR-FL
TNKS1BP1	0.61	0.47	AR-FL
TSSC4	0.69	0.47	AR-FL
MAP3K11	0.98	0.47	AR-FL
GPR137	1.12	0.47	AR-FL
FAM91A1	0.76	0.47	AR-FL
ATP6V1D	0.83	0.47	AR-FL
TUBB2A	1.71	0.47	AR-FL
DNAJA1	0.63	0.47	AR-FL
NDUFA6	0.82	0.47	AR-FL
PSME3	0.71	0.47	AR-FL
FBXW5	0.86	0.47	AR-FL
POMP	0.64	0.47	AR-FL
TMEM208	0.59	0.47	AR-FL
NDUFB10	1.06	0.47	AR-FL
ZNF511	0.89	0.47	AR-FL
MEMO1	0.68	0.47	AR-FL
CLTC	0.72	0.47	AR-FL
FAM57A	0.89	0.47	AR-FL
STK40	1.11	0.47	AR-FL
CCDC124	0.72	0.47	AR-FL
LSR	0.90	0.47	AR-FL
SEC22B	0.91	0.47	AR-FL
LSM1	0.71	0.47	AR-FL
C2CD3	0.62	0.47	AR-FL
PPL	0.93	0.48	AR-FL
ASPSCR1	0.62	0.48	AR-FL
ZNF653	0.75	0.48	AR-FL
TIMM17A	0.72	0.48	AR-FL
TVP23C	0.96	0.48	AR-FL
TOMM40L	0.63	0.48	AR-FL
PSMC2	0.92	0.48	AR-FL
FASTKD5	0.69	0.48	AR-FL
ZNF35	1.07	0.48	AR-FL
TRIM5	1.12	0.48	AR-FL
NDUFS5	0.62	0.48	AR-FL
WDR24	0.78	0.48	AR-FL
ATP6V0C	1.01	0.48	AR-FL
JMJD8	0.78	0.48	AR-FL

COMMD5	1.09	0.48	AR-FL
RAB3B	1.16	0.48	AR-FL
NABP2	0.73	0.48	AR-FL
VPS13D	0.88	0.48	AR-FL
GBA	1.04	0.48	AR-FL
RBMS1	1.71	0.49	AR-FL
NUDC	0.59	0.49	AR-FL
PPP1CA	0.80	0.49	AR-FL
TMEM63B	1.12	0.49	AR-FL
ENTPD4	0.76	0.49	AR-FL
ATP6V0A2	1.37	0.49	AR-FL
TPRKB	0.73	0.49	AR-FL
TMEM33	0.90	0.49	AR-FL
YOD1	0.78	0.49	AR-FL
GPRIN1	0.58	0.49	AR-FL
SIL1	0.75	0.49	AR-FL
ARF1	0.92	0.49	AR-FL
IFT140	0.80	0.50	AR-FL
UBLCP1	0.73	0.50	AR-FL
GRINA	0.73	0.50	AR-FL
NXPH3	0.80	0.50	AR-FL
RNF145	0.58	0.50	AR-FL
LRWD1	0.72	0.50	AR-FL
TMEM191B	0.86	0.50	AR-FL
ZNF611	0.92	0.50	AR-FL
SDF4	0.77	0.50	AR-FL
PDE8B	0.77	0.50	AR-FL
GET4	0.72	0.50	AR-FL
SCYL1	0.97	0.50	AR-FL
SMIM12	0.60	0.50	AR-FL
ZNF66	0.98	0.50	AR-FL
DNAJC25	0.67	0.50	AR-FL
EMC7	0.67	0.50	AR-FL
AAGAB	0.76	0.50	AR-FL
TMEM167A	0.83	0.50	AR-FL
SLC25A48	0.66	0.50	AR-FL
HSPA13	0.68	0.50	AR-FL
PRKAR2A	0.84	0.51	AR-FL
IL20RA	2.02	0.51	AR-FL
VPS53	1.02	0.51	AR-FL
SWSAP1	0.90	0.51	AR-FL
TMEM251	1.18	0.51	AR-FL
ATP5E	0.66	0.51	AR-FL
CDC42EP2	2.55	0.51	AR-FL
MRPL34	0.82	0.51	AR-FL
ANKRD13D	0.84	0.51	AR-FL

SDHAF2	0.76	0.51	AR-FL
COASY	0.65	0.51	AR-FL
POLR2K	0.77	0.51	AR-FL
PGAM5	0.69	0.51	AR-FL
TAF10	0.70	0.51	AR-FL
ASNA1	0.64	0.51	AR-FL
CLPTM1L	0.79	0.52	AR-FL
GNG5	0.73	0.52	AR-FL
ACOT7	0.65	0.52	AR-FL
PTGES2	0.58	0.52	AR-FL
FGD4	0.73	0.52	AR-FL
SH3BP1	0.62	0.52	AR-FL
ACTR1A	1.13	0.52	AR-FL
RER1	0.66	0.52	AR-FL
CAPZB	1.08	0.52	AR-FL
ANGPT2	2.54	0.52	AR-FL
HEXIM1	0.94	0.52	AR-FL
EMC9	0.91	0.52	AR-FL
GLB1L2	0.80	0.52	AR-FL
VPS18	0.81	0.52	AR-FL
CCPG1	0.65	0.52	AR-FL
FAM219A	1.09	0.52	AR-FL
SOCS4	0.83	0.52	AR-FL
C16orf71	0.79	0.52	AR-FL
RHOT2	0.65	0.52	AR-FL
BIRC5	0.65	0.52	AR-FL
ATF5	1.00	0.52	AR-FL
TPM3	0.85	0.52	AR-FL
C10orf35	0.61	0.52	AR-FL
FHOD1	1.02	0.52	AR-FL
HCFC2	0.70	0.53	AR-FL
PICALM	0.68	0.53	AR-FL
EFR3B	1.20	0.53	AR-FL
CAMK1	1.09	0.53	AR-FL
OST4	0.66	0.53	AR-FL
GOLGA4	0.83	0.53	AR-FL
FRMD8	0.72	0.53	AR-FL
SLC4A2	0.71	0.53	AR-FL
MFAP3L	0.63	0.53	AR-FL
SOGA1	0.69	0.53	AR-FL
TSPAN14	0.83	0.53	AR-FL
CYB5B	0.67	0.53	AR-FL
UBL5	0.60	0.53	AR-FL
C19orf47	0.66	0.53	AR-FL
IGF1R	0.69	0.53	AR-FL
CTSD	0.91	0.53	AR-FL

PI4K2A	0.69	0.53	AR-FL
SLC35B2	1.09	0.53	AR-FL
GPT2	0.82	0.53	AR-FL
DRAP1	0.70	0.53	AR-FL
TECR	0.63	0.53	AR-FL
PSMD8	1.04	0.53	AR-FL
SHISA4	0.66	0.53	AR-FL
COX8A	0.83	0.54	AR-FL
SYP	0.82	0.54	AR-FL
ZHX1	0.61	0.54	AR-FL
TRIM11	1.18	0.54	AR-FL
TXNDC17	1.14	0.54	AR-FL
TMEM205	1.23	0.54	AR-FL
FOS	0.94	0.54	AR-FL
CPSF2	0.88	0.54	AR-FL
USMG5	0.82	0.54	AR-FL
ZSCAN5A	0.69	0.54	AR-FL
HIST1H1C	0.87	0.54	AR-FL
DMWD	0.83	0.54	AR-FL
SLC9A1	0.81	0.54	AR-FL
CHMP2A	0.93	0.54	AR-FL
MAPRE1	0.78	0.54	AR-FL
MAPK11	0.82	0.54	AR-FL
MRPS11	0.70	0.54	AR-FL
CD63	1.17	0.54	AR-FL
WHAMM	0.63	0.54	AR-FL
C17orf104	1.01	0.54	AR-FL
ZNF513	0.77	0.54	AR-FL
GPAT4	0.72	0.54	AR-FL
PCCB	0.73	0.55	AR-FL
ATP5J2	0.87	0.55	AR-FL
ABCA7	0.74	0.55	AR-FL
KIAA1841	0.64	0.55	AR-FL
RAB20	0.74	0.55	AR-FL
ZNF697	1.79	0.55	AR-FL
TAF6L	0.63	0.55	AR-FL
NTMT1	0.98	0.55	AR-FL
DOT1L	1.05	0.55	AR-FL
RAB1B	1.10	0.55	AR-FL
KCNG3	1.37	0.55	AR-FL
EPDR1	0.74	0.55	AR-FL
PIP5K1A	0.65	0.55	AR-FL
SHFM1	0.75	0.55	AR-FL
PTGS1	2.96	0.55	AR-FL
GBA2	0.88	0.55	AR-FL
ACSS2	0.60	0.55	AR-FL

ECI2	0.77	0.55	AR-FL
ALDH1A2	0.90	0.55	AR-FL
ZCCHC24	1.16	0.55	AR-FL
SNX11	0.89	0.55	AR-FL
SC5D	0.84	0.55	AR-FL
RPS6KB2	0.94	0.55	AR-FL
TMEM151A	1.22	0.55	AR-FL
TESK1	1.33	0.55	AR-FL
NCLN	0.73	0.55	AR-FL
SLC25A53	0.72	0.55	AR-FL
PPP6R2	1.20	0.55	AR-FL
C9orf69	0.92	0.55	AR-FL
ELMO3	0.68	0.55	AR-FL
HYAL2	0.95	0.56	AR-FL
HK1	1.18	0.56	AR-FL
CASD1	0.92	0.56	AR-FL
PCBP4	0.63	0.56	AR-FL
MYEOV2	0.65	0.56	AR-FL
SGOL1	0.66	0.56	AR-FL
GHRHR	1.13	0.56	AR-FL
TEX2	0.82	0.56	AR-FL
MTA2	1.01	0.56	AR-FL
SLC25A1	0.73	0.56	AR-FL
CANT1	1.55	0.56	AR-FL
CYP4F12	1.82	0.56	AR-FL
LSM4	0.65	0.56	AR-FL
ATRNL1	0.80	0.56	AR-FL
TALDO1	0.72	0.56	AR-FL
SLC41A1	0.60	0.56	AR-FL
IFT22	0.61	0.57	AR-FL
IDH1	1.52	0.57	AR-FL
LRRC16A	0.78	0.57	AR-FL
GTF3C6	0.97	0.57	AR-FL
ISOC2	0.99	0.57	AR-FL
ASL	0.67	0.57	AR-FL
RNF25	0.68	0.57	AR-FL
EMC6	1.29	0.57	AR-FL
CRADD	0.65	0.57	AR-FL
PSMB2	0.82	0.57	AR-FL
LSM10	0.89	0.57	AR-FL
PDXK	0.88	0.57	AR-FL
MESDC2	0.87	0.57	AR-FL
ZNF668	0.97	0.57	AR-FL
TSC22D3	0.64	0.57	AR-FL
CABIN1	0.67	0.57	AR-FL
CASKIN2	0.73	0.57	AR-FL

RABAC1	1.14	0.57	AR-FL
PRPF18	0.60	0.57	AR-FL
UQCC2	0.61	0.57	AR-FL
SLC30A2	1.90	0.57	AR-FL
KIAA0513	0.85	0.57	AR-FL
XKR8	0.68	0.57	AR-FL
OSCP1	0.63	0.57	AR-FL
AP2S1	1.10	0.57	AR-FL
CTSV	0.92	0.58	AR-FL
ZNF341	0.83	0.58	AR-FL
PSMB6	1.08	0.58	AR-FL
CALML4	0.77	0.58	AR-FL
CKAP4	0.74	0.58	AR-FL
PHLDA2	1.27	0.58	AR-FL
ATP6V0E2	1.36	0.58	AR-FL
RNASEH1	0.63	0.58	AR-FL
ATPIF1	0.70	0.58	AR-FL
STIP1	0.91	0.58	Both Isoforms
CISD3	0.65	0.58	Both Isoforms
AGR3	2.93	0.58	Both Isoforms
JUNB	0.69	0.58	Both Isoforms
ERLEC1	0.62	0.58	Both Isoforms
CHRNA10	0.70	0.58	Both Isoforms
RABL6	0.65	0.58	Both Isoforms
TMED2	0.94	0.58	Both Isoforms
ACTB	0.69	0.58	Both Isoforms
FBXO16	0.70	0.58	Both Isoforms
KDELR2	1.38	0.58	Both Isoforms
COX7B	0.88	0.58	Both Isoforms
ZC3HAV1	0.68	0.58	Both Isoforms
C1orf210	0.70	0.58	Both Isoforms
STX5	0.93	0.58	Both Isoforms
PSMD14	1.04	0.59	Both Isoforms
DPAGT1	1.19	0.59	Both Isoforms
KIF1C	1.30	0.59	Both Isoforms
KLF4	1.78	0.59	Both Isoforms
ZNF813	0.85	0.59	Both Isoforms
KLHL18	0.76	0.59	Both Isoforms
PSMC5	0.92	0.59	Both Isoforms
PXN	0.88	0.59	Both Isoforms
RAP1A	0.89	0.59	Both Isoforms
TCEB2	0.76	0.59	Both Isoforms
HMGCR	1.07	0.59	Both Isoforms
IKBKG	0.90	0.59	Both Isoforms
DIAPH1	0.63	0.59	Both Isoforms
AC004381.6	0.64	0.59	Both Isoforms

PQLC2	0.91	0.59	Both Isoforms
FLAD1	0.90	0.59	Both Isoforms
ALG8	1.16	0.59	Both Isoforms
NANS	1.22	0.59	Both Isoforms
MICALL1	0.89	0.59	Both Isoforms
OSBPL7	0.96	0.59	Both Isoforms
MRPS26	0.66	0.59	Both Isoforms
PFN1	0.95	0.59	Both Isoforms
RALY	0.80	0.59	Both Isoforms
LETM1	0.83	0.59	Both Isoforms
RNF181	0.66	0.59	Both Isoforms
KCNS3	0.65	0.59	Both Isoforms
UBA1	0.89	0.59	Both Isoforms
ABHD5	0.88	0.59	Both Isoforms
ANAPC11	0.79	0.59	Both Isoforms
PSMB3	0.94	0.59	Both Isoforms
MYL12A	1.30	0.59	Both Isoforms
IFT27	0.67	0.59	Both Isoforms
NUP62CL	0.83	0.59	Both Isoforms
WDR1	0.74	0.60	Both Isoforms
SWT1	1.22	0.60	Both Isoforms
MAP4K2	1.01	0.60	Both Isoforms
C17orf89	0.73	0.60	Both Isoforms
GALE	0.69	0.60	Both Isoforms
ZNF467	2.12	0.60	Both Isoforms
ARHGDIA	0.88	0.60	Both Isoforms
IDH3G	1.06	0.60	Both Isoforms
SAPCD2	1.42	0.60	Both Isoforms
PLEKHB2	0.99	0.60	Both Isoforms
CHSY1	1.34	0.60	Both Isoforms
TUBA1C	1.21	0.60	Both Isoforms
DNM2	0.68	0.60	Both Isoforms
BAHD1	0.59	0.60	Both Isoforms
WNT7B	0.84	0.60	Both Isoforms
ALG14	0.87	0.60	Both Isoforms
CAMSAP3	0.89	0.60	Both Isoforms
NPLOC4	0.93	0.60	Both Isoforms
SLC25A10	0.73	0.60	Both Isoforms
FPGS	0.62	0.60	Both Isoforms
KIF4B	0.61	0.61	Both Isoforms
ABAT	0.92	0.61	Both Isoforms
ZNF724P	0.64	0.61	Both Isoforms
MTCH1	0.88	0.61	Both Isoforms
ZDHHC12	0.59	0.61	Both Isoforms
FUT1	0.70	0.61	Both Isoforms
FAAP24	0.79	0.61	Both Isoforms

PPP2R3C	0.86	0.61	Both Isoforms
KIF22	1.51	0.61	Both Isoforms
PCDHB5	1.00	0.61	Both Isoforms
ATP5G1	0.73	0.61	Both Isoforms
SLC25A13	1.02	0.61	Both Isoforms
UBE2M	0.92	0.61	Both Isoforms
RAB1A	1.06	0.61	Both Isoforms
ACADVL	1.07	0.61	Both Isoforms
PSMC4	1.26	0.61	Both Isoforms
ADCY6	1.21	0.61	Both Isoforms
MPC2	1.73	0.61	Both Isoforms
TRIM3	1.07	0.61	Both Isoforms
COX14	0.68	0.61	Both Isoforms
HMG20B	1.41	0.61	Both Isoforms
EDEM1	1.09	0.62	Both Isoforms
PSMD11	0.95	0.62	Both Isoforms
UGT2B28	2.97	0.62	Both Isoforms
ALDOA	1.18	0.62	Both Isoforms
LMNA	1.05	0.62	Both Isoforms
IWS1	0.81	0.62	Both Isoforms
CLPTM1	1.01	0.62	Both Isoforms
C17orf107	0.87	0.62	Both Isoforms
IMPDH1	1.22	0.62	Both Isoforms
TSEN15	0.66	0.62	Both Isoforms
EMC3	0.95	0.62	Both Isoforms
CIB1	1.12	0.62	Both Isoforms
IPPK	1.66	0.62	Both Isoforms
MLX	0.88	0.62	Both Isoforms
ALG12	0.90	0.62	Both Isoforms
TRAPPC10	0.75	0.62	Both Isoforms
TFRC	1.09	0.63	Both Isoforms
APOO	0.59	0.63	Both Isoforms
DOLPP1	1.03	0.63	Both Isoforms
ZFHX3	0.92	0.63	Both Isoforms
SEC61A2	0.74	0.63	Both Isoforms
TMEM141	1.22	0.63	Both Isoforms
PSMD2	1.02	0.63	Both Isoforms
EIF2B2	0.58	0.63	Both Isoforms
PNP	0.59	0.63	Both Isoforms
ZNF726	0.60	0.63	Both Isoforms
RBX1	1.09	0.63	Both Isoforms
CAP2	1.75	0.63	Both Isoforms
DERL2	0.82	0.64	Both Isoforms
FZD8	1.86	0.64	Both Isoforms
SLC50A1	1.37	0.64	Both Isoforms
HNRNPAB	0.90	0.64	Both Isoforms

RAB4B	1.07	0.64	Both Isoforms
ZGPAT	0.64	0.64	Both Isoforms
C14orf1	0.86	0.64	Both Isoforms
SAP130	0.82	0.64	Both Isoforms
HID1	1.66	0.64	Both Isoforms
GUK1	0.78	0.64	Both Isoforms
PIGK	0.93	0.64	Both Isoforms
CPTP	0.93	0.64	Both Isoforms
CREB3L4	1.67	0.64	Both Isoforms
SLC25A42	1.32	0.64	Both Isoforms
LDLRAP1	1.04	0.65	Both Isoforms
ZNF563	1.89	0.65	Both Isoforms
ALDH4A1	1.09	0.65	Both Isoforms
NDUFAF1	0.72	0.65	Both Isoforms
MRPL36	1.01	0.65	Both Isoforms
PCDHGC3	1.35	0.65	Both Isoforms
MYH13	2.25	0.65	Both Isoforms
PSMG3	0.60	0.65	Both Isoforms
C16orf54	1.37	0.65	Both Isoforms
SSR2	0.95	0.65	Both Isoforms
FNDC3B	1.09	0.65	Both Isoforms
RXFP4	1.26	0.65	Both Isoforms
UQCR10	0.81	0.65	Both Isoforms
HIST1H2BJ	0.81	0.65	Both Isoforms
PDE12	0.95	0.65	Both Isoforms
GPATCH4	0.70	0.65	Both Isoforms
TMED7	1.14	0.65	Both Isoforms
SEMA3G	2.44	0.66	Both Isoforms
SLC27A4	1.35	0.66	Both Isoforms
CARD10	1.15	0.66	Both Isoforms
PIK3C2A	0.73	0.66	Both Isoforms
NACC2	1.26	0.66	Both Isoforms
LSS	0.74	0.66	Both Isoforms
TTC38	0.63	0.66	Both Isoforms
HSD17B10	0.73	0.66	Both Isoforms
SLC31A1	0.93	0.66	Both Isoforms
CHKA	0.81	0.66	Both Isoforms
SNX19	0.92	0.66	Both Isoforms
SLC33A1	0.75	0.66	Both Isoforms
SRM	0.67	0.66	Both Isoforms
UBE2S	1.14	0.66	Both Isoforms
TMEM150C	1.19	0.66	Both Isoforms
VPS26B	2.53	0.66	Both Isoforms
PYM1	0.99	0.66	Both Isoforms
SDSL	1.09	0.66	Both Isoforms
RAB5C	0.99	0.66	Both Isoforms

LGMN	1.50	0.66	Both Isoforms
IDH3A	1.18	0.66	Both Isoforms
GOLGA3	1.14	0.66	Both Isoforms
ENG	1.34	0.66	Both Isoforms
GDI1	0.80	0.66	Both Isoforms
TMCC3	0.63	0.66	Both Isoforms
SHKBP1	1.15	0.66	Both Isoforms
CABLES2	0.60	0.66	Both Isoforms
BRMS1	0.97	0.66	Both Isoforms
TOR3A	0.68	0.66	Both Isoforms
TMA7	0.64	0.66	Both Isoforms
ZNF808	0.60	0.67	Both Isoforms
IQCE	0.79	0.67	Both Isoforms
DLX2	1.48	0.67	Both Isoforms
SCAMP3	1.02	0.67	Both Isoforms
IFI27L1	0.78	0.67	Both Isoforms
MPZL2	0.88	0.67	Both Isoforms
MRPL41	0.92	0.67	Both Isoforms
CNN3	0.63	0.67	Both Isoforms
PRKACA	0.73	0.67	Both Isoforms
HERPUD1	0.95	0.67	Both Isoforms
PSMC3	0.86	0.67	Both Isoforms
TBRG1	0.59	0.67	Both Isoforms
GREB1	1.03	0.67	Both Isoforms
ZNF101	0.81	0.67	Both Isoforms
SLC25A11	0.92	0.67	Both Isoforms
SF3B5	0.90	0.67	Both Isoforms
MEA1	0.76	0.68	Both Isoforms
SEC24A	1.41	0.68	Both Isoforms
SLC35A2	0.79	0.68	Both Isoforms
MFSD3	1.21	0.68	Both Isoforms
RGMB	0.69	0.68	Both Isoforms
AP5B1	0.60	0.68	Both Isoforms
WDYHV1	0.61	0.68	Both Isoforms
PPFIA4	1.20	0.68	Both Isoforms
DDA1	0.77	0.68	Both Isoforms
ACAD8	1.38	0.68	Both Isoforms
DPP9	0.67	0.68	Both Isoforms
SQLE	0.95	0.68	Both Isoforms
DEF8	0.61	0.69	Both Isoforms
TRPM2	1.25	0.69	Both Isoforms
PCSK7	1.15	0.69	Both Isoforms
FAM129B	0.70	0.69	Both Isoforms
SEC24C	1.19	0.69	Both Isoforms
PTS	0.94	0.69	Both Isoforms
DNAL1	0.65	0.69	Both Isoforms

SCD	1.82	0.69	Both Isoforms
YKT6	1.03	0.69	Both Isoforms
TMEM125	1.60	0.69	Both Isoforms
RBM14	0.64	0.69	Both Isoforms
ATP10A	1.81	0.69	Both Isoforms
SSR4	0.90	0.69	Both Isoforms
SLC25A35	2.56	0.69	Both Isoforms
DDX5	0.89	0.69	Both Isoforms
PXMP4	0.78	0.69	Both Isoforms
MGST2	1.11	0.69	Both Isoforms
SPHK2	0.81	0.69	Both Isoforms
CALU	1.36	0.70	Both Isoforms
PRDX4	0.81	0.70	Both Isoforms
SERTAD3	1.22	0.70	Both Isoforms
RYR3	0.77	0.70	Both Isoforms
KLC2	1.34	0.70	Both Isoforms
CTPS1	0.69	0.70	Both Isoforms
OSTM1	1.48	0.70	Both Isoforms
CCDC173	3.09	0.70	Both Isoforms
TSPAN1	1.29	0.70	Both Isoforms
APLN	1.09	0.70	Both Isoforms
CCDC64	1.20	0.70	Both Isoforms
ICMT	0.97	0.70	Both Isoforms
VCP	1.20	0.70	Both Isoforms
MAGED1	1.48	0.70	Both Isoforms
CYB561	1.05	0.70	Both Isoforms
FAM213A	0.62	0.70	Both Isoforms
CACNG4	1.91	0.70	Both Isoforms
CLPP	0.66	0.70	Both Isoforms
GNPNAT1	0.79	0.70	Both Isoforms
PSMA7	1.09	0.70	Both Isoforms
SLC9A3R1	1.54	0.70	Both Isoforms
TIMM23B	0.61	0.70	Both Isoforms
TOB1	0.81	0.70	Both Isoforms
CYB561D2	1.24	0.70	Both Isoforms
GLA	0.71	0.70	Both Isoforms
SLX4	0.71	0.71	Both Isoforms
SPCS2	1.03	0.71	Both Isoforms
CCDC96	0.95	0.71	Both Isoforms
ATP2A2	1.01	0.71	Both Isoforms
TMEM50B	1.14	0.71	Both Isoforms
TMEM120A	0.63	0.71	Both Isoforms
MTOR	1.44	0.71	Both Isoforms
CD151	1.24	0.71	Both Isoforms
NDOR1	0.60	0.71	Both Isoforms
FAM64A	0.64	0.71	Both Isoforms

NDUFAF3	0.84	0.71	Both Isoforms
ACVR1	1.04	0.71	Both Isoforms
CFL1	1.25	0.71	Both Isoforms
NME4	1.21	0.72	Both Isoforms
ARFIP2	1.19	0.72	Both Isoforms
NDUFS7	0.84	0.72	Both Isoforms
ATP5I	0.94	0.72	Both Isoforms
DAG1	1.02	0.72	Both Isoforms
AKIRIN1	1.16	0.72	Both Isoforms
NACC1	1.10	0.72	Both Isoforms
NXN	0.92	0.72	Both Isoforms
TMED10	1.03	0.72	Both Isoforms
NDUFA3	0.82	0.72	Both Isoforms
BORCS8	0.98	0.72	Both Isoforms
THSD7A	2.57	0.72	Both Isoforms
ADRM1	1.24	0.72	Both Isoforms
SYNGR2	1.61	0.73	Both Isoforms
LIG4	1.36	0.73	Both Isoforms
EMC8	0.83	0.73	Both Isoforms
PIR	1.28	0.73	Both Isoforms
FAM110A	1.10	0.73	Both Isoforms
UQCRQ	1.24	0.73	Both Isoforms
LUZP1	0.93	0.73	Both Isoforms
MRPL33	0.69	0.73	Both Isoforms
ARPC5L	1.08	0.73	Both Isoforms
SLC41A2	0.94	0.73	Both Isoforms
LINC00116	0.92	0.73	Both Isoforms
SEC23B	1.06	0.73	Both Isoforms
AP2A1	0.66	0.73	Both Isoforms
MICALL2	1.19	0.73	Both Isoforms
NFKBIA	0.64	0.74	Both Isoforms
TCF7	1.20	0.74	Both Isoforms
APIP	0.80	0.74	Both Isoforms
TBC1D10A	0.79	0.74	Both Isoforms
SLC36A1	1.07	0.74	Both Isoforms
ST14	0.76	0.74	Both Isoforms
PPP1CB	1.06	0.74	Both Isoforms
TRIM37	1.35	0.74	Both Isoforms
ELMO2	1.01	0.74	Both Isoforms
PLPPR2	1.05	0.74	Both Isoforms
PRR4	0.61	0.74	Both Isoforms
ACLY	1.49	0.75	Both Isoforms
GPRC5B	2.67	0.75	Both Isoforms
PFKP	0.71	0.75	Both Isoforms
DEFB132	1.76	0.75	Both Isoforms
PTGES3L	1.28	0.75	Both Isoforms

CHFR	1.31	0.75	Both Isoforms
SRA1	1.57	0.75	Both Isoforms
C19orf48	0.68	0.75	Both Isoforms
OCEL1	1.76	0.75	Both Isoforms
ISG20	0.90	0.76	Both Isoforms
KDELR3	2.43	0.76	Both Isoforms
KL	0.65	0.76	Both Isoforms
MIEN1	1.40	0.76	Both Isoforms
SIDT2	0.69	0.76	Both Isoforms
ZNF701	0.75	0.76	Both Isoforms
SLC35E4	0.78	0.76	Both Isoforms
SYNJ1	0.68	0.76	Both Isoforms
KLHDC7A	1.48	0.76	Both Isoforms
GPAA1	0.64	0.76	Both Isoforms
MGAT2	0.64	0.77	Both Isoforms
SHC1	0.91	0.77	Both Isoforms
MYO9B	0.92	0.77	Both Isoforms
FAM81A	1.12	0.77	Both Isoforms
GALNT16	1.01	0.77	Both Isoforms
PCNXL3	0.77	0.77	Both Isoforms
VMP1	1.31	0.77	Both Isoforms
ARF4	1.49	0.77	Both Isoforms
ATP6V0D1	1.20	0.77	Both Isoforms
MAP2K6	0.72	0.77	Both Isoforms
C6orf226	1.41	0.77	Both Isoforms
SWI5	1.04	0.78	Both Isoforms
CCDC15	0.68	0.78	Both Isoforms
LMAN2	0.95	0.78	Both Isoforms
MPDU1	1.27	0.78	Both Isoforms
SYVN1	1.10	0.78	Both Isoforms
RAPH1	1.14	0.78	Both Isoforms
MELK	0.60	0.78	Both Isoforms
ZNF718	0.71	0.78	Both Isoforms
AP1S3	1.11	0.78	Both Isoforms
ETV2	0.99	0.78	Both Isoforms
CSRP1	1.39	0.78	Both Isoforms
CRYAB	1.99	0.78	Both Isoforms
TANGO2	0.85	0.78	Both Isoforms
LRRC26	2.42	0.79	Both Isoforms
PLOD3	0.90	0.79	Both Isoforms
ST3GAL1	0.79	0.79	Both Isoforms
ALYREF	0.88	0.79	Both Isoforms
AGFG2	0.89	0.79	Both Isoforms
OPN1SW	1.51	0.79	Both Isoforms
PPARGC1B	0.70	0.79	Both Isoforms
LTBR	0.77	0.79	Both Isoforms

C18orf8	1.33	0.79	Both Isoforms
C4orf48	0.84	0.79	Both Isoforms
CKS1B	0.63	0.79	Both Isoforms
YIF1A	1.18	0.80	Both Isoforms
INTS6	0.84	0.80	Both Isoforms
PIP4K2A	1.14	0.80	Both Isoforms
PLXNB3	0.83	0.80	Both Isoforms
MPPE1	0.67	0.80	Both Isoforms
PLPP1	1.35	0.80	Both Isoforms
SLC35C1	1.49	0.80	Both Isoforms
CTTN	0.89	0.80	Both Isoforms
DPH3	0.98	0.80	Both Isoforms
TRPT1	1.13	0.80	Both Isoforms
HM13	1.16	0.80	Both Isoforms
SAR1B	1.16	0.80	Both Isoforms
WWC1	0.96	0.80	Both Isoforms
LMNB2	0.68	0.81	Both Isoforms
SEC16A	1.40	0.81	Both Isoforms
PSMD1	1.11	0.81	Both Isoforms
GPS1	0.82	0.81	Both Isoforms
CLTB	0.97	0.81	Both Isoforms
CYCS	1.10	0.81	Both Isoforms
SMTN	1.57	0.81	Both Isoforms
PYCRL	0.59	0.81	Both Isoforms
TMEM55B	0.86	0.81	Both Isoforms
EPHB2	0.87	0.81	Both Isoforms
ST20	1.10	0.81	Both Isoforms
CYP4F11	0.81	0.82	Both Isoforms
NDUFS8	1.02	0.82	Both Isoforms
PDX1	1.44	0.82	Both Isoforms
ZNF682	0.88	0.82	Both Isoforms
ADAMTS7	2.79	0.82	Both Isoforms
TSPAN13	1.91	0.82	Both Isoforms
ENTPD2	1.90	0.82	Both Isoforms
ANGPTL3	1.35	0.82	Both Isoforms
PPP2R5B	0.77	0.82	Both Isoforms
TUBA1B	0.80	0.82	Both Isoforms
CDR2	1.01	0.83	Both Isoforms
CBWD5	0.71	0.83	Both Isoforms
YIF1B	1.41	0.83	Both Isoforms
ZC3H12A	1.28	0.83	Both Isoforms
C15orf65	1.13	0.83	Both Isoforms
PFKFB4	1.36	0.83	Both Isoforms
ZFP36	1.53	0.83	Both Isoforms
GDF11	1.46	0.83	Both Isoforms
DAD1	1.16	0.83	Both Isoforms

ANO4	0.78	0.83	Both Isoforms
C14orf142	1.10	0.84	Both Isoforms
GK	0.74	0.84	Both Isoforms
PRKCD	0.99	0.84	Both Isoforms
FAM73B	1.06	0.84	Both Isoforms
KIAA1919	1.07	0.84	Both Isoforms
BIN3	0.81	0.84	Both Isoforms
C2CD2L	1.29	0.84	Both Isoforms
ITGAE	0.88	0.84	Both Isoforms
COPG1	1.36	0.85	Both Isoforms
CAMK2N2	1.33	0.85	Both Isoforms
MORF4L2	0.87	0.85	Both Isoforms
LRCH4	1.06	0.85	Both Isoforms
SLC25A22	0.63	0.85	Both Isoforms
PCDHB7	1.04	0.85	Both Isoforms
ZCCHC6	1.09	0.85	Both Isoforms
SLC25A44	1.04	0.85	Both Isoforms
B4GALNT1	1.72	0.85	Both Isoforms
GCAT	0.73	0.85	Both Isoforms
RRP36	1.28	0.85	Both Isoforms
TM9SF1	1.28	0.86	Both Isoforms
MED31	0.85	0.86	Both Isoforms
SPAG4	1.30	0.86	Both Isoforms
SH3TC2	0.61	0.86	Both Isoforms
GPR89B	0.81	0.86	Both Isoforms
MSTO1	0.82	0.86	Both Isoforms
EDEM3	0.79	0.86	Both Isoforms
MAPRE3	1.22	0.86	Both Isoforms
RHBDF1	1.15	0.86	Both Isoforms
PLCXD2	1.52	0.86	Both Isoforms
PLEKHG2	1.89	0.87	Both Isoforms
PIP4K2C	1.13	0.87	Both Isoforms
EPS8L2	0.77	0.87	Both Isoforms
C7orf43	1.12	0.87	Both Isoforms
PVRL1	1.68	0.88	Both Isoforms
AEN	1.08	0.88	Both Isoforms
RHBDD3	1.09	0.88	Both Isoforms
TTC12	1.48	0.88	Both Isoforms
PSENEN	0.98	0.88	Both Isoforms
TCEAL3	1.85	0.88	Both Isoforms
FAM214B	1.50	0.88	Both Isoforms
SLC38A10	0.79	0.88	Both Isoforms
NOL12	0.69	0.89	Both Isoforms
TXNDC11	1.39	0.89	Both Isoforms
BBIP1	0.90	0.89	Both Isoforms
CHEK1	0.61	0.89	Both Isoforms

IDE	1.48	0.89	Both Isoforms
EVI5L	1.21	0.90	Both Isoforms
AP4E1	1.05	0.90	Both Isoforms
CYB5A	0.68	0.90	Both Isoforms
C17orf62	1.93	0.90	Both Isoforms
SLC2A5	3.85	0.90	Both Isoforms
AUNIP	0.61	0.90	Both Isoforms
ZNF761	1.47	0.90	Both Isoforms
SGK1	2.17	0.90	Both Isoforms
MTFR2	0.72	0.90	Both Isoforms
ABCC1	0.96	0.90	Both Isoforms
FAM43A	0.91	0.90	Both Isoforms
SELK	1.11	0.90	Both Isoforms
P4HA2	1.98	0.90	Both Isoforms
MFSD5	1.42	0.91	Both Isoforms
MAP2K4	0.77	0.91	Both Isoforms
POLR3K	1.02	0.91	Both Isoforms
GPC2	2.48	0.91	Both Isoforms
FDPS	1.42	0.91	Both Isoforms
C19orf73	0.87	0.91	Both Isoforms
PGAM1	1.17	0.91	Both Isoforms
GSG2	0.65	0.91	Both Isoforms
RTN4IP1	0.67	0.91	Both Isoforms
TAOK2	0.95	0.91	Both Isoforms
CHPF	0.86	0.91	Both Isoforms
CCDC87	1.45	0.92	Both Isoforms
SEC13	1.20	0.92	Both Isoforms
VIMP	1.26	0.92	Both Isoforms
RASSF7	1.16	0.92	Both Isoforms
MON2	0.61	0.92	Both Isoforms
B3GAT3	1.06	0.92	Both Isoforms
CDYL2	1.43	0.92	Both Isoforms
YIPF1	2.19	0.92	Both Isoforms
RCAN1	0.97	0.92	Both Isoforms
SRPRB	0.80	0.92	Both Isoforms
DAP	0.82	0.92	Both Isoforms
SLC43A1	1.01	0.92	Both Isoforms
SUOX	1.19	0.93	Both Isoforms
RFX6	0.70	0.93	Both Isoforms
HSD17B7	1.03	0.93	Both Isoforms
PDXP	1.06	0.93	Both Isoforms
SLC35F6	1.30	0.93	Both Isoforms
GBF1	1.16	0.93	Both Isoforms
EPS8L1	1.94	0.93	Both Isoforms
TAF13	1.24	0.93	Both Isoforms
OCRL	0.76	0.93	Both Isoforms

JMJD6	1.08	0.93	Both Isoforms
SH3D21	1.16	0.93	Both Isoforms
FDFT1	1.33	0.93	Both Isoforms
CDK2	1.44	0.93	Both Isoforms
ENTPD7	1.64	0.94	Both Isoforms
CMTM3	1.19	0.94	Both Isoforms
ATP6V0B	1.52	0.94	Both Isoforms
CCDC107	1.00	0.94	Both Isoforms
TPI1	0.64	0.94	Both Isoforms
GOT1	1.05	0.94	Both Isoforms
FZD1	1.37	0.94	Both Isoforms
HIST1H2AI	0.82	0.95	Both Isoforms
CENPO	0.94	0.95	Both Isoforms
TUBA4A	1.22	0.95	Both Isoforms
ARHGEF18	0.92	0.95	Both Isoforms
MAGT1	1.16	0.95	Both Isoforms
FKBP2	1.35	0.95	Both Isoforms
KCNN3	1.26	0.95	Both Isoforms
GORASP1	1.23	0.95	Both Isoforms
CYP51A1	0.89	0.95	Both Isoforms
FECH	0.66	0.96	Both Isoforms
NDUFS6	1.17	0.96	Both Isoforms
CXCL8	1.69	0.96	Both Isoforms
ORAI2	1.08	0.96	Both Isoforms
KLK4	1.85	0.96	Both Isoforms
MCM6	0.78	0.96	Both Isoforms
ATP6V0E1	1.07	0.97	Both Isoforms
LRRC45	1.62	0.97	Both Isoforms
MSRA	1.34	0.97	Both Isoforms
STRA13	1.73	0.97	Both Isoforms
SLC2A6	1.35	0.97	Both Isoforms
CFAP53	0.67	0.97	Both Isoforms
DDT	1.53	0.97	Both Isoforms
PRDM16	1.80	0.97	Both Isoforms
MYDGF	1.51	0.97	Both Isoforms
C2orf76	1.37	0.97	Both Isoforms
FITM2	1.22	0.97	Both Isoforms
MCM8	0.89	0.98	Both Isoforms
USP43	0.59	0.98	Both Isoforms
USB1	1.59	0.98	Both Isoforms
PHTF1	0.99	0.98	Both Isoforms
CHP1	1.48	0.98	Both Isoforms
TMED9	1.58	0.98	Both Isoforms
FANCB	0.91	0.98	Both Isoforms
RPN2	1.31	0.98	Both Isoforms
PLIN2	0.84	0.98	Both Isoforms

MIDN	1.15	0.98	Both Isoforms
KRT10	1.22	0.98	Both Isoforms
CENPM	0.73	0.98	Both Isoforms
SNCG	1.70	0.98	Both Isoforms
PDIA6	1.35	0.98	Both Isoforms
STT3A	1.36	0.99	Both Isoforms
ARL13B	1.29	0.99	Both Isoforms
WDR34	0.71	0.99	Both Isoforms
GNAO1	1.06	0.99	Both Isoforms
SSR1	1.24	0.99	Both Isoforms
PHKG2	1.00	0.99	Both Isoforms
FAM151B	1.00	0.99	Both Isoforms
DUS1L	0.80	0.99	Both Isoforms
P2RY2	1.68	0.99	Both Isoforms
LDLRAD3	0.92	0.99	Both Isoforms
ABHD11	0.97	0.99	Both Isoforms
ZFAND2B	1.07	0.99	Both Isoforms
SHROOM3	1.03	1.00	Both Isoforms
DNAJB9	1.28	1.00	Both Isoforms
TRAPPC2P1	1.15	1.00	Both Isoforms
LIN52	0.84	1.00	Both Isoforms
DNMT1	0.77	1.00	Both Isoforms
PVRL2	1.89	1.00	Both Isoforms
CST1	3.06	1.00	Both Isoforms
ULBP2	1.06	1.00	Both Isoforms
CNKSR2	1.32	1.00	Both Isoforms
ERCC6L	0.97	1.00	Both Isoforms
PIAS1	0.81	1.00	Both Isoforms
TMEM53	1.02	1.01	Both Isoforms
PLEKHO2	2.07	1.01	Both Isoforms
DHRS2	1.59	1.01	Both Isoforms
UFD1L	1.23	1.01	Both Isoforms
IKZF1	2.77	1.01	Both Isoforms
MCM3	0.71	1.01	Both Isoforms
C20orf24	1.44	1.01	Both Isoforms
RTEL1	0.74	1.01	Both Isoforms
PUSL1	1.15	1.01	Both Isoforms
TIMELESS	0.68	1.01	Both Isoforms
XBP1	0.98	1.02	Both Isoforms
HOXA11	0.99	1.02	Both Isoforms
METRNL	1.39	1.02	Both Isoforms
KRT18	1.52	1.02	Both Isoforms
SAFB2	0.81	1.02	Both Isoforms
COPE	1.47	1.02	Both Isoforms
UGGT1	1.15	1.02	Both Isoforms
PDIA3	1.51	1.03	Both Isoforms

GALNS	0.97	1.03	Both Isoforms
SLC25A20	1.90	1.03	Both Isoforms
DOLK	1.62	1.03	Both Isoforms
EIF2AK3	1.10	1.04	Both Isoforms
CTB-55O6.8	2.12	1.04	Both Isoforms
TMEM214	1.48	1.04	Both Isoforms
NUDT16L1	1.27	1.04	Both Isoforms
PNPLA8	0.66	1.04	Both Isoforms
SLC52A2	1.80	1.04	Both Isoforms
KIAA0232	1.03	1.04	Both Isoforms
GLRX	1.11	1.04	Both Isoforms
H2AFZ	0.67	1.05	Both Isoforms
UBE2T	0.89	1.05	Both Isoforms
CTXN1	1.41	1.05	Both Isoforms
MBOAT2	1.36	1.05	Both Isoforms
C19orf57	0.58	1.06	Both Isoforms
LYZ	1.79	1.06	Both Isoforms
CHAF1B	0.66	1.06	Both Isoforms
AK1	1.57	1.06	Both Isoforms
PRSS36	0.70	1.06	Both Isoforms
CCDC141	3.00	1.07	Both Isoforms
PACSIN2	1.08	1.07	Both Isoforms
ORMDL2	1.60	1.07	Both Isoforms
A2M	1.08	1.07	Both Isoforms
AQP3	0.69	1.07	Both Isoforms
ACSM1	2.63	1.07	Both Isoforms
XRCC3	0.64	1.07	Both Isoforms
POLR2L	1.34	1.07	Both Isoforms
DNAJC10	2.10	1.07	Both Isoforms
CHPF2	1.35	1.07	Both Isoforms
JAG1	1.15	1.07	Both Isoforms
ORC1	0.76	1.07	Both Isoforms
CHRNA5	0.80	1.07	Both Isoforms
DLX1	1.26	1.08	Both Isoforms
MORN2	0.92	1.08	Both Isoforms
CALR	1.41	1.08	Both Isoforms
EHD4	1.56	1.08	Both Isoforms
SERTAD1	1.91	1.08	Both Isoforms
CDKN2D	0.94	1.09	Both Isoforms
MERTK	1.22	1.09	Both Isoforms
BCAR1	1.55	1.09	Both Isoforms
ROMO1	1.26	1.09	Both Isoforms
POP5	0.80	1.10	Both Isoforms
RHAG	5.72	1.10	Both Isoforms
GPS2	1.13	1.10	Both Isoforms
GSTT2B	1.25	1.10	Both Isoforms

SRP19	1.09	1.10	Both Isoforms
SURF4	1.64	1.10	Both Isoforms
B3GNT2	2.23	1.11	Both Isoforms
UNC13B	0.79	1.11	Both Isoforms
RHBDD2	1.31	1.11	Both Isoforms
SRPR	1.39	1.11	Both Isoforms
BEAN1	1.73	1.11	Both Isoforms
MASTL	0.78	1.11	Both Isoforms
NUDT22	1.46	1.11	Both Isoforms
ENDOG	0.88	1.11	Both Isoforms
GINS1	0.65	1.11	Both Isoforms
PPIB	1.37	1.11	Both Isoforms
ANKRD54	0.62	1.11	Both Isoforms
GNRH2	1.45	1.13	Both Isoforms
SLC39A3	1.35	1.13	Both Isoforms
TNFAIP8	1.43	1.13	Both Isoforms
PRSS8	0.86	1.13	Both Isoforms
RFPL2	3.69	1.14	Both Isoforms
DNAJB5	2.17	1.14	Both Isoforms
CCNE2	1.19	1.14	Both Isoforms
SEC14L5	1.95	1.14	Both Isoforms
CORO1B	2.71	1.14	Both Isoforms
CKLF-CMTM1	1.74	1.14	Both Isoforms
IL1R1	0.88	1.15	Both Isoforms
SLCO3A1	1.85	1.15	Both Isoforms
HSP90B1	1.34	1.15	Both Isoforms
GPR75	1.14	1.15	Both Isoforms
ATP1B1	1.25	1.15	Both Isoforms
HES4	2.41	1.15	Both Isoforms
SREBF1	1.63	1.15	Both Isoforms
MVK	1.34	1.15	Both Isoforms
SEMA3C	0.97	1.15	Both Isoforms
FAM129A	1.55	1.16	Both Isoforms
PTPRM	1.51	1.16	Both Isoforms
TLCD2	1.15	1.16	Both Isoforms
ELOVL6	1.25	1.16	Both Isoforms
B3GAT1	1.26	1.17	Both Isoforms
TMEM104	1.36	1.17	Both Isoforms
SGK2	0.73	1.17	Both Isoforms
SAMD12	1.61	1.17	Both Isoforms
SH3BGRL3	1.49	1.17	Both Isoforms
NR4A1	1.42	1.17	Both Isoforms
MSMO1	1.51	1.17	Both Isoforms
FKBP11	1.41	1.17	Both Isoforms
SLC35B1	1.54	1.17	Both Isoforms
PCYT2	1.18	1.17	Both Isoforms

GIPC1	1.17	1.18	Both Isoforms
LRRC46	0.83	1.18	Both Isoforms
LDLR	2.05	1.18	Both Isoforms
CCDC167	1.40	1.18	Both Isoforms
KBTBD8	1.25	1.18	Both Isoforms
GCH1	1.35	1.18	Both Isoforms
CRACR2B	1.13	1.18	Both Isoforms
GADD45G	2.91	1.19	Both Isoforms
ARRDC1	1.27	1.19	Both Isoforms
KCNJ11	1.12	1.19	Both Isoforms
SFXN5	2.00	1.19	Both Isoforms
PSMC3IP	1.07	1.19	Both Isoforms
C16orf59	0.93	1.19	Both Isoforms
LRRC59	1.87	1.20	Both Isoforms
GMPPA	1.51	1.20	Both Isoforms
DHCR24	2.13	1.20	Both Isoforms
SLC1A4	0.97	1.20	Both Isoforms
SEC61G	1.56	1.20	Both Isoforms
PCNA	0.98	1.20	Both Isoforms
PREB	1.52	1.20	Both Isoforms
EGFR	0.96	1.20	Both Isoforms
CCDC57	0.84	1.20	Both Isoforms
SERINC2	3.32	1.21	Both Isoforms
ZWINT	0.69	1.21	Both Isoforms
KLHL2	1.34	1.21	Both Isoforms
TMEM110	1.43	1.21	Both Isoforms
SMPD1	1.44	1.21	Both Isoforms
DTX2	1.09	1.21	Both Isoforms
ISG15	1.92	1.22	Both Isoforms
P4HB	1.70	1.22	Both Isoforms
GUCA2A	2.10	1.23	Both Isoforms
CDK2AP2	1.48	1.23	Both Isoforms
BMPR1B	1.51	1.23	Both Isoforms
UBTD1	1.47	1.23	Both Isoforms
LACTB	1.11	1.23	Both Isoforms
PEA15	1.28	1.23	Both Isoforms
ATG4A	1.62	1.23	Both Isoforms
COQ10B	1.81	1.24	Both Isoforms
UNC5C	1.00	1.24	Both Isoforms
MCM5	1.07	1.24	Both Isoforms
SLC25A33	0.98	1.24	Both Isoforms
LRRC8A	0.77	1.24	Both Isoforms
TUBB4B	1.50	1.24	Both Isoforms
CLEC16A	0.72	1.24	Both Isoforms
PICK1	1.70	1.25	Both Isoforms
AHCYL2	1.18	1.25	Both Isoforms

CETN2	0.72	1.25	Both Isoforms
ABHD3	1.67	1.25	Both Isoforms
FAM58A	0.76	1.26	Both Isoforms
KLF15	0.65	1.26	Both Isoforms
FKBP1B	2.25	1.26	Both Isoforms
PPARGC1A	0.67	1.26	Both Isoforms
DONSON	0.96	1.26	Both Isoforms
ALDOC	0.94	1.27	Both Isoforms
HSPA5	1.87	1.27	Both Isoforms
POLA2	0.98	1.27	Both Isoforms
SLC45A3	2.28	1.27	Both Isoforms
AGR2	3.34	1.27	Both Isoforms
PEBP4	2.18	1.27	Both Isoforms
CREB3L2	1.49	1.27	Both Isoforms
SPCS3	1.60	1.28	Both Isoforms
DNAJB11	1.76	1.28	Both Isoforms
SSR3	1.48	1.28	Both Isoforms
USP5	0.60	1.28	Both Isoforms
MVD	1.47	1.28	Both Isoforms
TBX15	4.18	1.28	Both Isoforms
SEC61B	1.67	1.28	Both Isoforms
ABRACL	1.07	1.29	Both Isoforms
DDIAS	1.74	1.29	Both Isoforms
HIPK2	1.93	1.29	Both Isoforms
RAB26	1.41	1.29	Both Isoforms
PLAUR	1.72	1.29	Both Isoforms
SDK1	1.24	1.30	Both Isoforms
RPN1	1.32	1.30	Both Isoforms
2-Mar	2.22	1.30	Both Isoforms
SLC5A4	3.24	1.30	Both Isoforms
SEC61A1	1.47	1.31	Both Isoforms
ST6GALNAC1	1.22	1.31	Both Isoforms
ARPC1B	2.16	1.31	Both Isoforms
IVD	0.61	1.31	Both Isoforms
ADAM9	0.89	1.31	Both Isoforms
HMGXB3	1.67	1.31	Both Isoforms
POP1	0.92	1.31	Both Isoforms
MAN2B2	1.28	1.31	Both Isoforms
SLC22A18	1.66	1.32	Both Isoforms
NNAT	0.76	1.32	Both Isoforms
CRIP2	1.51	1.32	Both Isoforms
ABCB8	1.07	1.32	Both Isoforms
SLCO4A1	0.77	1.33	Both Isoforms
DNAJC3	2.01	1.33	Both Isoforms
MSRB1	0.98	1.33	Both Isoforms
ASRGL1	2.30	1.33	Both Isoforms

HIST2H2BE	1.16	1.34	Both Isoforms
FANCA	0.93	1.34	Both Isoforms
RAPGEF3	2.40	1.34	Both Isoforms
DGKA	1.53	1.34	Both Isoforms
CPE	0.70	1.34	Both Isoforms
HOMER2	1.55	1.34	Both Isoforms
PDIA4	1.98	1.35	Both Isoforms
GCHFR	1.61	1.35	Both Isoforms
GAL	0.66	1.35	Both Isoforms
DNAJB1	1.02	1.35	Both Isoforms
WDFY2	0.87	1.35	Both Isoforms
SLFN13	1.00	1.35	Both Isoforms
ACADL	0.97	1.36	Both Isoforms
BRCA1	0.61	1.36	Both Isoforms
SNAI2	1.82	1.36	Both Isoforms
ACAT2	1.40	1.36	Both Isoforms
NPIPA5	2.55	1.37	Both Isoforms
GCC2	1.27	1.37	Both Isoforms
KNSTRN	0.95	1.37	Both Isoforms
SLC22A3	1.00	1.37	Both Isoforms
SHCBP1	1.37	1.37	Both Isoforms
KCNAB1	0.92	1.37	Both Isoforms
ZBTB7B	1.68	1.37	Both Isoforms
C15orf52	1.35	1.38	Both Isoforms
ARSA	1.97	1.38	Both Isoforms
LAT2	2.29	1.38	Both Isoforms
SLC22A31	0.93	1.38	Both Isoforms
CST3	1.22	1.38	Both Isoforms
NCAPD3	4.14	1.38	Both Isoforms
KITLG	2.04	1.39	Both Isoforms
TMEM38A	0.63	1.40	Both Isoforms
GINS3	1.10	1.40	Both Isoforms
CROT	0.68	1.40	Both Isoforms
SOCS2	1.93	1.41	Both Isoforms
E2F8	0.78	1.41	Both Isoforms
CST2	3.10	1.41	Both Isoforms
CCDC102B	0.64	1.41	Both Isoforms
DRD2	1.47	1.41	Both Isoforms
GINS4	1.01	1.41	Both Isoforms
ORC6	1.24	1.42	Both Isoforms
TRIM62	1.82	1.42	Both Isoforms
SEC11C	2.49	1.42	Both Isoforms
SOX9	1.47	1.42	Both Isoforms
AFMID	0.73	1.42	Both Isoforms
SLC4A7	1.17	1.42	Both Isoforms
FAM13C	2.29	1.43	Both Isoforms

TFEB	0.97	1.43	Both Isoforms
RBM24	3.72	1.43	Both Isoforms
LONRF1	1.07	1.43	Both Isoforms
SLC22A17	1.12	1.43	Both Isoforms
AP1B1	1.32	1.44	Both Isoforms
PKMYT1	1.01	1.44	Both Isoforms
SYTL4	1.01	1.45	Both Isoforms
CCDC163P	1.10	1.45	Both Isoforms
SLC26A6	0.88	1.45	Both Isoforms
CDC7	1.10	1.46	Both Isoforms
WDR66	2.04	1.46	Both Isoforms
GAS2L1	1.29	1.46	Both Isoforms
ARHGEF37	0.73	1.46	Both Isoforms
STK39	1.54	1.47	Both Isoforms
IDI1	2.22	1.47	Both Isoforms
HGH1	1.05	1.47	Both Isoforms
WFS1	1.47	1.47	Both Isoforms
MCM4	1.00	1.47	Both Isoforms
TUBG1	2.40	1.47	Both Isoforms
DHCR7	1.17	1.48	Both Isoforms
EID3	0.90	1.48	Both Isoforms
CEBPD	0.68	1.48	Both Isoforms
BLM	0.73	1.49	Both Isoforms
MPV17L2	1.59	1.49	Both Isoforms
PGP	1.91	1.49	Both Isoforms
TSC22D1	1.36	1.49	Both Isoforms
SPSB2	1.40	1.50	Both Isoforms
TMEM169	1.14	1.50	Both Isoforms
CITED2	0.59	1.50	Both Isoforms
EXO1	0.87	1.51	Both Isoforms
ASF1B	0.82	1.52	Both Isoforms
C15orf62	0.64	1.52	Both Isoforms
CDO1	0.93	1.52	Both Isoforms
HAUS8	0.99	1.53	Both Isoforms
MAGEH1	1.53	1.53	Both Isoforms
EMP1	2.79	1.53	Both Isoforms
CRLF1	0.99	1.53	Both Isoforms
CBWD1	0.79	1.53	Both Isoforms
ADGRG2	2.16	1.54	Both Isoforms
CMTM1	1.37	1.54	Both Isoforms
PAQR4	2.33	1.54	Both Isoforms
PIK3R3	1.28	1.55	Both Isoforms
NDRG1	2.30	1.55	Both Isoforms
POLE2	1.54	1.55	Both Isoforms
PLEKHG3	0.92	1.55	Both Isoforms
B4GALT1	1.33	1.55	Both Isoforms

SYNC	1.83	1.55	Both Isoforms
MANF	2.28	1.55	Both Isoforms
PLA2G4F	2.65	1.55	Both Isoforms
TBXAS1	1.90	1.56	Both Isoforms
EFCAB12	3.08	1.56	Both Isoforms
SUCO	0.86	1.57	Both Isoforms
PCDH1	2.31	1.57	Both Isoforms
EAF2	2.85	1.57	Both Isoforms
SMS	2.11	1.57	Both Isoforms
HIST1H3C	1.84	1.58	Both Isoforms
NNMT	1.40	1.58	Both Isoforms
PRADC1	1.79	1.58	Both Isoforms
SLF1	0.80	1.59	Both Isoforms
C1orf122	1.48	1.60	Both Isoforms
CMC2	1.98	1.60	Both Isoforms
MAF	1.70	1.60	Both Isoforms
MTFP1	1.83	1.61	Both Isoforms
USP18	1.12	1.61	Both Isoforms
SLC6A17	0.68	1.61	Both Isoforms
HYOU1	1.95	1.61	Both Isoforms
RAD51	1.25	1.62	Both Isoforms
RRBP1	1.95	1.62	Both Isoforms
PRRG4	1.27	1.62	Both Isoforms
ACSL3	3.05	1.63	Both Isoforms
HOXA1	2.68	1.63	Both Isoforms
B3GLCT	0.92	1.65	Both Isoforms
SLC15A2	2.75	1.65	Both Isoforms
WBSCR17	1.71	1.67	Both Isoforms
EBP	2.03	1.67	Both Isoforms
WDR76	1.50	1.68	Both Isoforms
UHRF1	1.48	1.68	Both Isoforms
MICAL2	0.63	1.68	Both Isoforms
ROBO1	0.77	1.70	Both Isoforms
IGFLR1	1.34	1.70	Both Isoforms
CCDC189	0.82	1.70	Both Isoforms
INSIG1	2.31	1.72	Both Isoforms
DCXR	1.60	1.72	Both Isoforms
RELT	1.65	1.73	Both Isoforms
ZBTB16	0.83	1.74	Both Isoforms
LIPG	1.70	1.74	Both Isoforms
LIFR	1.63	1.75	Both Isoforms
MYO1E	1.98	1.75	Both Isoforms
HMGCS1	1.91	1.75	Both Isoforms
TBC1D8B	0.79	1.76	Both Isoforms
HARBI1	1.61	1.76	Both Isoforms
ATP2B4	3.35	1.76	Both Isoforms

TUBA3C	2.26	1.78	Both Isoforms
TK1	1.18	1.78	Both Isoforms
RDH10	1.28	1.78	Both Isoforms
CNIH2	1.29	1.78	Both Isoforms
OLFM1	1.94	1.79	Both Isoforms
KRT8	2.21	1.80	Both Isoforms
MYBL2	1.36	1.80	Both Isoforms
MUM1L1	1.34	1.80	Both Isoforms
RAET1E	3.13	1.80	Both Isoforms
RAD54L	1.22	1.81	Both Isoforms
SMOC2	1.89	1.81	Both Isoforms
ANK1	2.43	1.82	Both Isoforms
CDC25A	1.40	1.82	Both Isoforms
KCNH6	1.66	1.82	Both Isoforms
CENPN	2.16	1.83	Both Isoforms
COX17	1.87	1.83	Both Isoforms
SLC20A1	1.30	1.83	Both Isoforms
PDCD4	2.05	1.84	Both Isoforms
NUDT11	0.79	1.84	Both Isoforms
DTL	1.56	1.84	Both Isoforms
FAM189A2	2.69	1.85	Both Isoforms
DERL3	1.49	1.85	Both Isoforms
STYK1	1.42	1.87	Both Isoforms
KRT15	2.94	1.87	Both Isoforms
ITPR3	1.59	1.88	Both Isoforms
VDR	0.68	1.88	Both Isoforms
GAS7	1.56	1.88	Both Isoforms
STRIP2	1.74	1.88	Both Isoforms
DOCK11	2.40	1.89	Both Isoforms
ATP1A1	2.34	1.90	Both Isoforms
TRIB2	1.72	1.91	Both Isoforms
CHRNA2	1.57	1.91	Both Isoforms
RRM2	1.07	1.91	Both Isoforms
HS3ST4	1.28	1.91	Both Isoforms
HPGD	1.38	1.92	Both Isoforms
CYP1B1	2.20	1.93	Both Isoforms
TMEM204	2.17	1.94	Both Isoforms
LOX	1.18	1.94	Both Isoforms
NUCB2	2.58	1.95	Both Isoforms
CDC45	1.46	1.96	Both Isoforms
C1orf87	3.20	1.97	Both Isoforms
HACD2	1.13	1.97	Both Isoforms
AFF3	1.26	1.97	Both Isoforms
PPP1R3G	1.34	1.98	Both Isoforms
ELOVL5	1.62	1.99	Both Isoforms
PLEKHD1	2.57	2.00	Both Isoforms

CSRNP1	1.25	2.00	Both Isoforms
ANXA13	2.83	2.01	Both Isoforms
EPHA5	1.72	2.01	Both Isoforms
CPM	1.62	2.01	Both Isoforms
SMOX	0.86	2.01	Both Isoforms
FAM111B	1.39	2.02	Both Isoforms
E2F2	1.92	2.03	Both Isoforms
OSGIN1	2.74	2.04	Both Isoforms
GMPPB	2.52	2.04	Both Isoforms
SLCO2A1	2.17	2.05	Both Isoforms
JADE1	1.05	2.05	Both Isoforms
FEN1	1.61	2.05	Both Isoforms
CDC6	2.00	2.06	Both Isoforms
B2M	0.83	2.07	Both Isoforms
MYO1G	2.82	2.08	Both Isoforms
TESC	3.25	2.08	Both Isoforms
LAMA1	3.46	2.09	Both Isoforms
ZNF367	1.71	2.09	Both Isoforms
KRT19	1.58	2.09	Both Isoforms
ASMTL	1.93	2.10	Both Isoforms
CKLF	2.31	2.11	Both Isoforms
SLC27A3	1.45	2.11	Both Isoforms
CDKN1A	1.73	2.12	Both Isoforms
ACPT	1.97	2.14	Both Isoforms
GINS2	1.12	2.14	Both Isoforms
RIBC2	1.19	2.15	Both Isoforms
SLC37A2	2.78	2.15	Both Isoforms
PER1	1.03	2.15	Both Isoforms
EVA1C	1.39	2.15	Both Isoforms
ANGPT4	2.81	2.16	Both Isoforms
AMACR	3.35	2.16	Both Isoforms
PFKFB2	2.12	2.16	Both Isoforms
C22orf42	7.11	2.16	Both Isoforms
SPOCK2	1.87	2.17	Both Isoforms
PRR15L	1.98	2.17	Both Isoforms
WLS	0.80	2.18	Both Isoforms
CLSPN	1.84	2.18	Both Isoforms
KLK3	3.54	2.19	Both Isoforms
SEC24D	2.61	2.19	Both Isoforms
ELL2	1.11	2.20	Both Isoforms
AZGP1	1.84	2.20	Both Isoforms
FAM196B	1.36	2.20	Both Isoforms
ATAD2	1.18	2.20	Both Isoforms
IL18R1	1.18	2.24	Both Isoforms
TMEM8A	3.15	2.24	Both Isoforms
E2F1	2.15	2.24	Both Isoforms

MYO16	3.87	2.26	Both Isoforms
CECR6	2.41	2.26	Both Isoforms
STARD4	3.00	2.27	Both Isoforms
FICD	2.35	2.30	Both Isoforms
MCM10	1.85	2.31	Both Isoforms
TUBA3E	2.20	2.32	Both Isoforms
C9orf66	1.45	2.33	Both Isoforms
SDF2L1	2.99	2.33	Both Isoforms
KCNH2	0.98	2.33	Both Isoforms
PATE2	1.41	2.37	Both Isoforms
FSTL4	0.66	2.37	Both Isoforms
LGI3	2.11	2.42	Both Isoforms
CPEB2	1.52	2.43	Both Isoforms
CRELD2	2.31	2.43	Both Isoforms
FADS3	1.26	2.46	Both Isoforms
CA2	1.37	2.46	Both Isoforms
GPR153	0.64	2.46	Both Isoforms
PRPS2	0.61	2.47	Both Isoforms
RPRM	1.18	2.53	Both Isoforms
IRAK3	1.85	2.56	Both Isoforms
SPOCK1	1.95	2.56	Both Isoforms
PHOSPHO1	5.08	2.58	Both Isoforms
WIPI1	3.28	2.59	Both Isoforms
TGM2	1.71	2.60	Both Isoforms
CTNNA2	2.36	2.60	Both Isoforms
FGFR1	1.36	2.61	Both Isoforms
DDIT4	2.05	2.61	Both Isoforms
GNMT	1.94	2.62	Both Isoforms
TMEM47	1.75	2.63	Both Isoforms
ZNF812	3.37	2.64	Both Isoforms
NDRG4	1.11	2.65	Both Isoforms
CKB	2.29	2.65	Both Isoforms
ARMC12	1.47	2.66	Both Isoforms
FAM107A	1.88	2.67	Both Isoforms
CAPN14	1.47	2.67	Both Isoforms
TUBA3D	2.94	2.70	Both Isoforms
FA2H	2.17	2.70	Both Isoforms
BHLHA15	3.87	2.70	Both Isoforms
CYP4F8	1.83	2.71	Both Isoforms
HSPH1	0.71	2.72	Both Isoforms
DBI	3.19	2.74	Both Isoforms
THSD1	2.99	2.77	Both Isoforms
MAP1B	1.80	2.79	Both Isoforms
GLIS3	1.24	2.81	Both Isoforms
KCNMA1	3.05	2.81	Both Isoforms
DYNC1I1	2.18	2.81	Both Isoforms

	CST4	4.37	2.84	Both Isoforms
	HSPA2	0.74	2.86	Both Isoforms
	AKAP12	0.93	2.91	Both Isoforms
	MAP3K6	2.43	2.93	Both Isoforms
	SLC26A2	2.61	2.95	Both Isoforms
	FAM166B	0.90	2.96	Both Isoforms
	CLGN	1.30	2.97	Both Isoforms
	NSDHL	1.45	3.01	Both Isoforms
	SMIM24	2.50	3.03	Both Isoforms
	FADS2	2.76	3.06	Both Isoforms
	ELMO1	2.97	3.08	Both Isoforms
	FASN	2.27	3.15	Both Isoforms
	IL31RA	1.17	3.16	Both Isoforms
	MFSD2A	2.26	3.21	Both Isoforms
	TMEM61	1.42	3.22	Both Isoforms
	RBM20	3.35	3.25	Both Isoforms
	TNFRSF1A	0.60	3.27	Both Isoforms
	PDE6A	1.12	3.28	Both Isoforms
	TDRD9	0.92	3.31	Both Isoforms
	RHOB	1.67	3.37	Both Isoforms
	TIPARP	1.27	3.38	Both Isoforms
	CHST2	1.59	3.46	Both Isoforms
	SP6	2.48	3.51	Both Isoforms
	ABCC8	0.64	3.52	Both Isoforms
	NEK10	2.12	3.56	Both Isoforms
	SLC2A3	5.06	3.59	Both Isoforms
	FAM196A	0.62	3.59	Both Isoforms
	FADS1	2.33	3.60	Both Isoforms
	HES1	2.62	3.62	Both Isoforms
	HS6ST1	1.78	3.63	Both Isoforms
	RASD1	2.17	3.64	Both Isoforms
	DNER	0.61	3.64	Both Isoforms
	SERPINF2	1.60	3.70	Both Isoforms
	NPR3	0.83	3.71	Both Isoforms
	ERRFI1	2.80	3.73	Both Isoforms
	ABHD6	1.43	3.82	Both Isoforms
	CDKN2B	1.95	3.82	Both Isoforms
	ITGA11	3.81	3.85	Both Isoforms
	SLC31A2	1.46	3.85	Both Isoforms
	PPP1R16B	0.78	3.86	Both Isoforms
	GP2	1.70	3.90	Both Isoforms
	TIMP3	1.67	3.91	Both Isoforms
	TSPYL2	1.62	3.96	Both Isoforms
	MOGAT2	2.47	3.97	Both Isoforms
	ADAMTS15	1.57	4.03	Both Isoforms
	SERPINA4	1.17	4.03	Both Isoforms

SNTG2	1.45	4.16	Both Isoforms
ABCG2	2.29	4.37	Both Isoforms
NUDT10	2.41	4.54	Both Isoforms
PLA2G4E	4.66	4.56	Both Isoforms
TMEM37	1.42	4.57	Both Isoforms
NPR1	1.89	4.57	Both Isoforms
CYP4F22	1.53	4.58	Both Isoforms
ANGPT1	1.74	4.62	Both Isoforms
EFEMP1	1.09	4.74	Both Isoforms
EDN2	0.79	4.74	Both Isoforms
TSPAN18	1.34	4.86	Both Isoforms
SULT1C4	2.15	4.91	Both Isoforms
FKBP5	4.29	4.99	Both Isoforms
DISP2	3.48	5.09	Both Isoforms
PGF	0.69	5.09	Both Isoforms
PGLYRP2	3.55	5.17	Both Isoforms
FIBCD1	0.83	5.27	Both Isoforms
PHGR1	3.74	5.31	Both Isoforms
CRISP1	7.03	5.43	Both Isoforms
DOCK8	3.55	5.49	Both Isoforms
CA4	4.10	5.62	Both Isoforms
IVL	3.95	5.68	Both Isoforms
NEFH	2.41	5.89	Both Isoforms
CD99	1.23	5.97	Both Isoforms
THBD	0.86	6.09	Both Isoforms
SPINK2	2.29	6.33	Both Isoforms
SLC38A4	4.14	6.42	Both Isoforms
CPVL	2.23	7.05	Both Isoforms
SLC1A3	3.05	7.08	Both Isoforms
ORM2	4.89	7.27	Both Isoforms
TLR7	4.52	7.32	Both Isoforms
ANGPTL5	3.58	7.35	Both Isoforms
PGC	8.31	7.42	Both Isoforms
ATP1A2	6.12	7.46	Both Isoforms
DPEP1	3.06	7.52	Both Isoforms
SLC26A3	4.96	7.55	Both Isoforms
SPINK13	2.22	7.72	Both Isoforms
SERPINA3	2.23	7.74	Both Isoforms
MORC1	4.52	7.76	Both Isoforms
KRTAP9-6	4.52	7.86	Both Isoforms
PNLIP	5.27	8.05	Both Isoforms
EBI3	2.22	8.06	Both Isoforms
GPR6	3.06	8.53	Both Isoforms
ORM1	4.74	9.00	Both Isoforms
SLC2A14	3.05	9.09	Both Isoforms
C7orf57	2.22	10.14	Both Isoforms

	PLP1	7.12	10.41	Both Isoforms
